# Supplementary material for: Deep learning the cis-regulatory code for gene expression in selected model plants
Source: Nat Commun. 2024 Apr 25;15:3488. doi: 10.1038/s41467-024-47744-0 (PMC11045779; doi:10.1038/s41467-024-47744-0)
Supplement: Supplementary file 1 — Supplementary Information [file 41467_2024_47744_MOESM1_ESM.pdf]

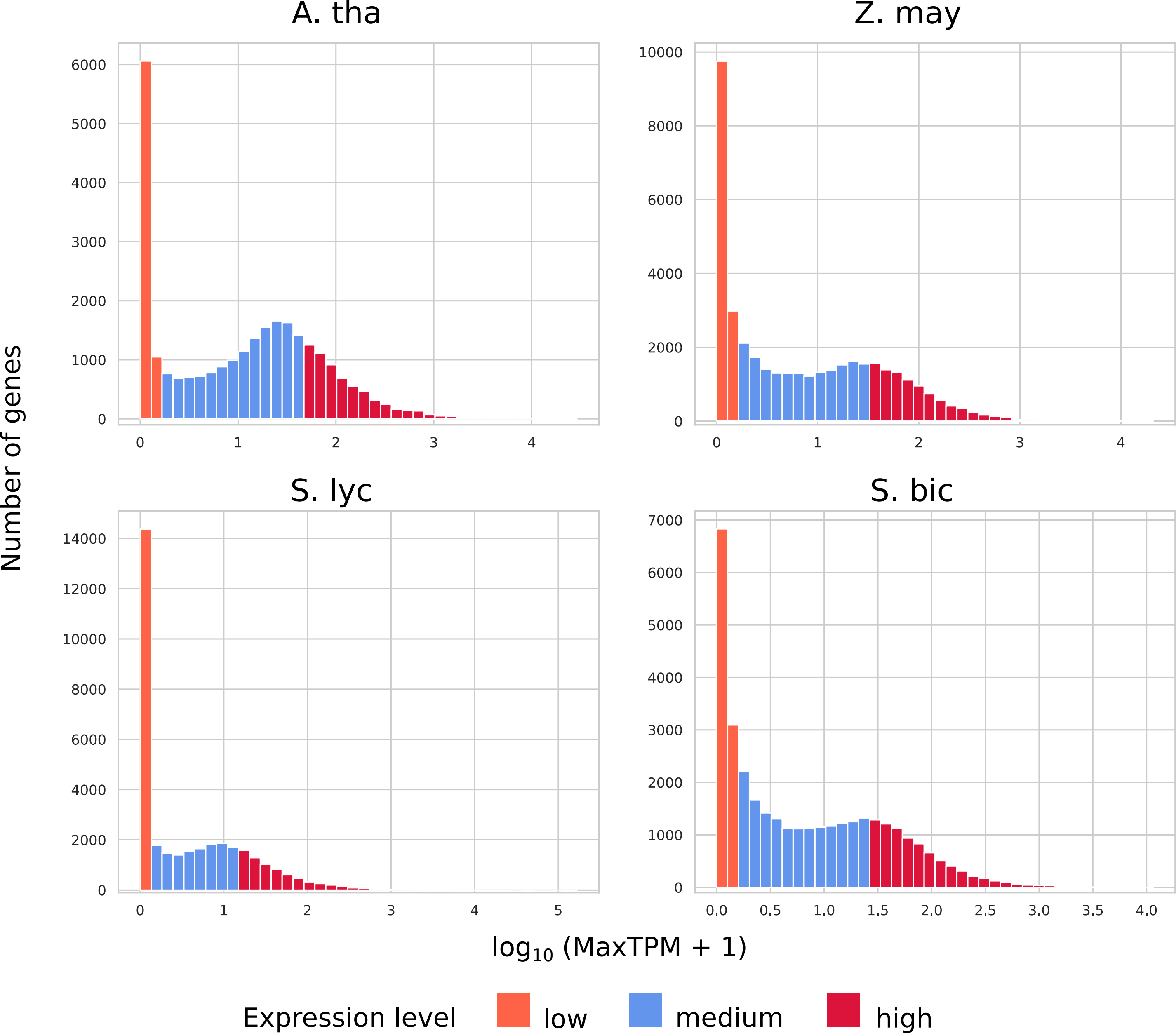

A. tha
Z. may
S. lyc
S. bic
Number of genes
log$_{10}$ (MaxTPM + 1)
Expression level
low
medium
high

Supplementary Figure 1 - Short read transcriptome samples prepared from leafs were mapped to for the four reference species *A. thaliana, S. lycopersicum, S. bicolor,* and *Z. mays*. respectively. This produced normalized expression counts in transcripts per million (TPM). For each gene, we applied a logarithmic transformation to the maximum TPM value across samples to represent the expression level. Genes were categorized into low (dark orange), medium (blue), high (red) expression classes based on the lower and upper 25% quantiles of the log10maxTPM+1 distributions. The threshold values for leaf transcript profiles of *A. thaliana, S. lycopersicum, S. bicolor,* and *Z. mays* were 0.199, 0.000, 0.153, and 0.113 log10maxTPM+1 for the lower and 1.621, 1.051, 1.389, and 1.465 log10maxTPM+1 for the higher quartile, respectively (**Supplementary Data 1**).

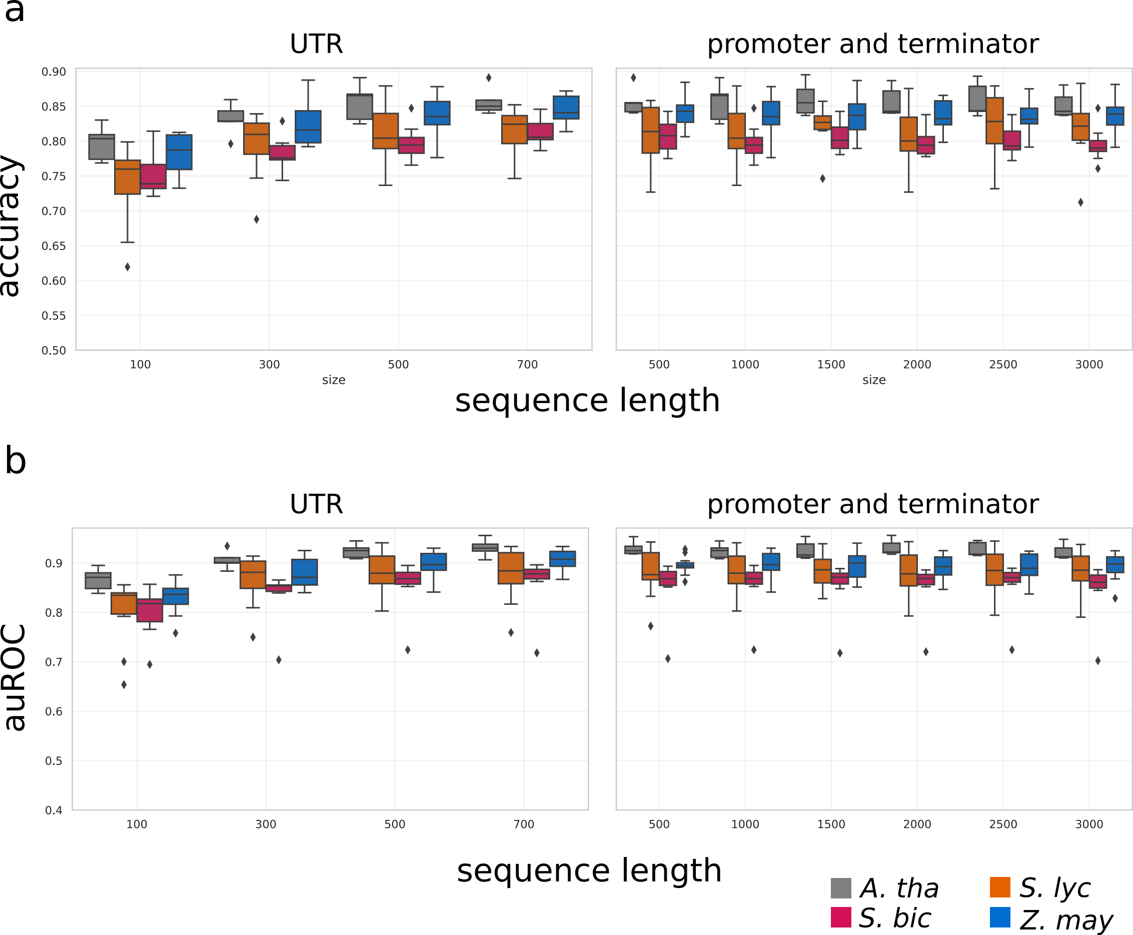

a
UTR
promoter and terminator
accuracy
0.90
0.85
0.80
0.75
0.70
0.65
0.60
0.55
0.50
100
300
500
700
size
500
1000
1500
2000
2500
3000
size
sequence length
b
UTR
promoter and terminator
auROC
0.9
0.8
0.7
0.6
0.5
0.4
100
300
500
700
500
1000
1500
2000
2500
3000
sequence length
A. tha
S. lyc
S. bic
Z. may

Supplementary Figure 2 - The comparison of model performance for different promoter, terminator and UTR lengths. Lengths of the 5’ and 3’ UTRs were varied from 100 to 700 nt while keeping the terminator and promoter lengths constant at 1000 nt. Alternatively, the 5’ and 3’ UTR lengths were kept constant at 500 nt while varying the promoter and terminator lengths from 500 to 3000 nt. Model performance in accuracy (a) and area under the receiver operating characteristic curve (b) was recorded for varied UTR (left) and varied promoter and terminator lengths (right). Boxplot depicting sample characteristics using the 25th, 50th (median), and 75th percentiles along with the interquartile range, representing the central 50% of the data. Whiskers extend from the minimum to maximum values, showcasing the spread of the dataset.

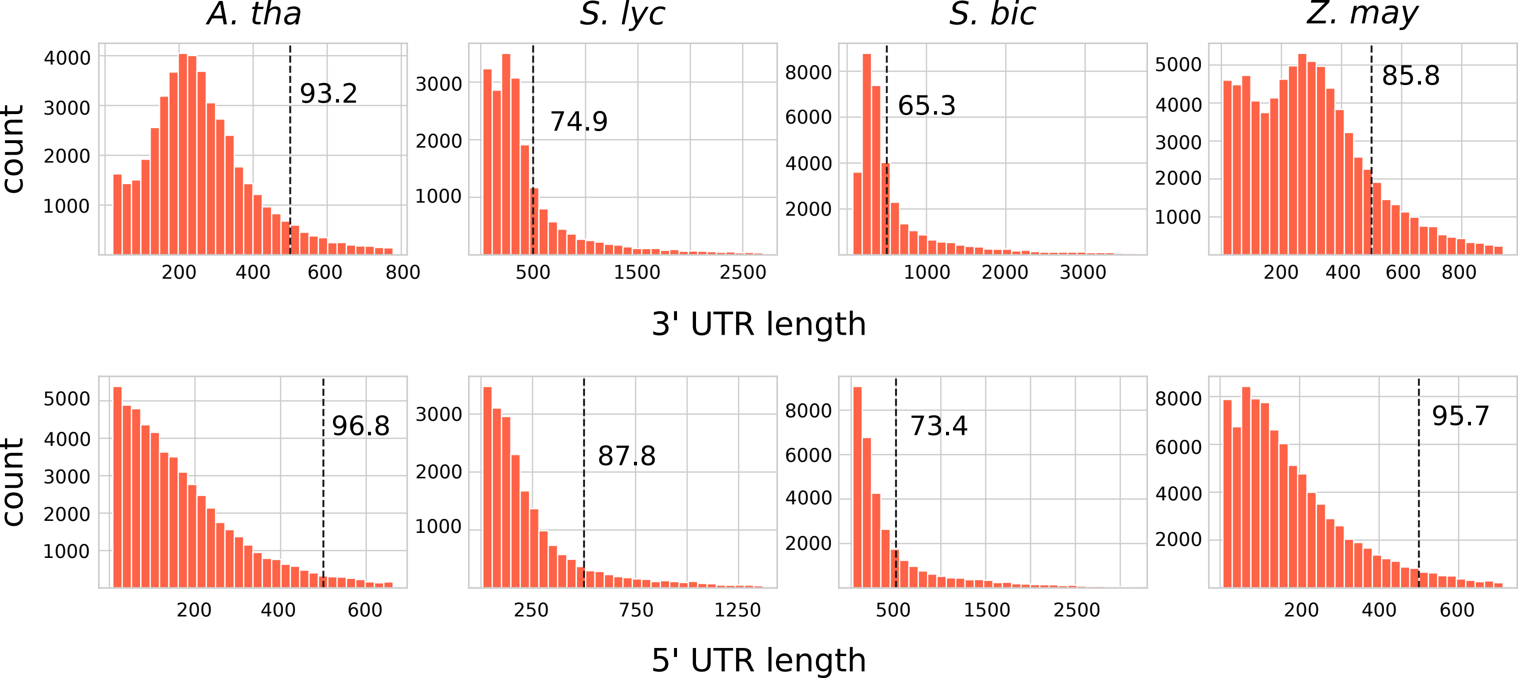
A. tha
S. lyc
S. bic
Z. may
count
93.2
74.9
65.3
85.8
3' UTR length
count
96.8
87.8
73.4
95.7
5' UTR length

Supplementary Figure 3 - The distribution of the UTR lengths of three prime and five prime ends for the four reference species *A. thaliana* (A. tha.), *S. lycopersicum* (S. lyc.), *S. bicolor* (S. bic.) and *Z. mays* (Z. may). Histograms show the counts of genes with distinct UTR lengths (red bars). There were 65.3 to 96.8 % of *S. bicolor* three prime UTR to *A. thaliana* five prime UTR shorter than 500 bp, indicated by the dashed line.

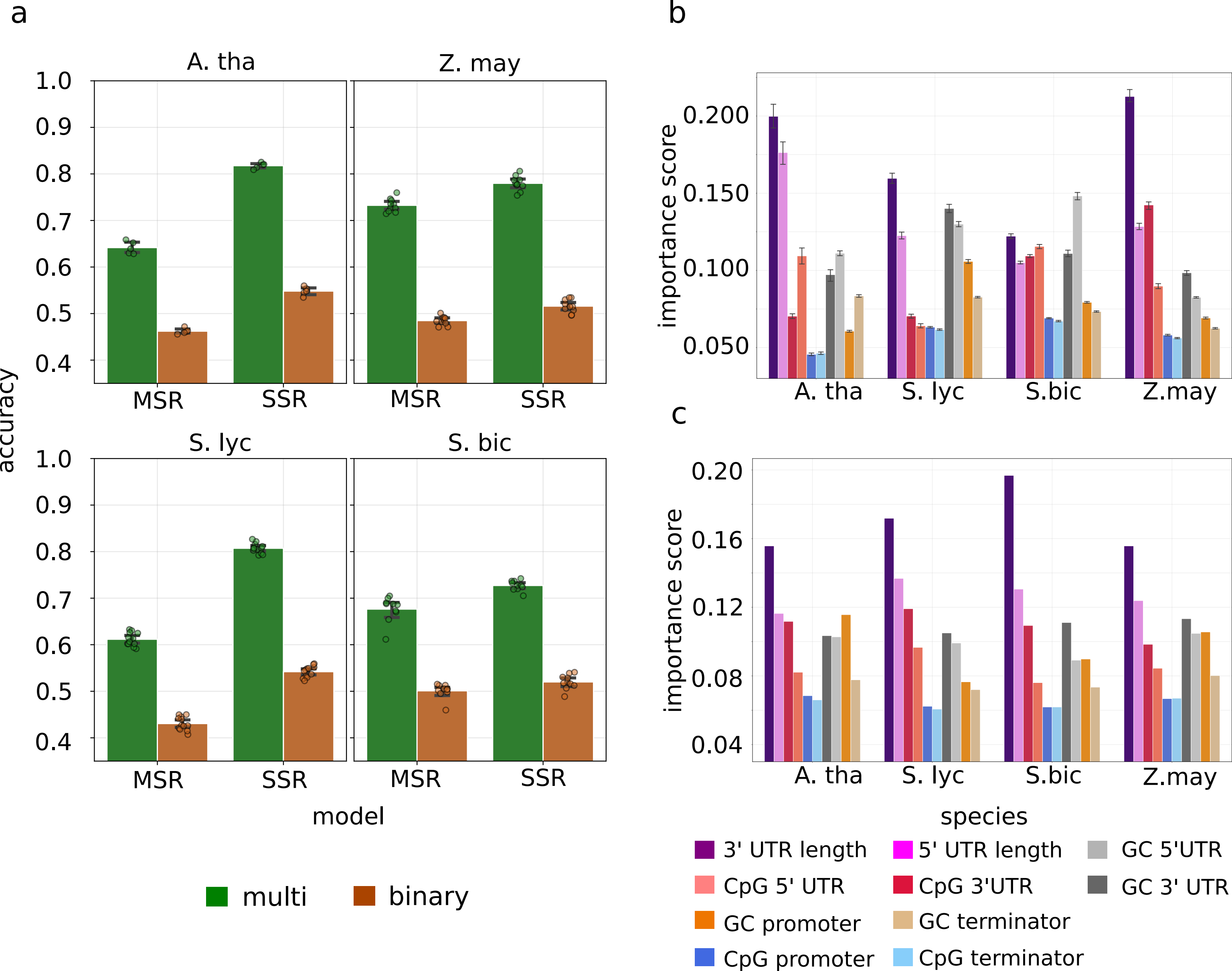

a
A. tha
Z. may
S. lyc
S. bic
1.0
0.9
0.8
0.7
0.6
0.5
0.4
accuracy
MSR
SSR
model
multi
binary
b
importance score
0.200
0.150
0.100
0.050
A. tha
S. lyc
S.bic
Z.may
c
importance score
0.20
0.16
0.12
0.08
0.04
species
3' UTR length
5' UTR length
GC 5'UTR
CpG 5' UTR
CpG 3'UTR
GC 3' UTR
GC promoter
GC terminator
CpG promoter
CpG terminator

Supplementary Figure 4 - Random forest models for the four reference plants trained on generic features generated based on the reference gene models. The models achieved high accuracies on test performance for SSR models but not the MSR setup, but outperformed prediction of multiple transcript level classes.

(a) Accuracy of random forest classifiers for binary (low-high) and multi (low-medium-high) classification models, are shown for the reference species *A. thaliana, Z. mays, S. lycopersicum,* and *S. bicolor*. Individual data points are depicted as circles (n = 5, 12, 10 and 10 for *A. thaliana, S. lycopersicum, S. bicolor,* and *Z. mays*). The error bars show the 95% confidence intervals and levels.

(b) Impurity-based feature importance from SSR random forest classifiers. random forest classifiers trained (c) Impurity-based feature importance from MSR random forest classifiers. The error bars show the 95% confidence intervals and levels.

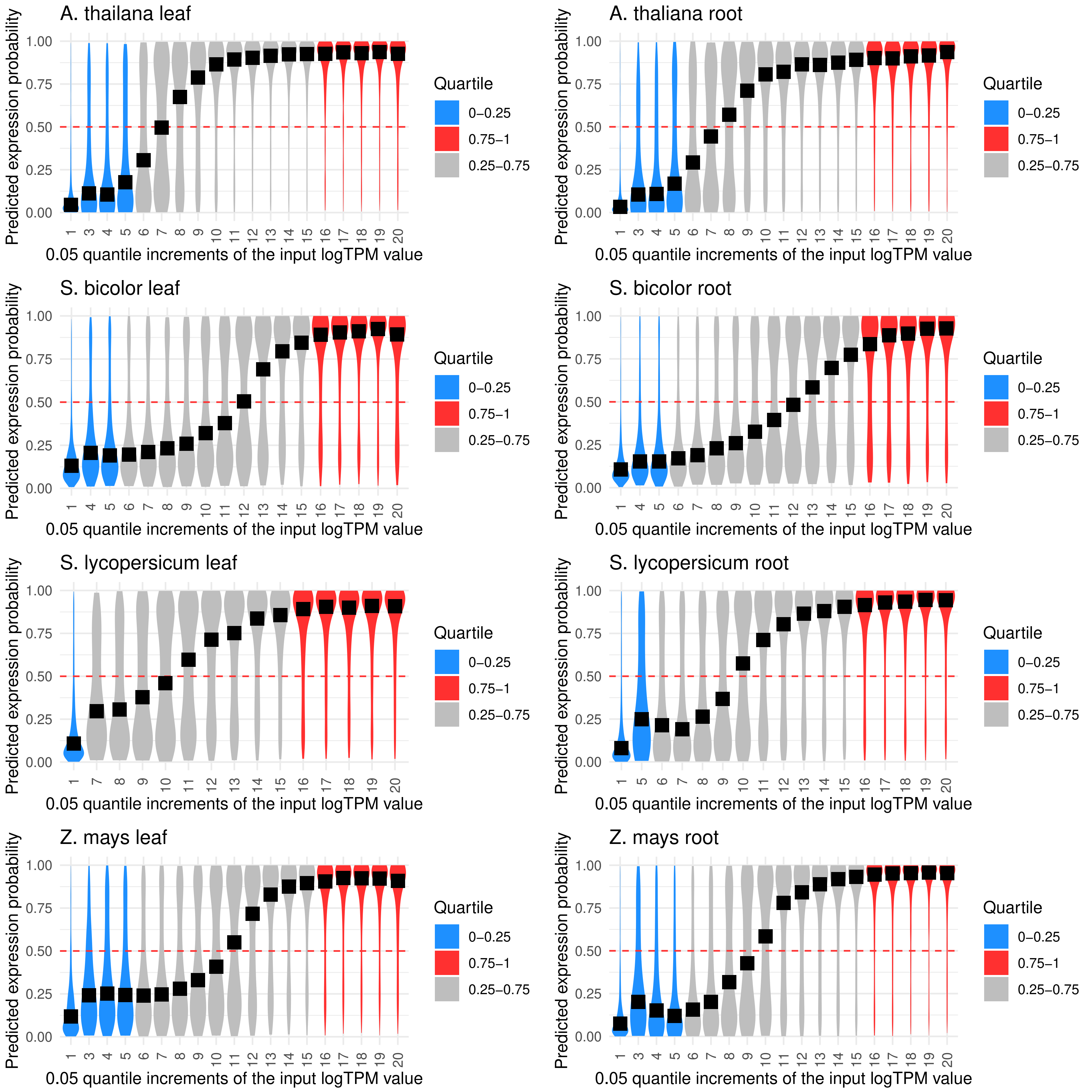
A. thailana leaf
A. thaliana root
S. bicolor leaf
S. bicolor root
S. lycopersicum leaf
S. lycopersicum root
Z. mays leaf
Z. mays root
Predicted expression probability
0.05 quantile increments of the input logTPM value
0.00
0.25
0.50
0.75
1.00
Quartile
0–0.25
0.75–1
0.25–0.75

Supplementary Figure 5 - Distribution of prediction probabilities for low and high levels of gene expression of SSR models. Violin plots display the predicted probabilities with mean (black square) for each consecutive 5% quantile of the log10maxTPM+1 values for each of the four species *A. thaliana, S. bicolor, S. lycopersicum* and *Z. mays* leaf and root samples, respectively.

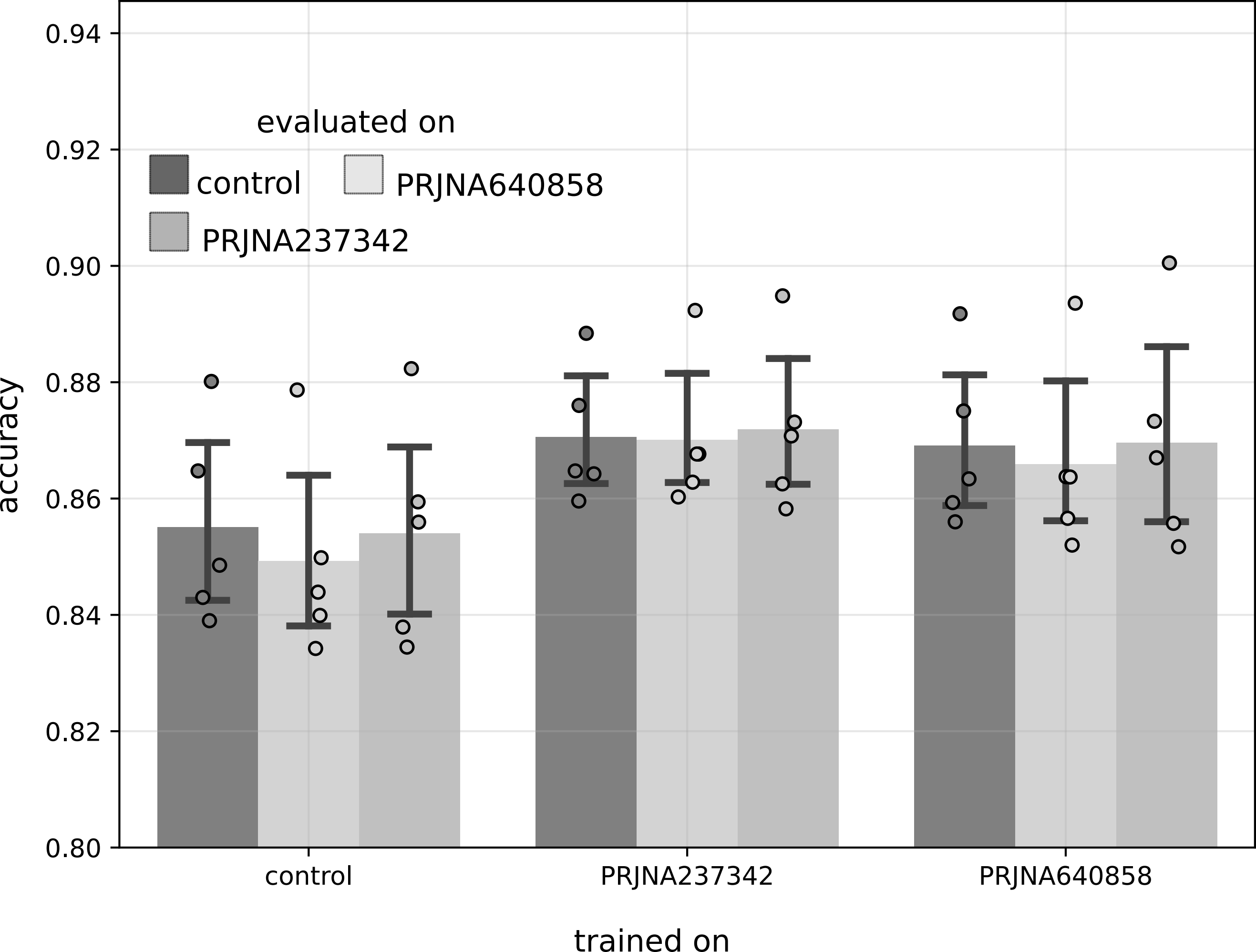
evaluated on
control
PRJNA640858
PRJNA237342
0.94
0.92
0.90
0.88
0.86
0.84
0.82
0.80
accuracy
control
PRJNA237342
PRJNA640858
trained on

Supplementary Figure 6 - Cross prediction performance for SSR models trained by different experimental data. SSR models for *A. thaliana* were generated from RNAseq experiment profiles PRJEB32665, PRJNA237342 and PRJNA640858 and individual predictive performance was cross evaluated to estimate the training strategy sensitivity to the effects of technical or experimental variation. Individual data points (n =5) are depicted as circles. The error bars show the 95% confidence intervals.

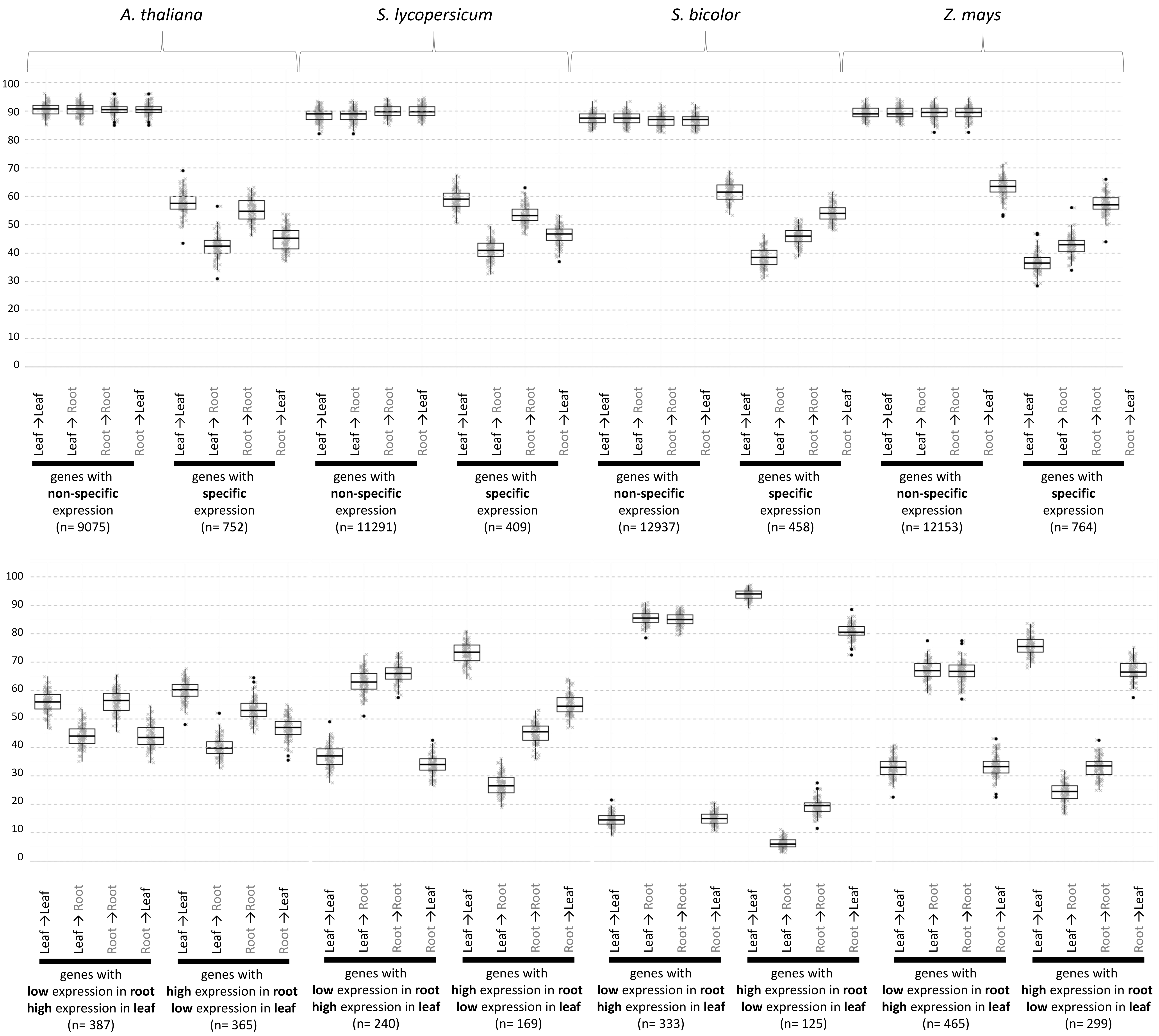
A. thaliana
S. lycopersicum
S. bicolor
Z. mays
100
90
80
70
60
50
40
30
20
10
0
Leaf →Leaf
Leaf → Root
Root →Root
Root →Leaf
genes with non-specific expression (n= 9075)
genes with specific expression (n= 752)
genes with non-specific expression (n= 11291)
genes with specific expression (n= 409)
genes with non-specific expression (n= 12937)
genes with specific expression (n= 458)
genes with non-specific expression (n= 12153)
genes with specific expression (n= 764)
genes with low expression in root high expression in leaf (n= 387)
genes with high expression in root low expression in leaf (n= 365)
genes with low expression in root high expression in leaf (n= 240)
genes with high expression in root low expression in leaf (n= 169)
genes with low expression in root high expression in leaf (n= 333)
genes with high expression in root low expression in leaf (n= 125)
genes with low expression in root high expression in leaf (n= 465)
genes with high expression in root low expression in leaf (n= 299)

Supplementary Figure 7 - Cross-validation of deep learning SSR models of leaf and root tissue was conducted for four plant species: *A. thaliana, S. lycopersicum, S. bicolor,* and *Z. mays*. To assess the predictive accuracy of these models for gene expression levels, we partitioned the test sets into genes with tissue-specific and non-specific expression patterns. Tissue specificity was determined based on gene expression classification; genes exhibiting opposite expression patterns in root and leaf tissues were considered tissue specific. We performed 1,000 bootstrap iterations to calculate average accuracies, accounting for varying sample sizes. Additionally, we further subdivided genes with tissue-specific expression to investigate tissue and expression class-specific effects on model performance, despite very small sample sizes. The boxplots depict summary statistics of individual average accuracies (gray crosses) per test condition randomly sampled by bootstrapping using the 25th, 50th (median), and 75th percentiles along with the interquartile range, representing the central 50% of the data. Whiskers extend from the minimum to maximum values, showcasing the spread of the dataset and outliers are depicted as ovals.

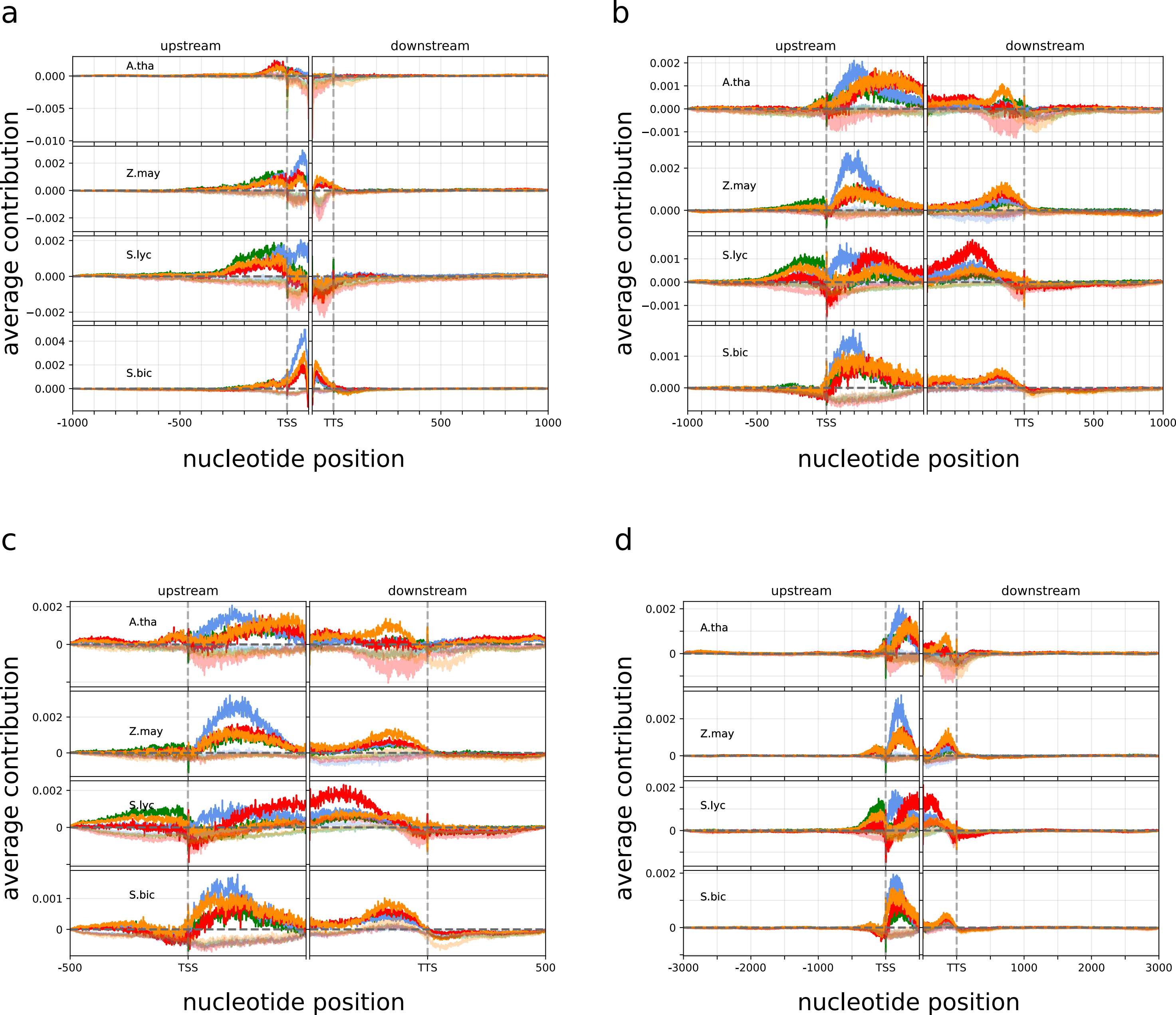

a
b
c
d
upstream
downstream
A.tha
Z.may
S.lyc
S.bic
average contribution
nucleotide position
TSS
TTS
-1000
-500
500
1000
-3000
-2000
-1000
2000
3000

Supplementary Figure 8 - Saliency maps for model training on different input sequence lengths. The model are interpreted by the calculation of DeepLift importance score for models trained with shorter UTRs of 100 nt (a), longer UTRs of 700 nt (b), shorter promoter-terminators of 500 nt (c) and longer promoter-terminators of 3000 nt (d).

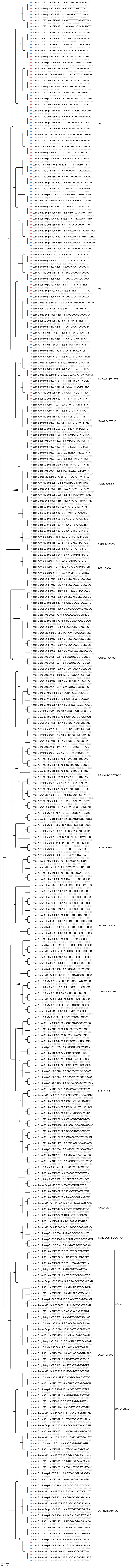

5W*
5W*
AAYNAA/ TTNRTT
MWCAG/ CTGWK
YACA/ TGTR 2
RARAR/ YTYTY
2CT+/ 2AG+
GSRGV/ BCYSC
RGAGAR/ YTCYTCY
4CNN/ 4NNG
2GCB+/ 2VGC+
CDGSV/ BSCHG
3SSN/ NSS3
KYKS/ SKRK
TNNGCCS/ SGGCNNA
CATG
2CWY/ 2RWG
CATC/ GTAG
CGNCGT/ ACNCG

Supplementary Figure 9 - Dendrogram of 520 EPMs of *A. thaliana, S. bicolor, S. lycopersicum* and *Z. mays* from SSR and MSR leaf models. EPMs were clustered by the Smith-Waterman algorithm. The resulting clades of EPMs with similar consensus sequences were named after alignment (**Supplementary Data 5**). EPMs associated with low (blue leaf-tips) and high rates (pink leaf tips) of gene expression find similar motifs across different models from SSR leaf (S0) and MSR leaf (M0). EPMs in reverse orientation are displayed gray.

a

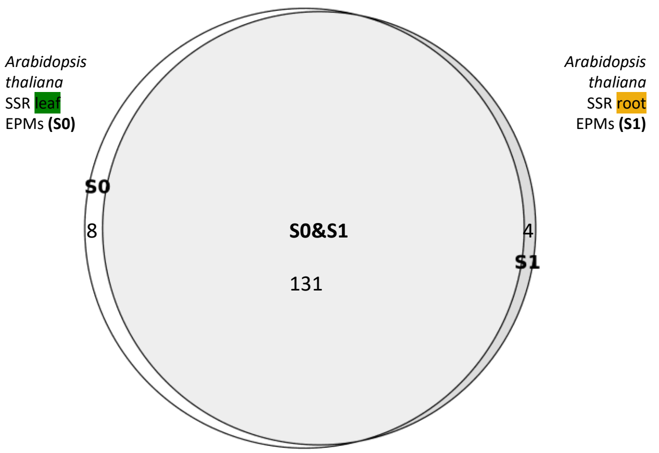

Unique root (S1) EPM	Common EPM			Unique leaf (S0) EPM
epm_Arth_S1_p0m24	epm_Arth_S1_p0m00	epm_Arth_S1_p0m22	epm_Arth_S0_p0m03	epm_Arth_S0_p0m05
epm_Arth_S1_p1m13	epm_Arth_S1_p0m01	epm_Arth_S1_p0m23	epm_Arth_S0_p0m04	epm_Arth_S0_p1m03
Root F=2, R=2; **4**	epm_Arth_S1_p0m02	epm_Arth_S1_p0m25	epm_Arth_S0_p0m06	epm_Arth_S0_p1m05
	epm_Arth_S1_p0m03	epm_Arth_S1_p1m00	epm_Arth_S0_p0m07	epm_Arth_S0_p1m14
	epm_Arth_S1_p0m04	epm_Arth_S1_p1m01	epm_Arth_S0_p0m08	Leaf F= 4, R= 4; **8**
	epm_Arth_S1_p0m05	epm_Arth_S1_p1m02	epm_Arth_S0_p0m09	
	epm_Arth_S1_p0m06	epm_Arth_S1_p1m03	epm_Arth_S0_p0m10	
	epm_Arth_S1_p0m07	epm_Arth_S1_p1m04	epm_Arth_S0_p0m11	
	epm_Arth_S1_p0m08	epm_Arth_S1_p1m05	epm_Arth_S0_p0m12	
	epm_Arth_S1_p0m09	epm_Arth_S1_p1m06	epm_Arth_S0_p0m13	
	epm_Arth_S1_p0m10	epm_Arth_S1_p1m07	epm_Arth_S0_p1m00	
	epm_Arth_S1_p0m11	epm_Arth_S1_p1m08	epm_Arth_S0_p1m01	
	epm_Arth_S1_p0m12	epm_Arth_S1_p1m09	epm_Arth_S0_p1m02	
	epm_Arth_S1_p0m13	epm_Arth_S1_p1m10	epm_Arth_S0_p1m04	
	epm_Arth_S1_p0m14	epm_Arth_S1_p1m11	epm_Arth_S0_p1m06	
	epm_Arth_S1_p0m15	epm_Arth_S1_p1m12	epm_Arth_S0_p1m07	
	epm_Arth_S1_p0m16	epm_Arth_S1_p1m14	epm_Arth_S0_p1m08	
	epm_Arth_S1_p0m17	epm_Arth_S1_p1m15	epm_Arth_S0_p1m09	
	epm_Arth_S1_p0m18	epm_Arth_S1_p1m16	epm_Arth_S0_p1m10	
	epm_Arth_S1_p0m19	epm_Arth_S0_p0m00	epm_Arth_S0_p1m11	
	epm_Arth_S1_p0m20	epm_Arth_S0_p0m01	epm_Arth_S0_p1m12	
	epm_Arth_S1_p0m21	epm_Arth_S0_p0m02	epm_Arth_S0_p1m13	
	Root F=41, R=41; **81**		Leaf F=25, R=25; **50**	

b

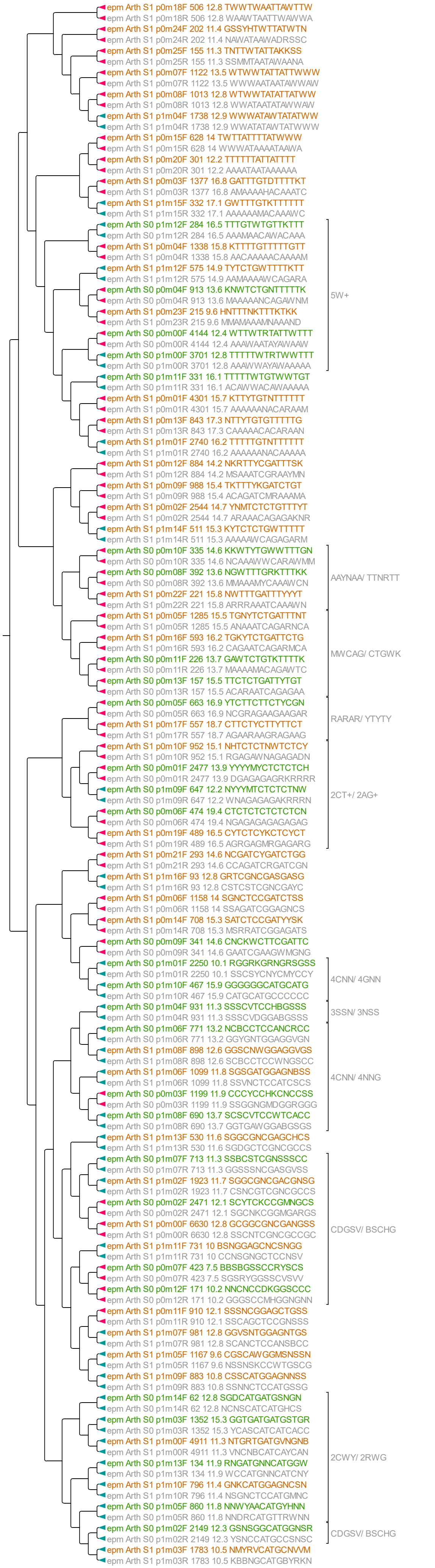

c

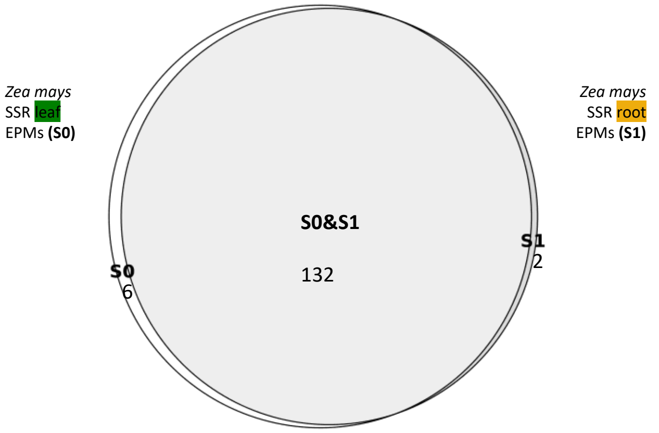

Unique root (S1) EPM	Common EPMs			Unique leaf (S0) EPM
epm_Zema_S1_p0m11	epm_Zema_S1_p0m00	epm_Zema_S1_p1m12	epm_Zema_S0_p0m15	epm_Zema_S0_p0m03
epm_Zema_S1_p1m01	epm_Zema_S1_p0m01	epm_Zema_S1_p1m13	epm_Zema_S0_p0m16	Root F=1, R=1; **2**
epm_Zema_S1_p1m04	epm_Zema_S1_p0m02	epm_Zema_S1_p1m14	epm_Zema_S0_p0m17	
Root F=3, R=3; **6**	epm_Zema_S1_p0m03	epm_Zema_S1_p1m15	epm_Zema_S0_p1m00	
	epm_Zema_S1_p0m04	epm_Zema_S1_p1m16	epm_Zema_S0_p1m01	
	epm_Zema_S1_p0m05	epm_Zema_S1_p1m17	epm_Zema_S0_p1m02	
	epm_Zema_S1_p0m06	epm_Zema_S1_p1m18	epm_Zema_S0_p1m04	
	epm_Zema_S1_p0m07	epm_Zema_S0_p0m00	epm_Zema_S0_p1m05	
	epm_Zema_S1_p0m08	epm_Zema_S0_p0m01	epm_Zema_S0_p1m06	
	epm_Zema_S1_p0m09	epm_Zema_S0_p0m02	epm_Zema_S0_p1m07	
	epm_Zema_S1_p0m10	epm_Zema_S0_p0m03	epm_Zema_S0_p1m08	
	epm_Zema_S1_p0m12	epm_Zema_S0_p0m04	epm_Zema_S0_p1m09	
	epm_Zema_S1_p1m00	epm_Zema_S0_p0m05	epm_Zema_S0_p1m10	
	epm_Zema_S1_p1m02	epm_Zema_S0_p0m06	epm_Zema_S0_p1m11	
	epm_Zema_S1_p1m03	epm_Zema_S0_p0m07	epm_Zema_S0_p1m12	
	epm_Zema_S1_p1m05	epm_Zema_S0_p0m08	epm_Zema_S0_p1m13	
	epm_Zema_S1_p1m06	epm_Zema_S0_p0m09	epm_Zema_S0_p1m14	
	epm_Zema_S1_p1m07	epm_Zema_S0_p0m10	epm_Zema_S0_p1m15	
	epm_Zema_S1_p1m08	epm_Zema_S0_p0m11	epm_Zema_S0_p1m16	
	epm_Zema_S1_p1m09	epm_Zema_S0_p0m12	epm_Zema_S0_p1m17	
	epm_Zema_S1_p1m10	epm_Zema_S0_p0m13	epm_Zema_S0_p1m18	
	epm_Zema_S1_p1m11	epm_Zema_S0_p0m14	epm_Zema_S0_p1m19	
	Root F=29, R=29; **58**		Leaf F= 37, R=37: **74**	

Supplementary Figure 10 – The comparison of EPMs from *A. thaliana* and *Z. mays* SSR leaf and root models. EPMs of *A. thaliana* (a) and *Z. mays* (b) SSR leaf (green) and root (brown) models exhibit a high degree of similarity. With over 80% of motifs having significant similarity towards one or more EPMs comparing both leaf and root EPMs with over 95% (p-value > 0.05). This similarity is determined through Pearson Correlation Coefficient.

(c) Smith-waterman-clustered dendrogram for *A. thaliana* SSR leaf and root model EPMs with similar clades found in the comparison of SSR and MSR leaf model EPMs (**Supplementary Figure 9**).

Supplementary Note 1 - Identification and characterization of predictive gene model features. While the interpretation of the models revealed a conserved pattern of associated saliency scores and the presence of specific EPMs, the contribution of general features of gene models is difficult to infer from the CNN model. Therefore, we generated ten generic features per gene and trained random forest (RF) classifiers for the SSR and MSR training strategy that were chosen arbitrarily by contribution scoring. For the 5' and 3' regions five generic features each were chosen: UTR length, CpG UTR content, CpG promoter region content, GC UTR content and GC promoter region content. Remarkably, our RF-SSR classifiers achieved accuracy values of 81.76%, 80.72%, 72.72%, and 77.98% in *A. thaliana*, *S. lycopersicum*, *S.bicolor*, and *Zea mays*, respectively (**Supplementary Figure 4**). In contrast, a lower performance for the RF-MSR models with accuracy values of 64.16%, 61.17%, 67.65% and 73.25% was observed for the species, respectively (**Supplementary Figure 4**). The impurity-based feature contributions calculated for the RF-SSR model indicated that the UTR-based features were the most important for classification with the lengths of the 3' UTRs being the most important feature across all species (**Supplementary Figure 4**). 5'UTR length showed higher importance for genes of *A. thaliana*, while 3'UTR GC content was highly predictive in genes of *S. lycopersicum*. The impurity-based contributions for the RF-MSR model also showed the importance of the UTR-based features, with the length of the 3'UTR being the most important feature (**Supplementary Figure 4**).
