# Supplementary material for: Deep learning the cis-regulatory code for gene expression in selected model plants
Source: Nat Commun. 2024 Apr 25;15:3488. doi: 10.1038/s41467-024-47744-0 (PMC11045779; doi:10.1038/s41467-024-47744-0)
Supplement: Supplementary file 3 — Description of Additional Supplementary Files [file 41467_2024_47744_MOESM3_ESM.pdf]

Description of Additional Supplementary Files:

Supplementary Data 1: Short read transcriptome profiles for leaf and root of A. thaliana, S. lycopersicum, S. bicolor, and Z. mays used for the training of CNN models predicting gene expression classes. Gene expression rates were determined using Kallisto (version 0.46.2). Gene expression rates were normalized using log10maxTPM+1 over multiple RNAseq experiments per organism (Source Data).

Supplementary Data 2: Evaluation of CNN-models predicting gene expression classes of A. thaliana, S. lycopersicum, S. bicolor, and Z. mays. The performance of convolutional neural networks (CNN) trained with leaf and root transcript profiles for plant species was estimated by accuracy (val_acc) and area under receiver operating characteristic curve (val_auROC) metrics. CNNs were trained using chromosomal level cross-validation, in which for each iteration genes located on one of the chromosomes were used as validation set and the rest for training. Training of models consisted of single-species references (SSR), multi-species references (MSR), species-specific references with homologous sequences (SSRU) and shuffled-sequence controls (SSC). Model performance was evaluated for the SSR and MSR models trained on expression profiles of leaf and root tissues independently. Performance was measured using accuracy (val_acc), area under receiver operating characteristic curve (val_auROC), F1 micro (val_f1_micro), F1 macro (val_f1_macro) and F1 weighted (val_f1_weighted) scores (Source Data).

Supplementary Data 3: Prediction accuracy, F1 scores and means of log10maxTPM expression for Mercator4 functional gene categories (provided in Supplementary Data 11). The table contains only functional categories with the number of annotated genes equal or larger than 100 in at least one of the four analyzed species.

Supplementary Data 4: Characterization of 260 EPMs of the SSR and MSR models from leaf data of A. thaliana, S. bicolor, S. lycopersicum and Z. mays. We obtained expression predictive motifs (EPMs) using DeepLift and TFModisco, which provides both cluster-weighted models and position weight matrices (PWMs). To further refine and cluster these EPMs, with the Smith-Waterman algorithm through the R package motifStack (Ou et al. 2018). Our nomenclature system for EPMs follows a structured format. It starts with abbreviations denoting the plant species based on their genus and epithet. This is followed by the model used for EPM generation SSR or MSR, abbreviated with “S” or “M”, respectively. The numerical value indicates the physiological conditions of the plant (0 for standard condition in leaf, 1 for standard condition in root). To convey the predictiveness of each motif, we use 'p' followed by a series of 1s and 0s, representing its association with low and high rates of gene expression, respectively. This is followed by a delimiter, motif number within the metacluster, and its orientation, abbreviated as “F” or “R” for forward or reverse, respectively. The EPM information is concluded with details such as the number of seqlets included, Motifstack-derived sequence information content, and consensus sequences derived from importance scores converted to a PWM. Clusters of EPMs are named based on their consensus sequence using the IUPAC nucleotide code, with the fewest repetitions as a prefix after their alignments (see

Supplementary Data 5). Additionally, we compared these EPMs to well-characterized transcription factor binding sites and associated factors available in the JASPAR 2022 (Castro-Mondragon et al. 2022) plants database. Notably, we listed significant top matches for each EPM with p-values below 0.05, enhancing their relevance and context. The predictive performance of each EPM is assessed through importance scores (IS) generated with DeepLift. Positive IS values are indicative of high gene expression predictions, while negative IS values suggest low gene expression predictions. We provide a table summarizing the sum of 14-nt long EPMs along with their maximum and minimum IS values. To define positional preferences in upstream and downstream regions, we extracted this information from the HDF5 files of CNN models generated using DeepLift/TFMoDisco (Avsec et al. 2021; Shrikumar, Greenside, and Kundaje 06--11 Aug 2017). The reported range reflects the occurrence of seqlets across the training set used for EPM generation, providing valuable insights into their spatial distribution.

Supplementary Data 5: Alignment of consensus sequences of 260 EPMs from leaf SSR and MSR models following Smith-Waterman clustering. Common consensus sequences within the 19 different clusters were used for naming the clusters. Alignment and Consensus view using JalView (Procter et al. 2021) was used for visualization and assistance for the nomenclature.

Supplementary Data 6: Inference of predictive performance of EPMs from A. thaliana SSR and MSR leaf models. The EPMs occurrences across the A. thaliana genes classified and predicted to be lowly and highly expressed were checked for enrichment. Initially, EPMs were mapped to genes 1 kbp up- and downstream extension of their reference genome, employing a high sensitivity threshold of 1-e3 through the utilization of BLAMM (Fostier 2020). EPMs of precisely 14 nucleotides in length, with a matching score above 10 were retained. Subsequently, these motifs were filtered based on their positional preferences, ensuring their placement within the inner 80% quantile, as dictated by the distribution of seqlets generated by DeepLift/TFMoDisco. In addition, EPMs were deemed relevant if present in these areas in at least 20% of cases, according to the distribution of related seqlets, too. Those occurrences that did not meet this criterion were excluded from further consideration. In addition, occurrences of each EPM within annotated gene features (transcribed or non-transcribed/IGS) were counted. As a variation of a method initially proposed by Smet and colleagues (Smet, Opdebeeck, and Vandepoele 2023), we calculated feature enrichment of EPMs per associated class. Here, a positive enrichment is indicative of an EPM being present in its respective set of lowly or highly expressed genes. The proportional occurrences in percentages of EPMs were calculated within their respective gene expression classes and the predicted one. In addition, we calculated rates for their occurrence among true positive and negative samples in the prediction.

Supplementary Data 7: Predictions of gene expression for Solanum genotypes with structural variation using the S. lycopersicum MSR leaf model. Predicted probabilities are calculated from the varying results of the chromosome-specific test-set outcomes of the model ranging from 0 to 1. If predicted probabilities are <= 0.5, predicted gene expression levels are "low" and vice versa. For downstream analyses in distinction between predicted homogenous and differential gene expression, variances between the predicted probabilities were calculated. Here, variances > 0.005 of the predicted probabilities for gene expression classes, were used as threshold for classification (Source Data).

Supplementary Data 8: Determination of EPMs conservation state from S. lycopersicum MSR leaf model in fifteen Solanum genotypes. Using BLAMM genes with predicted homogenous and differential gene expression levels shown in supplementary data 7 were screened for variance in occurrence of S. lycopersicum MSR leaf EPMs among orthologous genes. If an EPM occurred at the same position among orthologous genes, it is considered “conserved”, else, it is considered “mutated” (Source Data).

Supplementary Data 9: Alignment of exemplary candidate genes from fifteen Solanum genotypes with predicted differential gene expression levels and mutated EPMs. The selected genes were aligned using MAFFT and alignments visualized using GeneDoc (Nicholas and Nicholas 1997).

Supplementary Data 10: MapMan categories of differentially expressed genes in S. pennellii leaf in respect to S. lycopersicum MSR leaf models. Functional annotation of S. lycopersicum and S. pennellii genes to MapMan functional categories was performed using Mercator4 (Lohse et al. 2014) on ITAG 4.1 and the latest S. pennellii genome Schmidt and colleagues (2017) (Schmidt et al. 2017), as described in Mercator4. The p-values of a two-sided Wilcoxon rank-sum test are provided with the respective FDR calculated using Benjamini-Hochberg correction.

Significance of the shift of median between S. lycopersicum and S. pennellii shown in Figure 6c was performed by a two-sided Wilcoxon rank sum test with BenjaminiHochberg p-value correction.

Supplementary Data 11: Mercator4 annotations for A. thaliana, S.lycopersicum, Sorghum bicolor, and Zea mays genomes based on peptide fasta files from Ensembl Plant database (v52)
