# Supplementary material for: Deep learning the cis-regulatory code for gene expression in selected model plants
Source: Nat Commun. 2024 Apr 25;15:3488. doi: 10.1038/s41467-024-47744-0 (PMC11045779; doi:10.1038/s41467-024-47744-0)
Supplement: Supplementary file 12 — Supplementary Data 9 [file 41467_2024_47744_MOESM12_ESM.pdf]

Supplementary data 9 – **Alignments of fifteen *Solanum* genotypes with differently predicted gene expression rates, structural variations (SVs) and mutated expression predictive motifs (EPM).** We used the MSR models trained with *Solanum lycopersicum* as the validation set to generate predictions for a subset of diverse varieties and their genotypes of *Solanum pimpinellifolium* (PAS014479, BGV006775), *S. lycopersicum* var. cerasiforme (BGV006865, BGV007931, BGV007989), *S. lycopersium* processing (M82), *S. lycopersicum* fresh (EA00371, Fla.8924, LYC1410),*S. lycopersicum* vintage (PI69588, Brandywine, EA00990, PI303721) and *S. lycorpersicum* ITAG 3.0 for reference. Highly and lowly predicted genotypes are coloured in red and blue in their first line of appearance per gene, respectively. As structural variations all InDel mutations larger than 10 are considered. All genes shown here were the object of SVs coinciding with variance in gene expression levels observed by Alonge et al., 2020. Gene sequences were aligned using MAFFT (Katoh et al., 2019) and manual adjustment in MEGA7.0 (Kumar et al., 1993). Conserved nucleotide region was shaded from light grey (>80%), to grey (90%) to black (100%) in GeneDoc V2.7 (Nicholas et al., 2006). The seven genes, shown here (Solyc01g111020.3, Solyc02g087170.4, Solyc11g012385.1, Solyc02g080300.3, Solyc09g064610.3, and Solyc04g051600.4).,were selected arbitrarily from a pool of 100 genes with highest variance (>0.02) of the predicted expression levels and independently of the relationship between the genotypes. The transcription start sites (TSS) and transcription termination sites (TTS) of genes lies at position 1000 nt and 2020 nt, respectively. All sequences contain the 20 nt padding sequence separating the genes flanking regions shaded brown. Mutated EPM regions that coincide with changes in expression class are underlined.

Solyc01g111020

```
                      *        20         *        40         *        60         *        80         *       100         *       120         *
ITAG.3     : ----------------------------------------------------------------------------------------------------------------------------------- :    -
BGV006865. : ----------------------------------------------------------------------------------------------------------------------------------- :    -
BGV007931. : ----------------------------------------------------------------------------------------------------------------------------------- :    -
BGV007989. : ----------------------------------------------------------------------------------------------------------------------------------- :    -
M82.3      : TAAGCTATACTCTGTGAATAACATAAACGCTTCACCTTTCTTTTCATGAAATAGACGCCGATTTCTTGATTCTTGAAAATTTTGTATTGTAAAATCAAGAATGTAGTTGGTTCAGTACATGCTATTTGATT :  131
EA00371.3  : TAAGCTATACTCTGTGAATAACATAAACGCTTCACCTTTCTTTTCATGAAATAGACGCCGATTTCTTGATTCTTGAAAATTTTGTATTGTAAAATCAAGAATGTAGTTGGTTCAGTACATGCTATTTGATT :  131
Fla.8924.3 : ----------------------------------------------------------------------------------------------------------------------------------- :    -
Floradade. : ----------------------------------------------------------------------------------------------------------------------------------- :    -
LYC1410.3  : ----------------------------------------------------------------------------------------------------------------------------------- :    -
PI169588.3 : ----------------------------------------------------------------------------------------------------------------------------------- :    -
Brandywine : TAAGCTATACTCTGTGAATAACATAAACGCTTCACCTTTCTTTTCATGAAATAGACGCCGATTTCTTGATTCTTGAAAATTTTGTATTGTAAAATCAAGAATGTAGTTGGTTCAGTACATGCTATTTGATT :  131
EA00990.3  : ----------------------------------------------------------------------------------------------------------------------------------- :    -
PI1303721. : ----------------------------------------------------------------------------------------------------------------------------------- :    -
BGV006775. : ----------------------------------------------------------------------------------------------------------------------------------- :    -
PAS014479. : ----------------------------------------------------------------------------------------------------------------------------------- :    -

                 140         *       160         *       180         *       200         *       220         *       240         *       260
ITAG.3     : ---------------------------------------------------------------------------------------------TTCTTATGCTTACTTTATAAGAAAGTTGCTCCATAATA :   38
BGV006865. : ---------------------------------------------------------------------------------------------TTCTTATGCTTACTTTATAAGAAAGTTGCTCCATAATA :   38
BGV007931. : ---------------------------------------------------------------------------------------------TTCTTATGCTTACTTTATAAGAAAGTTGCTCCATAATA :   38
BGV007989. : ---------------------------------------------------------------------------------------------TTCTTATGCTTACTTTATAAGAAAGTTGCTCCATAATA :   38
M82.3      : GCAATAAGGCTAACTCTTCATTTGTTAATAGTGATTTGGAGTTGTTTATATGGAAGGGAAATAATATACTAATTATTAATATCTCAGAACTAT-------------------------------------- :  224
EA00371.3  : GCAATAAGGCTAACTCTTCATTTGTTAATAGTGATTTGGAGTTGTTTATATGGAAGGGAAATAATATACTAATTATTAATATCTCAGAACTAT-------------------------------------- :  224
Fla.8924.3 : ---------------------------------------------------------------------------------------------TTCTTATGCTTACTTTATAAGAAAGTTGCTCCATAATA :   38
Floradade. : ---------------------------------------------------------------------------------------------TTCTTATGCTTACTTTATAAGAAAGTTGCTCCATAATA :   38
LYC1410.3  : ---------------------------------------------------------------------------------------------TTCTTATGCTTACTTTATAAGAAAGTTGCTCCATAATA :   38
PI169588.3 : ---------------------------------------------------------------------------------------------TTCTTATGCTTACTTTATAAGAAAGTTGCTCCATAATA :   38
Brandywine : GCAATAAGGCTAACTCTTCATTTGTTAATAGTGATTTGGAGTTGTTTATATGGAAGGGAAATAATATACTAATTATTAATATCTCAGAACTAT-------------------------------------- :  224
EA00990.3  : ----------------------------------------------------------------------------------------------TCTTATGCTTACTTTATAAGAAAGTTGCTCCATAATA :   37
PI1303721. : ---------------------------------------------------------------------------------------------TTCTTATGCTTACTTTATAAGAAAGTTGCTCCATAATA :   38
BGV006775. : ---------------------------------------------------------------------------------------------TTCTTATGCTTACTTTATAAGAAAGTTGCTCCATAATA :   38
PAS014479. : ---------------------------------------------------------------------------------------------TTCTTATGCTTACTTTATAAGAAAGTTGCTCCATAATA :   38
                                                                                                          tcttatgcttactttataagaaagttgctccataata

                    *       280         *       300         *       320         *       340         *       360         *       380         *
ITAG.3     : ATTACAATAAAGTTTTTGGCACAATAATATTACCTTAGCTCTTGAAATATTTATTTTACTTATGTGGTAGCACCCAAGATAATAAATAGACTCTCTAAATAATAACATTAGTATGTGAAATAATATTGAGA :  169
BGV006865. : ATTACAATAAAGTTTTTGGCACAATAATATTACCTTAGCTCTTGAAATATTTATTTTACTTATGTGGTAGCACCCAAGATAATAAATAGACTCTCTAAATAATAACATTAGTATGTGAAATAATATTGAGA :  169
BGV007931. : ATTACAATAAAGTTTTTGGCACAATAATATTACCTTAGCTCTTGAAATATTTATTTTACTCATGTGGTAGCACCCAAGATAATAAATAGACTCTCTAAATAATAACATTAGTATGTGAAATAATATTGAGA :  169
BGV007989. : ATTACAATAAAGTTTTTGGCACAATAATATTACCTTAGCTCTTGAAATATTTATTTTACTTATGTGGTAGCACCCAAGATAATAAATAGACTCTCTAAATAATAACATTAGTATGTGAAATAATATTGAGA :  169
M82.3      : ----------------------------------------------------------------------------------------------------------------------------------- :    -
EA00371.3  : ----------------------------------------------------------------------------------------------------------------------------------- :    -
Fla.8924.3 : ATTACAATAAAGTTTTTGGCACAATAATATTACCTTAGCTCTTGAAATATTTATTTTACTTATGTGGTAGCACCCAAGATAATAAATAGACTCTCTAAATAATAACATTAGTATGTGAAATAATATTGAGA :  169
Floradade. : ATTACAATAAAGTTTTTGGCACAATAATATTACCTTAGCTCTTGAAATATTTATTTTACTTATGTGGTAGCACCCAAGATAATAAATAGACTCTCTAAATAATAACATTAGTATGTGAAATAATATTGAGA :  169
LYC1410.3  : ATTACAATAAAGTTTTTGGCACAATAATATTACCTTAGCTCTTGAAATATTTATTTTACTTATGTGGTAGCACCCAAGATAATAAATAGACTCTCTAAATAATAACATTAGTATGTGAAATAATATTGAGA :  169
PI169588.3 : ATTACAATAAAGTTTTTGGCACAATAATATTACCTTAGCTCTTGAAATATTTATTTTACTTATGTGGTAGCACCCAAGATAATAAATAGACTCTCTAAATAATAACATTAGTATGTGAAATAATATTGAGA :  169
Brandywine : ----------------------------------------------------------------------------------------------------------------------------------- :    -
EA00990.3  : ATTACAATAAAGTTTTAGGCACAATAATATTACCTTAGTTCTTGAAATATTTATTTTACTTATGTGGTAGCACCCAAGATAATAAATAGACTCTCTAAATAATAACATTAGTATGTGAAATAATATTGAGA :  168
PI1303721. : ATTACAATAAAGTTTTTGGCACAATAATATTACCTTAGCTCTTGAAATATTTATTTTACTTATGTGGTAGCACCCAAGATAATAAATAGACTCTCTAAATAATAACATTAGTATGTGAAATAATATTGAGA :  169
BGV006775. : ATTACAATAAAGTTTTTGGCACAATAATATTACCTTAGCTCTTGAAATATTTATTTTACTTATGTGGTAGCACCCAAGATAATAAATAGACTCTCTAAATAATAACATTAGTATGTGAAATAATATTGAGA :  169
PAS014479. : ATTACAATAAAGTTTTAGGCACAATAATATTACCTTAGTTCTTGAAATATTTATTTTACTTATGTGGTAGCACCCAAGATAATAAATAGACTCTCTAAATAATAACATTAGTATGTGAAATAATATTGAGA :  169
             attacaataaagtttt ggcacaataatattaccttag tcttgaaatatttattttact atgtggtagcacccaagataataaatagactctctaaataataacattagtatgtgaaataatattgaga

                400         *       420         *       440         *       460         *       480         *       500         *       520
ITAG.3     : TTATTCTTTTGATTATTTATTAAAAACTATTTTTAGACATGAATAATGTTGTTCTTTTTTTCATACATGTTCCAAGATATTAATTATGGTGCAAATCTTGTTATTTATTCTTACTCCTATTATACGTAGTA :  300
BGV006865. : TTATTCTTTTGATTATTTATTAAAAACTATTTTTAGACATGAATAATGTTGTTCTTTTTTTCATACATGTTCCAAGATATTAATTATGGTGCAAATCTTGTTATTTATTCTTACTCCTATTATACGTAGTA :  300
BGV007931. : TTATTCTTTTGATTATTTATTAAAAACTATTTTTAGACATGAATAATGTTGTTCTTTTTTTCATACATGTTCCAAGATATTAATTATGGTGCAAATCTTGTTATTTATTCTTACTCCTATTATACGTAGTA :  300
BGV007989. : TTATTCTTTTGATTATTTATTAAAAACTATTTTTAGACATGAATAATGTTGTTCTTTTTTTCATACATGTTCCAAGATATTAATTATGGTGCAAATCTTGTTATTTATTCTTACTCCTATTATACGTAGTA :  300
M82.3      : ----------------------------------------------------------------------------------------------------------------------------------- :    -
EA00371.3  : ----------------------------------------------------------------------------------------------------------------------------------- :    -
Fla.8924.3 : TTATTCTTTTGATTATTTATTAAAAACTATTTTTAGACATGAATAATGTTGTTCTTTTTTTCATACATGTTCCAAGATATTAATTATGGTGCAAATCTTGTTATTTATTCTTACTCCTATTATACGTAGTA :  300
Floradade. : TTATTCTTTTGATTATTTATTAAAAACTATTTTTAGACATGAATAATGTTGTTCTTTTTTTCATACATGTTCCAAGATATTAATTATGGTGCAAATCTTGTTATTTATTCTTACTCCTATTATACGTAGTA :  300
LYC1410.3  : TTATTCTTTTGATTATTTATTAAAAACTATTTTTAGACATGAATAATGTTGTTCTTTTTTTCATACATGTTCCAAGATATTAATTATGGTGCAAATCTTGTTATTTATTCTTACTCCTATTATACGTAGTA :  300
PI169588.3 : TTATTCTTTTGATTATTTATTAAAAACTATTTTTAGACATGAATAATGTTGTTCTTTTTTTCATACATGTTCCAAGATATTAATTATGGTGCAAATCTTGTTATTTATTCTTACTCCTATTATACGTAGTA :  300
Brandywine : ----------------------------------------------------------------------------------------------------------------------------------- :    -
EA00990.3  : TTATTCTTTTGATTATTTATTAAAAACTATTTTTAGACATGAATAATGTTGTTCTTTTTTTCATACATGTTCCAAGATATTAATTATGGTGCAAATCTTGTTATTTATTCTTACTCCTATTATACGTAGTA :  299
PI1303721. : TTATTCTTTTGATTATTTATTAAAAACTATTTTTAGACATGAATAATGTTGTTCTTTTTTTCATACATGTTCCAAGATATTAATTATGGTGCAAATCTTGTTATTTATTCTTACTCCTATTATACGTAGTA :  300
BGV006775. : TTATTCTTTTGATTATTTATTAAAAACTATTTTTAGACATGAATAATGTTGTTCTTTTTTTCATACATGTTCCAAGATATTAATTATGGTGCAAATCTTGTTATTTATTCTTACTCCTATTATACGTAGTA :  300
PAS014479. : TTATTCTTTTGATTATTTATTAAAAACTATTTTTAGACATGAATAATGTTGTTCTTTTTTTCATACATGTTCCAAGATATTAATTATGGTGCAAATCTTGTTATTTATTCTTACTCCTATTATACGTAGTA :  300
             ttattcttttgattatttattaaaaactatttttagacatgaataatgttgttctttttttcatacatgttccaagatattaattatggtgcaaatcttgttatttattcttactcctattatacgtagta

                  *       540         *       560         *       580         *       600         *       620         *       640         *
ITAG.3     : GAACTGCACGATCGTCAAAGATCGTGATAGTATAATACATATTTTCTTATTTTCAATTACTTATTTCACATAAAATATTTATACTTTTTAAAGTCGTATACTATTTGATCCGGCCTACTTTAATTTTTATC :  431
BGV006865. : GAATTGCACGATCGTCAAAGATCGTGATAGTATAATACATATTTTCTTATTTTCAATTACTTATTTCACATAAAATATTTATACTTTTTAAAGTTGTATATTATTTGATCCGGCCTACTTTAATTTTTATC :  431
BGV007931. : GAACTGCACGATCGTCAAAGATCGTGATAGTATAATACATATTTTCTTATTTTCAATTACTTATTTCACATAAAATATTTATACTTTTTAAAGTTGTATATTATTTGATCCGGCCTACTTTAATTTTTATC :  431
BGV007989. : GAACTGCACGATCGTCAAAGATCGTGATAGTATAATACATATTTTCTTATTTTCAATTACTTATTTCACATAAAATATTTATACTTTTTAAAGTCGTATACTATTTGATCCGGCCTACTTTAATTTTTATC :  431
M82.3      : ----------------------------------------------------------------------------------------------------------------------------------- :    -
EA00371.3  : ----------------------------------------------------------------------------------------------------------------------------------- :    -
Fla.8924.3 : GAACTGCACGATCGTCAAAGATCGTGATAGTATAATACATATTTTCTTATTTTCAATTACTTATTTCACATAAAATATTTATACTTTTTAAAGTCGTATACTATTTGATCCGGCCTACTTTAATTTTTATC :  431
Floradade. : GAACTGCACGATCGTCAAAGATCGTGATAGTATAATACATATTTTCTTATTTTCAATTACTTATTTCACATAAAATATTTATACTTTTTAAAGTCGTATACTATTTGATCCGGCCTACTTTAATTTTTATC :  431
LYC1410.3  : GAACTGCACGATCGTCAAAGATCGTGATAGTATAATACATATTTTCTTATTTTCAATTACTTATTTCACATAAAATATTTATACTTTTTAAAGTCGTATACTATTTGATCCGGCCTACTTTAATTTTTATC :  431
PI169588.3 : GAACTGCACGATCGTCAAAGATCGTGATAGTATAATACATATTTTCTTATTTTCAATTACTTATTTCACATAAAATATTTATACTTTTTAAAGTCGTATACTATTTGATCCGGCCTACTTTAATTTTTATC :  431
Brandywine : ----------------------------------------------------------------------------------------------------------------------------------- :    -
EA00990.3  : GAACTGCACAATCGTCAAAGATCGTGATAGTATAATACATATTTTCTTATTTTCAATTACTTATTTCACATAAAATATTTATACTTTTTAAAGTTGTATACTATTTGATCCGATCTACTTTAATTTTTATC :  430
PI1303721. : GAACTGCACGATCGTCAAAGATCGTGATAGTATAATACATATTTTCTTATTTTCAATTACTTATTTCACATAAAATATTTATACTTTTTAAAGTCGTATACTATTTGATCCGGCCTACTTTAATTTTTATC :  431
BGV006775. : GAACTGCACGATCGTCAAAGATCGTGATAGTATAATACATATTTTCTTATTTTCAATTACTTATTTCACATAAAATATTTATACTTTTTAAAGTTGTATACTATTTGATCCGGCCTACTTTAATTTTTATC :  431
PAS014479. : GAACTGCACAATCGTCAAAGATCGTGATAGTATAATACATATTTTCTTATTTTCAATTACTTATTTCACATAAAATATTTATACTTTTTAAAGTTGTATACTATTTGATCCGATCTACTTTAATTTTTATC :  431
             gaa tgcac atcgtcaaagatcgtgatagtataatacatattttcttattttcaattacttatttcacataaaatatttatactttttaaagt gtata tatttgatccg  ctactttaatttttatc
                                                                                          epm_Soly_M0_p0m11R_294 731
                                                                                                epm_Soly_M0_p0m11R_294   731

              660         *       680         *       700         *       720         *       740         *       760         *       780
ITAG.3     : AATTCGAATTTACTAAAAATATCCGATTTAAACTAATTATCGTTTTGAGAATTAAAACATAATTAATGATAAATTATAATTAATAATTTTTATTTGATAAAATATGAAGATATTAAAAAGTAATATTAAATA :  562
BGV006865. : AATTCGAATTTACTAAAAACATCCGATTTAAACTAATTATCGTTTTGAGAATTAAAACATAATTAATAATAAATTATAATTAATAATTTTTATTTAATAAAATATGAAGATATTAAAAAGTAATATTAAATA :  562
BGV007931. : AATTCGAATTTACTAAAAACATCCGATTTAAACTAATTATCGTTTTGAGAATTAAAACATAATTAATAATAAATTATAATTAATAATTTTTATTTAATAAAATATGAAGATATTAAAAAGTAATATTAAATA :  562
BGV007989. : AATTCGAATTTACTAAAAATATCCGATTTAAACTAATTATCGTTTTGAGAATTAAAACATAATTAATGATAAATTATAATTAATAATTTTTATTTGATAAAATATGAAGATATTAAAAAGTAATATTAAATA :  562
M82.3      : ----------------------------------------------------------------------------------------------------------------------------------- :    -
EA00371.3  : ----------------------------------------------------------------------------------------------------------------------------------- :    -
Fla.8924.3 : AATTCGAATTTACTAAAAATATCCGATTTAAACTAATTATCGTTTTGAGAATTAAAACATAATTAATGATAAATTATAATTAATAATTTTTATTTGATAAAATATGAAGATATTAAAAAGTAATATTAAATA :  562
Floradade. : AATTCGAATTTACTAAAAATATCCGATTTAAACTAATTATCGTTTTGAGAATTAAAACATAATTAATGATAAATTATAATTAATAATTTTTATTTGATAAAATATGAAGATATTAAAAAGTAATATTAAATA :  562
LYC1410.3  : AATTCGAATTTACTAAAAATATCCGATTTAAACTAATTATCGTTTTGAGAATTAAAACATAATTAATGATAAATTATAATTAATAATTTTTATTTGATAAAATATGAAGATATTAAAAAGTAATATTAAATA :  562
PI169588.3 : AATTCGAATTTACTAAAAATATCCGATTTAAACTAATTATCGTTTTGAGAATTAAAACATAATTAATGATAAATTATAATTAATAATTTTTATTTGATAAAATATGAAGATATTAAAAAGTAATATTAAATA :  562
Brandywine : ----------------------------------------------------------------------------------------------------------------------------------- :    -
EA00990.3  : AATTCGAATTTACTAAAAATACCCGATTTAAAATAATTATCATTTTGAGAATTAAAACATAATTAATAATAAATTATAATTTTAATTTTTATTTAATAAAATATGAAGATATTAAAAAGTAATATTTAAAT :  561
PI1303721. : AATTCGAATTTACTAAAAATATCCGATTTAAACTAATTATCGTTTTGAGAATTAAAACATAATTAATGATAAATTATAATTAATAATTTTTATTTGATAAAATATGAAGATATTAAAAAGTAATATTAAATA :  562
BGV006775. : AATTCGAATTTACTAAAAATATCCGATTTAAACTAATTATCGTTTTGAGAATTAAAACATTATTAATGATAAATTATAATTAATAATTTTTATTTAATAAAATATGAAGATATTAAAAAGTAATATTAAATA :  562
PAS014479. : AATTCGAATTTACTAAAAATACCCGATTTAAAATAATTATCATTTTGAGAATTAAAACATAATTAATAATAAATTATAATTTTAATTTTTATTTAATAAAATATGAAGATATTAAAAAGTAATATTAAATA :  562
             aattcgaatttactaaaaa a ccgatttaaa taattatc ttttgagaattaaaacat attaat ataaattataatt taatttttattt ataaaatatgaagatattaaaaagtaatatt aa
                                        epm_Soly_M0_p1m16R_193 804
                                          epm_Soly_M0_p0m11F_294 1500 805
                                     epm_Soly_M0_p0m11F_294 806

*      800         *       820         *       840         *       860         *       880         *       900         *
ITAG.3     : AATTTTGACAATAAGTTAATATTTTATTTTTTT-CTAAGAAAGAAGAAAAACATGATATGAATTGAAGGGAATAGTTGTAGTTATTACGCCCTTTGTCCACAATTGTTTTTCGAGATAATGTCGTGAACTC :  692
BGV006865. : AATTTTGACAATAAGTTAATGTTTTATTTTTTT-CTAAGAAAGAAGAAAAACATGATATGAATTTAAGGGAATAGTTGTAGTTATTACGCCCTTTGTCCACAATTGTTTTTCGAGATAATGTCGTGAACTC :  692
BGV007931. : AATTTTGACAATAAGTTAATATTTTATTTTTTT-CTAAGAAAGAAGAAAAACATGATATGAATTTAAGGGAATAGTTGTAGTTATTACGCCCTTTGTCCACAATTGTTTTTCGAGATAATGTCGTGAACTC :  692
BGV007989. : AATTTTGACAATAAGTTAATATTTTATTTTTTT-CTAAGAAAGAAGAAAAACATGATATGAATTGAAGGGAATAGTTGTAGTTATTACGCCCTTTGTCCACAATTGTTTTTCGAGATAATGTCGTGAACTC :  692
```

```
M82.3       : ----------------------------------------------------------------------------------------------------------------------------------- :    -
EA00371.3   : ----------------------------------------------------------------------------------------------------------------------------------- :    -
Fla.8924.3 : AATTTTGACAATAAGTTAATATTTTATTTTTTT-CTAAGAAAGAAGAAAAACATGATATGAATTGAAGGGAATAGTTGTAGTTATTACGCCCTTTGTCCACAATTGTTTTTCGAGATAATGTCGTGAACTC :  692
Floradade. : AATTTTGACAATAAGTTAATATTTTATTTTTTT-CTAAGAAAGAAGAAAAACATGATATGAATTGAAGGGAATAGTTGTAGTTATTACGCCCTTTGTCCACAATTGTTTTTCGAGATAATGTCGTGAACTC :  692
LYC1410.3  : AATTTTGACAATAAGTTAATATTTTATTTTTTT-CTAAGAAAGAAGAAAAACATGATATGAATTGAAGGGAATAGTTGTAGTTATTACGCCCTTTGTCCACAATTGTTTTTCGAGATAATGTCGTGAACTC :  692
PI169588.3 : AATTTTGACAATAAGTTAATATTTTATTTTTTT-CTAA  AAGAAGAAAAACATGATATGAATTGAAGGGAATAGTTGTAGTTATTACGCCCTTTGTCCACAATTGTTTTTCGAGATAATGTCGTGAACTC :  692
Brandywine : ----------------------------------------------------------------------------------------------------------------------------------- :    -
EA00990.3  : AAATTTTGACAATAAGTTAATATTTTATTTTTTTCTAAGAAAGAAGAAAAACATGATATGAATTGAAGGGAATAGTTGTAGTTATTACGCCCTTTGTCCACAATTGTTTTTCAAGATAATGTCGTGAACTC :  692
PI1303721. : AATTTTGACAATAAGTTAATATTTTATTTTTTT-CTAAGAAAGAAGAAAAACATGATATGAATTGAAGGGAATAGTTGTAGTTATTACGCCCTTTGTCCACAATTGTTTTTCGAGATAATGTCGTGAACTC :  692
BGV006775. : AATTTTGACAATAAGTTAATATTTTATTTTTTT-CTAA AAAGAAGAAAAACATGATATGAATTTAAGGGAATAGTTGTAGTTATTACGCCCTTTGTCCACAATTGTTTTTCGAGATAATGTCGTGAACTC :  692
PAS014479. : AATTTTGACAATAAGTTAATATTTTATTTTTTT-CTAAGAAAGAAGAAAAACATGATATGAATTGAAGGGAATAGTTGTAGTTATTACGCCCTTTGTCCACAATTGTTTTTCAAGATAATGTCGTGAACTC :  692
 aa ttt    a  a  t a   ttt  tttttt ctaagaaagaagaaaaacatgatatgaatt aagggaatagttgtagttattacgccctttgtccacaattgtttttc agataatgtcgtgaactc

              920         *       940         *       960         *       980         *      1000         *      1020         *      1040
ITAG.3      : TTCTCAATGAAATTTAATATAAGGTATAAATTAATTAATATAATTGTACTGTACCAAGAATAGTCTACATAACTTTTTTATTGAATAAAATGGAGTAATTATTAAGGATAGAATTGAAAAAATTGACTAAT :  823
BGV006865.  : TTCTCAATGAAATTTAATATAAGGTATAAATTAATTAATATAATTGTACTGTACCAAGAATAGTCTACATAACTTTTTTATTGAATAAAATGGAGTAATTATTAAGGATAGAATTGAAAAAATTGACTAAT :  823
BGV007931.  : TTCTCAATGAAATTTAATATAAGGTATAAATTAATTAATATAATTGTACTGTACCAAGAATAGTCTACATAACTTTTTTATTGAATAAAATGGAGTAATTATTAAGGATAGAATTGAAAAAATTGACTAAT :  823
BGV007989.  : TTCTCAATGAAATTTAATATAAGGTATAAATTAATTAATATAATTGTACTGTACCAAGAATAGTCTACATAACTTTTTTATTGAATAAAATGGAGTAATTATTAAGGATAGAATTGAAAAAATTGACTAAT :  823
M82.3       : ----------------------------------------------------------------------------------------------------------------------------------- :    -
EA00371.3   : ----------------------------------------------------------------------------------------------------------------------------------- :    -
Fla.8924.3  : TTCTCAATGAAATTTAATATAAGGTATAAATTAATTAATATAATTGTACTGTACCAAGAATAGTCTACATAACTTTTTTATTGAATAAAATGGAGTAATTATTAAGGATAGAATTGAAAAAATTGACTAAT :  823
Floradade.  : TTCTCAATGAAATTTAATATAAGGTATAAATTAATTAATATAATTGTACTGTACCAAGAATAGTCTACATAACTTTTTTATTGAATAAAATGGAGTAATTATTAAGGATAGAATTGAAAAAATTGACTAAT :  823
LYC1410.3   : TTCTCAATGAAATTTAATATAAGGTATAAATTAATTAATATAATTGTACTGTACCAAGAATAGTCTACATAACTTTTTTATTGAATAAAATGGAGTAATTATTAAGGATAGAATTGAAAAAATTGACTAAT :  823
PI169588.3  : TTCTCAATGAAATTTAATATAAGGTATAAATTAATTAATATAATTGTACTGTACCAAGAATAGTCTACATAACTTTTTTATTGAATAAAATGGAGTAATTATTAAGGATAGAATTGAAAAAATTGACTAAT :  823
Brandywine  : ----------------------------------------------------------------------------------------------------------------------------------- :    -
EA00990.3   : TTCTCAATGAAATTTAATATAAGGTATAAATTAATTAATATAATTGTACTGTACCAAGAATAGTCTACATAACTTTTTTATTGAATAAAATGGAGTAATTATTAAGGATAGAATTGAAAAAATTGACTAAT :  823
PI1303721.  : TTCTCAATGAAATTTAATATAAGGTATAAATTAATTAATATAATTGTACTGTACCAAGAATAGTCTACATAACTTTTTTATTGAATAAAATGGAGTAATTATTAAGGATAGAATTGAAAAAATTGACTAAT :  823
BGV006775.  : TTCTCAATGAAATTTAATATAAGGTATAAATTAATTAATATAATTGTACTGTACCAAGAATAGTCTACATAACTTTTTTATTGAATAAAATGGAGTAATTATTAAGGATAGAATTGAAAAAATTGACTAAT :  823
PAS014479.  : TTCTCAATGAAATTTAATATAAGGTATAAATTAATTAATATAATTGTACTGTACCAAGAATAGTCTACATAACTTTTTTATTGAATAAAATGGAGTAATTATTAAGGATAGAATTGAAAAAATTGACTAAT :  823
              ttctcaatgaaatttaatataaggtataaattaattaatataattgtactgtaccaagaatagtctacataactttttttattgaataaaatggagtaattattaaggatagaattgaaaaaattgactaat
              epm_Soly_M0_p0m16R_193 1047
TTTAAATTGACAATTAATCTTGATCAAACAATCGTGGACGGAAGAAGTAAATTTATTTTCTTTTTCTCTTTTTATAACAAAAGAAAATAAATAAAAAAAGAAAAAGAATAAAAAATATCTGTAACGGCT  :  954
BGV006865. : TTATTTAAATTGACAATTAATCTTGATCAAACAATCGTGGACGGAAGAAGTAAATTTATTTTCTTTTTCTCTTTTTATAACAAAAGAAAATAAATAAAAAAAGAAAAAGAATAAAAAATATCTGTAACGGCT :  954
BGV007931. : TTATTTAAATTGACAATTAATCTTGATCAAACAATCGTGGACGGAAGAAGTAAATTTATTTTCTTTTTCTCTTTTTATAACAAAAGAAAATAAATAAAAAAAGAAAAAGAATAAAAAATATCTGTAACGGCT :  954
BGV007989. : TTATTTAAATTGACAATTAATCTTGATCAAACAATCGTGGACGGAAGAAGTAAATTTATTTTCTTTTTCTCTTTTTATAACAAAAGAAAATAAATAAAAAAAGAAAAAGAATAAAAAATATCTGTAACGGCT :  954
M82.3      : ----------------------------------------------------------------------------------------------------------------------------------- :    -
EA00371.3  : ----------------------------------------------------------------------------------------------------------------------------------- :    -
Fla.8924.3 : TTATTTAAATTGACAATTAATCTTGATCAAACAATCGTGGACGGAAGAAGTAAATTTATTTTCTTTTTCTCTTTTTATAACAAAAGAAAATAAATAAAAAAAGAAAAAGAATAAAAAATATCTGTAACGGCT :  954
Floradade. : TTATTTAAATTGACAATTAATCTTGATCAAACAATCGTGGACGGAAGAAGTAAATTTATTTTCTTTTTCTCTTTTTATAACAAAAGAAAATAAATAAAAAAAGAAAAAGAATAAAAAATATCTGTAACGGCT :  954
LYC1410.3  : TTATTTAAATTGACAATTAATCTTGATCAAACAATCGTGGACGGAAGAAGTAAATTTATTTTCTTTTTCTCTTTTTATAACAAAAGAAAATAAATAAAAAAAGAAAAAGAATAAAAAATATCTGTAACGGCT :  954
PI169588.3 : TTATTTAAATTGACAATTAATCTTGATCAAACAATCGTGGACGGAAGAAGTAAATTTATTTTCTTTTTCTCTTTTTATAACAAAAGAAAATAAATAAAAAAAGAAAAAGAATAAAAAATATCTGTAACGGCT :  954
Brandywine : ----------------------------------------------------------------------------------------------------------------------------------- :    -
EA00990.3  : TTATTTAAATTGACAATTAATCTTGATCAAACAATCGTAGACGGAAGGAGTAAATTTATTTTCTTTTTCTCTTTTTATAACAAAAGAAAATAAATAAAAAAAGAAAAAGAATAAAAAATATCTGTAACGGCT :  954
PI1303721. : TTATTTAAATTGACAATTAATCTTGATCAAACAATCGTGGACGGAAGAAGTAAATTTATTTTCTTTTTCTCTTTTTATAACAAAAGAAAATAAATAAAAAAAGAAAAAGAATAAAAAATATCTGTAACGGCT :  954
BGV006775. : TTATTTAAATTGACAATTAATCTTGATCAAACAATCGTGGACGGAAGAAGTAAATTTATTTTCTTTTTCTCTTTTTATAACAAAAGAAAATAAATAAAAAAAGAAAAAGAATAAAAAATATCTGTAACGGCT :  954
PAS014479. : TTATTTAAATTGACAATTAATCTTGATCAAACAATCGTAGACGGAAGGAGTAAATTTATTTTCTTTTTCTCTTTTTATAACAAAAGAAAATAAATAAAAAAAGAAAAAGAATAAAAAATATCTGTAACGGCT :  954
 ttatttaaattgacaattaatcttgatcaaacaatcgt gacggaag agtaaatttattttctttttctctttttataacaaaagaaaataaataaaaaaagaaaaagaataaaaaatatctgtaacggct

                                                                                                      epm_Soly_M0_p1m03F_636 1281
                                                                                                      epm_Soly_M0_p1m11F_213  1281
              0         *      1200         *      1220         *      1240         *      1260         *      1280         *      1300         *
ITAG.3      : CGGATTTTGATATATAAAAGGCCACGTAAGTAACTTGGTTTCTTCCGTCAAATGGCTCATGGCTTATAACTTCCTATCCAGTCATCACGCTATCTTCACTTTCTCTTCTTCTTCCTCTCCAATTTCATGGA : 1085
BGV006865.  : CGGATTTTGATATATAAAAGGCCACGTAAGTAACTTGGTTTCTTCCGTCAAATGGCTCATGGCTTATAACTTCCTATCCAGTCATCACGCTATCTTCACTTTCTCTTCTTCTTCCTCTCCAATTTCATGGA : 1085
BGV007931.  : CGGATTTTGATATATAAAAGGCCACGTAAGTAACTTGGTTTCTTCCGTCAAATGGCTCATGGCTTATAACTTCCTATCCAGTCATCACGCTATCTTCACTTTCTCTTCTTCTTCCTCTCCAATTTCATGGA : 1085
BGV007989.  : CGGATTTTGATATATAAAAGGCCACGTAAGTAACTTGGTTTCTTCCGTCAAATGGCTCATGGCTTATAACTTCCTATCCAGTCATCACGCTATCTTCACTTTCTCTTCTTCTTCCTCTCCAATTTCATGGA : 1085
M82.3       : ----------------------------------------------------------------------------------------------------------------------------------- :    -
EA00371.3   : ----------------------------------------------------------------------------------------------------------------------------------- :    -
Fla.8924.3  : CGGATTTTGATATATAAAAGGCCACGTAAGTAACTTGGTTTCTTCCGTCAAATGGCTCATGGCTTATAACTTCCTATCCAGTCATCACGCTATCTTCACTTTCTCTTCTTCTTCCTCTCCAATTTCATGGA : 1085
Floradade.  : CGGATTTTGATATATAAAAGGCCACGTAAGTAACTTGGTTTCTTCCGTCAAATGGCTCATGGCTTATAACTTCCTATCCAGTCATCACGCTATCTTCACTTTCTCTTCTTCTTCCTCTCCAATTTCATGGA : 1085
LYC1410.3   : CGGATTTTGATATATAAAAGGCCACGTAAGTAACTTGGTTTCTTCCGTCAAATGGCTCATGGCTTATAACTTCCTATCCAGTCATCACGCTATCTTCACTTTCTCTTCTTCTTCCTCTCCAATTTCATGGA : 1085
PI169588.3  : CGGATTTTGATATATAAAAGGCCACGTAAGTAACTTGGTTTCTTCCGTCAAATGGCTCATGGCTTATAACTTCCTATCCAGTCATCACGCTATCTTCACTTTCTCTTCTTCTTCCTCTCCAATTTCATGGA : 1085
Brandywine  : ----------------------------------------------------------------------------------------------------------------------------------- :    -
EA00990.3   : CGGATTTTGATATATAAAAGGCCACGTAAGTAACTTGGTTTCTTCCGTCAAATGGCTCATGGCTTATAACTTCCTATCCAGTCATCACGCTATCTTCACTTTCTCTTCTTCTTCCTCTCCAATTTCATGGA : 1085
PI1303721.  : CGGATTTTGATATATAAAAGGCCACGTAAGTAACTTGGTTTCTTCCGTCAAATGGCTCATGGCTTATAACTTCCTATCCAGTCATCACGCTATCTTCACTTTCTCTTCTTCTTCCTCTCCAATTTCATGGA : 1085
BGV006775.  : CGGATTTTGATATATAAAAGGCCACGTAAGTAACTTGGTTTCTTCCGTCAAATGGCTCATGGCTTATAACTTCCTATCCAGTCATCACGCTATCTTCACTTTCTCTTCTTCTTCCTCTCCAATTTCATGGA : 1085
PAS014479.  : CGGATTTTGATATATAAAAGGCCACGTAAGTAACTTGGTTTCTTCCGTCAAATGGCTCATGGCTTATAACTTCCTATCCAGTCATCACGCTATCTTCACTTTCTCTTCTTCTTCCTCTCCAATTTCATGGA : 1085
              cggattttgatatataaaaggccacgtaagtaacttggtttcttccgtcaaatggctcatggcttataacttcctatccagtcatcacgctatcttcactttctcttcttcttcctctccaatttcatgga

                                   epm_Soly_M0_p0m06R_527 1330
                                                    epm_Soly_M0_p0m05R_586 1355                                              epm_Soly_M0_p1m03R_719 1436
                                                     epm_Soly_M0_p0m05R_586 1356                                            epm_Soly_M0_p0m05F_54 1435
                                                    epm_Soly_M0_p1m08F_108 1355
                                                                epm_Soly_M0_p0m09R_382 1369
1269
                  1320         *      1340         *      1360         *      1380         *      1400         *      1420         *      1440
ITAG.3      : CTCAATCTTCTCTCCAAAACTTTCTCTCCTTTCTTGTTCTCCAATCTCCTCTTCTCTCGTTCCTCGCCGCCAATTTCTCTCCGGTTCTACCCATTCT---TTACGGCCTCCTGGC---CTTCACTCGCGGA : 1210
BGV006865.  : CTCAATCTTCTCTCCAAAACTTTCTCTCCTTTCTTGTTCTCCAATCTCCTCTTCTCTCGTTCCTCGCCGCCAATTTCTCTCCGGTTTTACCCATTCT---TTACGGCCTCCTGGC---CTTCACTCGCGGA : 1210
BGV007931.  : CTCAATCTTCTCTCCAAAACTTTCTCTCCTTTCTTGTTCTCCAATCTCCTCTTCTCTCGTTCCTCGCCGCCAATTTCTCTCCGGTTTTACCCATTCT---TTACGGCCTCCTGGC---CTTCACTCGCGGA : 1210
BGV007989.  : CTCAATCTTCTCTCCAAAACTTTCTCTCCTTTCTTGTTCTCCAATCTCCTCTTCTCTCGTTCCTCGCCGCCAATTTCTCTCCGGTTCTACCCATTCT---TTACGGCCTCCTGGC---CTTCACTCGCGGA : 1210
M82.3       : ----------------------------------------------------------------------------------------------------------------------------------- :    -
EA00371.3   : ----------------------------------------------------------------------------------------------------------------------------------- :    -
Fla.8924.3  : CTCAATCTTCTCTCCAAAACTTTCTCTCCTTTCTTGTTCTCCAATCTCCTCTTCTCTCGTTCCTCGCCGCCAATTTCTCTCCGGTTCTACCCATTCT---TTACGGCCTCCTGGC---CTTCACTCGCGGA : 1210
Floradade.  : CTCAATCTTCTCTCCAAAACTTTCTCTCCTTTCTTGTTCTCCAATCTCCTCTTCTCTCGTTCCTCGCCGCCAATTTCTCTCCGGTTCTACCCATTCT---TTACGGCCTCCTGGC---CTTCACTCGCGGA : 1210
LYC1410.3   : CTCAATCTTCTCTCCAAAACTTTCTCTCCTTTCTTGTTCTCCAATCTCCTCTTCTCTCGTTCCTCGCCGCCAATTTCTCTCCGGTTCTACCCATTCT---TTACGGCCTCCTGGC---CTTCACTCGCGGA : 1210
PI169588.3  : CTCAATCTTCTCTCCAAAACTTTCTCTCCTTTCTTGTTCTCCAATCTCCTCTTCTCTCGTTCCTCGCCGCCAATTTCTCTCCGGTTCTACCCATTCT---TTACGGCCTCCTGGC---CTTCACTCGCGGA : 1210
Brandywine  : ----------------------------------------------------------------------------------------------------------------------------------- :    -
EA00990.3   : CTCAATCTTCTCTCCAAAACTTTCTCTCCTTTCTTGTTCTCCAATCTCCTCTTCTCTCGTTCCTCGCCGCCAATTTCTCTCCGGTTCTACCCATTCT---TTACGGCCTCCTGGC---CTTCACTCGCGGA : 1210
PI1303721.  : CTCAATCTTCTCTCCAAAACTTTCTCTCCTTTCTTGTTCTCCAATCTCCTCTTCTCTCGTTCCTCGCCGCCAATTTCTCTCCGGTTCTACCCATTCT---TTACGGCCTCCTGGC---CTTCACTCGCGGA : 1210
BGV006775.  : CTCAATCTTCTCTCCAAAACTTTCTCTCCTTTCTTGTTCTCCAATCTCCTCTTCTCTCGTTCCTCGCCGCCAATTT  CTCCGGTTTTACCCATTCT---TTACGGCCTCCCGGC---CTTCACTCGCGGA : 1210
PAS014479.  : CTCAATCTTCTCTCCAAAACTTTCTCTCCTTTCTTGTTCTCCAATCTCCTCTTCTCTCGTTCCTCGCCGCCAATTTCTCTCCGGTTCTACCCATTCT---TTACGGCCTCCTGGC---CTTCACTCGCGGA : 1210
              ctcaatcttctctccaaaactttctctcctttcttgttctccaatctcctcttctctcgttcctcgccgccaatttctctccggtt tacccattct   ttacggcctcc ggc   cttcactcgcgga

                    *      1460         *      1480         *      1500         *      1520         *      1540         *      1560         *
ITAG.3      : GGAGATGTCGAAATATCGGCTTTCAATTCGGCGGCAACACTTCAAGATTTGTTCTCAGAGCTTCTCTTGACTCGCAAACCGTAGTTTTTGCATCAGTTGTCACTATCTCCGCTCTTACAGTCGTCTTTCTT : 1341
BGV006865.  : GGAGATGTCGAAATATCGGCTTTCAATTCGGCGGCAACACTTCAAGATTTGTTCTCAGAGCTTCTCTTGACTCGCAAACCGTAGTTTTTGCATCAGTTGTCACTATCTCCGCTCTTACAGTCGTCTTTCTT : 1341
BGV007931.  : GGAGATGTCGAAATATCGGCTTTCAATTCGGCGGCAACACTTCAAGATTTGTTCTCAGAGCTTCTCTTGACTCGCAAACCGTAGTTTTTGCATCAGTTGTCACTATCTCCGCTCTTACAGTCGTCTTTCTT : 1341
BGV007989.  : GGAGATGTCGAAATATCGGCTTTCAATTCGGCGGCAACACTTCAAGATTTGTTCTCAGAGCTTCTCTTGACTCGCAAACCGTAGTTTTTGCATCAGTTGTCACTATCTCCGCTCTTACAGTCGTCTTTCTT : 1341
M82.3       : -----------------------------------------------------------GCTTCTCTTGACTCGCTAAAGGTAGTTTTTGCATCCGTTGTCACTATCTTTGCTCTTATAATCATCTTTCTT :  296
EA00371.3   : -----------------------------------------------------------GCTTCTCTTGACTCGCTAAAGGTAGTTTTTGCATCCGTTGTCACTATCTTTGCTCTTATAATCATCTTTCTT :  296
Fla.8924.3  : GGAGATGTCGAAATATCGGCTTTCAATTCGGCGGCAACACTTCAAGATTTGTTCTCAGAGCTTCTCTTGACTCGCAAACCGTAGTTTTTGCATCAGTTGTCACTATCTCCGCTCTTACAGTCGTCTTTCTT : 1341
Floradade.  : GGAGATGTCGAAATATCGGCTTTCAATTCGGCGGCAACACTTCAAGATTTGTTCTCAGAGCTTCTCTTGACTCGCAAACCGTAGTTTTTGCATCAGTTGTCACTATCTCCGCTCTTACAGTCGTCTTTCTT : 1341
LYC1410.3   : GGAGATGTCGAAATATCGGCTTTCAATTCGGCGGCAACACTTCAAGATTTGTTCTCAGAGCTTCTCTTGACTCGCAAACCGTAGTTTTTGCATCAGTTGTCACTATCTCCGCTCTTACAGTCGTCTTTCTT : 1341
PI169588.3  : GGAGATGTCGAAATATCGGCTTTCAATTCGGCGGCAACACTTCAAGATTTGTTCTCAGAGCTTCTCTTGACTCGCAAACCGTAGTTTTTGCATCAGTTGTCACTATCTCCGCTCTTACAGTCGTCTTTCTT : 1341
Brandywine  : -----------------------------------------------------------GCTTCTCTTGACTCGCTAAAGGTAGTTTTTGCATCCGTTGTCACTATCTTTGCTCTTATAATCATCTTTCTT :  296
EA00990.3   : GGAGATGTCGAAATATCGGCTTTCAATTCGGCGGCAACTCTTCAAGATTTGTTCTCAGAGCTTCTCTTGACTCGCAAACCGTAGTTTTTGCATCAGTTGTCACTATCTCCGCTCTTACAGTCGTCTTTCTT : 1341
PI1303721.  : GGAGATGTCGAAATATCGGCTTTCAATTCGGCGGCAACACTTCAAGATTTGTTCTCAGAGCTTCTCTTGACTCGCAAACCGTAGTTTTTGCATCAGTTGTCACTATCTCCGCTCTTACAGTCGTCTTTCTT : 1341
BGV006775.  : GGAGATGTCGAAATATCGGCTTTCAATTCGGCGGCAACACTTCAAGATTTGTTCTCAGAGCTTCTCTTGACTCGCAAACCGTAGTTTTTGCATCAGTTGTCACTATCTCCGCTCTTACAGTCGTCTTTCTT : 1341
PAS014479.  : GGAGATGTCGAAATATCGGCTTTCAATTCGGCGGCAACTCTTCAAGATTTGTTCTCAGAGCTTCTCTTGACTCGCAAACCGTAGTTTTTGCATCAGTTGTCACTATCTCCGCTCTTACAGTCGTCTTTCTT : 1341
              ggagatgtcgaaatatcggctttcaattcggcggcaac cttcaagatttgttctcagaGCTTCTCTTGACTCGCaAAccGTAGTTTTTGCATCaGTTGTCACTATCTccGCTCTTAcAgTCgTCTTTCTT
                                        epm_Soly_M0_p1m07R_163 1605
                                          epm_Soly_M0_p1m03R_719 1608

                 1580         *      1600         *      1620         *      1640         *      1660         *      1680         *      1700
ITAG.3      : GAATTTTCCAAAAGAAATACCAATGCCAACGCCAAGTTCAAGGAGGTAAGCTATACTCTGTGAATAACATAAACGCTTCACCTTTCTTTTCATGAAATAGACGCCGA------------------------ : 1448
BGV006865.  : GAATTTTCCAAAAGAAATACCAATGCCAACGCCAAGTTCAAGGAGGTAAGCTATACTCTGTGAATAACATAAACGCTTCACCTTTCTTTTCATGAAATAGACGCCGA------------------------ : 1448
BGV007931.  : GAATTTTCCAAAAGAAATACCAATGCCAACGCCAAGTTCAAGGAGGTAAGCTATACTCTGTGAATAACATAAACGCTTCACCTTTCTTTTCATGAAATAGACGCCGA------------------------ : 1448
BGV007989.  : GAATTTTCCAAAAGAAATACCAATGCCAACGCCAAGTTCAAGGAGGTAAGCTATACTCTGTGAATAACATAAACGCTTCACCTTTCTTTTCATGAAATAGACGCCGA------------------------ : 1448
M82.3       : GAATTTTCCAAAATAAATACCAATGCCAACGCCAAGTTCAAAGAGGTAAACTCTACTTTGTGAATAACATTACCGGTTCACTTTTTACTGTTATTTCTTGAAAGAGACCAATTTCTTGTAAGCCTAGCAAT :  427
EA00371.3   : GAATTTTCCAAAATAAATACCAATGCCAACGCCAAGTTCAAAGAGGTAAACTCTACTTTGTGAATAACATTACCGGTTCACTTTTTACTGTTATTTCTTGAAAGAGACCAATTTCTTGTAAGCCTAGCAAT :  427
Fla.8924.3  : GAATTTTCCAAAAGAAATACCAATGCCAACGCCAAGTTCAAGGAGGTAAGCTATACTCTGTGAATAACATAAACGCTTCACCTTTCTTTTCATGAAATAGACGCCGA------------------------ : 1448
Floradade.  : GAATTTTCCAAAAGAAATACCAATGCCAACGCCAAGTTCAAGGAGGTAAGCTATACTCTGTGAATAACATAAACGCTTCACCTTTCTTTTCATGAAATAGACGCCGA------------------------ : 1448
LYC1410.3   : GAATTTTCCAAAAGAAATACCAATGCCAACGCCAAGTTCAAGGAGGTAAGCTATACTCTGTGAATAACATAAACGCTTCACCTTTCTTTTCATGAAATAGACGCCGA------------------------ : 1448
PI169588.3  : GAATTTTCCAAAAGAAATACCAATGCCAACGCCAAGTTCAAGGAGGTAAGCTATACTCTGTGAATAACATAAACGCTTCACCTTTCTTTTCATGAAATAGACGCCGA------------------------ : 1448
Brandywine  : GAATTTTCCAAAATAAATACCAATGCCAACGCCAAGTTCAAAGAGGTAAACTCTACTTTGTGAATAACATTACCGGTTCACTTTTTACTGTTATTTCTTGAAAGAGACCAATTTCTTGTAAGCCTAGCAAT :  427
EA00990.3   : GAATTTTCCAAAAGAAATACCAATGCCAACGCCAAGTTCAAGGAGGTAAGCTATACTCTGTGAATAACATAAACGCTTCACCTTTCTTTTCATGAAATAGACGCCGA------------------------ : 1448
PI1303721.  : GAATTTTCCAAAAGAAATACCAATGCCAACGCCAAGTTCAAGGAGGTAAGCTATACTCTGTGAATAACATAAACGCTTCACCTTTCTTTTCATGAAATAGACGCCGA------------------------ : 1448
BGV006775.  : GAATTTTCCAAAAGAAATACCAATGCCAACGCCAAGTTCAAGGAGGTAAGCTATACTCTGTGAATAACATAAACGCTTCACCTTTCTTTTCATGAAATAGACGCCGA------------------------ : 1448
PAS014479.  : GAATTTTCCAAAAGAAATACCAATGCCAACGCCAAGTTCAAGGAGGTAAGCTATACTCTGTGAATAACATAAACGCTTCACCTTTCTTTTCATGAAATAGACGCCGA------------------------ : 1448
              GAATTTTCCAAAAgAAATACCAATGCCAACGCCAAGTTCAAgGAGGTAAgCTaTACTcTGTGAATAACATaAaCGcTTCACcTTTcttTtcatgaaaTaGAcgccGA
                                   epm_Soly_M0_p0m07R_528 1739
*     1720         *      1740         *      1760         *      1780         *      1800         *      1820         *
ITAG.3      : --------------------------------------------------------------------------TTTCTTGATTCTTGAAAATTTTGTATTGTAAAATCAAGAATGTAGTTG-------- : 1496
```

```
BGV006865. : ----------------------------------------------------------------------------TTTCTTGATTCTTGAAAATTTTGTATTGTAAAATCAAGAATGTAGTTG------- : 1496
BGV007931. : ----------------------------------------------------------------------------TTTCTTGATTCTTGAAAATTTTGTATTGTAAAATCAAGAATGTAGTTG------- : 1496
BGV007989. : ----------------------------------------------------------------------------TTTCTTGATTCTTGAAAATTTTGTATTGTAAAATCAAGAATGTAGTTG------- : 1496
M82.3      : TTCTCGGTGTATAACTTTACTAGTTAACTTTTGTTGCCAAATACAGAAAGAGGATGTGTCTTGAGTCTGTATACGTCTTAGTGATTCT-----ATTGTGTTGTGTAGAATCAAGAATGTAGATGTTGATAG :  553
EA00371.3  : TTCTCGGTGTATAACTTTACTAGTTAACTTTTGTTGCCAAATACAGAAAGAGGATGTGTCTTGAGTCTGTATACGTCTTAGTGATTCT-----ATTGTGTTGTGTAGAATCAAGAATGTAGATGTTGATAG :  553
Fla.8924.3 : ----------------------------------------------------------------------------TTTCTTGATTCTTGAAAATTTTGTATTGTAAAATCAAGAATGTAGTTG------- : 1496
Floradade. : ----------------------------------------------------------------------------TTTCTTGATTCTTGAAAATTTTGTATTGTAAAATCAAGAATGTAGTTG------- : 1496
LYC1410.3  : ----------------------------------------------------------------------------TTTCTTGATTCTTGAAAATTTTGTATTGTAAAATCAAGAATGTAGTTG------- : 1496
PI169588.3 : ----------------------------------------------------------------------------TTTCTTGATTCTTGAAAATTTTGTATTGTAAAATCAAGAATGTAGTTG------- : 1496
Brandywine : TTCTCGGTGTATAACTTTACTAGTTAACTTTTGTTGCCAAATACAGAAAGAGGATGTGTCTTGAGTCTGTATACGTCTTAGTGATTCT-----ATTGTGTTGTGTAGAATCAAGAATGTAGATGTTGATAG :  553
EA00990.3  : ----------------------------------------------------------------------------TTTCTTGATTCTTGAAAATTTTGTATTGTAAAATCAAGAATGTAGTTG------- : 1496
PI1303721. : ----------------------------------------------------------------------------TTTCTTGATTCTTGAAAATTTTGTATTGTAAAATCAAGAATGTAGTTG------- : 1496
BGV006775. : ----------------------------------------------------------------------------TTTCTTGATTCTTGAAAATTTTGTATTGTAAAATCAAGAATGTAGTTG------- : 1496
PAS014479. : ----------------------------------------------------------------------------TTTCTTGATTCTTGAAAATTTTGTATTGTAAAATCAAGAATGTAGTTG------- : 1496
                                                                                         tTTctTGATTCTtgaaaATTtTGTatTGTAaAATCAAGAATGTAGtTG

               epm_Soly_M0_p1m04F_296 1840
                 epm_Soly_M0_p1m04R_296 1841                                                      epm_Soly_M0_p0m18R_163 1935

               1840         *       1860         *       1880         *       1900         *       1920         *       1940         *       1960
ITAG.3     : ----------------------------------------------------------------------------------------------------------------------------------- :    -
BGV006865. : ----------------------------------------------------------------------------------------------------------------------------------- :    -
BGV007931. : ----------------------------------------------------------------------------------------------------------------------------------- :    -
BGV007989. : ----------------------------------------------------------------------------------------------------------------------------------- :    -
M82.3      : CTAATTTCTATCCTCATCATTTAACATAGATCATCATTTGTTCAGTACACATGCTGTTTGATTGCAACAAGGCTAACTCTTAATTTTCCAATCTTCAATTAGTATTTGGAGTTGTATATCTCAGTAATATG :  684
EA00371.3  : CTAATTTCTATCCTCATCATTTAACATAGATCATCATTTGTTCAGTACACATGCTGTTTGATTGCAACAAGGCTAACTCTTAATTTTCCAATCTTCAATTAGTATTTGGAGTTGTATATCTCAGTAATATG :  684
Fla.8924.3 : ----------------------------------------------------------------------------------------------------------------------------------- :    -
Floradade. : ----------------------------------------------------------------------------------------------------------------------------------- :    -
LYC1410.3  : ----------------------------------------------------------------------------------------------------------------------------------- :    -
PI169588.3 : ----------------------------------------------------------------------------------------------------------------------------------- :    -
Brandywine : CTAATTTCTATCCTCATCATTTAACATAGATCATCATTTGTTCAGTACACATGCTGTTTGATTGCAACAAGGCTAACTCTTAATTTTCCAATCTTCAATTAGTATTTGGAGTTGTATATCTCAGTAATATG :  684
EA00990.3  : ----------------------------------------------------------------------------------------------------------------------------------- :    -
PI1303721. : ----------------------------------------------------------------------------------------------------------------------------------- :    -
BGV006775. : ----------------------------------------------------------------------------------------------------------------------------------- :    -
PAS014479. : ----------------------------------------------------------------------------------------------------------------------------------- :    -


                 *       1980         *       2000         *       2020         *       2040         *       2060         *       2080         *
ITAG.3     : ----------------------------------------------------------------------------------------------------------------------------------- :    -
BGV006865. : ----------------------------------------------------------------------------------------------------------------------------------- :    -
BGV007931. : ----------------------------------------------------------------------------------------------------------------------------------- :    -
BGV007989. : ----------------------------------------------------------------------------------------------------------------------------------- :    -
M82.3      : AAAGTGAAATATTATCCTAGTTGTTAATATGTCAGAACATGTTTCTCTTCAGATAAGTTCTTGGTCTTAGAAGTCATCTTGGAAACATTGAGTCCTCTACCATCTCAACTCTTAGTTGTTGGATAAATTTA :  815
EA00371.3  : AAAGTGAAATATTATCCTAGTTGTTAATATGTCAGAACATGTTTCTCTTCAGATAAGTTCTTGGTCTTAGAAGTCATCTTGGAAACATTGAGTCCTCTACCATCTCAACTCTTAGTTGTTGGATAAATTTA :  815
Fla.8924.3 : ----------------------------------------------------------------------------------------------------------------------------------- :    -
Floradade. : ----------------------------------------------------------------------------------------------------------------------------------- :    -
LYC1410.3  : ----------------------------------------------------------------------------------------------------------------------------------- :    -
PI169588.3 : ----------------------------------------------------------------------------------------------------------------------------------- :    -
Brandywine : AAAGTGAAATATTATCCTAGTTGTTAATATGTCAGAACATGTTTCTCTTCAGATAAGTTCTTGGTCTTAGAAGTCATCTTGGAAACATTGAGTCCTCTACCATCTCAACTCTTAGTTGTTGGATAAATTTA :  815
EA00990.3  : ----------------------------------------------------------------------------------------------------------------------------------- :    -
PI1303721. : ----------------------------------------------------------------------------------------------------------------------------------- :    -
BGV006775. : ----------------------------------------------------------------------------------------------------------------------------------- :    -
PAS014479. : ----------------------------------------------------------------------------------------------------------------------------------- :    -


             2100         *       2120         *       2140         *       2160         *       2180         *       2200         *       2220
ITAG.3     : ----------------------------------------------------------------------------------------------------------------------------------- :    -
BGV006865. : ----------------------------------------------------------------------------------------------------------------------------------- :    -
BGV007931. : ----------------------------------------------------------------------------------------------------------------------------------- :    -
BGV007989. : ----------------------------------------------------------------------------------------------------------------------------------- :    -
M82.3      : TAGAAACTGAAAATATTATGTCTGAAGTTTTCTGGTCATTGGCATCTGTGGTAAGTCAAATTTTTTAGTCATTGGTACTTAAAAGTAGGAATATTTTTTTCTAAAACACGTTAGCAAGAGGTGTTACAGTT :  946
EA00371.3  : TAGAAACTGAAAATATTATGTCTGAAGTTTTCTGGTCATTGGCATCTGTGGTAAGTCAAATTTTTTAGTCATTGGTACTTAAAAGTAGGAATATTTTTTTCTAAAACACGTTAGCAAGAGGTGTTACAGTT :  946
Fla.8924.3 : ----------------------------------------------------------------------------------------------------------------------------------- :    -
Floradade. : ----------------------------------------------------------------------------------------------------------------------------------- :    -
LYC1410.3  : ----------------------------------------------------------------------------------------------------------------------------------- :    -
PI169588.3 : ----------------------------------------------------------------------------------------------------------------------------------- :    -
Brandywine : TAGAAACTGAAAATATTATGTCTGAAGTTTTCTGGTCATTGGCATCTGTGGTAAGTCAAATTTTTTAGTCATTGGTACTTAAAAGTAGGAATATTTTTTTCTAAAACACGTTAGCAAGAGGTGTTACAGTT :  946
EA00990.3  : ----------------------------------------------------------------------------------------------------------------------------------- :    -
PI1303721. : ----------------------------------------------------------------------------------------------------------------------------------- :    -
BGV006775. : ----------------------------------------------------------------------------------------------------------------------------------- :    -
PAS014479. : ----------------------------------------------------------------------------------------------------------------------------------- :    -

                                                                                                                              epm_Soly_M0_p1m00R_9780 2344

               *       2240         *       2260         *       2280         *       2300         *       2320         *       2340         *       23
ITAG.3     : ----------------------------------------------------------------------------------------------------------------------------------- :    -
BGV006865. : ----------------------------------------------------------------------------------------------------------------------------------- :    -
BGV007931. : ----------------------------------------------------------------------------------------------------------------------------------- :    -
BGV007989. : ----------------------------------------------------------------------------------------------------------------------------------- :    -
M82.3      : TTACCAGAGTTACTTCAGGAGGTCCATATTTCTGATGTCCACAATTTTTCATAGATTTCAGCTGAGTTAACTCTTGCTTTGCGGCGACAAATAAGACATGTGATGAACTGGTTTCCACGTCATGTATTTGC : 1077
EA00371.3  : TTACCAGAGTTACTTCAGGAGGTCCATATTTCTGATGTCCACAATTTTTCATAGATTTCAGCTGAGTTAACTCTTGCTTTGCGGCGACAAATAAGACATGTGATGAACTGGTTTCCACGTCATGTATTTGC : 1077
Fla.8924.3 : ----------------------------------------------------------------------------------------------------------------------------------- :    -
Floradade. : ----------------------------------------------------------------------------------------------------------------------------------- :    -
LYC1410.3  : ----------------------------------------------------------------------------------------------------------------------------------- :    -
PI169588.3 : ----------------------------------------------------------------------------------------------------------------------------------- :    -
Brandywine : TTACCAGAGTTACTTCAGGAGGTCCATATTTCTGATGTCCACAATTTTTCATAGATTTCAGCTGAGTTAACTCTTGCTTTGCGGCGACAAATAAGACATGTGATGAACTGGTTTCCACGTCATGTATTTGC : 1077
EA00990.3  : ----------------------------------------------------------------------------------------------------------------------------------- :    -
PI1303721. : ----------------------------------------------------------------------------------------------------------------------------------- :    -
BGV006775. : ----------------------------------------------------------------------------------------------------------------------------------- :    -
PAS014479. : ----------------------------------------------------------------------------------------------------------------------------------- :    -


             60         *       2380         *       2400         *       2420         *       2440         *       2460         *       2480
ITAG.3     : ----------------------------------------------------------------------------------------------------------------------------------- :    -
BGV006865. : ----------------------------------------------------------------------------------------------------------------------------------- :    -
BGV007931. : ----------------------------------------------------------------------------------------------------------------------------------- :    -
BGV007989. : ----------------------------------------------------------------------------------------------------------------------------------- :    -
M82.3      : TCTTATAAACATACAGGAGGAGAAATCTGTCAAAACTCAAATGAAGGAAGTAACCAAAGTCAGCAATGAGCGTGAGGATGGGGAGGCAGATGTGCTGCAACATGATGGAACCTATTTGATACAAACGTTTG : 1208
EA00371.3  : TCTTATAAACATACAGGAGGAGAAATCTGTCAAAACTCAAATGAAGGAAGTAACCAAAGTCAGCAATGAGCGTGAGGATGGGGAGGCAGATGTGCTGCAACATGATGGAACCTATTTGATACAAACGTTTG : 1208
Fla.8924.3 : ----------------------------------------------------------------------------------------------------------------------------------- :    -
Floradade. : ----------------------------------------------------------------------------------------------------------------------------------- :    -
LYC1410.3  : ----------------------------------------------------------------------------------------------------------------------------------- :    -
PI169588.3 : ----------------------------------------------------------------------------------------------------------------------------------- :    -
Brandywine : TCTTATAAACATACAGGAGGAGAAATCTGTCAAAACTCAAATGAAGGAAGTAACCAAAGTCAGCAATGAGCGTGAGGATGGGGAGGCAGATGTGCTGCAACATGATGGAACCTATTTGATACAAACGTTTG : 1208
EA00990.3  : ----------------------------------------------------------------------------------------------------------------------------------- :    -
PI1303721. : ----------------------------------------------------------------------------------------------------------------------------------- :    -
BGV006775. : ----------------------------------------------------------------------------------------------------------------------------------- :    -
PAS014479. : ----------------------------------------------------------------------------------------------------------------------------------- :    -


             *       2500         *       2520         *       2540         *       2560         *       2580         *       2600         *       2620
ITAG.3     : ----------------------------------------------------------------------------------------------------------------------------------- :    -
BGV006865. : ----------------------------------------------------------------------------------------------------------------------------------- :    -
BGV007931. : ----------------------------------------------------------------------------------------------------------------------------------- :    -
BGV007989. : ----------------------------------------------------------------------------------------------------------------------------------- :    -
M82.3      : TTACCAACAATATTGAGTCTCTAGATACTAATCAGTTGGCTCCTAGTTCCAATGGCTCTTTGACCTTGGGAGCATCTGTACCTAATGAACACACTGAATCAGATGCAGTACCATCTTCTTTTGTGGCAGAT : 1339
EA00371.3  : TTACCAACAATATTGAGTCTCTAGATACTAATCAGTTGGCTCCTAGTTCCAATGGCTCTTTGACCTTGGGAGCATCTGTACCTAATGAACACACTGAATCAGATGCAGTACCATCTTCTTTTGTGGCAGAT : 1339
Fla.8924.3 : ----------------------------------------------------------------------------------------------------------------------------------- :    -
Floradade. : ----------------------------------------------------------------------------------------------------------------------------------- :    -
LYC1410.3  : ----------------------------------------------------------------------------------------------------------------------------------- :    -
PI169588.3 : ----------------------------------------------------------------------------------------------------------------------------------- :    -
Brandywine : TTACCAACAATATTGAGTCTCTAGATACTAATCAGTTGGCTCCTAGTTCCAATGGCTCTTTGACCTTGGGAGCATCTGTACCTAATGAACACACTGAATCAGATGCAGTACCATCTTCTTTTGTGGCAGAT : 1339
EA00990.3  : ----------------------------------------------------------------------------------------------------------------------------------- :    -
PI1303721. : ----------------------------------------------------------------------------------------------------------------------------------- :    -
BGV006775. : ----------------------------------------------------------------------------------------------------------------------------------- :    -
PAS014479. : ----------------------------------------------------------------------------------------------------------------------------------- :    -
                                                                                                                                                       !
!br0ken!!

!!br0ken!!!!br0ken!!!!br0ken!!                        *       2640         *       2660         *       2680         *       2700         *       2720         *       2740
*
```

```
ITAG.3      : ----------------------------------------------------------------------------------------------------------------------------------- :    -
BGV006865.  : ----------------------------------------------------------------------------------------------------------------------------------- :    -
BGV007931.  : ----------------------------------------------------------------------------------------------------------------------------------- :    -
BGV007989.  : ----------------------------------------------------------------------------------------------------------------------------------- :    -
M82.3       : TCGAATAACATTTATCTGCAGGAAAATATACAGACGACTAAAATGAGTAACATATTGACTACAGAGGAAGTCAGAGAACCTGAACCTATTGCACACACTGAATCAGATGTAGTACCATCTTCTTTTGTGGC : 1470
EA00371.3   : TCGAATAACATTTATCTGCAGGAAAATATACAGACGACTAAAATGAGTAACATATTGACTACAGAGGAAGTCAGAGAACCTGAACCTATTGCACACACTGAATCAGATGTAGTACCATCTTCTTTTGTGGC : 1470
Fla.8924.3  : ----------------------------------------------------------------------------------------------------------------------------------- :    -
Floradade.  : ----------------------------------------------------------------------------------------------------------------------------------- :    -
LYC1410.3   : ----------------------------------------------------------------------------------------------------------------------------------- :    -
PI169588.3  : ----------------------------------------------------------------------------------------------------------------------------------- :    -
Brandywine  : TCGAATAACATTTATCTGCAGGAAAATATACAGACGACTAAAATGAGTAACATATTGACTACAGAGGAAGTCAGAGAACCTGAACCTATTGCACACACTGAATCAGATGTAGTACCATCTTCTTTTGTGGC : 1470
EA00990.3   : ----------------------------------------------------------------------------------------------------------------------------------- :    -
PI1303721.  : ----------------------------------------------------------------------------------------------------------------------------------- :    -
BGV006775.  : ----------------------------------------------------------------------------------------------------------------------------------- :    -
PAS014479.  : ----------------------------------------------------------------------------------------------------------------------------------- :    -

                   2760         *        2780         *        2800         *        2820         *        2840         *        2860         *        2880
ITAG.3      : --------------------------GTTCNNNNNNNNNNNNNNNNNNNNATCAACAACGTACTATTCGAACTGGTGATTTGTCCTTAGATTAGGAGAAGTTCAAGGAGGGCT-AATGAAACTAGACAACT : 1600
BGV006865.  : --------------------------GTTCNNNNNNNNNNNNNNNNNNNN-TCAACAACGTACTATTCGAACTGGTGATTTGTCCTTAGATTAGGAGAAGTTCAAGGAGGGCTAAATGAAACTAGACAGCT : 1600
BGV007931.  : --------------------------GTTCNNNNNNNNNNNNNNNNNNNN-TCAACAACGTACTATTCGAACTGGTGATTTGTCCTTAGATTAGGAGAAGTTCAAGGAGGGCTAAATGAAACTAGACAGCT : 1600
BGV007989.  : --------------------------GTTCNNNNNNNNNNNNNNNNNNNNATCAACAACGTACTATTCGAACTGGTGATTTGTCCTTAGATTAGGAGAAGTTCAAGGAGGGCT-AATGAAACTAGACAACT : 1600
M82.3       : AGAGTCGAATAACATTTATCTGCAGGAAAANNNNNNNNNNNNNNNNNNNNATCAACAACGTACTATTCGAACTGGTGATTTGTCCTTAGATTAGGAGAAGTTCAAGGAGGGCT-AATGAAACTAGACAACT : 1600
EA00371.3   : AGAGTCGAATAACATTTATCTGCAGGAAAANNNNNNNNNNNNNNNNNNNNATCAACAACGTACTATTCGAACTGGTGATTTGTCCTTAGATTAGGAGAAGTTCAAGGAGGGCT-AATGAAACTAGACAACT : 1600
Fla.8924.3  : --------------------------GTTCNNNNNNNNNNNNNNNNNNNNATCAACAACGTACTATTCGAACTGGTGATTTGTCCTTAGATTAGGAGAAGTTCAAGGAGGGCT-AATGAAACTAGACAACT : 1600
Floradade.  : --------------------------GTTCNNNNNNNNNNNNNNNNNNNNATCAACAACGTACTATTCGAACTGGTGATTTGTCCTTAGATTAGGAGAAGTTCAAGGAGGGCT-AATGAAACTAGACAACT : 1600
LYC1410.3   : --------------------------GTTCNNNNNNNNNNNNNNNNNNNNATCAACAACGTACTATTCGAACTGGTGATTTGTCCTTAGATTAGGAGAAGTTCAAGGAGGGCT-AATGAAACTAGACAACT : 1600
PI169588.3  : --------------------------GTTCNNNNNNNNNNNNNNNNNNNNATCAACAACGTACTATTCGAACTGGTGATTTGTCCTTAGATTAGGAGAAGTTCAAGGAGGGCT-AATGAAACTAGACAACT : 1600
Brandywine  : AGAGTCGAATAACATTTATCTGCAGGAAAANNNNNNNNNNNNNNNNNNNNATCAACAACGTACTATTCGAACTGGTGATTTGTCCTTAGATTAGGAGAAGTTCAAGGAGGGCT-AATGAAACTAGACAACT : 1600
EA00990.3   : --------------------------GTTCNNNNNNNNNNNNNNNNNNNN----------TACTATTCGAACTGGTGATTTGTCCTTAGATTAGGAGAAGTTCAAGGAGGGCT-AATGAAACTAGACAGCT : 1590
PI1303721.  : --------------------------GTTCNNNNNNNNNNNNNNNNNNNNATCAACAACGTACTATTCGAACTGGTGATTTGTCCTTAGATTAGGAGAAGTTCAAGGAGGGCT-AATGAAACTAGACAACT : 1600
BGV006775.  : --------------------------GTTCNNNNNNNNNNNNNNNNNNNN-TCAACAACGTACTATTCGAACTGGTGATTTGTCCTTAGATTAGGAGAAGTTCAAGGAGGGCTAAATGAAACTAGACAGCT : 1600
PAS014479.  : --------------------------GTTCNNNNNNNNNNNNNNNNNNNN----------TACTATTCGAACTGGTGATTTGTCCTTAGATTAGGAGAAGTTCAAGGAGGGCT-AATGAAACTAGACAGCT : 1590
                                        gttcNNNNNNNNNNNNNNNNNNNN tcaacaacgTACTATTCGAACTGGTGATTTGTCCTTAGATTAGGAGAAGTTCAAGGAGGGCT AATGAAACTAGACA CT

                    *        2900         *        2920         *        2940         *        2960         *        2980         *        3000         *
ITAG.3      : CAATTAATTAATGAATTAATGGTCTGGTTTAGTTAATGACCATGATGATACAACAGAGTGCTGTAGCTGCCAGTTGACTTTGTAATGAAAATTGGAAAACTGGCTAGGTGAATTTTTGATGCCAGAGGAGA : 1731
BGV006865.  : CAATTAATTAATGAATTAATGGTCTGGTTTAGTTAATGACCATGATGATACAACAGAGTGCTGTAGCTGCCAGTTGACTTTGTAATGAAAATTGGAAAACTGGCTAGGTGAATTTTTGATGCCAGAGGAGA : 1731
BGV007931.  : CAATTAATTAATGAATTAATGGTCTGGTTTAGTTAATGACCATGATGATACAACAGAGTGCTGTAGCTGCCAGTTGACTTTGTAATGAAAATTGGAAAACTGGCTAGGTGAATTTTTGATGCCAGAGGAGA : 1731
BGV007989.  : CAATTAATTAATGAATTAATGGTCTGGTTTAGTTAATGACCATGATGATACAACAGAGTGCTGTAGCTGCCAGTTGACTTTGTAATGAAAATTGGAAAACTGGCTAGGTGAATTTTTGATGCCAGAGGAGA : 1731
M82.3       : CAATTAATTAATGAATTAATGGTCTGGTTTAGTTAATGACCATGATGATACAACAGAGTGCTGTAGCTGCCAGTTGACTTTGTAATGAAAATTGGAAAACTGGCTAGGTGAATTTTTGATGCCAGAGGAGA : 1731
EA00371.3   : CAATTAATTAATGAATTAATGGTCTGGTTTAGTTAATGACCATGATGATACAACAGAGTGCTGTAGCTGCCAGTTGACTTTGTAATGAAAATTGGAAAACTGGCTAGGTGAATTTTTGATGCCAGAGGAGA : 1731
Fla.8924.3  : CAATTAATTAATGAATTAATGGTCTGGTTTAGTTAATGACCATGATGATACAACAGAGTGCTGTAGCTGCCAGTTGACTTTGTAATGAAAATTGGAAAACTGGCTAGGTGAATTTTTGATGCCAGAGGAGA : 1731
Floradade.  : CAATTAATTAATGAATTAATGGTCTGGTTTAGTTAATGACCATGATGATACAACAGAGTGCTGTAGCTGCCAGTTGACTTTGTAATGAAAATTGGAAAACTGGCTAGGTGAATTTTTGATGCCAGAGGAGA : 1731
LYC1410.3   : CAATTAATTAATGAATTAATGGTCTGGTTTAGTTAATGACCATGATGATACAACAGAGTGCTGTAGCTGCCAGTTGACTTTGTAATGAAAATTGGAAAACTGGCTAGGTGAATTTTTGATGCCAGAGGAGA : 1731
PI169588.3  : CAATTAATTAATGAATTAATGGTCTGGTTTAGTTAATGACCATGATGATACAACAGAGTGCTGTAGCTGCCAGTTGACTTTGTAATGAAAATTGGAAAACTGGCTAGGTGAATTTTTGATGCCAGAGGAGA : 1731
Brandywine  : CAATTAATTAATGAATTAATGGTCTGGTTTAGTTAATGACCATGATGATACAACAGAGTGCTGTAGCTGCCAGTTGACTTTGTAATGAAAATTGGAAAACTGGCTAGGTGAATTTTTGATGCCAGAGGAGA : 1731
EA00990.3   : CAATTAATTAATGAATTAATGGTCTGGTTTAGTTAATGACCATGATGATACAACAGAGTGCTGTAGCTGCCAGTTGACTTTGTAATGAAAATTGGAAAACTGGCTAGGTGAATTTTTGATGCCAGAGGAGA : 1721
PI1303721.  : CAATTAATTAATGAATTAATGGTCTGGTTTAGTTAATGACCATGATGATACAACAGAGTGCTGTAGCTGCCAGTTGACTTTGTAATGAAAATTGGAAAACTGGCTAGGTGAATTTTTGATGCCAGAGGAGA : 1731
BGV006775.  : CAATTAATTAATGAATTAATGGTCTGGTTTAGTTAATGACCATGATGATACAACAGAGTGCTGTAGCTGCCAGTTGACTTTGTAATGAAAATTGGAAAACTGGCTAGGTGAATTTTTGATGCCAGAGGAGA : 1731
PAS014479.  : CAATTAATTAATGAATTAATGGTCTGGTTTAGTTAATGACCATGATGATACAACAGAGTGCTGTAGCTGCCAGTTGACTTTGTAATGAAAATTGGAAAACTGGCTAGGTGAATTTTTGATGCCAGAGGAGA : 1721
              CAATTAATTAATGAATTAATGGTCTGGTTTAGTTAATGACCATGATGATACAACAGAGTGCTGTAGCTGCCAGTTGACTTTGTAATGAAAATTGGAAAACTGGCTAGGTGAATTTTTGATGCCAGAGGAGA

                 3020         *        3040         *        3060         *        3080         *        3100         *        3120         *        3140
ITAG.3      : AAGAAGCAAGAAGAAAGCATACAAACCGTTACCGCAGAATCTGTGCTTATCAATTGGACTACACATGGAGCAGTCCAAGTGCCACTAGTTGTTCTATGTGCAGTTTGTGATATTGAGTCG----------- : 1851
BGV006865.  : AAGAAGCAAGAAGAAAGCATACAAACCGTTACCGCAGAATCTGTGCTTATCAATTGGACTACACATGGAGCAGTCCAAGTGCCACTGGTTGTTCTATGTGCAGTTTGTGATATTGAGTCG----------- : 1851
BGV007931.  : AAGAAGCAAGAAGAAAGCATACAAACCGTTACCGCAGAATCTGTGCTTATCAATTGGACTACACATGGAGCAGTCCAAGTGCCACTGGTTGTTCTATGTGCAGTTTGTGATATTGAGTCG----------- : 1851
BGV007989.  : AAGAAGCAAGAAGAAAGCATACAAACCGTTACCGCAGAATCTGTGCTTATCAATTGGACTACACATGGAGCAGTCCAAGTGCCACTAGTTGTTCTATGTGCAGTTTGTGATATTGAGTCG----------- : 1851
M82.3       : AAGAAGCAAGAAGAAAGCATACAAACCGTTACCGCAGAATCTGTGCTTATCAATTGGACTACACATGGAGCAGTCCAAGTGCCACTAGTTGTTCTATGTGCAGTTTGTGATATTGAGTCG----------- : 1851
EA00371.3   : AAGAAGCAAGAAGAAAGCATACAAACCGTTACCGCAGAATCTGTGCTTATCAATTGGACTACACATGGAGCAGTCCAAGTGCCACTAGTTGTTCTATGTGCAGTTTGTGATATTGAGTCG----------- : 1851
Fla.8924.3  : AAGAAGCAAGAAGAAAGCATACAAACCGTTACCGCAGAATCTGTGCTTATCAATTGGACTACACATGGAGCAGTCCAAGTGCCACTAGTTGTTCTATGTGCAGTTTGTGATATTGAGTCG----------- : 1851
Floradade.  : AAGAAGCAAGAAGAAAGCATACAAACCGTTACCGCAGAATCTGTGCTTATCAATTGGACTACACATGGAGCAGTCCAAGTGCCACTAGTTGTTCTATGTGCAGTTTGTGATATTGAGTCG----------- : 1851
LYC1410.3   : AAGAAGCAAGAAGAAAGCATACAAACCGTTACCGCAGAATCTGTGCTTATCAATTGGACTACACATGGAGCAGTCCAAGTGCCACTAGTTGTTCTATGTGCAGTTTGTGATATTGAGTCG----------- : 1851
PI169588.3  : AAGAAGCAAGAAGAAAGCATACAAACCGTTACCGCAGAATCTGTGCTTATCAATTGGACTACACATGGAGCAGTCCAAGTGCCACTAGTTGTTCTATGTGCAGTTTGTGATATTGAGTCG----------- : 1851
Brandywine  : AAGAAGCAAGAAGAAAGCATACAAACCGTTACCGCAGAATCTGTGCTTATCAATTGGACTACACATGGAGCAGTCCAAGTGCCACTAGTTGTTCTATGTGCAGTTTGTGATATTGAGTCG----------- : 1851
EA00990.3   : AAGAAGCAAGAAGAAAGCATACAAACCGTTACCGCAGAATCTGTGCTT---AATTGGACTACACATGGAGCAGTCCAAGTGCCACTGGTTGTTCTATGTGCAGTTTGTGATATTGAGTCGGGCTATGATGA : 1849
PI1303721.  : AAGAAGCAAGAAGAAAGCATACAAACCGTTACCGCAGAATCTGTGCTTATCAATTGGACTACACATGGAGCAGTCCAAGTGCCACTAGTTGTTCTATGTGCAGTTTGTGATATTGAGTCG----------- : 1851
BGV006775.  : AAGAAGCAAGAAGAAAGCATACAAACCGTTACCGCAGAATCTGTGCTTATCAATTGGACTACACATGGAGCAGTCCAAGTGCCACTGGTTGTTCTATGTGCAGTTTGTGATATTGAGTCG----------- : 1851
PAS014479.  : AAGAAGCAAGAAGAAAGCATACAAACCGTTACCGCAGAATCTGTGCTT---AATTGGACTACACATGGAGCAGTCCAAGTGCCACTGGTTGTTCTATGTGCAGTTTGTGATATTGAGTCGGGCTATGATGA : 1849
              AAGAAGCAAGAAGAAAGCATACAAACCGTTACCGCAGAATCTGTGCTTatcAATTGGACTACACATGGAGCAGTCCAAGTGCCACT GTTGTTCTATGTGCAGTTTGTGATATTGAGTCG

                  *        3160         *        3180         *        3200         *        3220         *        3240         *        3260         *
ITAG.3      : --GGCTATTTTTGAGCGAATGCTAGCTAAACAATTGACAAGGATACAAGTTATATGTTTACCACTGATAGGGATTTTGTTTGCAATAAGTACATGATTTGCAAAAACTACTACTACACTATCTCTGATTGA : 1980
BGV006865.  : --GGCTATTTTTGAGCGAATGCTAGCTAAACAATTGACAAGGATACAAGTTATATGTTTACCACTGATAGGGATTTTGTTTGCAATAAGTACATGATTTGCAAAAACTACTACTACACTATCTCTGATTGA : 1980
BGV007931.  : --GGCTATTTTTGAGCGAATGCTAGCTAAACAATTGACAAGGATACAAGTTATATGTTTACCACTGATAGGGATTTTGTTTGCAATAAGTACATGATTTGCAAAAACTACTACTACACTATCTCTGATTGA : 1980
BGV007989.  : --GGCTATTTTTGAGCGAATGCTAGCTAAACAATTGACAAGGATACAAGTTATATGTTTACCACTGATAGGGATTTTGTTTGCAATAAGTACATGATTTGCAAAAACTACTACTACACTATCTCTGATTGA : 1980
M82.3       : --GGCTATTTTTGAGCGAATGCTAGCTAAACAATTGACAAGGATACAAGTTATATGTTTACCACTGATAGGGATTTTGTTTGCAATAAGTACATGATTTGCAAAAACTACTACTACACTATCTCTGATTGA : 1980
EA00371.3   : --GGCTATTTTTGAGCGAATGCTAGCTAAACAATTGACAAGGATACAAGTTATATGTTTACCACTGATAGGGATTTTGTTTGCAATAAGTACATGATTTGCAAAAACTACTACTACACTATCTCTGATTGA : 1980
Fla.8924.3  : --GGCTATTTTTGAGCGAATGCTAGCTAAACAATTGACAAGGATACAAGTTATATGTTTACCACTGATAGGGATTTTGTTTGCAATAAGTACATGATTTGCAAAAACTACTACTACACTATCTCTGATTGA : 1980
Floradade.  : --GGCTATTTTTGAGCGAATGCTAGCTAAACAATTGACAAGGATACAAGTTATATGTTTACCACTGATAGGGATTTTGTTTGCAATAAGTACATGATTTGCAAAAACTACTACTACACTATCTCTGATTGA : 1980
LYC1410.3   : --GGCTATTTTTGAGCGAATGCTAGCTAAACAATTGACAAGGATACAAGTTATATGTTTACCACTGATAGGGATTTTGTTTGCAATAAGTACATGATTTGCAAAAACTACTACTACACTATCTCTGATTGA : 1980
PI169588.3  : --GGCTATTTTTGAGCGAATGCTAGCTAAACAATTGACAAGGATACAAGTTATATGTTTACCACTGATAGGGATTTTGTTTGCAATAAGTACATGATTTGCAAAAACTACTACTACACTATCTCTGATTGA : 1980
Brandywine  : --GGCTATTTTTGAGCGAATGCTAGCTAAACAATTGACAAGGATACAAGTTATATGTTTACCACTGATAGGGATTTTGTTTGCAATAAGTACATGATTTGCAAAAACTACTACTACACTATCTCTGATTGA : 1980
EA00990.3   : GTGGCTATTTTTGAGCGAATGCTAGCTAAACAATTGACAAGGATACAAGTTATATGTTTACCACTGATAGGGATTTTGTTTGCAATAAGTACATGATTTGCAAAAACTACTACTACACTATCTCTGATTGA : 1980
PI1303721.  : --GGCTATTTTTGAGCGAATGCTAGCTAAACAATTGACAAGGATACAAGTTATATGTTTACCACTGATAGGGATTTTGTTTGCAATAAGTACATGATTTGCAAAAACTACTACTACACTATCTCTGATTGA : 1980
BGV006775.  : --GGCTATTTTTGAGCGAATGCTAGCTAAACAATTGACAAGGATACAAGTTATATGTTTACCACTGATAGGGATTTTGTTTGCAATAAGTACATGATTTGCAAAAACTACTACTACACTATCTCTGATTGA : 1980
PAS014479.  : GTGGCTATTTTTGAGCGAATGCTAGCTAAACAATTGACAAGGATACAAGTTATATGTTTACCACTGATAGGGATTTTGTTTGCAATAAGTACATGATTTGCAAAAACTACTACTACACTATCTCTGATTGA : 1980
                GGCTATTTTTGAGCGAATGCTAGCTAAACAATTGACAAGGATACAAGTTATATGTTTACCACTGATAGGGATTTTGTTTGCAATAAGTACATGATTTGCAAAAACTACTACTACACTATCTCTGATTGA

                3280         *        3300         *        3320         *        3340         *        3360         *        3380         *        3400
ITAG.3      : ACAATGTCTCGATTATCGCTAAGAAATCATGCTTGTATCCATCAGTTTGGAAATGATCCGAAGGAATGATCTTTGTATAGAAGTAGAAAGCAAAACAAGCAAGTCCAGGCCCAACCCAAAAGATCCAATGC : 2111
BGV006865.  : ACAATGTCTCGATTATCGCTAAGAAATCATGCTTGTATCCATCAGTTTGGAAATGATCCGAAGGAATGATCTTTGTATAGAAGTAGAAAGCAAAACAAGCAAGTCCAGGCCCAACCCAAAAGATCCAATGC : 2111
BGV007931.  : ACAATGTCTCGATTATCGCTAAGAAATCATGCTTGTATCCATCAGTTTGGAAATGATCCGAAGGAATGATCTTTGTATAGAAGTAGAAAGCAAAACAAGCAAGTCCAGGCCCAACCCAAAAGATCCAATGC : 2111
BGV007989.  : ACAATGTCTCGATTATCGCTAAGAAATCATGCTTGTATCCATCAGTTTGGAAATGATCCGAAGGAATGATCTTTGTATAGAAGTAGAAAGCAAAACAAGCAAGTCCAGGCCCAACCCAAAAGATCCAATGC : 2111
M82.3       : ACAATGTCTCGATTATCGCTAAGAAATCATGCTTGTATCCATCAGTTTGGAAATGATCCGAAGGAATGATCTTTGTATAGAAGTAGAAAGCAAAACAAGCAAGTCCAGGCCCAACCCAAAAGATCCAATGC : 2111
EA00371.3   : ACAATGTCTCGATTATCGCTAAGAAATCATGCTTGTATCCATCAGTTTGGAAATGATCCGAAGGAATGATCTTTGTATAGAAGTAGAAAGCAAAACAAGCAAGTCCAGGCCCAACCCAAAAGATCCAATGC : 2111
Fla.8924.3  : ACAATGTCTCGATTATCGCTAAGAAATCATGCTTGTATCCATCAGTTTGGAAATGATCCGAAGGAATGATCTTTGTATAGAAGTAGAAAGCAAAACAAGCAAGTCCAGGCCCAACCCAAAAGATCCAATGC : 2111
Floradade.  : ACAATGTCTCGATTATCGCTAAGAAATCATGCTTGTATCCATCAGTTTGGAAATGATCCGAAGGAATGATCTTTGTATAGAAGTAGAAAGCAAAACAAGCAAGTCCAGGCCCAACCCAAAAGATCCAATGC : 2111
LYC1410.3   : ACAATGTCTCGATTATCGCTAAGAAATCATGCTTGTATCCATCAGTTTGGAAATGATCCGAAGGAATGATCTTTGTATAGAAGTAGAAAGCAAAACAAGCAAGTCCAGGCCCAACCCAAAAGATCCAATGC : 2111
PI169588.3  : ACAATGTCTCGATTATCGCTAAGAAATCATGCTTGTATCCATCAGTTTGGAAATGATCCGAAGGAATGATCTTTGTATAGAAGTAGAAAGCAAAACAAGCAAGTCCAGGCCCAACCCAAAAGATCCAATGC : 2111
Brandywine  : ACAATGTCTCGATTATCGCTAAGAAATCATGCTTGTATCCATCAGTTTGGAAATGATCCGAAGGAATGATCTTTGTATAGAAGTAGAAAGCAAAACAAGCAAGTCCAGGCCCAACCCAAAAGATCCAATGC : 2111
EA00990.3   : ACAATGTCTCGATTATCGCTAAGAAATCATGCTTGTATCCATCAGTTTGGAAATGATCCGAAGGAATGATCTTTGTATAGAAGTAGAAAGCAAAACAAGCAAGTCCAGGCCCAACCCAAAAGATCCAATGC : 2111
PI1303721.  : ACAATGTCTCGATTATCGCTAAGAAATCATGCTTGTATCCATCAGTTTGGAAATGATCCGAAGGAATGATCTTTGTATAGAAGTAGAAAGCAAAACAAGCAAGTCCAGGCCCAACCCAAAAGATCCAATGC : 2111
BGV006775.  : ACAATGTCTCGATTATCGCTAAGAAATCATGCTTGTATCCATCAGTTTGGAAATGATCCGAAGGAATGATCTTTGTATAGAAGTAGAAAGCAAAACAAGCAAGTCCAGGCCCAACCCAAAAGATCCAATGC : 2111
PAS014479.  : ACAATGTCTCGATTATCGCTAAGAAATCATGCTTGTATCCATCAGTTTGGAAATGATCCGAAGGAATGATCTTTGTATAGAAGTAGAAAGCAAAACAAGCAAGTCCAGGCCCAACCCAAAAGATCCAATGC : 2111
              ACAATGTCTCGATTATCGCTAAGAAATCATGCTTGTATCCATCAGTTTGGAAATGATCCGAAGGAATGATCTTTGTATAGAAGTAGAAAGCAAAACAAGCAAGTCCAGGCCCAACCCAAAAGATCCAATGC

                 *        3420         *        3440         *        3460         *        3480         *        3500         *        3520         *        3
ITAG.3      : CCATTCCACAGGTGGCCTCCTCTAACAAAAGCAGCTCCAAAGCACCTAGCTGGATTCATTCCAGCTCCAGCATAGCCCTTTTTAGCAGTAACTGAAGTTGAAATAAACACAAGAATCCCTGCTAGAAGCCC : 2242
BGV006865.  : CCATTCCACAGGTGGCCTCCTCTAACAAAAGCAGCTCCAAAGCACCTAGCTGGATTCATTCCAGCTCCAGCATAGCCCTTTTTAGCAGTAACTGAAGTTGAAATAAACACGAGAATCCCTGCTAGAAGCCC : 2242
BGV007931.  : CCATTCCACAGGTGGCCTCCTCTAACAAAAGCAGCTCCAAAGCACCTAGCTGGATTCATTCCAGCTCCAGCATAGCCCTTTTTAGCAGTAACTGAAGTTGAAATAAACACGAGAATCCCTGCTAGAAGCCC : 2242
BGV007989.  : CCATTCCACAGGTGGCCTCCTCTAACAAAAGCAGCTCCAAAGCACCTAGCTGGATTCATTCCAGCTCCAGCATAGCCCTTTTTAGCAGTAACTGAAGTTGAAATAAACACAAGAATCCCTGCTAGAAGCCC : 2242
M82.3       : CCATTCCACAGGTGGCCTCCTCTAACAAAAGCAGCTCCAAAGCACCTAGCTGGATTCATTCCAGCTCCAGCATAGCCCTTTTTAGCAGTAACTGAAGTTGAAATAAACACAAGAATCCCTGCTAGAAGCCC : 2242
EA00371.3   : CCATTCCACAGGTGGCCTCCTCTAACAAAAGCAGCTCCAAAGCACCTAGCTGGATTCATTCCAGCTCCAGCATAGCCCTTTTTAGCAGTAACTGAAGTTGAAATAAACACAAGAATCCCTGCTAGAAGCCC : 2242
Fla.8924.3  : CCATTCCACAGGTGGCCTCCTCTAACAAAAGCAGCTCCAAAGCACCTAGCTGGATTCATTCCAGCTCCAGCATAGCCCTTTTTAGCAGTAACTGAAGTTGAAATAAACACAAGAATCCCTGCTAGAAGCCC : 2242
Floradade.  : CCATTCCACAGGTGGCCTCCTCTAACAAAAGCAGCTCCAAAGCACCTAGCTGGATTCATTCCAGCTCCAGCATAGCCCTTTTTAGCAGTAACTGAAGTTGAAATAAACACAAGAATCCCTGCTAGAAGCCC : 2242
LYC1410.3   : CCATTCCACAGGTGGCCTCCTCTAACAAAAGCAGCTCCAAAGCACCTAGCTGGATTCATTCCAGCTCCAGCATAGCCCTTTTTAGCAGTAACTGAAGTTGAAATAAACACAAGAATCCCTGCTAGAAGCCC : 2242
PI169588.3  : CCATTCCACAGGTGGCCTCCTCTAACAAAAGCAGCTCCAAAGCACCTAGCTGGATTCATTCCAGCTCCAGCATAGCCCTTTTTAGCAGTAACTGAAGTTGAAATAAACACAAGAATCCCTGCTAGAAGCCC : 2242
Brandywine  : CCATTCCACAGGTGGCCTCCTCTAACAAAAGCAGCTCCAAAGCACCTAGCTGGATTCATTCCAGCTCCAGCATAGCCCTTTTTAGCAGTAACTGAAGTTGAAATAAACACAAGAATCCCTGCTAGAAGCCC : 2242
EA00990.3   : CCATTCCACAGGTGGCCTCCTCTAACAAAAGCAGCTCCAAAGCACCTAGCTGGATTCATTCCAGCTCCAGCATAGCCCTTTTTAGCAGTAACTGAAGTTGAAATAAACACAAGAATCCCTGCTAGAAGCCC : 2242
PI1303721.  : CCATTCCACAGGTGGCCTCCTCTAACAAAAGCAGCTCCAAAGCACCTAGCTGGATTCATTCCAGCTCCAGCATAGCCCTTTTTAGCAGTAACTGAAGTTGAAATAAACACAAGAATCCCTGCTAGAAGCCC : 2242
BGV006775.  : CCATTCCACAGGTGGCCTCCTCTAACAAAAGCAGCTCCAAAGCACCTAGCTGGATTCATTCCAGCTCCAGCATAGCCCTTTTTAGCAGTAACTGAAGTTGAAATAAACACGAGAATCCCTGCTAGAAGCCC : 2242
PAS014479.  : CCATTCCACAGGTGGCCTCCTCTAACAAAAGCAGCTCCAAAGCACCTAGCTGGATTCATTCCAGCTCCAGCATAGCCCTTTTTAGCAGTAACTGAAGTTGAAATAAACACAAGAATCCCTGCTAGAAGCCC : 2242
              CCATTCCACAGGTGGCCTCCTCTAACAAAAGCAGCTCCAAAGCACCTAGCTGGATTCATTCCAGCTCCAGCATAGCCCTTTTTAGCAGTAACTGAAGTTGAAATAAACACaAGAATCCCTGCTAGAAGCCC

              540         *        3560         *        3580         *        3600         *        3620         *        3640         *        3660
ITAG.3      : AACAATAGACATGACAGTAATAAGCCCAAGTGCCTTGGACTGGCGATGATCATAGGCCATCCAAAGTGAACCAAAAAGCAAAGCAAATGTACACATAAACTCAAGCCAAAATGCTTGGGCTATCTCCAAGC : 2373
BGV006865.  : AACAATAGACATGACAGTAATAAGCCCAAGTGCCTTGGACTGGCGATGATCATAGGCCATCCAAAGTGAACCAAAAAGCAAAGCAAATGTACACATAAACTCAAGCCAAAATGCTTGGGCTATCTCCAAGC : 2373
BGV007931.  : AACAATAGACATGACAGTAATAAGCCCAAGTGCCTTGGACTGGCGATGATCATAGGCCATCCAAAGTGAACCAAAAAGCAAAGCAAATGTACACATAAACTCAAGCCAAAATGCTTGGGCTATCTCCAAGC : 2373
BGV007989.  : AACAATAGACATGACAGTAATAAGCCCAAGTGCCTTGGACTGGCGATGATCATAGGCCATCCAAAGTGAACCAAAAAGCAAAGCAAATGTACACATAAACTCAAGCCAAAATGCTTGGGCTATCTCCAAGC : 2373
M82.3       : AACAATAGACATGACAGTAATAAGCCCAAGTGCCTTGGACTGGCGATGATCATAGGCCATCCAAAGTGAACCAAAAAGCAAAGCAAATGTACACATAAACTCAAGCCAAAATGCTTGGGCTATCTCCAAGC : 2373
EA00371.3   : AACAATAGACATGACAGTAATAAGCCCAAGTGCCTTGGACTGGCGATGATCATAGGCCATCCAAAGTGAACCAAAAAGCAAAGCAAATGTACACATAAACTCAAGCCAAAATGCTTGGGCTATCTCCAAGC : 2373
```

```
Fla.8924.3 : AACAATAGACATGACAGTAATAAGCCCAAGTGCCTTGGACTGGCGATGATCATAGGCCATCCAAAGTGAACCAAAAAGCAAAGCAAATGTACACATAAACTCAAGCCAAAATGCTTGGGCTATCTCCAAGC : 2373
Floradade. : AACAATAGACATGACAGTAATAAGCCCAAGTGCCTTGGACTGGCGATGATCATAGGCCATCCAAAGTGAACCAAAAAGCAAAGCAAATGTACACATAAACTCAAGCCAAAATGCTTGGGCTATCTCCAAGC : 2373
LYC1410.3  : AACAATAGACATGACAGTAATAAGCCCAAGTGCCTTGGACTGGCGATGATCATAGGCCATCCAAAGTGAACCAAAAAGCAAAGCAAATGTACACATAAACTCAAGCCAAAATGCTTGGGCTATCTCCAAGC : 2373
PI169588.3 : AACAATAGACATGACAGTAATAAGCCCAAGTGCCTTGGACTGGCGATGATCATAGGCCATCCAAAGTGAACCAAAAAGCAAAGCAAATGTACACATAAACTCAAGCCAAAATGCTTGGGCTATCTCCAAGC : 2373
Brandywine : AACAATAGACATGACAGTAATAAGCCCAAGTGCCTTGGACTGGCGATGATCATAGGCCATCCAAAGTGAACCAAAAAGCAAAGCAAATGTACACATAAACTCAAGCCAAAATGCTTGGGCTATCTCCAAGC : 2373
EA00990.3  : AACAATAGACATGACAGTAATAAGCCCAAGTGCCTTGGACTGGCGATGATCATAGGCCATCCAAAGTGAACCAAAAAGCAAAGCAAATGTACACATAAACTCAAGCCAAAATGCTTGGGCTATCTCCAAGC : 2373
PI1303721. : AACAATAGACATGACAGTAATAAGCCCAAGTGCCTTGGACTGGCGATGATCATAGGCCATCCAAAGTGAACCAAAAAGCAAAGCAAATGTACACATAAACTCAAGCCAAAATGCTTGGGCTATCTCCAAGC : 2373
BGV006775. : AACAATAGACATGACAGTAATAAGCCCAAGTGCCTTGGACTGGCGATGATCATAGGCCATCCAAAGTGAACCAAAAAGCAAAGCAAATGTACACATAAACTCAAGCCAAAATGCTTGGGCTATCTCCAAGC : 2373
PAS014479. : AACAATAGACATGACAGTAATAAGCCCAAGTGCCTTGGACTGGCGATGATCATAGGCCATCCAAAGTGAACCAAAAAGCAAAGCAAATGTACACATAAACTCAAGCCAAAATGCTTGGGCTATCTCCAAGC : 2373
             AACAATAGACATGACAGTAATAAGCCCAAGTGCCTTGGACTGGCGATGATCATAGGCCATCCAAAGTGAACCAAAAAGCAAAGCAAATGTACACATAAACTCAAGCCAAAATGCTTGGGCTATCTCCAAGC

               *      3680         *      3700         *      3720         *      3740         *      3760         *      3780         *      380
ITAG.3     : CCACAATTTCAGGCCCATTGAGCCCAGGCGTAACTACCGTAACGGTACAACCACCAAGAGAGAATCTTTGTTCAATGGTACTACTCAACACAGCTTTGAGTGCTAGTGCACCTAAAATAGCACCAACACAT : 2504
BGV006865. : CCACAATTTCAGGCCCATTGAGCCCAGGCGTAACTACCGTAACGGTACAACCACCAAGAGAGAATCTTTGTTCAATGGTACTACTCAACACAGCTTTGAGTGCTAGTGCACCTAAAATAGCACCAACACAT : 2504
BGV007931. : CCACAATTTCAGGCCCATTGAGCCCAGGCGTAACTACCGTAACGGTACAACCACCAAGAGAGAATCTTTGTTCAATGGTACTACTCAACACAGCTTTGAGTGCTAGTGCACCTAAAATAGCACCAACACAT : 2504
BGV007989. : CCACAATTTCAGGCCCATTGAGCCCAGGCGTAACTACCGTAACGGTACAACCACCAAGAGAGAATCTTTGTTCAATGGTACTACTCAACACAGCTTTGAGTGCTAGTGCACCTAAAATAGCACCAACACAT : 2504
M82.3      : CCACAATTTCAGGCCCATTGAGCCCAGGCGTAACTACCGTAACGGTACAACCACCAAGAGAGAATCTTTGTTCAATGGTACTACTCAACACAGCTTTGAGTGCTAGTGCACCTAAAATAGCACCAACACAT : 2504
EA00371.3  : CCACAATTTCAGGCCCATTGAGCCCAGGCGTAACTACCGTAACGGTACAACCACCAAGAGAGAATCTTTGTTCAATGGTACTACTCAACACAGCTTTGAGTGCTAGTGCACCTAAAATAGCACCAACACAT : 2504
Fla.8924.3 : CCACAATTTCAGGCCCATTGAGCCCAGGCGTAACTACCGTAACGGTACAACCACCAAGAGAGAATCTTTGTTCAATGGTACTACTCAACACAGCTTTGAGTGCTAGTGCACCTAAAATAGCACCAACACAT : 2504
Floradade. : CCACAATTTCAGGCCCATTGAGCCCAGGCGTAACTACCGTAACGGTACAACCACCAAGAGAGAATCTTTGTTCAATGGTACTACTCAACACAGCTTTGAGTGCTAGTGCACCTAAAATAGCACCAACACAT : 2504
LYC1410.3  : CCACAATTTCAGGCCCATTGAGCCCAGGCGTAACTACCGTAACGGTACAACCACCAAGAGAGAATCTTTGTTCAATGGTACTACTCAACACAGCTTTGAGTGCTAGTGCACCTAAAATAGCACCAACACAT : 2504
PI169588.3 : CCACAATTTCAGGCCCATTGAGCCCAGGCGTAACTACCGTAACGGTACAACCACCAAGAGAGAATCTTTGTTCAATGGTACTACTCAACACAGCTTTGAGTGCTAGTGCACCTAAAATAGCACCAACACAT : 2504
Brandywine : CCACAATTTCAGGCCCATTGAGCCCAGGCGTAACTACCGTAACGGTACAACCACCAAGAGAGAATCTTTGTTCAATGGTACTACTCAACACAGCTTTGAGTGCTAGTGCACCTAAAATAGCACCAACACAT : 2504
EA00990.3  : CCACAATTTCAGGCCCATTGAGCCCAGGCGTAACTACCGTAACGGTACAACCACCAAGAGAGAATCTTTGTTCAATGGTACTACTCAACACAGCTTTGAGTGCTAGTGCACCTAAAATAGCACCAACACAT : 2504
PI1303721. : CCACAATTTCAGGCCCATTGAGCCCAGGCGTAACTACCGTAACGGTACAACCACCAAGAGAGAATCTTTGTTCAATGGTACTACTCAACACAGCTTTGAGTGCTAGTGCACCTAAAATAGCACCAACACAT : 2504
BGV006775. : CCACAATTTCAGGCCCATTGAGCCCAGGCGTAACTACCGTAACGGTACAACCACCAAGAGAGAATCTTTGTTCAATGGTACTACTCAACACAGCTTTGAGTGCTAGTGCACCTAAAATAGCACCAACACAT : 2504
PAS014479. : CCACAATTTCAGGCCCATTGAGCCCAGGCGTAACTACCGTAACGGTACAACCACCAAGAGAGAATCTTTGTTCAATGGTACTACTCAACACAGCTTTGAGTGCTAGTGCACCTAAAATAGCACCAACACAT : 2504
             CCACAATTTCAGGCCCATTGAGCCCAGGCGTAACTACCGTAACGGTACAACCACCAAGAGAGAATCTTTGTTCAATGGTACTACTCAACACAGCTTTGAGTGCTAGTGCACCTAAAATAGCACCAACACAT

             0         *      3820         *      3840         *      3860         *      3880         *      3900         *      3920         *
ITAG.3     : TGTGCCACTATGTAAATTATGGCTCGTGACATTGATATTAGTCCCACTAGTGCTGAGGAGAAGGTAATTACAGGGCTAATGTGGCCACCAGACACAGGAAAAACAGCAAGGATTAGAATGGTGAGTGTAAT : 2635
BGV006865. : TGTGCCACTATGTAAATTATGGCTCGTGACATTGATATTAGTCCCACTAGTGCTGAGGAGAAGGTAATTACAGGGCTAATGTGGCCACCAGACACAGGAAAAACAGCAAGGATTAGAATGGTGAGTGTAAT : 2635
BGV007931. : TGTGCCACTATGTAAATTATGGCTCGTGACATTGATATTAGTCCCACTAGTGCTGAGGAGAAGGTAATTACAGGGCTAATGTGGCCACCAGACACAGGAAAAACAGCAAGGATTAGAATGGTGAGTGTAAT : 2635
BGV007989. : TGTGCCACTATGTAAATTATGGCTCGTGACATTGATATTAGTCCCACTAGTGCTGAGGAGAAGGTAATTACAGGGCTAATGTGGCCACCAGACACAGGAAAAACAGCAAGGATTAGAATGGTGAGTGTAAT : 2635
M82.3      : TGTGCCACTATGTAAATTATGGCTCGTGACATTGATATTAGTCCCACTAGTGCTGAGGAGAAGGTAATTACAGGGCTAATGTGGCCACCAGACACAGGAAAAACAGCAAGGATTAGAATGGTGAGTGTAAT : 2635
EA00371.3  : TGTGCCACTATGTAAATTATGGCTCGTGACATTGATATTAGTCCCACTAGTGCTGAGGAGAAGGTAATTACAGGGCTAATGTGGCCACCAGACACAGGAAAAACAGCAAGGATTAGAATGGTGAGTGTAAT : 2635
Fla.8924.3 : TGTGCCACTATGTAAATTATGGCTCGTGACATTGATATTAGTCCCACTAGTGCTGAGGAGAAGGTAATTACAGGGCTAATGTGGCCACCAGACACAGGAAAAACAGCAAGGATTAGAATGGTGAGTGTAAT : 2635
Floradade. : TGTGCCACTATGTAAATTATGGCTCGTGACATTGATATTAGTCCCACTAGTGCTGAGGAGAAGGTAATTACAGGGCTAATGTGGCCACCAGACACAGGAAAAACAGCAAGGATTAGAATGGTGAGTGTAAT : 2635
LYC1410.3  : TGTGCCACTATGTAAATTATGGCTCGTGACATTGATATTAGTCCCACTAGTGCTGAGGAGAAGGTAATTACAGGGCTAATGTGGCCACCAGACACAGGAAAAACAGCAAGGATTAGAATGGTGAGTGTAAT : 2635
PI169588.3 : TGTGCCACTATGTAAATTATGGCTCGTGACATTGATATTAGTCCCACTAGTGCTGAGGAGAAGGTAATTACAGGGCTAATGTGGCCACCAGACACAGGAAAAACAGCAAGGATTAGAATGGTGAGTGTAAT : 2635
Brandywine : TGTGCCACTATGTAAATTATGGCTCGTGACATTGATATTAGTCCCACTAGTGCTGAGGAGAAGGTAATTACAGGGCTAATGTGGCCACCAGACACAGGAAAAACAGCAAGGATTAGAATGGTGAGTGTAAT : 2635
EA00990.3  : TGTGCCACTATGTAAATTATGGCTCGTGACATTGATATTAGTCCCACTAGTGCTGAGGAGAAGGTAATTACAGGGCTAATGTGGCCACCAGACACAGGAAAAACAGCAAGGATTAGAATGGTGAGTGTAAT : 2635
PI1303721. : TGTGCCACTATGTAAATTATGGCTCGTGACATTGATATTAGTCCCACTAGTGCTGAGGAGAAGGTAATTACAGGGCTAATGTGGCCACCAGACACAGGAAAAACAGCAAGGATTAGAATGGTGAGTGTAAT : 2635
BGV006775. : TGTGCCACTATGTAAATTATGGCTCGTGACATTGATATTAGTCCCACTAGTGCTGAGGAGAAGGTAATTACAGGGCTAATGTGGCCACCAGACACAGGAAAAACAGCAAGGATTAGAATGGTGAGTGTAAT : 2635
PAS014479. : TGTGCCACTATGTAAATTATGGCTCGTGACATTGATATTAGTCCCACTAGTGCTGAGGAGAAGGTAATTACAGGACTAATGTGGCCACCAGACACAGGAAAAACAGCAAGGATTAGAATGGTGAGTGTAAT : 2635
             TGTGCCACTATGTAAATTATGGCTCGTGACATTGATATTAGTCCCACTAGTGCTGAGGAGAAGGTAATTACAGGgCTAATGTGGCCACCAGACACAGGAAAAACAGCAAGGATTAGAATGGTGAGTGTAAT

                     3940         *      3960         *      3980         *      4000         *      4020         *      4040         *      4060
ITAG.3     : TGCTATGAGAATTGACATAATCAAATTTGGCATTTTGACATCACTTTCTAGGGTGGAAATCACTACGGTGTCCAACATAAAAACAAGAACCGCAGAGCCTAAAAGCTCTCCAACCGATGCTCGCCACACCT : 2766
BGV006865. : TGCTATGAGAATTGACATAATCAAATTTGGCATTTTGACATCACTTTCTAGGGTGGAAATCACTACGGTGTCCAACATAAAAACAAGAACCGCAGAGCCTAAAAGCTCTCCAACCGATGCTCGCCACACCT : 2766
BGV007931. : TGCTATGAGAATTGACATAATCAAATTTGGCATTTTGACATCACTTTCTAGGGTGGAAATCACTACGGTGTCCAACATAAAAACAAGAACCGCAGAGCCTAAAAGCTCTCCAACCGATGCTCGCCACACCT : 2766
BGV007989. : TGCTATGAGAATTGACATAATCAAATTTGGCATTTTGACATCACTTTCTAGGGTGGAAATCACTACGGTGTCCAACATAAAAACAAGAACCGCAGAGCCTAAAAGCTCTCCAACCGATGCTCGCCACACCT : 2766
M82.3      : TGCTATGAGAATTGACATAATCAAATTTGGCATTTTGACATCACTTTCTAGGGTGGAAATCACTACGGTGTCCAACATAAAAACAAGAACCGCAGAGCCTAAAAGCTCTCCAACCGATGCTCGCCACACCT : 2766
EA00371.3  : TGCTATGAGAATTGACATAATCAAATTTGGCATTTTGACATCACTTTCTAGGGTGGAAATCACTACGGTGTCCAACATAAAAACAAGAACCGCAGAGCCTAAAAGCTCTCCAACCGATGCTCGCCACACCT : 2766
Fla.8924.3 : TGCTATGAGAATTGACATAATCAAATTTGGCATTTTGACATCACTTTCTAGGGTGGAAATCACTACGGTGTCCAACATAAAAACAAGAACCGCAGAGCCTAAAAGCTCTCCAACCGATGCTCGCCACACCT : 2766
Floradade. : TGCTATGAGAATTGACATAATCAAATTTGGCATTTTGACATCACTTTCTAGGGTGGAAATCACTACGGTGTCCAACATAAAAACAAGAACCGCAGAGCCTAAAAGCTCTCCAACCGATGCTCGCCACACCT : 2766
LYC1410.3  : TGCTATGAGAATTGACATAATCAAATTTGGCATTTTGACATCACTTTCTAGGGTGGAAATCACTACGGTGTCCAACATAAAAACAAGAACCGCAGAGCCTAAAAGCTCTCCAACCGATGCTCGCCACACCT : 2766
PI169588.3 : TGCTATGAGAATTGACATAATCAAATTTGGCATTTTGACATCACTTTCTAGGGTGGAAATCACTACGGTGTCCAACATAAAAACAAGAACCGCAGAGCCTAAAAGCTCTCCAACCGATGCTCGCCACACCT : 2766
Brandywine : TGCTATGAGAATTGACATAATCAAATTTGGCATTTTGACATCACTTTCTAGGGTGGAAATCACTACGGTGTCCAACATAAAAACAAGAACCGCAGAGCCTAAAAGCTCTCCAACCGATGCTCGCCACACCT : 2766
EA00990.3  : TGCTATGAGAATTGACATAATCAAATTTGGCATTTTGACATCACTTTCTAGGGTGGAAATCACTACCGTGTCCAACATAAAAACAAGAACCGCAGAGCCTAAAAGCTCTCCAACCGATGCTCGCCACACCT : 2766
PI1303721. : TGCTATGAGAATTGACATAATCAAATTTGGCATTTTGACATCACTTTCTAGGGTGGAAATCACTACGGTGTCCAACATAAAAACAAGAACCGCAGAGCCTAAAAGCTCTCCAACCGATGCTCGCCACACCT : 2766
BGV006775. : TGCTATGAGAATTGACATAATCAAATTTGGCATTTTGACATCACTTTCTAGGGTGGAAATCACTACGGTGTCCAACATAAAAACAAGAACCGCAGAGCCTAAAAGCTCTCCAACCGATGCTCGCCACACCT : 2766
PAS014479. : TGCTATGAGAATTGACATAATCAAATTTGGCATTTTGACATCACTTTCTAGGGTGGAAATCACTACCGTGTCCAACATAAAAACAAGAACCGCAGAGCCTAAAAGCTCTCCAACCGATGCTCGCCACACCT : 2766
             TGCTATGAGAATTGACATAATCAAATTTGGCATTTTGACATCACTTTCTAGGGTGGAAATCACTACgGTGTCCAACATAAAAACAAGAACCGCAGAGCCTAAAAGCTCTCCAACCGATGCTCGCCACACCT

                   *      4080         *      4100         *      4120         *      4140         *      4160         *      4180         *
ITAG.3     : ACATAAAAAAATAGTCGAATTCTATTGCTTAGTCAATACAGTCGTATAGAAGATGAGTAAGAGATGAAATATACTAACGTCCAAAGAGAAGAAGTCAGGTAAGCCCAGCCTTTCAGCCATTGTGAGACAAGT : 2897
BGV006865. : ACATAAAAAAATAGTCGAATTCTATTGCTTAGTCAATACAGTCGTATAGAAGATGAGTAAGAGATGAAATATACTAACGTCCAAAGAGAAGAAGTCAGGTAAGCCCAGCCTTTCAGCCATTGTGAGACAAGT : 2897
BGV007931. : ACATAAAAAAATAGTCGAATTCTATTGCTTAGTCAATACAGTCGTATAGAAGATGAGTAAGAGATGAAATATACTAACGTCCAAAGAGAAGAAGTCAGGTAAGCCCAGCCTTTCAGCCATTGTGAGACAAGT : 2897
BGV007989. : ACATAAAAAAATAGTCGAATTCTATTGCTTAGTCAATACAGTCGTATAGAAGATGAGTAAGAGATGAAATATACTAACGTCCAAAGAGAAGAAGTCAGGTAAGCCCAGCCTTTCAGCCATTGTGAGACAAGT : 2897
M82.3      : ACATAAAAAAATAGTCGAATTCTATTGCTTAGTCAATACAGTCGTATAGAAGATGAGTAAGAGATGAAATATACTAACGTCCAAAGAGAAGAAGTCAGGTAAGCCCAGCCTTTCAGCCATTGTGAGACAAGT : 2897
EA00371.3  : ACATAAAAAAATAGTCGAATTCTATTGCTTAGTCAATACAGTCGTATAGAAGATGAGTAAGAGATGAAATATACTAACGTCCAAAGAGAAGAAGTCAGGTAAGCCCAGCCTTTCAGCCATTGTGAGACAAGT : 2897
Fla.8924.3 : ACATAAAAAAATAGTCGAATTCTATTGCTTAGTCAATACAGTCGTATAGAAGATGAGTAAGAGATGAAATATACTAACGTCCAAAGAGAAGAAGTCAGGTAAGCCCAGCCTTTCAGCCATTGTGAGACAAGT : 2897
Floradade. : ACATAAAAAAATAGTCGAATTCTATTGCTTAGTCAATACAGTCGTATAGAAGATGAGTAAGAGATGAAATATACTAACGTCCAAAGAGAAGAAGTCAGGTAAGCCCAGCCTTTCAGCCATTGTGAGACAAGT : 2897
LYC1410.3  : ACATAAAAAAATAGTCGAATTCTATTGCTTAGTCAATACAGTCGTATAGAAGATGAGTAAGAGATGAAATATACTAACGTCCAAAGAGAAGAAGTCAGGTAAGCCCAGCCTTTCAGCCATTGTGAGACAAGT : 2897
PI169588.3 : ACATAAAAAAATAGTCGAATTCTATTGCTTAGTCAATACAGTCGTATAGAAGATGAGTAAGAGATGAAATATACTAACGTCCAAAGAGAAGAAGTCAGGTAAGCCCAGCCTTTCAGCCATTGTGAGACAAGT : 2897
Brandywine : ACATAAAAAAATAGTCGAATTCTATTGCTTAGTCAATACAGTCGTATAGAAGATGAGTAAGAGATGAAATATACTAACGTCCAAAGAGAAGAAGTCAGGTAAGCCCAGCCTTTCAGCCATTGTGAGACAAGT : 2897
EA00990.3  : ACATAAAAAAATAGTCGAATTCTG-TGCTTAGTCAATACAGTCGTATAGAAGATGAGTAAGAGATGAAATATACTAACGTCCAAAGAGAAGAAGTCAGGTAAGCCCAGCCTTTCAGCCATTGTGAGACAAGT : 2896
PI1303721. : ACATAAAAAAATAGTCGAATTCTATTGCTTAGTCAATACAGTCGTATAGAAGATGAGTAAGAGATGAAATATACTAACGTCCAAAGAGAAGAAGTCAGGTAAGCCCAGCCTTTCAGCCATTGTGAGACAAGT : 2897
BGV006775. : ACATAAAAAAATAGTCGAATTCTATTGCTTAGTCAATACAGTCGTATAGAAGATGAGTAAGAGATGAAATATACTAACGTCCAAAGAGAAGAAGTCAGGTAAGCCCAGCCTTTCAGCCATTGTGAGACAAGT : 2897
PAS014479. : ACATAAAAAAATAGTCGAATTCTG-TGCTTAGTCAATACAGTCGTATAGAAGATGAGTAAGAGATGAAATATACTAACGTCCAAAGAGAAGAAGTCAGGTAAGCCCAGCCTTTCAGCCATTGTGAGACAAGT : 2896
             ACATAAAAAAATAGTCGAATTCTatTGCTTAGTCAATACAGTCGTATAGAAGATGAGTAAGAGATGAAATATACTAACGTCCAAAGAGAAGAAGTCAGGTAAGCCCAGCCTTTCAGCCATTGTGAGACAAGT

                 4200         *      4220         *      4240         *      4260         *      4280         *      4300         *
ITAG.3     : ATACTTCTTCTTTTCATCATCTGAGCTGTTAAAAAAAGAAGCCAAACACCATACATATATGTAAGAACAAATTGAAAAGATATCAAACCAAAAAAAGATAAAATATCTGAGGCTTATCATAAA- : 3020
BGV006865. : ATACTTCTTCTTTTCATCATCTGAGCTGTTAAAAAAAGAAGCCAAACACCATACATATATGTAAGAACAAATTGAAAAGGTATCAAACCAAAAAAAGATAAAATATCTGAGGCTTATCATAAA- : 3020
BGV007931. : ATACTTCTTCTTTTCATCATCTGAGCTGTTAAAAAAAGAAGCCAAACACCATACATATATGTAAGAACAAATTGAAAAGGTATCAAACCAAAAAAAGATAAAATATCTGAGGCTTATCATAAA- : 3020
BGV007989. : ATACTTCTTCTTTTCATCATCTGAGCTGTTAAAAAAAGAAGCCAAACACCATACATATATGTAAGAACAAATTGAAAAGATATCAAACCAAAAAAAGATAAAATATCTGAGGCTTATCATAAA- : 3020
M82.3      : ATACTTCTTCTTTTCATCATCTGAGCTGTTAAAAAAAGAAGCCAAACACCATACATATATGTAAGAACAAATTGAAAAGATATCAAACCAAAAAAAGATAAAATATCTGAGGCTTATCATAAA- : 3020
EA00371.3  : ATACTTCTTCTTTTCATCATCTGAGCTGTTAAAAAAAGAAGCCAAACACCATACATATATGTAAGAACAAATTGAAAAGATATCAAACCAAAAAAAGATAAAATATCTGAGGCTTATCATAAA- : 3020
Fla.8924.3 : ATACTTCTTCTTTTCATCATCTGAGCTGTTAAAAAAAGAAGCCAAACACCATACATATATGTAAGAACAAATTGAAAAGATATCAAACCAAAAAAAGATAAAATATCTGAGGCTTATCATAAA- : 3020
Floradade. : ATACTTCTTCTTTTCATCATCTGAGCTGTTAAAAAAAGAAGCCAAACACCATACATATATGTAAGAACAAATTGAAAAGATATCAAACCAAAAAAAGATAAAATATCTGAGGCTTATCATAAA- : 3020
LYC1410.3  : ATACTTCTTCTTTTCATCATCTGAGCTGTTAAAAAAAGAAGCCAAACACCATACATATATGTAAGAACAAATTGAAAAGATATCAAACCAAAAAAAGATAAAATATCTGAGGCTTATCATAAA- : 3020
PI169588.3 : ATACTTCTTCTTTTCATCATCTGAGCTGTTAAAAAAAGAAGCCAAACACCATACATATATGTAAGAACAAATTGAAAAGATATCAAACCAAAAAAAGATAAAATATCTGAGGCTTATCATAAA- : 3020
Brandywine : ATACTTCTTCTTTTCATCATCTGAGCTGTTAAAAAAAGAAGCCAAACACCATACATATATGTAAGAACAAATTGAAAAGATATCAAACCAAAAAAAGATAAAATATCTGAGGCTTATCATAAA- : 3020
EA00990.3  : ATACTTCTTCTTTTCATCATCTGAGCTGTTAAAAAAAGAAGCCAAACACCATACATATATGTAAGAACAAATTGAAAAGATATCAAACCAAAAAAAGATAAAATATCTGAGGCTTATCATAAAT : 3020
PI1303721. : ATACTTCTTCTTTTCATCATCTGAGCTGTTAAAAAAAGAAGCCAAACACCATACATATATGTAAGAACAAATTGAAAAGATATCAAACCAAAAAAAGATAAAATATCTGAGGCTTATCATAAA- : 3020
BGV006775. : ATACTTCTTCTTTTCATCATCTGAGCTGTTAAAAAAAGAAGCCAAACACCATACATATATGTAAGAACAAATTGAAAAGGTATCAAACCAAAAAAAGATAAAATATCTGAGGCTTATCATAAA- : 3020
PAS014479. : ATACTTCTTCTTTTCATCATCTGAGCTGTTAAAAAAAGAAGCCAAACACCATACATATATGTAAGAACAAATTGAAAAGATATCAAACCAAAAAAAGATAAAATATCTGAGGCTTATCATAAAT : 3020
             ATACTTCTTCTTTTCATCATCTGAGCTGTTAAAAAAAGAAGCCAAACACCATACATATATGTAAGAACAAATTGAAAAGaTATCAAACCAAAAAAAGATAAAATATCTGAGGCTTATCATAAA
```

Solyc02g087170

```
                          *      20         *      40         *      60         *      80         *     100         *     120         *
ITAG.4     : ---------------caattgtgaatgacatgcattttcctataaatagctcgtttgagaacctgggaaaagcagatttcgaacagctaccttaatttccttcaaatagacaaagatggccttccaaaggt :  116
BGV006865. : ----------------aattgtgaatgacatgcattttcctataaatagctcgtttgagaacctgggaaaagcagatttcgaacagctaccttaatttccttcaaatagacaaagatggccttccaaaggt :  115
BGV007931. : ---------------caattgtgaatgacatgcattttcctataaatagctcgtttgagaacctgggaaaagcagatttcgaacagctaccttaatttccttcaaatagacaaagatggccttccaaaggt :  116
BGV007989. : actccattaattaaacaattgtgaatgacatgcattttcctataaatagctcgtttgagaacctgggaaaagcagatttcgaacagctaccttaatttccttcaaatagacaaagatggccttccaaaggt :  131
M82.4      : ---------------caattgtgaatgacatgcattttcctataaatagctcgtttgagaacctgggaaaagcagatttcgaacagctaccttaatttccttcaaatagacaaagatggccttccaaaggt :  116
EA00371.4  : actccattaattaaacaattgtgaatgacatgcattttcctataaatagctcgtttgagaacctgggaaaagcagatttcgaacagctaccttaatttccttcaaatagacaaagatggccttccaaaggt :  131
Fla.8924.4 : ---------------caattgtgaatgacatgcattttcctataaatagctcgtttgagaacctgggaaaagcagatttcgaacagctaccttaatttccttcaaatagacaaagatggccttccaaaggt :  116
Floradade. : ---------------caattgtgaatgacatgcattttcctataaatagctcgtttgagaacctgggaaaagcagatttcgaacagctaccttaatttccttcaaatagacaaagatggccttccaaaggt :  116
LYC1410.4  : ---------------caattgtgaatgacatgcattttcctataaatagctcgtttgagaacctgggaaaagcagatttcgaacagctaccttaatttccttcaaatagacaaagatggccttccaaaggt :  116
PI169588.4 : ---------------caattgtgaatgacatgcattttcctataaatagctcgtttgagaacctgggaaaagcagatttcgaacagctaccttaatttccttcaaatagacaaagatggccttccaaaggt :  116
Brandywine : ---------------caattgtgaatgacatgcattttcctataaatagctcgtttgagaacctgggaaaagcagatttcgaacagctaccttaatttccttcaaatagacaaagatggccttccaaaggt :  116
EA00990.4  : actccattaattaaacaattgtgaatgacatgcattttcctataaatagctcgtttgagaacctgggaaaagcagatttcgaacagctaccttaatttccttcaaatagacaaagatggccttccaaaggt :  131
PI303721.4 : ---------------caattgtgaatgacatgcattttcctataaatagctcgtttgagaacctgggaaaagcagatttcgaacagctaccttaatttccttcaaatagacaaagatggccttccaaaggt :  116
BGV006775. : ---------------caattgtgaatgacatgcattttcctataaatagctcgtttgagaacctgggaaaagcagatttcgaacagctaccttaatttccttcaaatagacaaagatggccttccaaaggt :  116
PAS014479. : actccattaattaaacaattgtgaatgacatgcattttcctataaatagctcgtttgagaacctgggaaaagcagatttcgaacagctaccttaatttccttcaaatagacaaagatggccttccaaaggt :  131
                            cAATTGTGAATGACATGCATTTTCCTATAAATAGCTCGTTTGAGAACCTGGGAAAAGCAGATTTCGAACAGCTACCTTAATTTCCTTCAAATAGACAAAGATGGCCTTCCAAAGGT

                 140         *      160         *      180         *      200         *      220         *      240         *      260
ITAG.4     : ttgtttttgtagcagtaattatgcttgtcttcatggctagtttatctgtgagagcacaacaagtagaagcacctactccaactccaggggggttcttcagacaactctaatgcatcaccaaatggactcgtt :  247
BGV006865. : ttgtttttgtagcagtaattatgcttgtcttcatggctagtttatctgtgagagcacaacaagtagaagcacctactccaactccaggggggttcttcagacaactctaatgcatcaccaaatggactcgtt :  246
BGV007931. : ttgtttttgtagcagtaattatgcttgtcttcatggctagtttatctgtgagagcacaacaagtagaagcacctactccaactccaggggggttcttcagacaactctaatgcatcaccaaatggactcgtt :  247
BGV007989. : ttgtttttgtagcagtaattatgcttgtcttcatggctagtttagctgtgagagcacaacaagtagaagcacctactccaactccaggggggttcttcagacaactctaatgcatcaccaaatggactcgtt :  262
M82.4      : ttgtttttgtagcagtaattatgcttgtcttcatggctagtttatctgtgagagcacaacaagtagaagcacctactccaactccaggggggttcttcagacaactctaatgcatcaccaaatggactcgtt :  247
EA00371.4  : ttgtttttgtagcagtaattatgcttgtcttcatggctagtttagctgtgagagcacaacaagtagaagcacctactccaactccaggggggttcttcagacaactctaatgcatcaccaaatggactcgtt :  262
Fla.8924.4 : ttgtttttgtagcagtaattatgcttgtcttcatggctagtttatctgtgagagcacaacaagtagaagcacctactccaactccaggggggttcttcagacaactctaatgcatcaccaaatggactcgtt :  247
Floradade. : ttgtttttgtagcagtaattatgcttgtcttcatggctagtttatctgtgagagcacaacaagtagaagcacctactccaactccaggggggttcttcagacaactctaatgcatcaccaaatggactcgtt :  247
LYC1410.4  : ttgtttttgtagcagtaattatgcttgtcttcatggctagtttatctgtgagagcacaacaagtagaagcacctactccaactccaggggggttcttcagacaactctaatgcatcaccaaatggactcgtt :  247
PI169588.4 : ttgtttttgtagcagtaattatgcttgtcttcatggctagtttatctgtgagagcacaacaagtagaagcacctactccaactccaggggggttcttcagacaactctaatgcatcaccaaatggactcgtt :  247
```

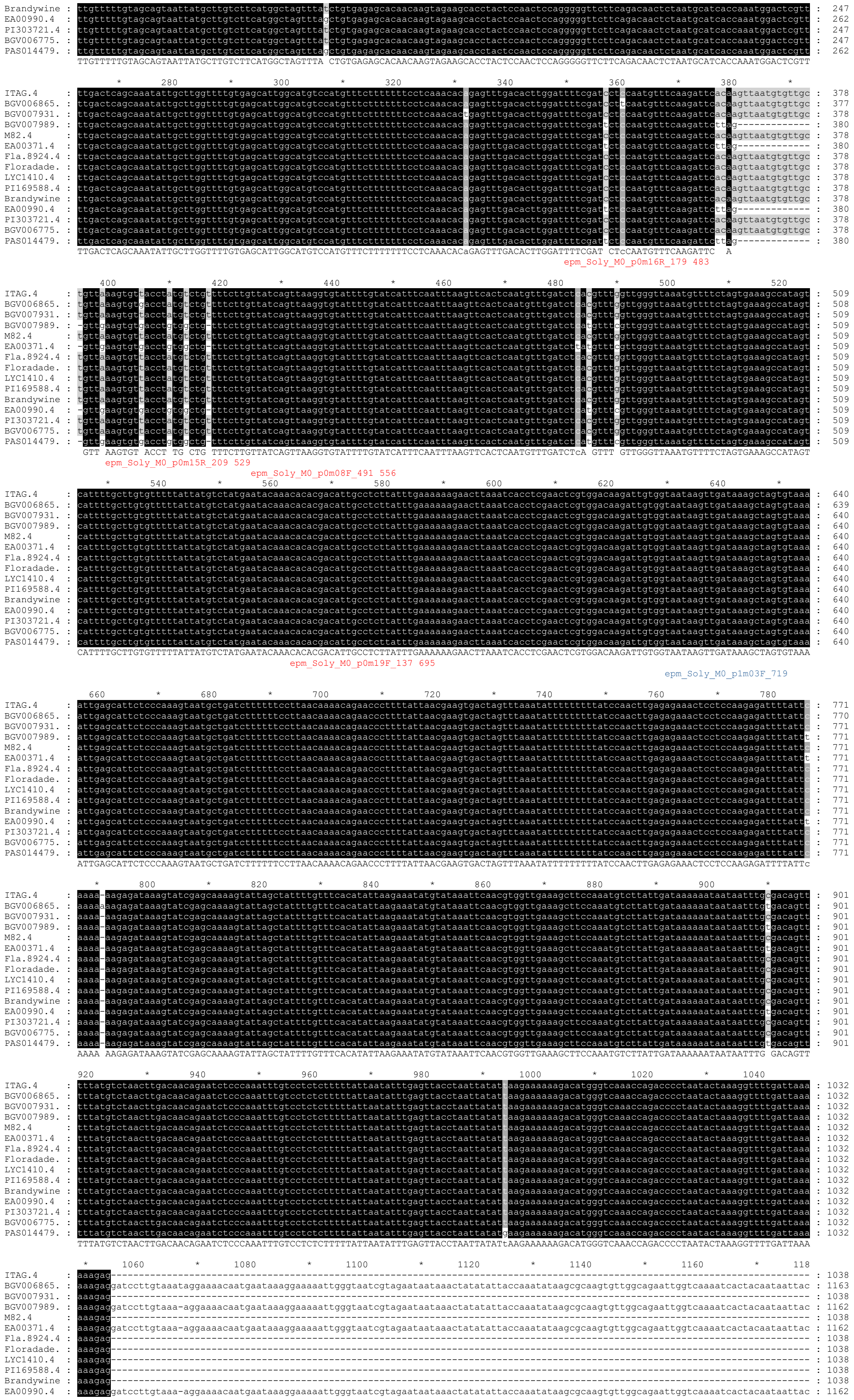

epm_Soly_M0_p0m16R_179 483
epm_Soly_M0_p0m15R_209 529
epm_Soly_M0_p0m08F_491 556
epm_Soly_M0_p0m19F_137 695
epm_Soly_M0_p1m03F_719

```
PI303721.4 : aaagag------------------------------------------------------------------------------------------------------------------------- : 1038
BGV006775. : aaagag------------------------------------------------------------------------------------------------------------------------- : 1038
PAS014479. : aaagaggatccttgtaaataggaaaacaatgaataaaggaaaaattgggtaatcgtagaataataaactatatattaccaaatataagcgcaagtgttggcagaattggtcaaaatcactacaataattac : 1163
             AAAGAG
                                                                     epm_Soly_M0_p0m07F_137 1243

             0        *       1200       *       1220       *       1240       *       1260       *       1280       *       1300       *
ITAG.4     : --------------------------------------------------------------------aacagaaacaactgccatagatcgaccaacttggaaattggaagacggagaagtgatagtaga : 1101
BGV006865. : tggaattttgggtttaacagagttgttaattagtaactaatctgaactacagttgggacatcaaatcaaacagaaacaactgccatagatcgaccaacttggaaattggaagacggagaagtgatagtaga : 1294
BGV007931. : --------------------------------------------------------------------aacagaaacaactgccatagatcgaccaacttggaaattggaagacggagaagtgatagtaga : 1101
BGV007989. : tggaattttgggtttaacagagttgttaattagtaactaatctgaactacagttgggacatcaaatcaaacagaaacaactgccatagatcgaccaacttggaaattggaagacggagaagtgatagtaga : 1293
M82.4      : --------------------------------------------------------------------aacagaaacaactgccatagatcgaccaacttggaaattggaagacggagaagtgatagtaga : 1101
EA00371.4  : tggaattttgggtttaacagagttgttaattagtaactaatctgaactacagttgtgacatcaaatcaaacagaaacaactgccatagatcgaccaacttggaaattggaagacggagaagtgatagtaga : 1293
Fla.8924.4 : --------------------------------------------------------------------aacagaaacaactgccatagatcgaccaacttggaaattggaagacggagaagtgatagtaga : 1101
Floradade. : --------------------------------------------------------------------aacagaaacaactgccatagatcgaccaacttggaaattggaagacggagaagtgatagtaga : 1101
LYC1410.4  : --------------------------------------------------------------------aacagaaacaactgccatagatcgaccaacttggaaattggaagacggagaagtgatagtaga : 1101
PI169588.4 : --------------------------------------------------------------------aacagaaacaactgccatagatcgaccaacttggaaattggaagacggagaagtgatagtaga : 1101
Brandywine : --------------------------------------------------------------------aacagaaacaactgccatagatcgaccaacttggaaattggaagacggagaagtgatagtaga : 1101
EA00990.4  : tggaattttgggtttaacagagttgttaattagtaactaatctgaactacagttgggacatcaaatcaaacagaaacaactgccatagatcgaccaacttggaaattggaagacggagaagtgatagtaga : 1293
PI303721.4 : --------------------------------------------------------------------aacagaaacaactgccatagatcgaccaacttggaaattggaagacggagaagtgatagtaga : 1101
BGV006775. : --------------------------------------------------------------------aacagaaacaactgccatagatcgaccaacttggaaattggaagacggagaagtgatagtaga : 1101
PAS014479. : tggaattttgggtttaacagagttgttaattagtaactaatctgaactacagttgggacatcaaatcaaacagaaacaactgccatagatcgaccaacttggaaattggaagacggagaagtgatagtaga : 1294
                                                                                 AACAGAAACAACTGCCATAGATCGACCAACTTGGAAATTGGAAGACGGAGAAGTGATAGTAGA
                                                                                                                     epm_Soly_M0_p0m16R_179
                   1320       *       1340       *       1360       *       1380       *       1400       *       1420       *       1440
ITAG.4     : tacgcaggtcatgaggaccgatgggatcttgggagccggaaagagagatggccggagatccgccattaatactgctcggaataagcagaaatggctcagcagaaaagagaaatggctggtggttcttgggg : 1232
BGV006865. : tacgcaggtcatgaggaccgatgggatcttgggagccggaaagagagatggccggagatccgccattaatactgctcggaataagcagaaatggctcagcagaaaagagaaatggctggtggttcttgggg : 1425
BGV007931. : tacgcaggtcatgaggaccgatgggatcttgggagccggaaagagagatggccggagatccgccattaatactgctcggaataagcagaaatggctcagcagaaaagagaaatggctggtggttcttgggg : 1232
BGV007989. : tacgcaggtcatgaggaccgatgggatcttgggagccggaaagagagatggccggagatccgccattaatactgctcggaataagcagaaatggctcagcagaaaagagaaatggctggtggttcttgggg : 1424
M82.4      : tacgcaggtcatgaggaccgatgggatcttgggagccggaaagagagatggccggagatccgccattaatactgctcggaataagcagaaatggctcagcagaaaagagaaatggctggtggttcttgggg : 1232
EA00371.4  : tacgcaggtcatgaggaccgatgggatcttgggagccggaaagagagatggccggagatccgccattaatactgctcggaataagcagaaatggctcagcagaaaagagaaatggctggtggttcttgggg : 1424
Fla.8924.4 : tacgcaggtcatgaggaccgatgggatcttgggagccggaaagagagatggccggagatccgccattaatactgctcggaataagcagaaatggctcagcagaaaagagaaatggctggtggttcttgggg : 1232
Floradade. : tacgcaggtcatgaggaccgatgggatcttgggagccggaaagagagatggccggagatccgccattaatactgctcggaataagcagaaatggctcagcagaaaagagaaatggctggtggttcttgggg : 1232
LYC1410.4  : tacgcaggtcatgaggaccgatgggatcttgggagccggaaagagagatggccggagatccgccattaatactgctcggaataagcagaaatggctcagcagaaaagagaaatggctggtggttcttgggg : 1232
PI169588.4 : tacgcaggtcatgaggaccgatgggatcttgggagccggaaagagagatggccggagatccgccattaatactgctcggaataagcagaaatggctcagcagaaaagagaaatggctggtggttcttgggg : 1232
Brandywine : tacgcaggtcatgaggaccgatgggatcttgggagccggaaagagagatggccggagatccgccattaatactgctcggaataagcagaaatggctcagcagaaaagagaaatggctggtggttcttgggg : 1232
EA00990.4  : tacgcaggtcatgaggaccgatgggatcttgggagccggaaagagagatggccggagatccgccattaatactgctcggaataagcagaaatggctcagcagaaaagagaaatggctggtggttcttgggg : 1424
PI303721.4 : tacgcaggtcatgaggaccgatgggatcttgggagccggaaagagagatggccggagatccgccattaatactgctcggaataagcagaaatggctcagcagaaaagagaaatggctggtggttcttgggg : 1232
BGV006775. : tacgcaggtcatgaggaccgatgggatcttgggagccggaaagagagatggccggagatccgccattaatactgctcggaataagcagaaatggctcagcagaaaagagaaatggctggtggttcttgggg : 1232
PAS014479. : tacgcaggtcatgaggaccgatgggatcttgggagccggaaagagagatggccggagatccgccattaatactgctcggaataagcagaaatggctcagcagaaaagagaaatggctggtggttcttgggg : 1425
             TACGCAGGTCATGAGGACCGATGGGATCTTGGGAGCCGGAAAGAGAGATGGCCGGAGATCCGCCATTAATACTGCTCGGAATAAGCAGAAATGGCTCAGCAGAAAAGAGAAATGGCTGGTGGTTCTTGGGG
                                                                                                                  epm_Soly_M0_p0m16R_179 1561
                  *       1460       *       1480       *       1500       *       1520       *       1540       *       1560       *
ITAG.4     : ttgtacttcatgcagtctacatgctaagtatcttcgatatttacttcaaaacccctattgttcacggaatggaccctgtcccacctcgtatgagtgccccagctaagcgccttgttcttctggttggtgcg : 1363
BGV006865. : ttgtacttcatgcagtctacatgctaagtatcttcgatatttacttcaaaacccctattgttcacggaatgga---------------------------------------------------------- : 1498
BGV007931. : ttgtacttcatgcagtctacatgctaagtatcttcgatatttacttcaaaacccctattgttcacggaatggaccctgtcccacctcgtatgagtgccccagctaagcgccttgttcttctggttggtgcg : 1363
BGV007989. : ttgtacttcatgcagtctacatgctaagtatcttcgatatttacttcaaaacccctattgttcacggaatggac--------------------------------------------------------- : 1498
M82.4      : ttgtacttcatgcagtctacatgctaagtatcttcgatatttacttcaaaacccctattgttcacggaatggaccctgtcccacctcgtatgagtgccccagctaagcgccttgttcttctggttggtgcg : 1363
EA00371.4  : ttgtacttcatgcagtctacatgctaagtatcttcgatatttacttcaaaacccctattgttcacggaatggac--------------------------------------------------------- : 1498
Fla.8924.4 : ttgtacttcatgcagtctacatgctaagtatcttcgatatttacttcaaaacccctattgttcacggaatggaccctgtcccacctcgtatgagtgccccagctaagcgccttgttcttctggttggtgcg : 1363
Floradade. : ttgtacttcatgcagtctacatgctaagtatcttcgatatttacttcaaaacccctattgttcacggaatggaccctgtcccacctcgtatgagtgccccagctaagcgccttgttcttctggttggtgcg : 1363
LYC1410.4  : ttgtacttcatgcagtctacatgctaagtatcttcgatatttacttcaaaacccctattgttcacggaatggaccctgtcccacctcgtatgagtgccccagctaagcgccttgttcttctggttggtgcg : 1363
PI169588.4 : ttgtacttcatgcagtctacatgctaagtatcttcgatatttacttcaaaacccctattgttcacggaatggaccctgtcccacctcgtatgagtgccccagctaagcgccttgttcttctggttggtgcg : 1363
Brandywine : ttgtacttcatgcagtctacatgctaagtatcttcgatatttacttcaaaacccctattgttcacggaatggaccctgtcccacctcgtatgagtgccccagctaagcgccttgttcttctggttggtgcg : 1363
EA00990.4  : ttgtacttcatgcagtctacatgctaagtatcttcgatatttacttcaaaacccctattgttcacggaatggac--------------------------------------------------------- : 1498
PI303721.4 : ttgtacttcatgcagtctacatgctaagtatcttcgatatttacttcaaaacccctattgttcacggaatggaccctgtcccacctcgtatgagtgccccagctaagcgccttgttcttctggttggtgcg : 1363
BGV006775. : ttgtacttcatgcagtctacatgctaagtatcttcgatatttacttcaaaacccctattgttcacggaatggaccctgtcccacctcgtatgagtgccccagctaagcgccttgttcttctggttggtgcg : 1363
PAS014479. : ttgtacttcatgcagtctacatgctaagtatcttcgatatttacttcaaaacccctattgttcacggaatgga---------------------------------------------------------- : 1498
             TTGTACTTCATGCAGTCTACATGCTAAGTATCTTCGATATTTACTTCAAAACCCCTATTGTTCACGGAATGGAc

                 1580       *       1600       *       1620       *       1640       *       1660       *       1680       *       1700
ITAG.4     : ttgtacttgcacacaacttgctcgatgatttgacacaatgaaatattttcagctcatgtttttatttttcttgcattgaattggccagtgggtgattttttttttcttttggttgataaattgcagccga : 1494
BGV006865. : ----------------------------------------------------------------------------------------------------------------------------------- :    -
BGV007931. : ttgtacttgcacacaacttgctcgatgatttgacacaatgaaatattttcagctcatgtttttatttttcttgcattgaattggccagtgggtgattttttttttcttttggttgataaattgcagccga : 1494
BGV007989. : ----------------------------------------------------------------------------------------------------------------------------------- :    -
M82.4      : ttgtacttgcacacaacttgctcgatgatttgacacaatgaaatattttcagctcatgtttttatttttcttgcattgaattggccagtgggtgattttttttttcttttggttgataaattgcagccga : 1494
EA00371.4  : ----------------------------------------------------------------------------------------------------------------------------------- :    -
Fla.8924.4 : ttgtacttgcacacaacttgctcgatgatttgacacaatgaaatattttcagctcatgtttttatttttcttgcattgaattggccagtgggtgattttttttttcttttggttgataaattgcagccga : 1494
Floradade. : ttgtacttgcacacaacttgctcgatgatttgacacaatgaaatattttcagctcatgtttttatttttcttgcattgaattggccagtgggtgattttttttttcttttggttgataaattgcagccga : 1494
LYC1410.4  : ttgtacttgcacacaacttgctcgatgatttgacacaatgaaatattttcagctcatgtttttatttttcttgcattgaattggccagtgggtgattttttttttcttttggttgataaattgcagccga : 1494
PI169588.4 : ttgtacttgcacacaacttgctcgatgatttgacacaatgaaatattttcagctcatgtttttatttttcttgcattgaattggccagtgggtgattttttttttcttttggttgataaattgcagccga : 1494
Brandywine : ttgtacttgcacacaacttgctcgatgatttgacacaatgaaatattttcagctcatgtttttatttttcttgcattgaattggccagtgggtgattttttttttcttttggttgataaattgcagccga : 1494
EA00990.4  : ----------------------------------------------------------------------------------------------------------------------------------- :    -
PI303721.4 : ttgtacttgcacacaacttgctcgatgatttgacacaatgaaatattttcagctcatgtttttatttttcttgcattgaattggccagtgggtgattttttttttcttttggttgataaattgcagccga : 1494
BGV006775. : ttgtacttgcacacaacttgctcgatgatttgacacaatgaaatattttcagctcatgtttttatttttcttgcattgaattggccagtgggtgattttttttttcttttggttgataaattgcagccga : 1494
PAS014479. : ----------------------------------------------------------------------------------------------------------------------------------- :    -

                                                                                                                     epm_Soly_M0_p0m15R_209
                    *       1720       *       1740       *       1760       *       1780       *       1800       *       1820       *
ITAG.4     : tggttt                    tgttttctgattggtaataggtccgaaataaaggaagctggatggaaattggcaacagcattagccatttcggaattatgagtgctcaagttgtgtttgtcctta : 1625
BGV006865. : ----ccnnnnnnnnnnnnnnnnnnnntgttttctgattggtaataggtccgaaataaaggaagctggatggaaattggcaacagcattagccatttcggaattatgagtgctcaagttgtgtttgtcctta : 1625
BGV007931. : tggtttnnnnnnnnnnnnnnnnnnnntgttttctgattggtaataggtccgaaataaaggaagctggatggaaattggcaacagcattagccatttcggaattatgagtgctcaagttgtgtttgtcctta : 1625
BGV007989. : ----ccnnnnnnnnnnnnnnnnnnnntgttttctgattggtaataggtccgaaataaaggaagctggatggaaattggcaacagcattagccatttcggaattatgagtgctcaagttgtgtttgtcctta : 1625
M82.4      : tggtttnnnnnnnnnnnnnnnnnnnntgttttctgattggtaataggtccgaaataaaggaagctggatggaaattggcaacagcattagccatttcggaattatgagtgctcaagttgtgtttgtcctta : 1625
EA00371.4  : ----ccnnnnnnnnnnnnnnnnnnnntgttttctgattggtaataggtccgaaataaaggaagctggatggaaattggcaacagcattagccatttcggaattatgagtgctcaagttgtgtttgtcctta : 1625
Fla.8924.4 : tggtttnnnnnnnnnnnnnnnnnnnntgttttctgattggtaataggtccgaaataaaggaagctggatggaaattggcaacagcattagccatttcggaattatgagtgctcaagttgtgtttgtcctta : 1625
Floradade. : tggtttnnnnnnnnnnnnnnnnnnnntgttttctgattggtaataggtccgaaataaaggaagctggatggaaattggcaacagcattagccatttcggaattatgagtgctcaagttgtgtttgtcctta : 1625
LYC1410.4  : tggtttnnnnnnnnnnnnnnnnnnnntgttttctgattggtaataggtccgaaataaaggaagctggatggaaattggcaacagcattagccatttcggaattatgagtgctcaagttgtgtttgtcctta : 1625
PI169588.4 : tggtttnnnnnnnnnnnnnnnnnnnntgttttctgattggtaataggtccgaaataaaggaagctggatggaaattggcaacagcattagccatttcggaattatgagtgctcaagttgtgtttgtcctta : 1625
Brandywine : tggtttnnnnnnnnnnnnnnnnnnnntgttttctgattggtaataggtccgaaataaaggaagctggatggaaattggcaacagcattagccatttcggaattatgagtgctcaagttgtgtttgtcctta : 1625
EA00990.4  : ----ccnnnnnnnnnnnnnnnnnnnntgttttctgattggtaataggtccgaaataaaggaagctggatggaaattggcaacagcattagccatttcggaattatgagtgctcaagttgtgtttgtcctta : 1625
PI303721.4 : tggtttnnnnnnnnnnnnnnnnnnnntgttttctgattggtaataggtccgaaataaaggaagctggatggaaattggcaacagcattagccatttcggaattatgagtgctcaagttgtgtttgtcctta : 1625
BGV006775. : tggtttnnnnnnnnnnnnnnnnnnnntgttttctgattggtaataggtccgaaataaaggaagctggatggaaattggcaacagcattagccatttcggaattatgagtgctcaagttgtgtttgtcctta : 1625
PAS014479. : ----ccnnnnnnnnnnnnnnnnnnnntgttttctgattggtaataggtccgaaataaaggaagctggatggaaattggcaacagcattagccatttcggaattatgagtgctcaagttgtgtttgtcctta : 1625
                   NNNNNNNNNNNNNNNNNNNNTGTTTTCTGATTGGTAATAGGTCCGAAATAAAGGAAGCTGGATGGAAATTGGCAACAGCATTAGCCATTTCGGAATTATGAGTGCTCAAGTTGTGTTTGTCCTTA


               1840       *       1860       *       1880       *       1900       *       1920       *       1940       *       1960
ITAG.4     : tgcttttgctgttacaaatgtattcacgaaagacatccaagttggatcagctcagcagttttctcgtaaaaaaatgtgattcgtttgaagcttcatcctttttgagatcccacatttctgggttgcaatt : 1756
BGV006865. : tgcttttgctgttacaaatgtattcacgaaagacatccaagttggatcagctcagcagttttctcgtaaaaaaatgtgattcatttgaagcttcatcctttttgagatcccacatttctgggttgcaatt : 1756
BGV007931. : tgcttttgctgttacaaatgtattcacgaaagacatccaagttggatcagctcagcagttttctcgtaaaaaaatgtgattcgtttgaagcttcatcctttttgagatcccacatttctgggttgcaatt : 1756
BGV007989. : tgcttttgctgttacaaatgtattcacgaaagacatccaagttggatcagctcagcagttttctcgtaaaaaaatgtgattcatttgaagcttcatcctttttgagatcccacatttctgggttgcaatt : 1756
M82.4      : tgcttttgctgttacaaatgtattcacgaaagacatccaagttggatcagctcagcagttttctcgtaaaaaaatgtgattcgtttgaagcttcatcctttttgagatcccacatttctgggttgcaatt : 1756
EA00371.4  : tgcttttgctgttacaaatgtattcacgaaagacatccaagttggatcagctcagcagttttctcgtaaaaaaatgtgattcatttgaagcttcatcctttttgagatcccacatttctgggttgcaatt : 1756
Fla.8924.4 : tgcttttgctgttacaaatgtattcacgaaagacatccaagttggatcagctcagcagttttctcgtaaaaaaatgtgattcgtttgaagcttcatcctttttgagatcccacatttctgggttgcaatt : 1756
Floradade. : tgcttttgctgttacaaatgtattcacgaaagacatccaagttggatcagctcagcagttttctcgtaaaaaaatgtgattcgtttgaagcttcatcctttttgagatcccacatttctgggttgcaatt : 1756
LYC1410.4  : tgcttttgctgttacaaatgtattcacgaaagacatccaagttggatcagctcagcagttttctcgtaaaaaaatgtgattcgtttgaagcttcatcctttttgagatcccacatttctgggttgcaatt : 1756
PI169588.4 : tgcttttgctgttacaaatgtattcacgaaagacatccaagttggatcagctcagcagttttctcgtaaaaaaatgtgattcgtttgaagcttcatcctttttgagatcccacatttctgggttgcaatt : 1756
Brandywine : tgcttttgctgttacaaatgtattcacgaaagacatccaagttggatcagctcagcagttttctcgtaaaaaaatgtgattcgtttgaagcttcatcctttttgagatcccacatttctgggttgcaatt : 1756
EA00990.4  : tgcttttgctgttacaaatgtattcacgaaagacatccaagttggatcagctcagcagttttctcgtaaaaaaatgtgattcatttgaagcttcatcctttttgagatcccacatttctgggttgcaatt : 1756
PI303721.4 : tgcttttgctgttacaaatgtattcacgaaagacatccaagttggatcagctcagcagttttctcgtaaaaaaatgtgattcgtttgaagcttcatcctttttgagatcccacatttctgggttgcaatt : 1756
BGV006775. : tgcttttgctgttacaaatgtattcacgaaagacatccaagttggatcagctcagcagttttctcgtaaaaaaatgtgattcgtttgaagcttcatcctttttgagatcccacatttctgggttgcaatt : 1756
PAS014479. : tgcttttgctgttacaaatgtattcacgaaagacatccaagttggatcagctcagcagttttctcgtaaaaaaatgtgattcatttgaagcttcatcctttttgagatcccacatttctgggttgcaatt : 1756
             TGCTTTTGCTGTTACAAATGTATTCACGAAAGACATCCAAGTTGGATCAGCTCAGCAGTTTTCTCGTAAAAAAATGTGATTC TTTGAAGCTTCATCCTTTTTGAGATCCCACATTTCTGGGTTGCAATT

                  *       1980       *       2000       *       2020       *       2040       *       2060       *       2080       *
ITAG.4     : ttggtagagtagcaattagttctcacacaaatcgttgtattgggcgatgctatatgaagtaaatcattcctttggatatcccatatttagggtggcaaagcgtatagtttcttgccaaaccagagtggat : 1887
BGV006865. : ttggtagagtagcaattagttctcacacaaatcgttgtattgagcgatgctatatgaagtaaatcattcctttggatatcccatatttagggtggcaaagcaaatagtttcttgccaaaccagagtggat : 1887
BGV007931. : ttggtagagtagcaattagttctcacacaaatcgttgtattgggcgatgctatatgaagtaaatcattcctttggatatcccatatttagggtggcaaagcgtatagtttcttgccaaaccagagtggat : 1887
BGV007989. : ttggtagagtagcaattagttctcacacaaatcgttgtattgagcgatgctatatgaagtaaatcattcctttggatatcccatatttagggtggcaaagcatatagtttcttgccaaaccagagtggat : 1887
M82.4      : ttggtagagtagcaattagttctcacacaaatcgttgtattgggcgatgctatatgaagtaaatcattcctttggatatcccatatttagggtggcaaagcgtatagtttcttgccaaaccagagtggat : 1887
EA00371.4  : ttggtagagtagcaattagttctcacacaaatcgttgtattgagcgatgctatatgaagtaaatcattcctttggatatcccatatttagggtggcaaagcatatagtttcttgccaaaccagagtggat : 1887
Fla.8924.4 : ttggtagagtagcaattagttctcacacaaatcgttgtattgggcgatgctatatgaagtaaatcattcctttggatatcccatatttagggtggcaaagcgtatagtttcttgccaaaccagagtggat : 1887
Floradade. : ttggtagagtagcaattagttctcacacaaatcgttgtattgggcgatgctatatgaagtaaatcattcctttggatatcccatatttagggtggcaaagcgtatagtttcttgccaaaccagagtggat : 1887
LYC1410.4  : ttggtagagtagcaattagttctcacacaaatcgttgtattgggcgatgctatatgaagtaaatcattcctttggatatcccatatttagggtggcaaagcgtatagtttcttgccaaaccagagtggat : 1887
PI169588.4 : ttggtagagtagcaattagttctcacacaaatcgttgtattgggcgatgctatatgaagtaaatcattcctttggatatcccatatttagggtggcaaagcgtatagtttcttgccaaaccagagtggat : 1887
Brandywine : ttggtagagtagcaattagttctcacacaaatcgttgtattgggcgatgctatatgaagtaaatcattcctttggatatcccatatttagggtggcaaagcgtatagtttcttgccaaaccagagtggat : 1887
EA00990.4  : ttggtagagtagcaattagttctcacacaaatcgttgtattgagcgatgctatatgaagtaaatcattcctttggatatcccatatttagggtggcaaagcatatagtttcttgccaaaccagagtggat : 1887
PI303721.4 : ttggtagagtagcaattagttctcacacaaatcgttgtattgggcgatgctatatgaagtaaatcattcctttggatatcccatatttagggtggcaaagcgtatagtttcttgccaaaccagagtggat : 1887
BGV006775. : ttggtagagtagcaattagttctcacacaaatcgttgtattgagcgatgctatatgaagtaaatcattcctttggatatcccatatttagggtggcaaagcgtatagtttcttgccaaaccagagtggat : 1887
PAS014479. : ttggtagagtagcaattagttctcacacaaatcgttgtattgagcgatgctatatgaagtaaatcattcctttggatatcccatatttagggtggcaaagcgtatagtttcttgccaaaccagagtggat : 1887
```

```
              TTGGTAGAGTAGCAATTAGTTCTCACACAAATCGTTGTATTG GCGATGCTATATGAAGTAAATCATTCCTTTGGATATCCCATATTTTAGGGTGGCAAAGC tATAGTTTCTTGCCAAACCAGAGTGGAT

              2100         *       2120         *       2140         *       2160         *       2180         *       2200         *       2220
ITAG.4      : agttgtatcgaatatatgatgatgagatgaaatggtgttgagctgctttgttggatgtattaaaattggttgcttcttgttactttgaatttcgagatgtgaaatcatttcagcagcgaatgcataaaatt : 2018
BGV006865.  : agttgtatcgaatatatgatgatgagatgaaatggtgttgagctgctttgttggatgtattaaaattggttgcttcttgttactttgaatttcgagatgtgaaatcatttcagcagcgaatgcataaaatt : 2018
BGV007931.  : agttgtatcgaatatatgatgatgagatgaaatggtgttgagctgctttgttggatgtattaaaattggttgcttcttgttactttgaatttcgagatgtgaaatcatttcagcagcgaatgcataaaatt : 2018
BGV007989.  : agttgtatcgaatatatgatgatgagatgaaatggtgttgagctgctttgttggatgtattaaaattggttgcttcttgttactttgaatttcgagatgtgaaatcatttcagcagcgaatgcataaaatt : 2018
M82.4       : agttgtatcgaatatatgatgatgagatgaaatggtgttgagctgctttgttggatgtattaaaattggttgcttcttgttactttgaatttcgagatgtgaaatcatttcagcagcgaatgcataaaatt : 2018
EA00371.4   : agttgtatcgaatatatgatgatgagatgaaatggtgttgagctgctttgttggatgtattaaaattggttgcttcttgttactttgaatttcgagatgtgaaatcatttcagcagcgaatgcataaaatt : 2018
Fla.8924.4  : agttgtatcgaatatatgatgatgagatgaaatggtgttgagctgctttgttggatgtattaaaattggttgcttcttgttactttgaatttcgagatgtgaaatcatttcagcagcgaatgcataaaatt : 2018
Floradade.  : agttgtatcgaatatatgatgatgagatgaaatggtgttgagctgctttgttggatgtattaaaattggttgcttcttgttactttgaatttcgagatgtgaaatcatttcagcagcgaatgcataaaatt : 2018
LYC1410.4   : agttgtatcgaatatatgatgatgagatgaaatggtgttgagctgctttgttggatgtattaaaattggttgcttcttgttactttgaatttcgagatgtgaaatcatttcagcagcgaatgcataaaatt : 2018
PI169588.4  : agttgtatcgaatatatgatgatgagatgaaatggtgttgagctgctttgttggatgtattaaaattggttgcttcttgttactttgaatttcgagatgtgaaatcatttcagcagcgaatgcataaaatt : 2018
Brandywine  : agttgtatcgaatatatgatgatgagatgaaatggtgttgagctgctttgttggatgtattaaaattggttgcttcttgttactttgaatttcgagatgtgaaatcatttcagcagcgaatgcataaaatt : 2018
EA00990.4   : agttgtatcgaatatatgatgatgagatgaaatggtgttgagctgctttgttggatgtattaaaattggttgcttcttgttactttgaatttcgagatgtgaaatcatttcagcagcgaatgcataaaatt : 2018
PI303721.4  : agttgtatcgaatatatgatgatgagatgaaatggtgttgagctgctttgttggatgtattaaaattggttgcttcttgttactttgaatttcgagatgtgaaatcatttcagcagcgaatgcataaaatt : 2018
BGV006775.  : agttgtatcgaatatatgatgatgagatgaaatggtgttgagctgctttgttggatgtattaaaattggttgcttcttgttactttgaatttcgagatgtgaaatcatttcagcagcgaatgcataaaatt : 2018
PAS014479.  : agttgtatcgaatatatgatgatgagatgaaatggtgttgagctgctttgttggatgtattaaaattggttgcttcttgttactttgaatttcgagatgtgaaatcatttcagcagcgaatgcataaaatt : 2018
              AGTTGTATCGAATATATGATGATGAGATGAAATGGTGTTGAGCTGCTTTGTTGGATGTATTAAAATTGGTTGCTTCTTGTTACTTTGAATTTCGAGATGTGAAATCATTTCAGCAGCGAATGCATAAAATT
                                                    epm_Soly_M0_p1m08F_108 2270
                                                        epm_Soly_M0_p0m04R_584 2274
                                                                                             epm_Soly_M0_p1m08F_108 2313
                                                                                                                                epm_Soly_M0_p1m05F_245 2351

                *       2240         *       2260         *       2280         *       2300         *       2320         *       2340         *       23
ITAG.4      : atcaaaattcattgatccgactaatataaatttaccgagcgttttcctctctccctccgtttggttggcagtggacaaaatttattctctcc----------------------ttctatgcctcatacaa : 2127
BGV006865.  : atcaaaattcattgatccgactaatataaatttaccgagcgttttcctctctccctccgtttggttggcagtggacaaaatttattctctccttcatctagtaaaatgatctacttctatgcctcatacaa : 2149
BGV007931.  : atcaaaattcattgatccgactaatataaatttaccgagcgttttcctctctccctccgtttggttggcagtggacaaaatttattctctcc----------------------ttctatgcctcatacaa : 2127
BGV007989.  : atcaaaattcattgatccgactaatataaatttaccgagcgttttcctctctccctccgtttggttggcagtggacaaaatttattctctccttcatctagtaaaatgatctacttctatgcctcatacaa : 2149
M82.4       : atcaaaattcattgatccgactaatataaatttaccgagcgttttcctctctccctccgtttggttggcagtggacaaaatttattctctcc----------------------ttctatgcctcatacaa : 2127
EA00371.4   : atcaaaattcattgatccgactaatataaatttaccgagcgttttcctctctccctccgtttggttggcagtggacaaaatttattctctccttcatctagtaaaatgatctacttctatgcctcatacaa : 2149
Fla.8924.4  : atcaaaattcattgatccgactaatataaatttaccgagcgttttcctctctccctccgtttggttggcagtggacaaaatttattctctcc----------------------ttctatgcctcatacaa : 2127
Floradade.  : atcaaaattcattgatccgactaatataaatttaccgagcgttttcctctctccctccgtttggttggcagtggacaaaatttattctctcc----------------------ttctatgcctcatacaa : 2127
LYC1410.4   : atcaaaattcattgatccgactaatataaatttaccgagcgttttcctctctccctccgtttggttggcagtggacaaaatttattctctcc----------------------ttctatgcctcatacaa : 2127
PI169588.4  : atcaaaattcattgatccgactaatataaatttaccgagcgttttcctctctccctccgtttggttggcagtggacaaaatttattctctcc----------------------ttctatgcctcatacaa : 2127
Brandywine  : atcaaaattcattgatccgactaatataaatttaccgagcgttttcctctctccctccgtttggttggcagtggacaaaatttattctctcc----------------------ttctatgcctcatacaa : 2127
EA00990.4   : atcaaaattcattgatccgactaatataaatttaccgagcgttttcctctctccctccgtttggttggcagtggacaaaatttattctctccttcatctagtaaaatgatctacttctatgcctcatacaa : 2149
PI303721.4  : atcaaaattcattgatccgactaatataaatttaccgagcgttttcctctctccctccgtttggttggcagtggacaaaatttattctctcc----------------------ttctatgcctcatacaa : 2127
BGV006775.  : atcaaaattcattgatccgactaatataaatttaccgagcgttttcctctctccctccgtttggttggcagtggacaaaatttattctctcc----------------------ttctatgcctcatacaa : 2127
PAS014479.  : atcaaaattcattgatccgactaatataaatttaccgagcgttttcctctctccctccgtttggttggcagtggacaaaatttattctctccttcatctagtaaaatgatctacttctatgcctcatacaa : 2149
              ATCAAAATTCATTGATCCGACTAATATAAATTTACCGAGCGTTTTCCTCTCTCCCTCCGTTTGGTTGGCAGTGGACAAAATTTATTCTCTCC                      TTCTATGCCTCATACAA

              60         *       2380         *       2400         *       2420         *       2440         *       2460         *       2480
ITAG.4      : aaagaagtggaacatcttttatttcttctcaaattgtaaaaataaattcccaatccaatgctattgtatcttttaaattcatttaatgacgtcatatatttcttatcaaatactcttttcaatattatgg : 2258
BGV006865.  : aaagaagtggaacatcttttatttcttctcaaattgtaaaaataaattcccaatccaatgctattgtatcttttaaattcatttaatgacgtcatatatttcttatcaaatactcttttcaatattatgg : 2280
BGV007931.  : aaagaagtggaacatcttttatttcttctcaaattgtaaaaataaattcccaatccaatgctattgtatcttttaaattcatttaatgacgtcatatatttcttatcaaatactcttttcaatattatgg : 2258
BGV007989.  : aaagaagtggaacatcttttatttcttctcaaattgtaaaaataaattcccaatccaatgctattgtatcttttaaattcatttaatgacgtcatatatttcttatcaaatactcttttcaatattatgg : 2280
M82.4       : aaagaagtggaacatcttttatttcttctcaaattgtaaaaataaattcccaatccaatgctattgtatcttttaaattcatttaatgacgtcatatatttcttatcaaatactcttttcaatattatgg : 2258
EA00371.4   : aaagaagtggaacatcttttatttcttctcaaattgtaaaaataaattcccaatccaatgctattgtatcttttaaattcatttaatgacgtcatatatttcttatcaaatactcttttcaatattatgg : 2280
Fla.8924.4  : aaagaagtggaacatcttttatttcttctcaaattgtaaaaataaattcccaatccaatgctattgtatcttttaaattcatttaatgacgtcatatatttcttatcaaatactcttttcaatattatgg : 2258
Floradade.  : aaagaagtggaacatcttttatttcttctcaaattgtaaaaataaattcccaatccaatgctattgtatcttttaaattcatttaatgacgtcatatatttcttatcaaatactcttttcaatattatgg : 2258
LYC1410.4   : aaagaagtggaacatcttttatttcttctcaaattgtaaaaataaattcccaatccaatgctattgtatcttttaaattcatttaatgacgtcatatatttcttatcaaatactcttttcaatattatgg : 2258
PI169588.4  : aaagaagtggaacatcttttatttcttctcaaattgtaaaaataaattcccaatccaatgctattgtatcttttaaattcatttaatgacgtcatatatttcttatcaaatactcttttcaatattatgg : 2258
Brandywine  : aaagaagtggaacatcttttatttcttctcaaattgtaaaaataaattcccaatccaatgctattgtatcttttaaattcatttaatgacgtcatatatttcttatcaaatactcttttcaatattatgg : 2258
EA00990.4   : aaagaagtggaacatcttttatttcttctcaaattgtaaaaataaattcccaatccaatgctattgtatcttttaaattcatttaatgacgtcatatatttcttatcaaatactcttttcaatattatgg : 2280
PI303721.4  : aaagaagtggaacatcttttatttcttctcaaattgtaaaaataaattcccaatccaatgctattgtatcttttaaattcatttaatgacgtcatatatttcttatcaaatactcttttcaatattatgg : 2258
BGV006775.  : aaagaagtggaacatcttttatttcttctcaaattgtaaaaataaattcccaatccaatgctattgtatcttttaaattcatttaatgacgtcatatatttcttatcaaatactcttttcaatattatgg : 2258
PAS014479.  : aaagaagtggaacatcttttatttcttctcaaattgtaaaaataaattcccaatccaatgctattgtatcttttaaattcatttaatgacgtcatatatttcttatcaaatactcttttcaatattatgg : 2280
              AAAGAAGTGGAACATCTTTTATTTCTTCTCAAATTGTAAAAATAAATTCCCAATCCAATGCTATTGTATCTTTTAAATTCATTTAATGACGTCATATATTTCTTATCAAATACTCTTTTCAATATTATGG

              *       2500         *       2520         *       2540         *       2560         *       2580         *       2600         *       2620
ITAG.4      : tgcttcgttaaagtacgtctaatcaaatcactttacaatccaactgaatcatttttggctcattggtaaagaaaaaacaagaacacacatatgtttacgaagccaaaagtcactcatgtaagctagcgaat : 2389
BGV006865.  : tgcttcgttaaagtacgtctaatcaaatcactttacaatccaactgaatcattttttagctcattggtaaagaaaaaacaagaacacacatatgtttacgaagccaaaagtcactcatgtaagctagcgaat : 2411
BGV007931.  : tgcttcgttaaagtacgtctaatcaaatcactttacaatccaactgaatcatttttggctcattggtaaagaaaaaacaagaacacacatatgtttacgaagccaaaagtcactcatgtaagctagcgaat : 2389
BGV007989.  : tgcttcgttaaagtacgtctaatcaaatcactttacaatccaactgaatcattttttagctcattggtaaagaaaaaacaagaacacacatatgtttacgaagccaaaagtcactcatgtaagctagcgaat : 2411
M82.4       : tgcttcgttaaagtacgtctaatcaaatcactttacaatccaactgaatcatttttggctcattggtaaagaaaaaacaagaacacacatatgtttacgaagccaaaagtcactcatgtaagctagcgaat : 2389
EA00371.4   : tgcttcgttaaagtacgtctaatcaaatcactttacaatccaactgaatcattttttagctcattggtaaagaaaaaacaagaacacacatatgtttacgaagccaaaagtcactcatgtaagctagcgaat : 2411
Fla.8924.4  : tgcttcgttaaagtacgtctaatcaaatcactttacaatccaactgaatcatttttggctcattggtaaagaaaaaacaagaacacacatatgtttacgaagccaaaagtcactcatgtaagctagcgaat : 2389
Floradade.  : tgcttcgttaaagtacgtctaatcaaatcactttacaatccaactgaatcatttttggctcattggtaaagaaaaaacaagaacacacatatgtttacgaagccaaaagtcactcatgtaagctagcgaat : 2389
LYC1410.4   : tgcttcgttaaagtacgtctaatcaaatcactttacaatccaactgaatcatttttggctcattggtaaagaaaaaacaagaacacacatatgtttacgaagccaaaagtcactcatgtaagctagcgaat : 2389
PI169588.4  : tgcttcgttaaagtacgtctaatcaaatcactttacaatccaactgaatcatttttggctcattggtaaagaaaaaacaagaacacacatatgtttacgaagccaaaagtcactcatgtaagctagcgaat : 2389
Brandywine  : tgcttcgttaaagtacgtctaatcaaatcactttacaatccaactgaatcatttttggctcattggtaaagaaaaaacaagaacacacatatgtttacgaagccaaaagtcactcatgtaagctagcgaat : 2389
EA00990.4   : tgcttcgttaaagtacgtctaatcaaatcactttacaatccaactgaatcattttttagctcattggtaaagaaaaaacaagaacacacatatgtttacgaagccaaaagtcactcatgtaagctagcgaat : 2411
PI303721.4  : tgcttcgttaaagtacgtctaatcaaatcactttacaatccaactgaatcatttttggctcattggtaaagaaaaaacaagaacacacatatgtttacgaagccaaaagtcactcatgtaagctagcgaat : 2389
BGV006775.  : tgcttcgttaaagtacgtctaatcaaatcactttacaatccaactgaatcatttttggctcattggtaaagaaaaaacaagaacacacatatgtttacgaagccaaaagtcactcatgtaagctagcgaat : 2389
PAS014479.  : tgcttcgttaaagtacgtctaatcaaatcactttacaatccaactgaatcattttttagctcattggtaaagaaaaaacaagaacacacatatgtttacgaagccaaaagtcactcatgtaagctagcgaat : 2411
              TGCTTCGTTAAAGTACGTCTAATCAAATCACTTTACAATCCAACTGAATCATTTTT GCTCATTGGTAAAGAAAAAACAAGAACACACATATGTTTACGAAGCCAAAAGTCACTCATGTAAGCTAGCGAAT

                      *       2640         *       2660         *       2680         *       2700         *       2720         *       2740         *
ITAG.4      : ccaaattctactcattttacttttc-----------aggtatatcatataacaatattttatttcatgttttaactatatttttctgaaatgtgctttataataaggaaatgtttaccaactatatatttt : 2509
BGV006865.  : ccaagttctactcattttacttttc-----------aggtatatcatataacaatattttatttcatgttttaaatatatttttctgaaatgtgctttataataaggaaatgtttaccaactatatatttt : 2531
BGV007931.  : ccaaattctactcattttacttttc-----------aggtatatcatataacaatattttatttcatgttttaactatatttttctgaaatgtgctttataataaggaaatgtttaccaactatatatttt : 2509
BGV007989.  : ccaaattctactcattttacttttcatgtgaaatgaagatatatcatataacaatattttatttcatgttttaaatatatttttctggaatgtgctttataataaggaaatgtttaccaactatatatttt : 2542
M82.4       : ccaaattctactcattttacttttc-----------aggtatatcatataacaatattttatttcatgttttaactatatttttctgaaatgtgctttataataaggaaatgtttaccaactatatatttt : 2509
EA00371.4   : ccaaattctactcattttacttttcatgtgaaatgaagatatatcatataacaatattttatttcatgttttaaatatatttttctggaatgtgctttataataaggaaatgtttaccaactatatatttt : 2542
Fla.8924.4  : ccaaattctactcattttacttttc-----------aggtatatcatataacaatattttatttcatgttttaactatatttttctgaaatgtgctttataataaggaaatgtttaccaactatatatttt : 2509
Floradade.  : ccaaattctactcattttacttttc-----------aggtatatcatataacaatattttatttcatgttttaactatatttttctgaaatgtgctttataataaggaaatgtttaccaactatatatttt : 2509
LYC1410.4   : ccaaattctactcattttacttttc-----------aggtatatcatataacaatattttatttcatgttttaactatatttttctgaaatgtgctttataataaggaaatgtttaccaactatatatttt : 2509
PI169588.4  : ccaaattctactcattttacttttc-----------aggtatatcatataacaatattttatttcatgttttaactatatttttctgaaatgtgctttataataaggaaatgtttaccaactatatatttt : 2509
Brandywine  : ccaaattctactcattttacttttc-----------aggtatatcatataacaatattttatttcatgttttaactatatttttctgaaatgtgctttataataaggaaatgtttaccaactatatatttt : 2509
EA00990.4   : ccaaattctactcattttacttttcatgtgaaatgaagatatatcatataacaatattttatttcatgttttaaatatatttttctggaatgtgctttataataaggaaatgtttaccaactatatatttt : 2542
PI303721.4  : ccaaattctactcattttacttttc-----------aggtatatcatataacaatattttatttcatgttttaactatatttttctgaaatgtgctttataataaggaaatgtttaccaactatatatttt : 2509
BGV006775.  : ccaaattctactcattttacttttc-----------aggtatatcatataacaatattttatttcatgttttaactatatttttctgaaatgtgctttataataaggaaatgtttaccaactatatatttt : 2509
PAS014479.  : ccaaattctactcattttacttttc-----------aggtatatcatataacaatattttatttcatgttttaaatatatttttctgaaatgtgctttataataaggaaatgtttaccaactatatatttt : 2531
              CCAAaTTCTACTCATTTTACTTTTC           AGgTATATCATATAACAATATTTTATTTCATGTTTTAA TATATTTTTCTGaAATGTGCTTTATAATAAGGAAATGTTTACCAACTATATATTTT
                                                                                                                             epm_Soly_M0_p0m01F_1500 2863

                  2760         *       2780         *       2800         *       2820         *       2840         *       2860         *       2880
ITAG.4      : gcatgttggagttcgatgtataatttaagcaagaaagctgcaatcaaataggattataccgcagaattaagggttaaaagaagatgtgtgatttatgtatatattggctaaataaaaataaaaataaaata : 2640
BGV006865.  : gcatgttggagttcgatgtataatttaagcaagaaagctgcaatcaaataggattataccgcagaattaagggttaaaagaagatgtgtgatttatgtatatattggctaaataaaaataaaaataaaata : 2662
BGV007931.  : gcatgttggagttcgatgtataatttaagcaagaaagctgcaatcaaataggattataccgcagaattaagggttaaaagaagatgtgtgatttatgtatatattggctaaataaaaataaaaataaaata : 2640
BGV007989.  : gcatgttggagttcgatgtataatttaagcaagaaagctgcaatcaaataggattatactgcagaattaagggttaaaagaagatgtgtgatttatgtatatattggctaaataaaaataaaaataaaata : 2673
M82.4       : gcatgttggagttcgatgtataatttaagcaagaaagctgcaatcaaataggattataccgcagaattaagggttaaaagaagatgtgtgatttatgtatatattggctaaataaaaataaaaataaaata : 2640
EA00371.4   : gcatgttggagttcgatgtataatttaagcaagaaagctgcaatcaaataggattatactgcagaattaagggttaaaagaagatgtgtgatttatgtatatattggctaaataaaaataaaaataaaata : 2673
Fla.8924.4  : gcatgttggagttcgatgtataatttaagcaagaaagctgcaatcaaataggattataccgcagaattaagggttaaaagaagatgtgtgatttatgtatatattggctaaataaaaataaaaataaaata : 2640
Floradade.  : gcatgttggagttcgatgtataatttaagcaagaaagctgcaatcaaataggattataccgcagaattaagggttaaaagaagatgtgtgatttatgtatatattggctaaataaaaataaaaataaaata : 2640
LYC1410.4   : gcatgttggagttcgatgtataatttaagcaagaaagctgcaatcaaataggattataccgcagaattaagggttaaaagaagatgtgtgatttatgtatatattggctaaataaaaataaaaataaaata : 2640
PI169588.4  : gcatgttggagttcgatgtataatttaagcaagaaagctgcaatcaaataggattataccgcagaattaagggttaaaagaagatgtgtgatttatgtatatattggctaaataaaaataaaaataaaata : 2640
Brandywine  : gcatgttggagttcgatgtataatttaagcaagaaagctgcaatcaaataggattataccgcagaattaagggttaaaagaagatgtgtgatttatgtatatattggctaaataaaaataaaaataaaata : 2640
EA00990.4   : gcatgttggagttcgatgtataatttaagcaagaaagctgcaatcaaataggattatactgcagaattaagggttaaaagaagatgtgtgatttatgtatatattggctaaataaaaataaaaataaaata : 2673
PI303721.4  : gcatgttggagttcgatgtataatttaagcaagaaagctgcaatcaaataggattataccgcagaattaagggttaaaagaagatgtgtgatttatgtatatattggctaaataaaaataaaaataaaata : 2640
BGV006775.  : gcatgttggagttcgatgtataatttaagcaagaaagctgcaatcaaataggattataccgcagaattaagggttaaaagaagatgtgtgatttatgtatatattggctaaataaaaataaaaataaaata : 2640
PAS014479.  : gcatgttggagttcgatgtataatttaagcaagaaagctgcaatcaaataggattataccgcagaattaagggttaaaagaagatgtgtgatttatgtatatattggctaaataaaaataaaaataaaata : 2662
              GCATGTTGGAGTTCGATGTATAATTTAAGCAAGAAAGCTGCAATCAAATAGGATTATACcGCAGAATTAAGGGTTAAAAGAAGATGTGTGATTTATGTATATATTGGCTAAATAAAAATAAAAATAAAATA

                      *       2900         *       2920         *       2940         *       2960         *       2980         *       3000         *
ITAG.4      : gtggatttaagaggcccaatttaaagccgttccaagtccgacttgtatcattcgccaaatttaatccgtccttcagatctccctctcctctgtgttagggcagaaaaatctgaagctgattcctccatttt : 2771
BGV006865.  : gtggatttaagaggcccaatttaaagccgttccaagtccgacttatatcattcgccaaatttaatccgtccttcagatctccctctcctctgtgttagggcagaaaaatctgaagctgattcctccatttt : 2793
BGV007931.  : gtggatttaagaggcccaatttaaagccgttccaagtccgacttgtatcattcgccaaatttaatccgtccttcagatctccctctcctctgtgttagggcagaaaaatctgaagctgattcctccatttt : 2771
BGV007989.  : gtggatttaagaggcccaatttaaagccgttccaagtccgacttatatcattcggcaaatttaatccgtccttcagatctccctctcctctgtgttagggcagaaaaatctgaagctgattcctccatttt : 2804
M82.4       : gtggatttaagaggcccaatttaaagccgttccaagtccgacttgtatcattcgccaaatttaatccgtccttcagatctccctctcctctgtgttagggcagaaaaatctgaagctgattcctccatttt : 2771
EA00371.4   : gtggatttaagaggcccaatttaaagccgttccaagtccgacttatatcattcggcaaatttaatccgtccttcagatctccctctcctctgtgttagggcagaaaaatctgaagctgattcctccatttt : 2804
Fla.8924.4  : gtggatttaagaggcccaatttaaagccgttccaagtccgacttgtatcattcgccaaatttaatccgtccttcagatctccctctcctctgtgttagggcagaaaaatctgaagctgattcctccatttt : 2771
Floradade.  : gtggatttaagaggcccaatttaaagccgttccaagtccgacttgtatcattcgccaaatttaatccgtccttcagatctccctctcctctgtgttagggcagaaaaatctgaagctgattcctccatttt : 2771
LYC1410.4   : gtggatttaagaggcccaatttaaagccgttccaagtccgacttgtatcattcgccaaatttaatccgtccttcagatctccctctcctctgtgttagggcagaaaaatctgaagctgattcctccatttt : 2771
PI169588.4  : gtggatttaagaggcccaatttaaagccgttccaagtccgacttgtatcattcgccaaatttaatccgtccttcagatctccctctcctctgtgttagggcagaaaaatctgaagctgattcctccatttt : 2771
Brandywine  : gtggatttaagaggcccaatttaaagccgttccaagtccgacttgtatcattcgccaaatttaatccgtccttcagatctccctctcctctgtgttagggcagaaaaatctgaagctgattcctccatttt : 2771
EA00990.4   : gtggatttaagaggcccaatttaaagccgttccaagtccgacttatatcattcggcaaatttaatccgtccttcagatctccctctcctctgtgttagggcagaaaaatctgaagctgattcctccatttt : 2804
PI303721.4  : gtggatttaagaggcccaatttaaagccgttccaagtccgacttgtatcattcgccaaatttaatccgtccttcagatctccctctcctctgtgttagggcagaaaaatctgaagctgattcctccatttt : 2771
BGV006775.  : gtggatttaagaggcccaatttaaagccgttccaagtccgacttgtatcattcgccaaatttaatccgtccttcagatctccctctcctctgtgttagggcagaaaaatctgaagctgattcctccatttt : 2771
PAS014479.  : gtggatttaagaggcccaatttaaagccgttccaagtccgacttatatcattcgccaaatttaatccgtccttcagatctccctctcctctgtgttagggcagaaaaatctgaagctgattcctccatttt : 2793
              GTGGATTTAAGAGGCCCAATTTAAAGCCGTTCCAAGTCCGACTT TATCATTCGcCAAATTTAATCCGTCCTTCAGATCTCCCTCTCCTCTGTGTTAGGGCAGAAAAATCTGAAGCTGATTCCTCCATTTT
```

```
               3020          *        3040          *        3060          *        3080          *        3100          *        3120          *        3140
ITAG.4     : cttccacaaacgaaaggtatacgctctattattttctccttcctgtacgttaactctattttcccactgtttccagttcgacatttgtagtttcttatctctgtttgtttcagttaagtcccaaatata : 2902
BGV006865. : cttcaacaaacgaaaggtatacgctctattattttctccttcctgtacgttaactctattttcccactgtttccagttcgacatttgtagtttcttatctctgtttgtttcagttaagtcccaaatata : 2924
BGV007931. : cttccacaaacgaaaggtatacgctctattattttctccttcctgtacgttaactctattttcccactgtttccagttcgacatttgtagtttcttatctctgtttgtttcagttaagtcccaaatata : 2902
BGV007989. : cttcaacaaacgaaaggtatacgctctattattttctccttcctgtacgttaactctattttcccactgtttccagttcgacatttgtagtttcttatctctgtttgtttcagttaagtcccaaatata : 2935
M82.4      : cttccacaaacgaaaggtatacgctctattattttctccttcctgtacgttaactctattttcccactgtttccagttcgacatttgtagtttcttatctctgtttgtttcagttaagtcccaaatata : 2902
EA00371.4  : cttcaacaaacgaaaggtatacgctctattattttctccttcctgtacgttaactctattttcccactgtttccagttcgacatttgtagtttcttatctctgtttgtttcagttaagtcccaaatata : 2935
Fla.8924.4 : cttccacaaacgaaaggtatacgctctattattttctccttcctgtacgttaactctattttcccactgtttccagttcgacatttgtagtttcttatctctgtttgtttcagttaagtcccaaatata : 2902
Floradade. : cttccacaaacgaaaggtatacgctctattattttctccttcctgtacgttaactctattttcccactgtttccagttcgacatttgtagtttcttatctctgtttgtttcagttaagtcccaaatata : 2902
LYC1410.4  : cttccacaaacgaaaggtatacgctctattattttctccttcctgtacgttaactctattttcccactgtttccagttcgacatttgtagtttcttatctctgtttgtttcagttaagtcccaaatata : 2902
PI169588.4 : cttccacaaacgaaaggtatacgctctattattttctccttcctgtacgttaactctattttcccactgtttccagttcgacatttgtagtttcttatctctgtttgtttcagttaagtcccaaatata : 2902
Brandywine : cttccacaaacgaaaggtatacgctctattattttctccttcctgtacgttaactctattttcccactgtttccagttcgacatttgtagtttcttatctctgtttgtttcagttaagtcccaaatata : 2902
EA00990.4  : cttcaacaaacgaaaggtatacgctctattattttctccttcctgtacgttaactctattttcccactgtttccagttcgacatttgtagtttcttatctctgtttgtttcagttaagtcccaaatata : 2935
PI303721.4 : cttccacaaacgaaaggtatacgctctattattttctccttcctgtacgttaactctattttcccactgtttccagttcgacatttgtagtttcttatctctgtttgtttcagttaagtcccaaatata : 2902
BGV006775. : cttccacaaacgaaaggtatacgctctattattttctccttcctgtacgttaactctattttcccactgtttccagttcgacatttgtagtttcttatctctgtttgtttcagttaagtcccaaatata : 2902
PAS014479. : cttcaacaaacgaaaggtatacgctctattattttctccttcctgcacgttaactctattttcccactgtttccagttcgacatttgtagtttcttatctctgtttgtttcagttaagtcccaaatata : 2924
             CTTC ACAAACGAAAGGTATACGCTCTATTATTTTCTCCTTCCTGtACGTTAACTCTATTTTCCCACTGTTTCCAGTTCGACATTTGTAGTTTCTTATCTCTGTTTGTTTCAGTTAAGTCCCAAATATA
```

```
                 *        3160          *        3180          *        3200          *        3220          *        3240          *        3260
ITAG.4     : tgatgggtcatgcgatgc--tttctgcattttgcggttgtttttgggatatatagatgtttattacttcttttatttgcctttgaaataaaataatttgtaatttgatggtaaagataa : 3020
BGV006865. : tgatgggtcatgcgatgctttttctgcattttgcggttgtttttgggatatatagatgtttattacttcttttatttgcctttgaaataaaataa------------------------ : 3020
BGV007931. : tgatgggtcatgcgatgc--tttctgcattttgcggttgtttttgggatatatagatgtttattacttcttttatttgcctttgaaataaaataatttgtaatttgatggtaaagataa : 3020
BGV007989. : tgatgggtcatgcgatgc--tttctgcattttgcggttgtttttgggatatatagatgtttattacttcttttatttgcctttgaa--------------------------------- : 3020
M82.4      : tgatgggtcatgcgatgc--tttctgcattttgcggttgtttttgggatatatagatgtttattacttcttttatttgcctttgaaataaaataatttgtaatttgatggtaaagataa : 3020
EA00371.4  : tgatgggtcatgcgatgc--tttctgcattttgcggttgtttttgggatatatagatgtttattacttcttttatttgcctttgaa--------------------------------- : 3020
Fla.8924.4 : tgatgggtcatgcgatgc--tttctgcattttgcggttgtttttgggatatatagatgtttattacttcttttatttgcctttgaaataaaataatttgtaatttgatggtaaagataa : 3020
Floradade. : tgatgggtcatgcgatgc--tttctgcattttgcggttgtttttgggatatatagatgtttattacttcttttatttgcctttgaaataaaataatttgtaatttgatggtaaagataa : 3020
LYC1410.4  : tgatgggtcatgcgatgc--tttctgcattttgcggttgtttttgggatatatagatgtttattacttcttttatttgcctttgaaataaaataatttgtaatttgatggtaaagataa : 3020
PI169588.4 : tgatgggtcatgcgatgc--tttctgcattttgcggttgtttttgggatatatagatgtttattacttcttttatttgcctttgaaataaaataatttgtaatttgatggtaaagataa : 3020
Brandywine : tgatgggtcatgcgatgc--tttctgcattttgcggttgtttttgggatatatagatgtttattacttcttttatttgcctttgaaataaaataatttgtaatttgatggtaaagataa : 3020
EA00990.4  : tgatgggtcatgcgatgc--tttctgcattttgcggttgtttttgggatatatagatgtttattacttcttttatttgcctttgaa--------------------------------- : 3020
PI303721.4 : tgatgggtcatgcgatgc--tttctgcattttgcggttgtttttgggatatatagatgtttattacttcttttatttgcctttgaaataaaataatttgtaatttgatggtaaagataa : 3020
BGV006775. : tgatgggtcatgcgatgc--tttctgcattttgcggttgtttttgggatatatagatgtttattacttcttttatttgcctttgaaataaaataatttgtaatttgatggtaaagataa : 3020
PAS014479. : tgatgggtcatgcgatgc--tttctgcattttgcggttgtttttgggatatatagatgtttattacttcttttatttgcctttgaaataaaataatt---------------------- : 3020
             TGATGGGTCATGCGATGC  TTTCTGCATTTTGCGGTTGTTTTTGGGATATATAGATGTTTATTACTTCTTTTATTTGCCTTTGAAataaaataa
```

Solyc11g012385

```
                     *        20          *        40          *        60          *        80          *       100          *       120          *
ITAG.1     : TTAACGTTGCATTTTATGTTACTTTCTTGCCCTTTGTCTTTCTAATTCTTTATATGCTTCTAAGTTCTAACCAAATTTGCTTTTAATTAATAAATTCTTCTCGTTAAAGAAAAAAGTGAATTTCAATTTTT :  131
BGV006865. : -------------------------------------------AATTCTTTATATGCTTCTAAGTTCTAACCAAATTTGCTTTTAATTAATAAATTCTTCTCGTTAAAGAAAAAAGTGAATTTCAATTTTT :   88
BGV007931. : ------------------------------------------------------------------------------------------------------GTTAAAGAAAAAAGTGAATTTCAATTTTT :   29
BGV007989. : TTAACGTTGCATTTTATGTTACTTTCTTGCCCTTTGTCTTTCTAATTCTTTATATGCTTCTAAGTTCTAACCAAATTTGCTTTTAATTAATAAATTCTTCTCGTTAAAGAAAAAAGTGAATTTCAATTTTT :  131
M82.1      : TTAACGTTGCATTTTATGTTACTTTCTTGCCCTTTGTCTTTCTAATTCTTTATATGCTTCTAAGTTCTAACCAAATTTGCTTTTAATTAATAAATTCTTCTCGTTAAAGAAAAAAGTGAATTTCAATTTTT :  131
EA00371.1  : TTAACGTTGCATTTTATGTTACTTTCTTGCCCTTTGTCTTTCTAATTCTTTATATGCTTCTAAGTTCTAACCAAATTTGCTTTTAATTAATAAATTCTTCTCGTTAAAGAAAAAAGTGAATTTCAATTTTT :  131
Fla.8924.1 : TTAACGTTGCATTTTATGTTACTTTCTTGCCCTTTGTCTTTCTAATTCTTTATATGCTTCTAAGTTCTAACCAAATTTGCTTTTAATTAATAAATTCTTCTCGTTAAAGAAAAAAGTGAATTTCAATTTTT :  131
Floradade. : TTAACGTTGCATTTTATGTTACTTTCTTGCCCTTTGTCTTTCTAATTCTTTATATGCTTCTAAGTTCTAACCAAATTTGCTTTTAATTAATAAATTCTTCTCGTTAAAGAAAAAAGTGAATTTCAATTTTT :  131
LYC1410.1  : TTAACGTTGCATTTTATGTTACTTTCTTGCCCTTTGTCTTTCTAATTCTTTATATGCTTCTAAGTTCTAACCAAATTTGCTTTTAATTAATAAATTCTTCTCGTTAAAGAAAAAAGTGAATTTCAATTTTT :  131
PI169588.1 : TTAACGTTGCATTTTATGTTACTTTCTTGCCCTTTGTCTTTCTAATTCTTTATATGCTTCTAAGTTCTAACCAAATTTGCTTTTAATTAATAAATTCTTCTCGTTAAAGAAAAAAGTGAATTTCAATTTTT :  131
Brandywine : TTAACGTTGCATTTTATGTTACTTTCTTGCCCTTTGTCTTTCTAATTCTTTATATGCTTCTAAGTTCTAACCAAATTTGCTTTTAATTAATAAATTCTTCTCGTTAAAGAAAAAAGTGAATTTCAATTTTT :  131
EA00990.1  : TTAACGTTGCATTTTATGTTACTTTCTTGCCCTTTGTCTTTCTAATTCTTTATATGCTTCTAAGTTCTAACCAAATTTGCTTTTAATTAATAAATTCTTCTCGTTAAAGAAAAAAGTGAATTTCAATTTTT :  131
PI303721.1 : TTAACGTTGCATTTTATGTTACTTTCTTGCCCTTTGTCTTTCTAATTCTTTATATGCTTCTAAGTTCTAACCAAATTTGCTTTTAATTAATAAATTCTTCTCGTTAAAGAAAAAAGTGAATTTCAATTTTT :  131
PAS014479. : --------------------------------------TTTCTAATTCTTTATATGCTTCTAAGTTCTAACCAAATTTGCTTTTAATTAATAAATTCTTCTCGTTAAAGAAAAAAGTGAATTTCAATTTTT :   93
BGV006775. : -------------------------------------------AATTCTTTATATGCTTCTAAGTTCTAACCAAATTTGCTTTTAATTAATAAATTCTTCTCGTTAAAGAAAAAAGTGAATTTCAATTTTT :   88
                                                   tttctaattctttatatgcttctaagttctaaccaaatttgcttttaattaataaattcttctcGTTAAAGAAAAAAGTGAATTTCAATTTTT
```

```
                 140          *       160          *       180          *       200          *       220          *       240          *       260
ITAG.1     : AATTCTTTGTTCTTGTTACTCTTTCAATAGTAATAATTGCTTCAATCTTGGGCCATAGTGTTGCAACAAACGATGGCCCAAGCCCACTTGGTGAATGGCAGCCCATTAAGAACCCAAAATTGATGAAAATG :  262
BGV006865. : AATTCTTTGTTCTTGTTACTCTTTCAATAGTAATAATTGCTTCAATCTTGGGCCATAGTGTTGCAACAAACGATGGCCCAAGCCCACTTGGTGAATGGCAGCCCATTAAGAACCCAAAATTGATGAAAATG :  219
BGV007931. : AATTCTTTGTTCTTGTTACTCTTTCAATAGTAATAATTGCTTCAATCTTGGGCCATAGTGTTGCAACAAACGATGGCCCAAGCCCACTTGGTGAATGGCAGCCCATTAAGAACCCAAAATTGATGAAAATG :  160
BGV007989. : AATTCTTTGTTCTTGTTACTCTTTCAATAGTAATAATTGCTTCAATCTTGGGCCATAGTGTTGCAACAAACGATGGCCCAAGCCCACTTGGTGAATGGCAGCCCATTAAGAACCCAAAATTGATGAAAATG :  262
M82.1      : AATTCTTTGTTCTTGTTACTCTTTCAATAGTAATAATTGCTTCAATCTTGGGCCATAGTGTTGCAACAAACGATGGCCCAAGCCCACTTGGTGAATGGCAGCCCATTAAGAACCCAAAATTGATGAAAATG :  262
EA00371.1  : AATTCTTTGTTCTTGTTACTCTTTCAATAGTAATAATTGCTTCAATCTTGGGCCATAGTGTTGCAACAAACGATGGCCCAAGCCCACTTGGTGAATGGCAGCCCATTAAGAACCCAAAATTGATGAAAATG :  262
Fla.8924.1 : AATTCTTTGTTCTTGTTACTCTTTCAATAGTAATAATTGCTTCAATCTTGGGCCATAGTGTTGCAACAAACGATGGCCCAAGCCCACTTGGTGAATGGCAGCCCATTAAGAACCCAAAATTGATGAAAATG :  262
Floradade. : AATTCTTTGTTCTTGTTACTCTTTCAATAGTAATAATTGCTTCAATCTTGGGCCATAGTGTTGCAACAAACGATGGCCCAAGCCCACTTGGTGAATGGCAGCCCATTAAGAACCCAAAATTGATGAAAATG :  262
LYC1410.1  : AATTCTTTGTTCTTGTTACTCTTTCAATAGTAATAATTGCTTCAATCTTGGGCCATAGTGTTGCAACAAACGATGGCCCAAGCCCACTTGGTGAATGGCAGCCCATTAAGAACCCAAAATTGATGAAAATG :  262
PI169588.1 : AATTCTTTGTTCTTGTTACTCTTTCAATAGTAATAATTGCTTCAATCTTGGGCCATAGTGTTGCAACAAACGATGGCCCAAGCCCACTTGGTGAATGGCAGCCCATTAAGAACCCAAAATTGATGAAAATG :  262
Brandywine : AATTCTTTGTTCTTGTTACTCTTTCAATAGTAATAATTGCTTCAATCTTGGGCCATAGTGTTGCAACAAACGATGGCCCAAGCCCACTTGGTGAATGGCAGCCCATTAAGAACCCAAAATTGATGAAAATG :  262
EA00990.1  : AATTCTTTGTTCTTGTTACTCTTTCAATAGTAATAATTGCTTCAATCTTGGGCCATAGTGTTGCAACAAACGATGGCCCAAGCCCACTTGGTGAATGGCAGCCCATTAAGAACCCAAAATTGATGAAAATG :  262
PI303721.1 : AATTCTTTGTTCTTGTTACTCTTTCAATAGTAATAATTGCTTCAATCTTGGGCCATAGTGTTGCAACAAACGATGGCCCAAGCCCACTTGGTGAATGGCAGCCCATTAAGAACCCAAAATTGATGAAAATG :  262
PAS014479. : AATTCTTTGTTCTTGTTACTCTTTCAATAGTAATAATTGCTTCAATCTTGGGCCATAGTGTTGCAACAAACGATGGCCCAAGCCCACTTGGTGAATGGCAGCCCATTAAGAACCCAAAATTGATGAAAATG :  224
BGV006775. : AATTCTTTGTTCTTGTTACTCTTTCAATAGTAATAATTGCTTCAATCTTGGGCCATAGTGTTGCAACAAACGATGGCCCAAGCCCACTTGGTGAATGGCAGCCCATTAAGAACCCAAAATTGATGAAAATG :  219
             AATTCTTTGTTCTTGTTACTCTTTCAATAGTAATAATTGCTTCAATCTTGGGCCATAGTGTTGCAACAAACGATGGCCCAAGCCCACTTGGTGAATGGCAGCCCATTAAGAACCCAAAATTGATGAAAATG
```

```
                     *       280          *       300          *       320          *       340          *       360          *       380          *
ITAG.1     : GAGAATTTAGATGCTTTAAATAATGTTAATATTAATATTAATAATA---------------------------------------------------ATAAAGAGAGAATCTAGATAAATCCGCTGCAACA :  342
BGV006865. : GAGAATTTAGATGCTTTAAATAATGTTAATATTAATAATAATAATAATAATAATAATA------------ATTATTATTATTCTTATTAATACAAGTATAAAGAGAGAATCTAGATAAATCCGCTGCAACA :  338
BGV007931. : GAGAATTTAGATGCTTTAAATAATGTTAATATTAATAATAATAATAATAATAATAATAATTATTATTATTATTATTATTATTATTATTAATACAAGTATAAAGAGAGAATCTAGATAAATCCGCTGCAACA :  291
BGV007989. : GAGAATTTAGATGCTTTAAATAATGTTAATATTAATATTAATAATA---------------------------------------------------ATAAAGAGAGAATCTAGATAAATCCGCTGCAACA :  342
M82.1      : GAGAATTTAGATGCTTTAAATAATGTTAATATTAATATTAATAATA---------------------------------------------------ATAAAGAGAGAATCTAGATAAATCCGCTGCAACA :  342
EA00371.1  : GAGAATTTAGATGCTTTAAATAATGTTAATATTAATATTAATAATA---------------------------------------------------ATAAAGAGAGAATCTAGATAAATCCGCTGCAACA :  342
Fla.8924.1 : GAGAATTTAGATGCTTTAAATAATGTTAATATTAATATTAATAATA---------------------------------------------------ATAAAGAGAGAATCTAGATAAATCCGCTGCAACA :  342
Floradade. : GAGAATTTAGATGCTTTAAATAATGTTAATATTAATATTAATAATA---------------------------------------------------ATAAAGAGAGAATCTAGATAAATCCGCTGCAACA :  342
LYC1410.1  : GAGAATTTAGATGCTTTAAATAATGTTAATATTAATATTAATAATA---------------------------------------------------ATAAAGAGAGAATCTAGATAAATCCGCTGCAACA :  342
PI169588.1 : GAGAATTTAGATGCTTTAAATAATGTTAATATTAATATTAATAATA---------------------------------------------------ATAAAGAGAGAATCTAGATAAATCCGCTGCAACA :  342
Brandywine : GAGAATTTAGATGCTTTAAATAATGTTAATATTAATATTAATAATA---------------------------------------------------ATAAAGAGAGAATCTAGATAAATCCGCTGCAACA :  342
EA00990.1  : GAGAATTTAGATGCTTTAAATAATGTTAATATTAATATTAATAATA---------------------------------------------------ATAAAGAGAGAATCTAGATAAATCCGCTGCAACA :  342
PI303721.1 : GAGAATTTAGATGCTTTAAATAATGTTAATATTAATATTAATAATA---------------------------------------------------ATAAAGAGAGAATCTAGATAAATCCGCTGCAACA :  342
PAS014479. : GAGAATTTAGATGCTTTAAATAATGTTAATATTAATAATAATAATAATAATAATAATA------------AT-----TTATTATTATTAATATAAGTATAAAGAGAGAATCTAGATAAATCCGCTGCAACA :  338
BGV006775. : GAGAATTTAGATGCTTTAAATAATGTTAATATTAATAATAATAATAATAATAATTATT------------ATTATTATTATTCTTATTAATACAAGTATAAAGAGAGAATCTAGATAAATCCGCTGCAACA :  338
             GAGAATTTAGATGCTTTAAATAATGTTAATATTAATA TAATAATA                                                   ATAAAGAGAGAATCTAGATAAATCCGCTGCAACA
```

epm_Soly_M0_p0m00R_5096 478
epm_Soly_M0_p0m07R_528 479
epm_Soly_M0_p0m01R_1500 502

```
                 400          *       420          *       440          *       460          *       480          *       500          *       520
ITAG.1     : AGTTCAACTAGAATGAAATCAATCAGAATGGTTGTTACCCGT------------------------------------------------TGTATTGGTTGTACCCATTGTATTATATTATTAATTTAAAT :  425
BGV006865. : AGTTCAACTAGAATGAAATCAATCAGAATGGTTGTTACCCGT------------------------------------------------TGTATTGGTTGTACCCATTATATTATATTATTAATTTAAAT :  421
BGV007931. : AGTTCAACTAGAATGAAATCAATCAGAATGGTTGTTACCCGTCGATCGGTTAATTCAATAAAATCGGAATTAGCACTCGATTTCGTTGGCTGTATTGGTTGTACCCATTGTATTATATTATTAATTTAAAT :  422
BGV007989. : AGTTCAACTAGAATGAAATCAATCAGAATGGTTGTTACCCGT------------------------------------------------TGTATTGGTTGTACCCATTGTATTATATTATTAATTTAAAT :  425
M82.1      : AGTTCAACTAGAATGAAATCAATCAGAATGGTTGTTACCCGT------------------------------------------------TGTATTGGTTGTACCCATTGTATTATATTATTAATTTAAAT :  425
EA00371.1  : AGTTCAACTAGAATGAAATCAATCAGAATGGTTGTTACCCGT------------------------------------------------TGTATTGGTTGTACCCATTGTATTATATTATTAATTTAAAT :  425
Fla.8924.1 : AGTTCAACTAGAATGAAATCAATCAGAATGGTTGTTACCCGT------------------------------------------------TGTATTGGTTGTACCCATTGTATTATATTATTAATTTAAAT :  425
Floradade. : AGTTCAACTAGAATGAAATCAATCAGAATGGTTGTTACCCGT------------------------------------------------TGTATTGGTTGTACCCATTGTATTATATTATTAATTTAAAT :  425
LYC1410.1  : AGTTCAACTAGAATGAAATCAATCAGAATGGTTGTTACCCGT------------------------------------------------TGTATTGGTTGTACCCATTGTATTATATTATTAATTTAAAT :  425
PI169588.1 : AGTTCAACTAGAATGAAATCAATCAGAATGGTTGTTACCCGT------------------------------------------------TGTATTGGTTGTACCCATTGTATTATATTATTAATTTAAAT :  425
Brandywine : AGTTCAACTAGAATGAAATCAATCAGAATGGTTGTTACCCGT------------------------------------------------TGTATTGGTTGTACCCATTGTATTATATTATTAATTTAAAT :  425
EA00990.1  : AGTTCAACTAGAATGAAATCAATCAGAATGGTTGTTACCCGT------------------------------------------------TGTATTGGTTGTACCCATTGTATTATATTATTAATTTAAAT :  425
PI303721.1 : AGTTCAACTAGAATGAAATCAATCAGAATGGTTGTTACCCGT------------------------------------------------TGTATTGGTTGTACCCATTGTATTATATTATTAATTTAAAT :  425
PAS014479. : AGTTCAACTAGAATGAAATCAATCAGAATGGTTGTTACCCGT------------------------------------------------TGTATTGGTTGTACCCATTGTATTATATTATTAATTTAAAT :  421
BGV006775. : AGTTCAACTAGAATGAAATCAATCAGAATGGTTGTTACCCGT------------------------------------------------TGTATTGGTTGTACCCATTATATTATATTATTAATTTAAAT :  421
             AGTTCAACTAGAATGAAATCAATCAGAATGGTTGTTACCCGT                                                TGTATTGGTTGTACCCATTgTATTATATTATTAATTTAAAT
```

```
                     *       540          *       560          *       580          *       600          *       620          *       640          *
ITAG.1     : ATAATATTTATTTTAATTATTACGTAATTATATTATATCAATTAAAATTCATTGAGATAACGATGAAATTAGTCATTTTGTGTAACAATTAAATTGATGTGATTACATTAGTCTTTACTTATTATTTAATA :  556
BGV006865. : ATAATATTTATTTTAATTATTACGTAATTATATTATATCAATTAAAATTCATTGAGATAACGATGAAATGAGTCATTTTGTGTAACAATTAAATTGATGTGATTACATTAGTCTTTACTTATTATTTAATA :  552
BGV007931. : ATAATATTTATTTTGATTATTACGTAATTATATTATATCAATTAAAATTCATTGAGATAACGATGAAATGAGTCATTTTGTGTAACAATTAAATTGATGTGATTACATTAGTCTTTACTTATTATTTAATA :  553
BGV007989. : ATAATATTTATTTTAATTATTACGTAATTATATTATATCAATTAAAATTCATTGAGATAACGATGAAATTAGTCATTTTGTGTAACAATTAAATTGATGTGATTACATTAGTCTTTACTTATTATTTAATA :  556
M82.1      : ATAATATTTATTTTAATTATTACGTAATTATATTATATCAATTAAAATTCATTGAGATAACGATGAAATTAGTCATTTTGTGTAACAATTAAATTGATGTGATTACATTAGTCTTTACTTATTATTTAATA :  556
EA00371.1  : ATAATATTTATTTTAATTATTACGTAATTATATTATATCAATTAAAATTCATTGAGATAACGATGAAATTAGTCATTTTGTGTAACAATTAAATTGATGTGATTACATTAGTCTTTACTTATTATTTAATA :  556
Fla.8924.1 : ATAATATTTATTTTAATTATTACGTAATTATATTATATCAATTAAAATTCATTGAGATAACGATGAAATTAGTCATTTTGTGTAACAATTAAATTGATGTGATTACATTAGTCTTTACTTATTATTTAATA :  556
Floradade. : ATAATATTTATTTTAATTATTACGTAATTATATTATATCAATTAAAATTCATTGAGATAACGATGAAATTAGTCATTTTGTGTAACAATTAAATTGATGTGATTACATTAGTCTTTACTTATTATTTAATA :  556
LYC1410.1  : ATAATATTTATTTTAATTATTACGTAATTATATTATATCAATTAAAATTCATTGAGATAACGATGAAATTAGTCATTTTGTGTAACAATTAAATTGATGTGATTACATTAGTCTTTACTTATTATTTAATA :  556
PI169588.1 : ATAATATTTATTTTAATTATTACGTAATTATATTATATCAATTAAAATTCATTGAGATAACGATGAAATTAGTCATTTTGTGTAACAATTAAATTGATGTGATTACATTAGTCTTTACTTATTATTTAATA :  556
Brandywine : ATAATATTTATTTTAATTATTACGTAATTATATTATATCAATTAAAATTCATTGAGATAACGATGAAATTAGTCATTTTGTGTAACAATTAAATTGATGTGATTACATTAGTCTTTACTTATTATTTAATA :  556
EA00990.1  : ATAATATTTATTTTAATTATTACGTAATTATATTATATCAATTAAAATTCATTGAGATAACGATGAAATTAGTCATTTTGTGTAACAATTAAATTGATGTGATTACATTAGTCTTTACTTATTATTTAATA :  556
PI303721.1 : ATAATATTTATTTTAATTATTACGTAATTATATTATATCAATTAAAATTCATTGAGATAACGATGAAATTAGTCATTTTGTGTAACAATTAAATTGATGTGATTACATTAGTCTTTACTTATTATTTAATA :  556
PAS014479. : ATAATATTTATTTTGATTATTACGTAATTATATTATATCAATTAAAATTCATTGAGATAACGATGAAATGAGTCATTTTGTGTAACAATTAAATTGATGTGATTACATTAGTCTTTACTTATTATTTAATA :  552
```

```
BGV006775. : ATAATATTTATTTTAATTATTACGTAATTATATTATATCAATTAAAATTCATTGAGATAACGATGAAATGAGTCATTTTGTGTAACAATTAAATTGATGTGATTACATTAGTCTTTACTTATTATTTAATA : 552
             ATAATATTTATTTTaATTATTACGTAATTATATTATATCAATTAAAATTCATTGAGATAACGATGAAAT AGTCATTTTGTGTAACAATTAAATTGATGTGATTACATTAGTCTTTACTTATTATTTAATA
                                                        epm_Soly_M0_p1m09R_86 702
               660       *       680       *       700       *       720       *       740       *       760       *       780
ITAG.1     : ATTGGTCAATATAA-TTTTTTCTTTTATCCTACTTTTAATAGTAACTTTGTCATGTCCTCTACTTTCTCTTGTAATTTATCA--CAAATTATATATGTGTGACAATATATAACAATGAAGAACAATAAAAT : 684
BGV006865. : ATTGGTCAATATAA-TTTTTTCTTTTATCCTACTTTTAATAGTAACTTTGTCATGTCCTCTACTTTCTCTCGTAATTTATCATGCAAATTATATATGTGTGACAATATATAACAATGAAGAACAATAAAAT : 682
BGV007931. : ATTGGTCAATATAATTTTTTTCTTTTATTCTACTTTTAATAGTAACTTTGTCATGTCCTCTACTTTCTCTTGTAATTTATCATGCAAATTATATATGTGTGACAATATATAACAATGAAGAACAATAAAAT : 684
BGV007989. : ATTGGTCAATATAA-TTTTTTCTTTTATCCTACTTTTAATAGTAACTTTGTCATGTCCTCTACTTTCTCTTGTAATTTATCA--CAAATTATATATGTGTGACAATATATAACAATGAAGAACAATAAAAT : 684
M82.1      : ATTGGTCAATATAA-TTTTTTCTTTTATCCTACTTTTAATAGTAACTTTGTCATGTCCTCTACTTTCTCTTGTAATTTATCA--CAAATTATATATGTGTGACAATATATAACAATGAAGAACAATAAAAT : 684
EA00371.1  : ATTGGTCAATATAA-TTTTTTCTTTTATCCTACTTTTAATAGTAACTTTGTCATGTCCTCTACTTTCTCTTGTAATTTATCA--CAAATTATATATGTGTGACAATATATAACAATGAAGAACAATAAAAT : 684
Fla.8924.1 : ATTGGTCAATATAA-TTTTTTCTTTTATCCTACTTTTAATAGTAACTTTGTCATGTCCTCTACTTTCTCTTGTAATTTATCA--CAAATTATATATGTGTGACAATATATAACAATGAAGAACAATAAAAT : 684
Floradade. : ATTGGTCAATATAA-TTTTTTCTTTTATCCTACTTTTAATAGTAACTTTGTCATGTCCTCTACTTTCTCTTGTAATTTATCA--CAAATTATATATGTGTGACAATATATAACAATGAAGAACAATAAAAT : 684
LYC1410.1  : ATTGGTCAATATAA-TTTTTTCTTTTATCCTACTTTTAATAGTAACTTTGTCATGTCCTCTACTTTCTCTTGTAATTTATCA--CAAATTATATATGTGTGACAATATATAACAATGAAGAACAATAAAAT : 684
PI169588.1 : ATTGGTCAATATAA-TTTTTTCTTTTATCCTACTTTTAATAGTAACTTTGTCATGTCCTCTACTTTCTCTTGTAATTTATCA--CAAATTATATATGTGTGACAATATATAACAATGAAGAACAATAAAAT : 684
Brandywine : ATTGGTCAATATAA-TTTTTTCTTTTATCCTACTTTTAATAGTAACTTTGTCATGTCCTCTACTTTCTCTTGTAATTTATCA--CAAATTATATATGTGTGACAATATATAACAATGAAGAACAATAAAAT : 684
EA00990.1  : ATTGGTCAATATAA-TTTTTTCTTTTATCCTACTTTTAATAGTAACTTTGTCATGTCCTCTACTTTCTCTTGTAATTTATCA--CAAATTATATATGTGTGACAATATATAACAATGAAGAACAATAAAAT : 684
PI303721.1 : ATTGGTCAATATAA-TTTTTTCTTTTATCCTACTTTTAATAGTAACTTTGTCATGTCCTCTACTTTCTCTTGTAATTTATCA--CAAATTATATATGTGTGACAATATATAACAATGAAGAACAATAAAAT : 684
PAS014479. : ATTGGTCAATATAA-TTTTTTCTTTTATCCTACTTTTAATAGTAACTTTGTCATGTCCTCTACTTTCTCTTGTAATTTATCATGCAAATTATATATGTGTGACAATATATAACAATGAAGAACAATAAAAT : 682
BGV006775. : ATTGGTCAATATAA-TTTTTTCTTTTATCCTACTTTTAATAGTAACTTTGTCATGTCCTCTACTTTCTCTTGTAATTTATCATGCAAATTATATATGTGTGACAATATATAACAATGAAGAACAATAAAAT : 682
             ATTGGTCAATATAA TTTTTTCTTTTATcCTACTTTTAATAGTAACTTTGTCATGTCCTCTACTTTCTCTtGTAATTTATCA  CAAATTATATATGTGTGACAATATATAACAATGAAGAACAATAAAAT
                                          epm_Soly_M0_p1m02F_1369 813
                *       800       *       820       *       840       *       860       *       880       *       900       *
ITAG.1     : CTATCCAAACGCTATATTCATTAAAACAATACAACATAATATAATACAATACATTATAAACAATATATAATAACCATCCAATTAAAGTATTACTTAACATTCTTATTCATTTTCTCCTCGTTCTTACCAAA : 815
BGV006865. : CTATCCAAACGCTATATTCATTAAAACAATACAACATAATATAATACAATACATTATAAACAATATATAATAACCATCCAATCAAAGTATTACTTAACATTCTTATTCATTTTCTCCTCGTTCTTACCAAA : 813
BGV007931. : CTATCCAAACGCTATATTCATTAAAACAATACAACATAATATAATACAATACATTATAAACAATATATAATAACCATCCAATTAAAGTATTACTTAACATTCTTATTCATTTTCTCCTCGTTCTTACCAAA : 815
BGV007989. : CTATCCAAACGCTATATTCATTAAAACAATACAACATAATATAATACAATACATTATAAACAATATATAATAACCATCCAATTAAAGTATTACTTAACATTCTTATTCATTTTCTCCTCGTTCTTACCAAA : 815
M82.1      : CTATCCAAACGCTATATTCATTAAAACAATACAACATAATATAATACAATACATTATAAACAATATATAATAACCATCCAATTAAAGTATTACTTAACATTCTTATTCATTTTCTCCTCGTTCTTACCAAA : 815
EA00371.1  : CTATCCAAACGCTATATTCATTAAAACAATACAACATAATATAATACAATACATTATAAACAATATATAATAACCATCCAATTAAAGTATTACTTAACATTCTTATTCATTTTCTCCTCGTTCTTACCAAA : 815
Fla.8924.1 : CTATCCAAACGCTATATTCATTAAAACAATACAACATAATATAATACAATACATTATAAACAATATATAATAACCATCCAATTAAAGTATTACTTAACATTCTTATTCATTTTCTCCTCGTTCTTACCAAA : 815
Floradade. : CTATCCAAACGCTATATTCATTAAAACAATACAACATAATATAATACAATACATTATAAACAATATATAATAACCATCCAATTAAAGTATTACTTAACATTCTTATTCATTTTCTCCTCGTTCTTACCAAA : 815
LYC1410.1  : CTATCCAAACGCTATATTCATTAAAACAATACAACATAATATAATACAATACATTATAAACAATATATAATAACCATCCAATTAAAGTATTACTTAACATTCTTATTCATTTTCTCCTCGTTCTTACCAAA : 815
PI169588.1 : CTATCCAAACGCTATATTCATTAAAACAATACAACATAATATAATACAATACATTATAAACAATATATAATAACCATCCAATTAAAGTATTACTTAACATTCTTATTCATTTTCTCCTCGTTCTTACCAAA : 815
Brandywine : CTATCCAAACGCTATATTCATTAAAACAATACAACATAATATAATACAATACATTATAAACAATATATAATAACCATCCAATTAAAGTATTACTTAACATTCTTATTCATTTTCTCCTCGTTCTTACCAAA : 815
EA00990.1  : CTATCCAAACGCTATATTCATTAAAACAATACAACATAATATAATACAATACATTATAAACAATATATAATAACCATCCAATTAAAGTATTACTTAACATTCTTATTCATTTTCTCCTCGTTCTTACCAAA : 815
PI303721.1 : CTATCCAAACGCTATATTCATTAAAACAATACAACATAATATAATACAATACATTATAAACAATATATAATAACCATCCAATTAAAGTATTACTTAACATTCTTATTCATTTTCTCCTCGTTCTTACCAAA : 815
PAS014479. : CTATCCAAACGCTATATTCATTAAAACAATACAACATAATATAATACAATACATTATAAACAATATATAATAACCATCCAATCAAAGTATTACTTAACATTCTTATTCATTTTCTCCTCGTTCTTACCAAA : 813
BGV006775. : CTATCTAAACGCTATATTCATTAAAACAATACAACATAATATAATACAATACATTATAAACAATATATAATAACCATCCAATCAAAGTATTACTTAACATTCTTATTCATTTTCTCCTCGTTCTTACCAAA : 813
             CTATCcAAACGCTATATTCATTAAAACAATACAACATAATATAATACAATACATTATAAACAATATATAATAACCATCCAATtAAAGTATTACTTAACATTCTTATTCATTTTCTCCTCGTTCTTACCAAA
      epm_Soly_M0_p1m02F_1369 915
              920       *       940       *       960       *       980       *      1000       *      1020       *      1040
ITAG.1     : AAAAATAAAACTTACAATTTTTTTTATGAAATATCACCTGCTACATTAATTACACGATCCTTAAACGTAATAACAATACTTCCATGATAATAATTTAAAATTTTTGTAAATATGAATATATGATTGTCGGT : 946
BGV006865. : AAAAATAAAACTTACAAATTTTTTTATGAGATATCACCTGCTACATTAATTACACGATCCTTAAACGTAATAACAATACTTCCATGATAATAATTTAAAATTTTTGTAAATATGAATATATGATTGTCGGT : 944
BGV007931. : AAAAATAAAACTTACAATTTTTTTTATGAAATATCACCTGCTACGTTAATTACACGATCCTTAAACGTAATAACAATACTTCCATGATAATAATTTAAAATTTTTGTAAATATGAATATATGATTGTCGGT : 946
BGV007989. : AAAAATAAAACTTACAATTTTTTTTATGAAATATCACCTGCTACATTAATTACACGATCCTTAAACGTAATAACAATACTTCCATGATAATAATTTAAAATTTTTGTAAATATGAATATATGATTGTCGGT : 946
M82.1      : AAAAATAAAACTTACAATTTTTTTTATGAAATATCACCTGCTACATTAATTACACGATCCTTAAACGTAATAACAATACTTCCATGATAATAATTTAAAATTTTTGTAAATATGAATATATGATTGTCGGT : 946
EA00371.1  : AAAAATAAAACTTACAATTTTTTTTATGAAATATCACCTGCTACATTAATTACACGATCCTTAAACGTAATAACAATACTTCCATGATAATAATTTAAAATTTTTGTAAATATGAATATATGATTGTCGGT : 946
Fla.8924.1 : AAAAATAAAACTTACAATTTTTTTTATGAAATATCACCTGCTACATTAATTACACGATCCTTAAACGTAATAACAATACTTCCATGATAATAATTTAAAATTTTTGTAAATATGAATATATGATTGTCGGT : 946
Floradade. : AAAAATAAAACTTACAATTTTTTTTATGAAATATCACCTGCTACATTAATTACACGATCCTTAAACGTAATAACAATACTTCCATGATAATAATTTAAAATTTTTGTAAATATGAATATATGATTGTCGGT : 946
LYC1410.1  : AAAAATAAAACTTACAATTTTTTTTATGAAATATCACCTGCTACATTAATTACACGATCCTTAAACGTAATAACAATACTTCCATGATAATAATTTAAAATTTTTGTAAATATGAATATATGATTGTCGGT : 946
PI169588.1 : AAAAATAAAACTTACAATTTTTTTTATGAAATATCACCTGCTACATTAATTACACGATCCTTAAACGTAATAACAATACTTCCATGATAATAATTTAAAATTTTTGTAAATATGAATATATGATTGTCGGT : 946
Brandywine : AAAAATAAAACTTACAATTTTTTTTATGAAATATCACCTGCTACATTAATTACACGATCCTTAAACGTAATAACAATACTTCCATGATAATAATTTAAAATTTTTGTAAATATGAATATATGATTGTCGGT : 946
EA00990.1  : AAAAATAAAACTTACAATTTTTTTTATGAAATATCACCTGCTACATTAATTACACGATCCTTAAACGTAATAACAATACTTCCATGATAATAATTTAAAATTTTTGTAAATATGAATATATGATTGTCGGT : 946
PI303721.1 : AAAAATAAAACTTACAATTTTTTTTATGAAATATCACCTGCTACATTAATTACACGATCCTTAAACGTAATAACAATACTTCCATGATAATAATTTAAAATTTTTGTAAATATGAATATATGATTGTCGGT : 946
PAS014479. : AAAAATAAAACTTACAAATTTTTTTATGAGATATCACCTGCTACATTAATTACACGATCCTTAAACGTAATAACAATACTTCCATGATAATAATTTAAAATTTTTGTAAATATGAATATATGATTGTCGGT : 944
BGV006775. : AAAAATAAAACTTACAATTTTTTTTATGAGATATCACCTGCTACATTAATTACACGATCCTTAAACGTAATAACAATACTTCCATGATAATAATTTAAAATTTTTGTAAATATGAATATATGATTGTCGGT : 944
             AAAAATAAAACTTACAAtTTTTTTTATGAaATATCACCTGCTACaTTAATTACACGATCCTTAAACGTAATAACAATACTTCCATGATAATAATTTAAAATTTTTGTAAATATGAATATATGATTGTCGGT
                                                         epm_Soly_M0_p1m08F_108 1102                           epm_Soly_M0_p1m02F_1369 1148
                                                                                                                epm_Soly_M0_p1m02R_1369 1149

                                                                                                                epm_Soly_M0_p0m03F_722 1149

               *      1060       *      1080       *      1100       *      1120       *      1140       *      1160       *      118
ITAG.1     : ATTCCATTTTACCTAATACTTTCCTTTAAATAAGTGCA--CATTCATTAAAAAAACCCCACTTTTTCTCTTGAATTTTTTTTTTTCATTTTTCTTTCTC-AAAAAAAACAAAAAACTAAAAAAATGACCATC : 1074
BGV006865. : ATTCCATTTTACCTAATACTTTCCTTTAAATAAGTGCACCCATTCATTAAAAAAACCCCACTTTTTCTCTTGAATTTTTTTTTTTCATTTTTCTTTCTC-AAAAAAAACAAAAAACTAAAAAAATGACCATC : 1074
BGV007931. : ATTCCATTTTACCTAATACTTTCCTTTAAATAAGTGCA--CATTCATTAAAAAAACCCCACTTTTTCTCTTGAA-TTTTTTTTTTCATTTTTCTTTCTCAAAAAAAAACAAAAAACTAAAAAAATGACCATC : 1074
BGV007989. : ATTCCATTTTACCTAATACTTTCCTTTAAATAAGTGCA--CATTCATTAAAAAAACCCCACTTTTTCTCTTGAATTTTTTTTTTTCATTTTTCTTTCTC-AAAAAAAACAAAAAACTAAAAAAATGACCATC : 1074
M82.1      : ATTCCATTTTACCTAATACTTTCCTTTAAATAAGTGCA--CATTCATTAAAAAAACCCCACTTTTTCTCTTGAATTTTTTTTTTTCATTTTTCTTTCTC-AAAAAAAACAAAAAACTAAAAAAATGACCATC : 1074
EA00371.1  : ATTCCATTTTACCTAATACTTTCCTTTAAATAAGTGCA--CATTCATTAAAAAAACCCCACTTTTTCTCTTGAATTTTTTTTTTTCATTTTTCTTTCTC-AAAAAAAACAAAAAACTAAAAAAATGACCATC : 1074
Fla.8924.1 : ATTCCATTTTACCTAATACTTTCCTTTAAATAAGTGCA--CATTCATTAAAAAAACCCCACTTTTTCTCTTGAATTTTTTTTTTTCATTTTTCTTTCTC-AAAAAAAACAAAAAACTAAAAAAATGACCATC : 1074
Floradade. : ATTCCATTTTACCTAATACTTTCCTTTAAATAAGTGCA--CATTCATTAAAAAAACCCCACTTTTTCTCTTGAATTTTTTTTTTTCATTTTTCTTTCTC-AAAAAAAACAAAAAACTAAAAAAATGACCATC : 1074
LYC1410.1  : ATTCCATTTTACCTAATACTTTCCTTTAAATAAGTGCA--CATTCATTAAAAAAACCCCACTTTTTCTCTTGAATTTTTTTTTTTCATTTTTCTTTCTC-AAAAAAAACAAAAAACTAAAAAAATGACCATC : 1074
PI169588.1 : ATTCCATTTTACCTAATACTTTCCTTTAAATAAGTGCA--CATTCATTAAAAAAACCCCACTTTTTCTCTTGAATTTTTTTTTTTCATTTTTCTTTCTC-AAAAAAAACAAAAAACTAAAAAAATGACCATC : 1074
Brandywine : ATTCCATTTTACCTAATACTTTCCTTTAAATAAGTGCA--CATTCATTAAAAAAACCCCACTTTTTCTCTTGAATTTTTTTTTTTCATTTTTCTTTCTC-AAAAAAAACAAAAAACTAAAAAAATGACCATC : 1074
EA00990.1  : ATTCCATTTTACCTAATACTTTCCTTTAAATAAGTGCA--CATTCATTAAAAAAACCCCACTTTTTCTCTTGAATTTTTTTTTTTCATTTTTCTTTCTC-AAAAAAAACAAAAAACTAAAAAAATGACCATC : 1074
PI303721.1 : ATTCCATTTTACCTAATACTTTCCTTTAAATAAGTGCA--CATTCATTAAAAAAACCCCACTTTTTCTCTTGAATTTTTTTTTTTCATTTTTCTTTCTC-AAAAAAAACAAAAAACTAAAAAAATGACCATC : 1074
PAS014479. : ATTCCATTTTACCTAATACTTTCCTTTAAATAAGTGCACCCATTCATTAAAAAAACCCCACTTTTTCTCTTGAATTTTTTTTTTTCATTTTTCTTTCTC-AAAAAAAACAAAAAACTAAAAAAATGACCATC : 1074
BGV006775. : ATTCCATTTTACCTAATACTTTCCTTTAAATAAGTGCACCCATTCATTAAAAAAACCCCACTTTTTCTCTTGAATTTTTTTTTTTCATTTTTCTTTCTC-AAAAAAAACAAAAAACTAAAAAAATGACCATC : 1074
             ATTCCATTTTACCTAATACTTTCCTTTAAATAAGTGCA  CATTCATTAAAAAAACCCCACTTTTTCTCTTGAAtTTTTTTTTTTCATTTTTCTTTCTC AAAAAAAACAAAAAACTAAAAAAATGACCATC
                                                                                  epm_Soly_M0_p0m12R_252 1251
             0       *      1200       *      1220       *      1240       *      1260       *      1280       *      1300       *
ITAG.1     : AAATTATTTTCTGTTTTCTCCTTGACTCTTTCAATTATCTTAATTTCTTCATTTTTCTTCTATTTCTCCGCCGCGTTGGGTGGCGTACCTGGTGGTTGGACCCCCTTAAATGACGTAAATACCCCTGATGT : 1205
BGV006865. : AAATTATTTTCTGTTTTCTCCTTGACTCTTTCAATTATCTTAATTTCTTCATTTTTCTTCCATTTCTCCGCCGCGTTGGGTGGCGTACCTGGTGGTTGGACCCCCTTAAATGACGTAAATACCCCTGATGT : 1205
BGV007931. : AAATTATTTTCTGTTTTCTCCTTGACTCTTTCAATTATCTTAATTTCTTCATTTTTCTTCTATTTCTCCGCCGCGTTGGGTGGCGTACCTGGTGGTTGGACCCCCTTAAATGACGTAAATACCCCTGATGT : 1205
BGV007989. : AAATTATTTTCTGTTTTCTCCTTGACTCTTTCAATTATCTTAATTTCTTCATTTTTCTTCTATTTCTCCGCCGCGTTGGGTGGCGTACCTGGTGGTTGGACCCCCTTAAATGACGTAAATACCCCTGATGT : 1205
M82.1      : AAATTATTTTCTGTTTTCTCCTTGACTCTTTCAATTATCTTAATTTCTTCATTTTTCTTCTATTTCTCCGCCGCGTTGGGTGGCGTACCTGGTGGTTGGACCCCCTTAAATGACGTAAATACCCCTGATGT : 1205
EA00371.1  : AAATTATTTTCTGTTTTCTCCTTGACTCTTTCAATTATCTTAATTTCTTCATTTTTCTTCTATTTCTCCGCCGCGTTGGGTGGCGTACCTGGTGGTTGGACCCCCTTAAATGACGTAAATACCCCTGATGT : 1205
Fla.8924.1 : AAATTATTTTCTGTTTTCTCCTTGACTCTTTCAATTATCTTAATTTCTTCATTTTTCTTCTATTTCTCCGCCGCGTTGGGTGGCGTACCTGGTGGTTGGACCCCCTTAAATGACGTAAATACCCCTGATGT : 1205
Floradade. : AAATTATTTTCTGTTTTCTCCTTGACTCTTTCAATTATCTTAATTTCTTCATTTTTCTTCTATTTCTCCGCCGCGTTGGGTGGCGTACCTGGTGGTTGGACCCCCTTAAATGACGTAAATACCCCTGATGT : 1205
LYC1410.1  : AAATTATTTTCTGTTTTCTCCTTGACTCTTTCAATTATCTTAATTTCTTCATTTTTCTTCTATTTCTCCGCCGCGTTGGGTGGCGTACCTGGTGGTTGGACCCCCTTAAATGACGTAAATACCCCTGATGT : 1205
PI169588.1 : AAATTATTTTCTGTTTTCTCCTTGACTCTTTCAATTATCTTAATTTCTTCATTTTTCTTCTATTTCTCCGCCGCGTTGGGTGGCGTACCTGGTGGTTGGACCCCCTTAAATGACGTAAATACCCCTGATGT : 1205
Brandywine : AAATTATTTTCTGTTTTCTCCTTGACTCTTTCAATTATCTTAATTTCTTCATTTTTCTTCTATTTCTCCGCCGCGTTGGGTGGCGTACCTGGTGGTTGGACCCCCTTAAATGACGTAAATACCCCTGATGT : 1205
EA00990.1  : AAATTATTTTCTGTTTTCTCCTTGACTCTTTCAATTATCTTAATTTCTTCATTTTTCTTCTATTTCTCCGCCGCGTTGGGTGGCGTACCTGGTGGTTGGACCCCCTTAAATGACGTAAATACCCCTGATGT : 1205
PI303721.1 : AAATTATTTTCTGTTTTCTCCTTGACTCTTTCAATTATCTTAATTTCTTCATTTTTCTTCTATTTCTCCGCCGCGTTGGGTGGCGTACCTGGTGGTTGGACCCCCTTAAATGACGTAAATACCCCTGATGT : 1205
PAS014479. : AAATTATTTTCTGTTTTCTCCTTGACTCTTTCAATTATCTTAATTTCTTCATTTTTCTTCCATTTCTCCGCCGCGTTGGGTGGCGTACCTGGTGGTTGGACCCCCTTAAATGACGTAAATACCCCTGATGT : 1205
BGV006775. : AAATTATTTTCTGTTTTCTCCTTGACTCTTTCAATTATCTTAATTTCTTCATTTTTCTTCCATTTCTCCGCCGCGTTGGGTGGCGTACCTGGTGGTTGGACCCCCTTAAATGACGTAAATACCCCTGATGT : 1205
             AAATTATTTTCTGTTTTCTCCTTGACTCTTTCAATTATCTTAATTTCTTCATTTTTCTTCtATTTCTCCGCCGCGTTGGGTGGCGTACCTGGTGGTTGGACCCCCTTAAATGACGTAAATACCCCTGATGT

                1320       *      1340       *      1360       *      1380       *      1400       *      1420       *      1440
ITAG.1     : GGTTGCGATAGGACAATTTGCAATAAATGAACACAACAAAGAGGCAGGGACAAAATTGGAATTTCAAAGTATTACTAAAGGAGAAAGTCAAGTTGTTGCCGGAACAAATTATCGACTTGTAATTAATGCTA : 1336
BGV006865. : GGTTGCGATAGGACAATTTGCGATAAATGAACACAACAAAGAGGCAGGGACAAAATTGGAATTTCAAAGTATTACTAAAGGAGAAAGTCAAGTTGTTGCCGGAACAAATTATCGACTTGTAATTAATGCTA : 1336
BGV007931. : GGTTGCGATAGGACAATTTGCAATAAATGAACACAACAAAGAGGCAGGGACAAAATTGGAATTTCAAAGTATTACTAAAGGAGAAAGTCAAGTTGTTGCCGGAACAAATTATCGACTTGTAATTAATGCTA : 1336
BGV007989. : GGTTGCGATAGGACAATTTGCAATAAATGAACACAACAAAGAGGCAGGGACAAAATTGGAATTTCAAAGTATTACTAAAGGAGAAAGTCAAGTTGTTGCCGGAACAAATTATCGACTTGTAATTAATGCTA : 1336
M82.1      : GGTTGCGATAGGACAATTTGCAATAAATGAACACAACAAAGAGGCAGGGACAAAATTGGAATTTCAAAGTATTACTAAAGGAGAAAGTCAAGTTGTTGCCGGAACAAATTATCGACTTGTAATTAATGCTA : 1336
EA00371.1  : GGTTGCGATAGGACAATTTGCAATAAATGAACACAACAAAGAGGCAGGGACAAAATTGGAATTTCAAAGTATTACTAAAGGAGAAAGTCAAGTTGTTGCCGGAACAAATTATCGACTTGTAATTAATGCTA : 1336
Fla.8924.1 : GGTTGCGATAGGACAATTTGCAATAAATGAACACAACAAAGAGGCAGGGACAAAATTGGAATTTCAAAGTATTACTAAAGGAGAAAGTCAAGTTGTTGCCGGAACAAATTATCGACTTGTAATTAATGCTA : 1336
Floradade. : GGTTGCGATAGGACAATTTGCAATAAATGAACACAACAAAGAGGCAGGGACAAAATTGGAATTTCAAAGTATTACTAAAGGAGAAAGTCAAGTTGTTGCCGGAACAAATTATCGACTTGTAATTAATGCTA : 1336
LYC1410.1  : GGTTGCGATAGGACAATTTGCAATAAATGAACACAACAAAGAGGCAGGGACAAAATTGGAATTTCAAAGTATTACTAAAGGAGAAAGTCAAGTTGTTGCCGGAACAAATTATCGACTTGTAATTAATGCTA : 1336
PI169588.1 : GGTTGCGATAGGACAATTTGCAATAAATGAACACAACAAAGAGGCAGGGACAAAATTGGAATTTCAAAGTATTACTAAAGGAGAAAGTCAAGTTGTTGCCGGAACAAATTATCGACTTGTAATTAATGCTA : 1336
Brandywine : GGTTGCGATAGGACAATTTGCAATAAATGAACACAACAAAGAGGCAGGGACAAAATTGGAATTTCAAAGTATTACTAAAGGAGAAAGTCAAGTTGTTGCCGGAACAAATTATCGACTTGTAATTAATGCTA : 1336
EA00990.1  : GGTTGCGATAGGACAATTTGCAATAAATGAACACAACAAAGAGGCAGGGACAAAATTGGAATTTCAAAGTATTACTAAAGGAGAAAGTCAAGTTGTTGCCGGAACAAATTATCGACTTGTAATTAATGCTA : 1336
PI303721.1 : GGTTGCGATAGGACAATTTGCAATAAATGAACACAACAAAGAGGCAGGGACAAAATTGGAATTTCAAAGTATTACTAAAGGAGAAAGTCAAGTTGTTGCCGGAACAAATTATCGACTTGTAATTAATGCTA : 1336
PAS014479. : GGTTGCGATAGGACAATTTGCGATAAATGAACACAACAAAGAGGCAGGGACAAAATTGGAATTTCAAAGTATTACTAAAGGAGAAAGTCAAGTTGTTGCCGGAACAAATTATCGACTTGTAATTAATGCTA : 1336
BGV006775. : GGTTGCGATAGGACAATTTGCGATAAATGAACACAACAAAGAGGCAGGGACAAAATTGGAATTTCAAAGTATTACTAAAGGAGAAAGTCAAGTTGTTGCCGGAACAAATTATCGACTTGTAATTAATGCTA : 1336
             GGTTGCGATAGGACAATTTGCaATAAATGAACACAACAAAGAGGCAGGGACAAAATTGGAATTTCAAAGTATTACTAAAGGAGAAAGTCAAGTTGTTGCCGGAACAAATTATCGACTTGTAATTAATGCTA
                                          epm_Soly_M0_p1m06F_175 1483                                            epm_Soly_M0_p0m17R_172 1556
                 *      1460       *      1480       *      1500       *      1520       *      1540       *      1560       *
ITAG.1     : AAGATGGAGGCTATATTCGTAAATATTTGGTGGTTGTATGGGATACACCATGGGAGAAAATTAAGAACTTACTTCCTTCAAAAAAATGTAAGTGTTAAAAAGCTTATGGTTTATGATCTCGAGTTAGTGA : 1467
BGV006865. : AAGATGGAGGCTATATTCGTAAATATTTGGTGGTTGTATGGGATAAACCATGGGAGAAAATTAAGAACTTACTTCCTTCAAAAAAATGTAAGTGTTAAAAGG-TTATGGTTTATGATTTCGAGTTAGTGA : 1466
BGV007931. : AAGATGGAGGCTATATTCGTAAATATTTGGTGGTTGTATGGGATACACCATGGGAGAAAATTAAGAACTTACTTCCTTCAAAAAAATGTAAGTGTTAAAAAGCTTATGGTTTATGATCTCGAGTTAGTGA : 1467
BGV007989. : AAGATGGAGGCTATATTCGTAAATATTTGGTGGTTGTATGGGATACACCATGGGAGAAAATTAAGAACTTACTTCCTTCAAAAAAATGTAAGTGTTAAAAAGCTTATGGTTTATGATCTCGAGTTAGTGA : 1467
M82.1      : AAGATGGAGGCTATATTCGTAAATATTTGGTGGTTGTATGGGATACACCATGGGAGAAAATTAAGAACTTACTTCCTTCAAAAAAATGTAAGTGTTAAAAAGCTTATGGTTTATGATCTCGAGTTAGTGA : 1467
EA00371.1  : AAGATGGAGGCTATATTCGTAAATATTTGGTGGTTGTATGGGATACACCATGGGAGAAAATTAAGAACTTACTTCCTTCAAAAAAATGTAAGTGTTAAAAAGCTTATGGTTTATGATCTCGAGTTAGTGA : 1467
Fla.8924.1 : AAGATGGAGGCTATATTCGTAAATATTTGGTGGTTGTATGGGATACACCATGGGAGAAAATTAAGAACTTACTTCCTTCAAAAAAATGTAAGTGTTAAAAAGCTTATGGTTTATGATCTCGAGTTAGTGA : 1467
Floradade. : AAGATGGAGGCTATATTCGTAAATATTTGGTGGTTGTATGGGATACACCATGGGAGAAAATTAAGAACTTACTTCCTTCAAAAAAATGTAAGTGTTAAAAAGCTTATGGTTTATGATCTCGAGTTAGTGA : 1467
LYC1410.1  : AAGATGGAGGCTATATTCGTAAATATTTGGTGGTTGTATGGGATACACCATGGGAGAAAATTAAGAACTTACTTCCTTCAAAAAAATGTAAGTGTTAAAAAGCTTATGGTTTATGATCTCGAGTTAGTGA : 1467
PI169588.1 : AAGATGGAGGCTATATTCGTAAATATTTGGTGGTTGTATGGGATACACCATGGGAGAAAATTAAGAACTTACTTCCTTCAAAAAAATGTAAGTGTTAAAAAGCTTATGGTTTATGATCTCGAGTTAGTGA : 1467
```

```
Brandywine : AAGATGGAGGCTATATTCGTAAATATTTGGTGGTTGTATGGGATACACCATGGGAGAAAATTAAGAAACTTACTTCCTTCAAAAAAATGTAAGTGTTAAAAAGCTTATGGTTTATGATCTCGAGTTAGTGA : 1467
EA00990.1  : AAGATGGAGGCTATATTCGTAAATATTTGGTGGTTGTATGGGATACACCATGGGAGAAAATTAAGAAACTTACTTCCTTCAAAAAAATGTAAGTGTTAAAAAGCTTATGGTTTATGATCTCGAGTTAGTGA : 1467
PI303721.1 : AAGATGGAGGCTATATTCGTAAATATTTGGTGGTTGTATGGGATACACCATGGGAGAAAATTAAGAAACTTACTTCCTTCAAAAAAATGTAAGTGTTAAAAAGCTTATGGTTTATGATCTCGAGTTAGTGA : 1467
PAS014479. : AAGATGGAGGCTATATTCGTAAATATTTGGTGGTTGTATGGGATAAACCATGGGAGAAAATTAAGAAACTTACTTCCTTCAAAAAAATGTAAGTGTTAAAAGG-TTATGGTTTATGATTTCGAGTTAGTGA : 1466
BGV006775. : AAGATGGAGGCTATATTCGTAAATATTTGGTGGTTGTATGGGATAAACCATGGGAGAAAATTAAGAAACTTACTTCCTTCAAAAAAATGTAAGTGTTAAAAGG-TTATGGTTTATGATTTCGAGTTAGTGA : 1466
             AAGATGGAGGCTATATTCGTAAATATTTGGTGGTTGTATGGGATAcACCATGGGAGAAAATTAAGAAACTTACTTCCTTCAAAAAAATGTAAGTGTTAAAAaGcTTATGGTTTATGATcTCGAGTTAGTGA

                  1580         *     1600         *     1620         *     1640         *     1660         *     1680         *     1700
ITAG.1      : ATTTAGAATAAACGCGATGTTATAATATATAT-T                    -------------AAAGTATTACTAAA-----GGAGAA------------AGTCAAGTTG---------TTGCCGGA : 1558
BGV006865.  : ATTCAGAATAAACGCGATGTTATAATATATATCTnnnnnnnnnnnnnnnnnnnnAGTTGAAATCGCGGAATTCATCTTAAATCCACGGAGAAAATATGCTAACTAGTCAATTTGTAGGGTTATTAGTTTGA : 1597
BGV007931.  : ATTTAGAATAAACGCGATGTTATAATATATAT-Tnnnnnnnnnnnnnnnnnnnn-------------AAAGTATTACTAAA-----GGAGAA------------AGTCAAGTTG---------TTGCCGGA : 1558
BGV007989.  : ATTTAGAATAAACGCGATGTTATAATATATAT-Tnnnnnnnnnnnnnnnnnnnn-------------AAAGTATTACTAAA-----GGAGAA------------AGTCAAGTTG---------TTGCCGGA : 1558
M82.1       : ATTTAGAATAAACGCGATGTTATAATATATAT-Tnnnnnnnnnnnnnnnnnnnn-------------AAAGTATTACTAAA-----GGAGAA------------AGTCAAGTTG---------TTGCCGGA : 1558
EA00371.1   : ATTTAGAATAAACGCGATGTTATAATATATAT-Tnnnnnnnnnnnnnnnnnnnn-------------AAAGTATTACTAAA-----GGAGAA------------AGTCAAGTTG---------TTGCCGGA : 1558
Fla.8924.1  : ATTTAGAATAAACGCGATGTTATAATATATAT-Tnnnnnnnnnnnnnnnnnnnn-------------AAAGTATTACTAAA-----GGAGAA------------AGTCAAGTTG---------TTGCCGGA : 1558
Floradade.  : ATTTAGAATAAACGCGATGTTATAATATATAT-Tnnnnnnnnnnnnnnnnnnnn-------------AAAGTATTACTAAA-----GGAGAA------------AGTCAAGTTG---------TTGCCGGA : 1558
LYC1410.1   : ATTTAGAATAAACGCGATGTTATAATATATAT-Tnnnnnnnnnnnnnnnnnnnn-------------AAAGTATTACTAAA-----GGAGAA------------AGTCAAGTTG---------TTGCCGGA : 1558
PI169588.1  : ATTTAGAATAAACGCGATGTTATAATATATAT-Tnnnnnnnnnnnnnnnnnnnn-------------AAAGTATTACTAAA-----GGAGAA------------AGTCAAGTTG---------TTGCCGGA : 1558
Brandywine  : ATTTAGAATAAACGCGATGTTATAATATATAT-Tnnnnnnnnnnnnnnnnnnnn-------------AAAGTATTACTAAA-----GGAGAA------------AGTCAAGTTG---------TTGCCGGA : 1558
EA00990.1   : ATTTAGAATAAACGCGATGTTATAATATATAT-Tnnnnnnnnnnnnnnnnnnnn-------------AAAGTATTACTAAA-----GGAGAA------------AGTCAAGTTG---------TTGCCGGA : 1558
PI303721.1  : ATTTAGAATAAACGCGATGTTATAATATATAT-Tnnnnnnnnnnnnnnnnnnnn-------------AAAGTATTACTAAA-----GGAGAA------------AGTCAAGTTG---------TTGCCGGA : 1558
PAS014479.  : ATTCAGAATAAACGCGATGTTATAATATATATCTNNNNNNNNNNNNNNNNNNNNAGTTGAAATCGCGGAATTCATCTTAAATCCACGGAGAAAATATGCTAACTAGTCAATTTGTAGGGTTATTAGTTTGA : 1597
BGV006775.  : ATTCAGAATAAACGCGATGTTATAATATATATCTNNNNNNNNNNNNNNNNNNNNAGTTGAAATCGCGGAATTCATCTTAAATCCACGGAGAAAATATGCTAACTAGTCAATTTGTAGGGTTATTAGTTTGA : 1597
              ATTtAGAATAAACGCGATGTTATAATATATAT TNNNNNNNNNNNNNNNNNNNN             aAAgTatTacTAAA     GGAGAA            AGTCAAgTTG         TtGccgGA
                                                                           epm_Soly_M0_p1m06F_175 1777
                      *     1720         *     1740         *     1760         *     1780         *     1800         *     1820         *
ITAG.1      : ACAAATTATCGACTTGTAATTAATGCTAAAGATGGAGGCTA---TATTCGTAAATATTTGGTGG-TTGTATGGGATACACCATGGGAGAAAATTAAGAAACTTACTTCCTTCAAAAAAATGTAAGTGTTAA : 1685
BGV006865.  : ATAACATGGTGTTTGTTAATCTCTTTTCTAAATCGACATTGAGTCATTTGAACGTTATTGTTGACTTGTCAATGACTCAACAT-----AAAATTAGCTAATTTA---TATTGAATATAATCTTCATATTAC : 1720
BGV007931.  : ACAAATTATCGACTTGTAATTAATGCTAAAGATGGAGGCTA---TATTCGTAAATATTTGGTGG-TTGTATGGGATACACCATGGGAGAAAATTAAGAAACTTACTTCCTTCAAAAAAATGTAAGTGTTAA : 1685
BGV007989.  : ACAAATTATCGACTTGTAATTAATGCTAAAGATGGAGGCTA---TATTCGTAAATATTTGGTGG-TTGTATGGGATACACCATGGGAGAAAATTAAGAAACTTACTTCCTTCAAAAAAATGTAAGTGTTAA : 1685
M82.1       : ACAAATTATCGACTTGTAATTAATGCTAAAGATGGAGGCTA---TATTCGTAAATATTTGGTGG-TTGTATGGGATACACCATGGGAGAAAATTAAGAAACTTACTTCCTTCAAAAAAATGTAAGTGTTAA : 1685
EA00371.1   : ACAAATTATCGACTTGTAATTAATGCTAAAGATGGAGGCTA---TATTCGTAAATATTTGGTGG-TTGTATGGGATACACCATGGGAGAAAATTAAGAAACTTACTTCCTTCAAAAAAATGTAAGTGTTAA : 1685
Fla.8924.1  : ACAAATTATCGACTTGTAATTAATGCTAAAGATGGAGGCTA---TATTCGTAAATATTTGGTGG-TTGTATGGGATACACCATGGGAGAAAATTAAGAAACTTACTTCCTTCAAAAAAATGTAAGTGTTAA : 1685
Floradade.  : ACAAATTATCGACTTGTAATTAATGCTAAAGATGGAGGCTA---TATTCGTAAATATTTGGTGG-TTGTATGGGATACACCATGGGAGAAAATTAAGAAACTTACTTCCTTCAAAAAAATGTAAGTGTTAA : 1685
LYC1410.1   : ACAAATTATCGACTTGTAATTAATGCTAAAGATGGAGGCTA---TATTCGTAAATATTTGGTGG-TTGTATGGGATACACCATGGGAGAAAATTAAGAAACTTACTTCCTTCAAAAAAATGTAAGTGTTAA : 1685
PI169588.1  : ACAAATTATCGACTTGTAATTAATGCTAAAGATGGAGGCTA---TATTCGTAAATATTTGGTGG-TTGTATGGGATACACCATGGGAGAAAATTAAGAAACTTACTTCCTTCAAAAAAATGTAAGTGTTAA : 1685
Brandywine  : ACAAATTATCGACTTGTAATTAATGCTAAAGATGGAGGCTA---TATTCGTAAATATTTGGTGG-TTGTATGGGATACACCATGGGAGAAAATTAAGAAACTTACTTCCTTCAAAAAAATGTAAGTGTTAA : 1685
EA00990.1   : ACAAATTATCGACTTGTAATTAATGCTAAAGATGGAGGCTA---TATTCGTAAATATTTGGTGG-TTGTATGGGATACACCATGGGAGAAAATTAAGAAACTTACTTCCTTCAAAAAAATGTAAGTGTTAA : 1685
PI303721.1  : ACAAATTATCGACTTGTAATTAATGCTAAAGATGGAGGCTA---TATTCGTAAATATTTGGTGG-TTGTATGGGATACACCATGGGAGAAAATTAAGAAACTTACTTCCTTCAAAAAAATGTAAGTGTTAA : 1685
PAS014479.  : ATAACATGGTGTTTGTTAATCTCTTTTCTAAATCGACATTGAGTCATTTGAACGTTATTGTTGACTTGTCAATGACTCAACAT-----AAAATTAGCTAATTTA---TATTGAATATAATCTTCATATTAC : 1720
BGV006775.  : ATAACATGGTGTTTGTTAATCTCTTTTCTAAATCGACATTGAGTCATTTGAACGTTATTGTTGACTTGTCAATGACTCAACAT-----AAAATTAGCTAATTTA---TATTGAATATAATCTTCATATTAC : 1720
              AcAAatTatcGacTtgTAATtaaTgcTaaAgATgGAggcTa   tATTcGtAaatTatTTGgTGg TTGTatggGAtaCAcCATgggagAAAATTaagaAAcTTActtccTTcAAaAaAATgTaagTgTTAa


                                                                                                   epm_Soly_M0_p0m15R_209 1924

                                                                                                   epm_Soly_M0_p0m08R_491 1924

                                                                                                   epm_Soly_M0_p0m13R_248 1924

                                                                                                          epm_Soly_M0_p0m08R_491 1928

                                                                                                            epm_Soly_M0_p0m13R_248 1930
                1840         *     1860         *     1880         *     1900         *     1920         *     1940         *     1960
ITAG.1      : AAAGCTTATG------GTTTATGATCTCG--AGTTAGTGAATTTAGAATAAACGCGATGTTATAATATATATTTATGATATGTTTTAATGTTTTTGTATGTGTATGTATC--GTTTGAGATATGAATTTGA : 1806
BGV006865.  : GTAGTATATATTATTTGTATATGATATTGTTAGTTCGTAAA---AATATAAAAATTGATCCAAAACTTAAAACTATGAAGTACTTTAAAA--TGAGTAGGTTGATGTATTAAGCTTTTGCTATG------- : 1839
BGV007931.  : AAAGCTTATG------GTTTATGATCTCG--AGTTAGTGAATTTAGAATAAACGCGATGTTATAATATATATTTATGGTATGTTTTAATGTTTTTGTATGTGTATGTATC--GTTTGAGATATGAATTTGA : 1806
BGV007989.  : AAAGCTTATG------GTTTATGATCTCG--AGTTAGTGAATTTAGAATAAACGCGATGTTATAATATATATTTATGATATGTTTTAATGTTTTTGTATGTGTATGTATC--GTTTGAGATATGAATTTGA : 1806
M82.1       : AAAGCTTATG------GTTTATGATCTCG--AGTTAGTGAATTTAGAATAAACGCGATGTTATAATATATATTTATGATATGTTTTAATGTTTTTGTATGTGTATGTATC--GTTTGAGATATGAATTTGA : 1806
EA00371.1   : AAAGCTTATG------GTTTATGATCTCG--AGTTAGTGAATTTAGAATAAACGCGATGTTATAATATATATTTATGATATGTTTTAATGTTTTTGTATGTGTATGTATC--GTTTGAGATATGAATTTGA : 1806
Fla.8924.1  : AAAGCTTATG------GTTTATGATCTCG--AGTTAGTGAATTTAGAATAAACGCGATGTTATAATATATATTTATGATATGTTTTAATGTTTTTGTATGTGTATGTATC--GTTTGAGATATGAATTTGA : 1806
Floradade.  : AAAGCTTATG------GTTTATGATCTCG--AGTTAGTGAATTTAGAATAAACGCGATGTTATAATATATATTTATGATATGTTTTAATGTTTTTGTATGTGTATGTATC--GTTTGAGATATGAATTTGA : 1806
LYC1410.1   : AAAGCTTATG------GTTTATGATCTCG--AGTTAGTGAATTTAGAATAAACGCGATGTTATAATATATATTTATGATATGTTTTAATGTTTTTGTATGTGTATGTATC--GTTTGAGATATGAATTTGA : 1806
PI169588.1  : AAAGCTTATG------GTTTATGATCTCG--AGTTAGTGAATTTAGAATAAACGCGATGTTATAATATATATTTATGATATGTTTTAATGTTTTTGTATGTGTATGTATC--GTTTGAGATATGAATTTGA : 1806
Brandywine  : AAAGCTTATG------GTTTATGATCTCG--AGTTAGTGAATTTAGAATAAACGCGATGTTATAATATATATTTATGATATGTTTTAATGTTTTTGTATGTGTATGTATC--GTTTGAGATATGAATTTGA : 1806
EA00990.1   : AAAGCTTATG------GTTTATGATCTCG--AGTTAGTGAATTTAGAATAAACGCGATGTTATAATATATATTTATGATATGTTTTAATGTTTTTGTATGTGTATGTATC--GTTTGAGATATGAATTTGA : 1806
PI303721.1  : AAAGCTTATG------GTTTATGATCTCG--AGTTAGTGAATTTAGAATAAACGCGATGTTATAATATATATTTATGATATGTTTTAATGTTTTTGTATGTGTATGTATC--GTTTGAGATATGAATTTGA : 1806
PAS014479.  : GTAGTATATATTATTTGTATATGATATTGTTAGTTCGTAAA---AATATAAAAATTGATCCAAAACTTAAAACTATGAAGTACTTTAAAA--TGAGTAGGTTGATGTATTAAGCTTTTGCTATG------- : 1839
BGV006775.  : GTAGTATATATTATTTGTATATGATATTGTTAGTTCGTAAA---AATATAAAAATTGATCCAAAACTTAAAACTATGAAGTACTTTAAAA--TGAGTAGGTTGATGTATTAAGCTTTTGCTATG------- : 1839
              aaAGctTATg      GTtTATGATcTcG  AGTTaGTgAAtttAgaATAAAcgcgatgttAtAAtaTAtAttTATGataTgtTTTAAtgttTttGTAtGTgtATGTATc  GtTTgaGaTATGaatttga
                                  epm_Soly_M0_p0m14R_211 1994                                 epm_Soly_M0_p0m13R_248 2063

                                  epm_Soly_M0_p0m17R_172 1994                                 epm_Soly_M0_p0m08R_491 2063

                                                                                                       epm_Soly_M0_p0m08R_491 2067

                                                                                                         epm_Soly_M0_p0m13R_248 2069

                                                                                                           epm_Soly_M0_p0m00R_5096 2072

                                                                                                           epm_Soly_M0_p1m00R_9780 2072
                    *     1980         *     2000         *     2020         *     2040         *     2060         *     2080         *
ITAG.1      : TTGTAAATTGAGAAAATTT--------AAGATCTTGAGTTAGTAGATTCGGAATAAATCTGATTTTATTTGTATATATGTATATATTTTGAGATATGTATTTTGTATGTGTATGTATTGTTTGAGATATGT : 1929
BGV006865.  : -TGCGAGATTTGGAAATTTAGTCCACAAAGATCTTTTGTTCGTAAGCTTATCATA----------TATTTTTATATAAGCTTATTTT--------CACATTTTAAATT------------------TATGA : 1933
BGV007931.  : TTGTAAATTGAGAAAATTT--------AAGATTTTGAGTTAGTAGATTCGGAATAAACCTGATTTTATTTGTATATATGTATATATTTTGAGATATGTATTTTGTATGTGTATGTATTGTTTGAGATATGT : 1929
BGV007989.  : TTGTAAATTGAGAAAATTT--------AAGATCTTGAGTTAGTAGATTCGGAATAAATCTGATTTTATTTGTATATATGTATATATTTTGAGATATGTATTTTGTATGTGTATGTATTGTTTGAGATATGT : 1929
M82.1       : TTGTAAATTGAGAAAATTT--------AAGATCTTGAGTTAGTAGATTCGGAATAAATCTGATTTTATTTGTATATATGTATATATTTTGAGATATGTATTTTGTATGTGTATGTATTGTTTGAGATATGT : 1929
EA00371.1   : TTGTAAATTGAGAAAATTT--------AAGATCTTGAGTTAGTAGATTCGGAATAAATCTGATTTTATTTGTATATATGTATATATTTTGAGATATGTATTTTGTATGTGTATGTATTGTTTGAGATATGT : 1929
Fla.8924.1  : TTGTAAATTGAGAAAATTT--------AAGATCTTGAGTTAGTAGATTCGGAATAAATCTGATTTTATTTGTATATATGTATATATTTTGAGATATGTATTTTGTATGTGTATGTATTGTTTGAGATATGT : 1929
Floradade.  : TTGTAAATTGAGAAAATTT--------AAGATCTTGAGTTAGTAGATTCGGAATAAATCTGATTTTATTTGTATATATGTATATATTTTGAGATATGTATTTTGTATGTGTATGTATTGTTTGAGATATGT : 1929
LYC1410.1   : TTGTAAATTGAGAAAATTT--------AAGATCTTGAGTTAGTAGATTCGGAATAAATCTGATTTTATTTGTATATATGTATATATTTTGAGATATGTATTTTGTATGTGTATGTATTGTTTGAGATATGT : 1929
PI169588.1  : TTGTAAATTGAGAAAATTT--------AAGATCTTGAGTTAGTAGATTCGGAATAAATCTGATTTTATTTGTATATATGTATATATTTTGAGATATGTATTTTGTATGTGTATGTATTGTTTGAGATATGT : 1929
Brandywine  : TTGTAAATTGAGAAAATTT--------AAGATCTTGAGTTAGTAGATTCGGAATAAATCTGATTTTATTTGTATATATGTATATATTTTGAGATATGTATTTTGTATGTGTATGTATTGTTTGAGATATGT : 1929
EA00990.1   : TTGTAAATTGAGAAAATTT--------AAGATCTTGAGTTAGTAGATTCGGAATAAATCTGATTTTATTTGTATATATGTATATATTTTGAGATATGTATTTTGTATGTGTATGTATTGTTTGAGATATGT : 1929
PI303721.1  : TTGTAAATTGAGAAAATTT--------AAGATCTTGAGTTAGTAGATTCGGAATAAATCTGATTTTATTTGTATATATGTATATATTTTGAGATATGTATTTTGTATGTGTATGTATTGTTTGAGATATGT : 1929
PAS014479.  : -TGCGAGATTTGGAAATTTAGTCCACAAAGATCTGTTGTTCGTAAGCTTATCATA----------TATTTTTATATAAGCTTATTTT--------CACATTTTAAATT------------------TATGA : 1933
BGV006775.  : -TGCGAGATTTGGAAATTTAGTCCACAAAGATCTTTTGTTCGTAAGCTTATCATA----------TATTTTTATATAAGCTTATTTT--------CACATTTTAAATT------------------TATGA : 1933
              tTGtaAatTgaGaAAATTT        AAGATcTtgaGTTaGTAgatTcggaATAaa ctgatttTATTTgTATATAtGtaTATaTTtgagatatgtATTTTgtATgtgtatgtattgtttgagaTATGt

              2100         *     2120         *     2140         *     2160         *     2180         *     2200         *     2220
ITAG.1      : ATTTGGTTGTAAATTAGGAAATTTTAAGATCTTTATTCAATTATGTTAAGACTTTCTCTATATATGAAACGCTCTAGAAAATAAGGATTTTAATATAAATAGAAGTATCGCAAATTTAGCTAGCGAAAAAA : 2060
BGV006865.  : CCTTATAATTAACTTCGTAAA----AATAACTTTATTCAATTATGTTAAGACTTTCTCTATATATGAAACGCTCTAGAAAATAAGGATTTTAATATAAATAGAAGTATCGCAAATTTAGCTAGCGAAAAAA : 2060
BGV007931.  : ATTTGGTTGTAAATTAGGAAATTTTAAGATCTTTATTCAATTATGTTAAGACTTTCTCTATATATGAAACGCTCTAGAAAATAAGGATTTTAATATAAATAGAAGTATCGCAAATTTAGCTAGCGAAAAAA : 2060
BGV007989.  : ATTTGGTTGTAAATTAGGAAATTTTAAGATCTTTATTCAATTATGTTAAGACTTTCTCTATATATGAAACGCTCTAGAAAATAAGGATTTTAATATAAATAGAAGTATCGCAAATTTAGCTAGCGAAAAAA : 2060
M82.1       : ATTTGGTTGTAAATTAGGAAATTTTAAGATCTTTATTCAATTATGTTAAGACTTTCTCTATATATGAAACGCTCTAGAAAATAAGGATTTTAATATAAATAGAAGTATCGCAAATTTAGCTAGCGAAAAAA : 2060
EA00371.1   : ATTTGGTTGTAAATTAGGAAATTTTAAGATCTTTATTCAATTATGTTAAGACTTTCTCTATATATGAAACGCTCTAGAAAATAAGGATTTTAATATAAATAGAAGTATCGCAAATTTAGCTAGCGAAAAAA : 2060
Fla.8924.1  : ATTTGGTTGTAAATTAGGAAATTTTAAGATCTTTATTCAATTATGTTAAGACTTTCTCTATATATGAAACGCTCTAGAAAATAAGGATTTTAATATAAATAGAAGTATCGCAAATTTAGCTAGCGAAAAAA : 2060
Floradade.  : ATTTGGTTGTAAATTAGGAAATTTTAAGATCTTTATTCAATTATGTTAAGACTTTCTCTATATATGAAACGCTCTAGAAAATAAGGATTTTAATATAAATAGAAGTATCGCAAATTTAGCTAGCGAAAAAA : 2060
LYC1410.1   : ATTTGGTTGTAAATTAGGAAATTTTAAGATCTTTATTCAATTATGTTAAGACTTTCTCTATATATGAAACGCTCTAGAAAATAAGGATTTTAATATAAATAGAAGTATCGCAAATTTAGCTAGCGAAAAAA : 2060
PI169588.1  : ATTTGGTTGTAAATTAGGAAATTTTAAGATCTTTATTCAATTATGTTAAGACTTTCTCTATATATGAAACGCTCTAGAAAATAAGGATTTTAATATAAATAGAAGTATCGCAAATTTAGCTAGCGAAAAAA : 2060
Brandywine  : ATTTGGTTGTAAATTAGGAAATTTTAAGATCTTTATTCAATTATGTTAAGACTTTCTCTATATATGAAACGCTCTAGAAAATAAGGATTTTAATATAAATAGAAGTATCGCAAATTTAGCTAGCGAAAAAA : 2060
EA00990.1   : ATTTGGTTGTAAATTAGGAAATTTTAAGATCTTTATTCAATTATGTTAAGACTTTCTCTATATATGAAACGCTCTAGAAAATAAGGATTTTAATATAAATAGAAGTATCGCAAATTTAGCTAGCGAAAAAA : 2060
PI303721.1  : ATTTGGTTGTAAATTAGGAAATTTTAAGATCTTTATTCAATTATGTTAAGACTTTCTCTATATATGAAACGCTCTAGAAAATAAGGATTTTAATATAAATAGAAGTATCGCAAATTTAGCTAGCGAAAAAA : 2060
PAS014479.  : CCTTATAATTAACTTCGTAAA----AATAACTTTATTCAATTATGTTAAGACTTTCTCTATATATGAAACGCTCTAGAAAATAAGGATTTTAATATAAATAGAAGTATCGCAAATTTAGCTAGCGAAAAAA : 2060
BGV006775.  : CCTTATAATTAACTTCGTAAA----AATAACTTTATTCAATTATGTTAAGACTTTCTCTATATATGAAACGCTCTAGAAAATAAGGATTTTAATATAAATAGAAGTATCGCAAATTTAGCTAGCGAAAAAA : 2060
              atTTggttgTAAaTTaGgAAAttttAAgAtCTTTATTCAATTATGTTAAGACTTTCTCTATATATGAAACGCTCTAGAAAATAAGGATTTTAATATAAATAGAAGTATCGCAAATTTAGCTAGCGAAAAAA

                *     2240         *     2260         *     2280         *     2300         *     2320         *     2340         *     23
ITAG.1      : GAAAAGGTTTATGATTTAAGAAAAACACATATAAATTATTTTTTTTCTCTTGAGAAAATCATACCCTAGCTATCTAGTATTCCTTTTTCCTATCTAAATTATCATCTTCTTTGTATTAAAATATATCTTGA : 2191
```

```
BGV006865. : GAAAAGGTTTATGATTTAAAAAAAACACATATAAATTATTTTTTTTCTCTTGAGAAAATCATACCCTAGCTATCTAGTATTCCTTTTTCCTATCTAAATTATTATCTTCTTTGTATTAAAATATATCTTGA : 2191
BGV007931. : GAAAAGGTTTATGATTTAAGAAAAACACATATAAATTATTTTTTTCTCTCTTGAGAAAATCATACCCTAGCTATCTAGTATTCCTTTTTCCTATCTAAATTATCATCTTCTTTGTATTAAAATATATCTTGA : 2191
BGV007989. : GAAAAGGTTTATGATTTAAGAAAAACACATATAAATTATTTTTTTTCTCTTGAGAAAATCATACCCTAGCTATCTAGTATTCCTTTTTCCTATCTAAATTATCATCTTCTTTGTATTAAAATATATCTTGA : 2191
M82.1      : GAAAAGGTTTATGATTTAAGAAAAACACATATAAATTATTTTTTTTCTCTTGAGAAAATCATACCCTAGCTATCTAGTATTCCTTTTTCCTATCTAAATTATCATCTTCTTTGTATTAAAATATATCTTGA : 2191
EA00371.1  : GAAAAGGTTTATGATTTAAGAAAAACACATATAAATTATTTTTTTTCTCTTGAGAAAATCATACCCTAGCTATCTAGTATTCCTTTTTCCTATCTAAATTATCATCTTCTTTGTATTAAAATATATCTTGA : 2191
Fla.8924.1 : GAAAAGGTTTATGATTTAAGAAAAACACATATAAATTATTTTTTTTCTCTTGAGAAAATCATACCCTAGCTATCTAGTATTCCTTTTTCCTATCTAAATTATCATCTTCTTTGTATTAAAATATATCTTGA : 2191
Floradade. : GAAAAGGTTTATGATTTAAGAAAAACACATATAAATTATTTTTTTTCTCTTGAGAAAATCATACCCTAGCTATCTAGTATTCCTTTTTCCTATCTAAATTATCATCTTCTTTGTATTAAAATATATCTTGA : 2191
LYC1410.1  : GAAAAGGTTTATGATTTAAGAAAAACACATATAAATTATTTTTTTTCTCTTGAGAAAATCATACCCTAGCTATCTAGTATTCCTTTTTCCTATCTAAATTATCATCTTCTTTGTATTAAAATATATCTTGA : 2191
PI169588.1 : GAAAAGGTTTATGATTTAAGAAAAACACATATAAATTATTTTTTTTCTCTTGAGAAAATCATACCCTAGCTATCTAGTATTCCTTTTTCCTATCTAAATTATCATCTTCTTTGTATTAAAATATATCTTGA : 2191
Brandywine : GAAAAGGTTTATGATTTAAGAAAAACACATATAAATTATTTTTTTTCTCTTGAGAAAATCATACCCTAGCTATCTAGTATTCCTTTTTCCTATCTAAATTATCATCTTCTTTGTATTAAAATATATCTTGA : 2191
EA00990.1  : GAAAAGGTTTATGATTTAAGAAAAACACATATAAATTATTTTTTTTCTCTTGAGAAAATCATACCCTAGCTATCTAGTATTCCTTTTTCCTATCTAAATTATCATCTTCTTTGTATTAAAATATATCTTGA : 2191
PI303721.1 : GAAAAGGTTTATGATTTAAGAAAAACACATATAAATTATTTTTTTTCTCTTGAGAAAATCATACCCTAGCTATCTAGTATTCCTTTTTCCTATCTAAATTATCATCTTCTTTGTATTAAAATATATCTTGA : 2191
PAS014479. : GAAAAGGTTTATGCTTTAAAAAAAACACATATAAATTATTTTTTTTCTCTTGAGAAAATCATACCCTAGCTATCTAGTATTCCTTTTTCCTATCTAAATTATCATCTTCTTTGTATTAAAATATATCTTGA : 2191
BGV006775. : GAAAAGGTTTATGATTTAAAAAAAACACATATAAATTATTTTTTTTCTCTTGAGAAAATCATACCCTAGCTATCTAGTATTCCTTTTTCCTATCTAAATTATCATCTTCTTTGTATTAAAATATATCTTGA : 2191
             GAAAAGGTTTATGaTTTAAgAAAAACACATATAAATTATTTTTTTtTCTCTTGAGAAAATCATACCCTAGCTATCTAGTATTCCTTTTTCCTATCTAAATTATcATCTTCTTTGTATTAAAATATATCTTGA
                                         epm_Soly_M0_p1m02F_1369 2397
             60         *     2380         *     2400         *     2420         *     2440         *     2460         *     2480
ITAG.1     : CACATGTAGATCAACTCAATATTGAGGTGTGTTTTAATT-AAAAAAAAAAAAGAACGAGTGATAGTTTAAATCGATGAATGAAACAATGGATGGTTGAAAAATAAAATCTACAAAAATGTGATAATTTAGA : 2321
BGV006865. : CACATGTAGATCAACTCAATATTGAGGTGTGTTTTAATT--AAAAAAAAAAAGAACGAGTGATAGTTTAAATCGATGAATGAAACAATGGATGGTTGAAAAATAAAATCTATAAAAATGTGATAATTTAGA : 2320
BGV007931. : CACATGTAGATCAACTCAATATTGAGGTGTGTTTTAATTAAAAAAAAAAAAAGAACGAGTGATAGTTTAAATCGATGAATGAAACAATGGATGGTTGAAAAATAAAATCTACAAAAATGTGATAATTTAGA : 2322
BGV007989. : CACATGTAGATCAACTCAATATTGAGGTGTGTTTTAATTAAAAAAAAAAAAAGAACGAGTGATAGTTTAAATCGATGAATGAAACAATGGATGGTTGAAAAATAAAATCTACAAAAATGTGATAATTTAGA : 2322
M82.1      : CACATGTAGATCAACTCAATATTGAGGTGTGTTTTAATTAAAAAAAAAAAAAGAACGAGTGATAGTTTAAATCGATGAATGAAACAATGGATGGTTGAAAAATAAAATCTACAAAAATGTGATAATTTAGA : 2322
EA00371.1  : CACATGTAGATCAACTCAATATTGAGGTGTGTTTTAATTAAAAAAAAAAAAAGAACGAGTGATAGTTTAAATCGATGAATGAAACAATGGATGGTTGAAAAATAAAATCTACAAAAATGTGATAATTTAGA : 2322
Fla.8924.1 : CACATGTAGATCAACTCAATATTGAGGTGTGTTTTAATTAAAAAAAAAAAAAGAACGAGTGATAGTTTAAATCGATGAATGAAACAATGGATGGTTGAAAAATAAAATCTACAAAAATGTGATAATTTAGA : 2322
Floradade. : CACATGTAGATCAACTCAATATTGAGGTGTGTTTTAATTAAAAAAAAAAAAAGAACGAGTGATAGTTTAAATCGATGAATGAAACAATGGATGGTTGAAAAATAAAATCTACAAAAATGTGATAATTTAGA : 2322
LYC1410.1  : CACATGTAGATCAACTCAATATTGAGGTGTGTTTTAATTAAAAAAAAAAAAAGAACGAGTGATAGTTTAAATCGATGAATGAAACAATGGATGGTTGAAAAATAAAATCTACAAAAATGTGATAATTTAGA : 2322
PI169588.1 : CACATGTAGATCAACTCAATATTGAGGTGTGTTTTAATTAAAAAAAAAAAAAGAACGAGTGATAGTTTAAATCGATGAATGAAACAATGGATGGTTGAAAAATAAAATCTACAAAAATGTGATAATTTAGA : 2322
Brandywine : CACATGTAGATCAACTCAATATTGAGGTGTGTTTTAATTAAAAAAAAAAAAAGAACGAGTGATAGTTTAAATCGATGAATGAAACAATGGATGGTTGAAAAATAAAATCTACAAAAATGTGATAATTTAGA : 2322
EA00990.1  : CACATGTAGATCAACTCAATATTGAGGTGTGTTTTAATTAAAAAAAAAAAAAGAACGAGTGATAGTTTAAATCGATGAATGAAACAATGGATGGTTGAAAAATAAAATCTACAAAAATGTGATAATTTAGA : 2322
PI303721.1 : CACATGTAGATCAACTCAATATTGAGGTGTGTTTTAATTAAAAAAAAAAAAAGAACGAGTGATAGTTTAAATCGATGAATGAAACAATGGATGGTTGAAAAATAAAATCTACAAAAATGTGATAATTTAGA : 2322
PAS014479. : CACATGTAGATCAACTCAATATTGAGGTGTGTTTTAATT---AAAAAAAAAAGAACGAGTGATAGTTTAAATCGATGAATGAAACAATGGATGGTTGAAAAATAAAATCTACAAAAATGTGATAATTTAGA : 2319
BGV006775. : CACATGTAGATCAACTCAATATTGAGGTGTGTTTTAATT--AAAAAAAAAAAGAACGAGTGATAGTTTAAATCGATGAATGAAACAATGGATGGTTGAAAAATAAAATCTACAAAAATGTGATAATTTAGA : 2320
             CACATGTAGATCAACTCAATATTGAGGTGTGTTTTAATT aaAAAAAAAAAAGAACGAGTGATAGTTTAAATCGATGAATGAAACAATGGATGGTTGAAAAATAAAATCTAcAAAAATGTGATAATTTAGA

             *      2500         *     2520         *     2540         *     2560         *     2580         *     2600         *     2620
ITAG.1     : TATATATTTTACGATCAACTCTG-AAAAAGAAAGTATTGAAGTGAGATTAAATGAGCCTATGATATCCAGAAAAGCAAGTTCTATAAAAATAGTGTTTATAAGATCAATAATATCAATTTTATGAAATGGA : 2451
BGV006865. : TATATATTTTACGATCAACTCTGAAAAAAGAAAGTATTGAAGTGAGATTAAATGAGCCTATGATATCCAGAAAAGCAAGTTCTATAAAAATAGTGTTTATAAGATCAATAATGTCAATTTTATGAAATGAA : 2451
BGV007931. : TATATATTTTACGATCAACTCTG-AAAAAGAAAGTATTGAAGTGAGATTAAATGAGCCTATGATATCCAGAAAAGCAAGTTCTATAAAAATAGTGTTTATAAGATCAATAATATCAATTTTATGAAATGGA : 2452
BGV007989. : TATATATTTTACGATCAACTCTG-AAAAAGAAAGTATTGAAGTGAGATTAAATGAGCCTATGATATCCAGAAAAGCAAGTTCTATAAAAATAGTGTTTATAAGATCAATAATATCAATTTTATGAAATGGA : 2452
M82.1      : TATATATTTTACGATCAACTCTG-AAAAAGAAAGTATTGAAGTGAGATTAAATGAGCCTATGATATCCAGAAAAGCAAGTTCTATAAAAATAGTGTTTATAAGATCAATAATATCAATTTTATGAAATGGA : 2452
EA00371.1  : TATATATTTTACGATCAACTCTG-AAAAAGAAAGTATTGAAGTGAGATTAAATGAGCCTATGATATCCAGAAAAGCAAGTTCTATAAAAATAGTGTTTATAAGATCAATAATATCAATTTTATGAAATGGA : 2452
Fla.8924.1 : TATATATTTTACGATCAACTCTG-AAAAAGAAAGTATTGAAGTGAGATTAAATGAGCCTATGATATCCAGAAAAGCAAGTTCTATAAAAATAGTGTTTATAAGATCAATAATATCAATTTTATGAAATGGA : 2452
Floradade. : TATATATTTTACGATCAACTCTG-AAAAAGAAAGTATTGAAGTGAGATTAAATGAGCCTATGATATCCAGAAAAGCAAGTTCTATAAAAATAGTGTTTATAAGATCAATAATATCAATTTTATGAAATGGA : 2452
LYC1410.1  : TATATATTTTACGATCAACTCTG-AAAAAGAAAGTATTGAAGTGAGATTAAATGAGCCTATGATATCCAGAAAAGCAAGTTCTATAAAAATAGTGTTTATAAGATCAATAATATCAATTTTATGAAATGGA : 2452
PI169588.1 : TATATATTTTACGATCAACTCTG-AAAAAGAAAGTATTGAAGTGAGATTAAATGAGCCTATGATATCCAGAAAAGCAAGTTCTATAAAAATAGTGTTTATAAGATCAATAATATCAATTTTATGAAATGGA : 2452
Brandywine : TATATATTTTACGATCAACTCTG-AAAAAGAAAGTATTGAAGTGAGATTAAATGAGCCTATGATATCCAGAAAAGCAAGTTCTATAAAAATAGTGTTTATAAGATCAATAATATCAATTTTATGAAATGGA : 2452
EA00990.1  : TATATATTTTACGATCAACTCTG-AAAAAGAAAGTATTGAAGTGAGATTAAATGAGCCTATGATATCCAGAAAAGCAAGTTCTATAAAAATAGTGTTTATAAGATCAATAATATCAATTTTATGAAATGGA : 2452
PI303721.1 : TATATATTTTACGATCAACTCTG-AAAAAGAAAGTATTGAAGTGAGATTAAATGAGCCTATGATATCCAGAAAAGCAAGTTCTATAAAAATAGTGTTTATAAGATCAATAATATCAATTTTATGAAATGGA : 2452
PAS014479. : TATATATTTTACGATCAACTCTG-AAAAAGAAAGTATTGAAGTGAGATTAAATGAGCCTTTGATATCCAGAAAAGCAAGTTCTATAAAAATAGTGTTTATAAGATCAATAATGTCAATTTTATGAAATGGA : 2449
BGV006775. : TATATATTTTACGATCAACTCTGAAAAAAGAAAGTATTGAAGTGAGATTAAATGAGCCTATGATATCCAGAAAAGCAAGTTCTATAAAAATAGTGTTTATAAGATCAATAATGTCAATTTTATGAAATGAA : 2451
             TATATATTTTACGATCAACTCTG AAAAAGAAAGTATTGAAGTGAGATTAAATGAGCCTaTGATATCCAGAAAAGCAAGTTCTATAAAAATAGTGTTTATAAGATCAATAATaTCAATTTTATGAAATGgA

                   *      2640         *     2660         *     2680         *     2700         *     2720         *     2740         *
ITAG.1     : AATAAGTCGAATAACGTAAAGTACAAATTTTTTTCATCTTATTGCATTATTGATTAGAGAGGACAAATATTTCGTAAAATTAATCAAGATACATGTAGACCTGAACAACACCATTGATAATTATACAAAAA : 2582
BGV006865. : AATAAGTCGAATAACGTAAAGTACAAATTTTTTTCATCTTATTGCATTATTGATTAGAGAGGACAAATATTTCGTAAAATTAATCAAGATACATGTAGACCTGAACAACACCATTGATAATTATACAAAAA : 2582
BGV007931. : AATAAGTCGAATAACGTAAAGTACAAATTTTTTTCATCTTATTGCATTATTGATTAGAGAGGACAAATATTTCGTAAAATTAATCAAGATACATGTAGACCTGAACAACACCATTGATAATTATACAAAAA : 2583
BGV007989. : AATAAGTCGAATAACGTAAAGTACAAATTTTTTTCATCTTATTGCATTATTGATTAGAGAGGACAAATATTTCGTAAAATTAATCAAGATACATGTAGACCTGAACAACACCATTGATAATTATACAAAAA : 2583
M82.1      : AATAAGTCGAATAACGTAAAGTACAAATTTTTTTCATCTTATTGCATTATTGATTAGAGAGGACAAATATTTCGTAAAATTAATCAAGATACATGTAGACCTGAACAACACCATTGATAATTATACAAAAA : 2583
EA00371.1  : AATAAGTCGAATAACGTAAAGTACAAATTTTTTTCATCTTATTGCATTATTGATTAGAGAGGACAAATATTTCGTAAAATTAATCAAGATACATGTAGACCTGAACAACACCATTGATAATTATACAAAAA : 2583
Fla.8924.1 : AATAAGTCGAATAACGTAAAGTACAAATTTTTTTCATCTTATTGCATTATTGATTAGAGAGGACAAATATTTCGTAAAATTAATCAAGATACATGTAGACCTGAACAACACCATTGATAATTATACAAAAA : 2583
Floradade. : AATAAGTCGAATAACGTAAAGTACAAATTTTTTTCATCTTATTGCATTATTGATTAGAGAGGACAAATATTTCGTAAAATTAATCAAGATACATGTAGACCTGAACAACACCATTGATAATTATACAAAAA : 2583
LYC1410.1  : AATAAGTCGAATAACGTAAAGTACAAATTTTTTTCATCTTATTGCATTATTGATTAGAGAGGACAAATATTTCGTAAAATTAATCAAGATACATGTAGACCTGAACAACACCATTGATAATTATACAAAAA : 2583
PI169588.1 : AATAAGTCGAATAACGTAAAGTACAAATTTTTTTCATCTTATTGCATTATTGATTAGAGAGGACAAATATTTCGTAAAATTAATCAAGATACATGTAGACCTGAACAACACCATTGATAATTATACAAAAA : 2583
Brandywine : AATAAGTCGAATAACGTAAAGTACAAATTTTTTTCATCTTATTGCATTATTGATTAGAGAGGACAAATATTTCGTAAAATTAATCAAGATACATGTAGACCTGAACAACACCATTGATAATTATACAAAAA : 2583
EA00990.1  : AATAAGTCGAATAACGTAAAGTACAAATTTTTTTCATCTTATTGCATTATTGATTAGAGAGGACAAATATTTCGTAAAATTAATCAAGATACATGTAGACCTGAACAACACCATTGATAATTATACAAAAA : 2583
PI303721.1 : AATAAGTCGAATAACGTAAAGTACAAATTTTTTTCATCTTATTGCATTATTGATTAGAGAGGACAAATATTTCGTAAAATTAATCAAGATACATGTAGACCTGAACAACACCATTGATAATTATACAAAAA : 2583
PAS014479. : AATAAGTCGAATAACGTAAAGTACAAATTTTTTTCATCTTATTGCATTATTAATTAGAGAGGACAAATATTTCGTAAAATTAATCAAGATACATGTAGACCTGAACAACACCATTGATAATTATACAAAAA : 2580
BGV006775. : AATAAGTCGAATAACGTAAAGTACAAATTTTTTTCATCTTATTGCATTATTGATTAGAGAGGACAAATATTTCGTAAAATTAATCAAGATACATGTAGACCTGAACAACACCATTGATAATTATACAAAAA : 2582
             AATAAGTCGAATAACGTAAAGTACAAATTTTTTTCATCTTATTGCATTATTgATTAGAGAGGACAAATATTTCGTAAAATTAATCAAGATACATGTAGACCTGAACAACACCATTGATAATTATACAAAAA

                  2760         *     2780         *     2800         *     2820         *     2840         *     2860         *     2880
ITAG.1     : AGGAATTACAAGAAACACTTACAAGAAACACAAAGAAAAGTCAATATTCCATAGCATATCCTTACTAGAAAAGTTGGTAGCCAGATGACAATGCAATAAATCTAAGGACTTTTCTAGCTAATTTAGTTGAA : 2713
BGV006865. : AGAAA-------------TTACAAGAAACACAAAGAAAAGTCAATATTCCATAGCATATCCTTACTAGAAAAGTTGGTAGCCAGATGACAATGCAATAAATCTAAGGACTTTTCTAGCTAATTTAGTTGAA : 2700
BGV007931. : AGGAATTACAAGAAACACTTACAAGAAACACAAAGAAAAGTCAATATTCCATAGCATATCCTTACTAGAAAAGTTGGTAGCCAGATGACAATGCAATAAATCTAAGGACTTTTCTAGCTAATTTAGTTGAA : 2714
BGV007989. : AGGAATTACAAGAAACACTTACAAGAAACACAAAGAAAAGTCAATATTCCATAGCATATCCTTACTAGAAAAGTTGGTAGCCAGATGACAATGCAATAAATCTAAGGACTTTTCTAGCTAATTTAGTTGAA : 2714
M82.1      : AGGAATTACAAGAAACACTTACAAGAAACACAAAGAAAAGTCAATATTCCATAGCATATCCTTACTAGAAAAGTTGGTAGCCAGATGACAATGCAATAAATCTAAGGACTTTTCTAGCTAATTTAGTTGAA : 2714
EA00371.1  : AGGAATTACAAGAAACACTTACAAGAAACACAAAGAAAAGTCAATATTCCATAGCATATCCTTACTAGAAAAGTTGGTAGCCAGATGACAATGCAATAAATCTAAGGACTTTTCTAGCTAATTTAGTTGAA : 2714
Fla.8924.1 : AGGAATTACAAGAAACACTTACAAGAAACACAAAGAAAAGTCAATATTCCATAGCATATCCTTACTAGAAAAGTTGGTAGCCAGATGACAATGCAATAAATCTAAGGACTTTTCTAGCTAATTTAGTTGAA : 2714
Floradade. : AGGAATTACAAGAAACACTTACAAGAAACACAAAGAAAAGTCAATATTCCATAGCATATCCTTACTAGAAAAGTTGGTAGCCAGATGACAATGCAATAAATCTAAGGACTTTTCTAGCTAATTTAGTTGAA : 2714
LYC1410.1  : AGGAATTACAAGAAACACTTACAAGAAACACAAAGAAAAGTCAATATTCCATAGCATATCCTTACTAGAAAAGTTGGTAGCCAGATGACAATGCAATAAATCTAAGGACTTTTCTAGCTAATTTAGTTGAA : 2714
PI169588.1 : AGGAATTACAAGAAACACTTACAAGAAACACAAAGAAAAGTCAATATTCCATAGCATATCCTTACTAGAAAAGTTGGTAGCCAGATGACAATGCAATAAATCTAAGGACTTTTCTAGCTAATTTAGTTGAA : 2714
Brandywine : AGGAATTACAAGAAACACTTACAAGAAACACAAAGAAAAGTCAATATTCCATAGCATATCCTTACTAGAAAAGTTGGTAGCCAGATGACAATGCAATAAATCTAAGGACTTTTCTAGCTAATTTAGTTGAA : 2714
EA00990.1  : AGGAATTACAAGAAACACTTACAAGAAACACAAAGAAAAGTCAATATTCCATAGCATATCCTTACTAGAAAAGTTGGTAGCCAGATGACAATGCAATAAATCTAAGGACTTTTCTAGCTAATTTAGTTGAA : 2714
PI303721.1 : AGGAATTACAAGAAACACTTACAAGAAACACAAAGAAAAGTCAATATTCCATAGCATATCCTTACTAGAAAAGTTGGTAGCCAGATGACAATGCAATAAATCTAAGGACTTTTCTAGCTAATTTAGTTGAA : 2714
PAS014479. : AGGAATTACAAGAAACACTTACAAGAAACACAAAGAAAAGTCAATATTCCATAGCATATCCTTACTAGAAAAGTTGGTAGCCAGATGACAATGCAATAAATCTAAGGACTTTTCTAGCTAATTTAGTTGAA : 2711
BGV006775. : AGAAA-------------TTACAAGAAACACAAAGAAAAGTCAATATTCCATAGCATATCCTTACTAGAAAAGTTGGTAGCCAGATGACAATGCAATAAATCTAAGGACTTTTCTAGCTAATTTAGTTGAA : 2700
             AGgAAttacaagaaacacTTACAAGAAACACAAAGAAAAGTCAATATTCCATAGCATATCCTTACTAGAAAAGTTGGTAGCCAGATGACAATGCAATAAATCTAAGGACTTTTCTAGCTAATTTAGTTGAA

                   *      2900         *     2920         *     2940         *     2960         *     2980         *     3000         *
ITAG.1     : AAGGACCTCTTTCATTATAATTTTAAATTAAAAGATTGTAATAATGAAAGAGAT-AATG--CAAGAATTCTCTCAACCTATGTCTGGAATTTTAGAGACACAATTATATTATACTAAGGTTCTATTATTCT : 2841
BGV006865. : AAGGACCTCTTTCATTATAATTTTAAATTAAAAGATTGTAATAATGAAAGAGATAAATGCACAAGAATTTTCTCAACCTATGTCTGGAATTTTAGAGACACAGTTATATTATACTAAGGTTCTATTATTCT : 2831
BGV007931. : AAGGACCTCTTTCATTATAATTTTAAATTAAAAGATTGTAATAATGAAAGAGAT-AATG--CAAGAATTCTCTCAACCTATGTCTGGAATTTTAGAGACACAATTATATTATACTAAGGTTCTATTATTCT : 2842
BGV007989. : AAGGACCTCTTTCATTATAATTTTAAATTAAAAGATTGTAATAATGAAAGAGAT-AATG--CAAGAATTCTCTCAACCTATGTCTGGAATTTTAGAGACACAATTATATTATACTAAGGTTCTATTATTCT : 2842
M82.1      : AAGGACCTCTTTCATTATAATTTTAAATTAAAAGATTGTAATAATGAAAGAGAT-AATG--CAAGAATTCTCTCAACCTATGTCTGGAATTTTAGAGACACAATTATATTATACTAAGGTTCTATTATTCT : 2842
EA00371.1  : AAGGACCTCTTTCATTATAATTTTAAATTAAAAGATTGTAATAATGAAAGAGAT-AATG--CAAGAATTCTCTCAACCTATGTCTGGAATTTTAGAGACACAATTATATTATACTAAGGTTCTATTATTCT : 2842
Fla.8924.1 : AAGGACCTCTTTCATTATAATTTTAAATTAAAAGATTGTAATAATGAAAGAGAT-AATG--CAAGAATTCTCTCAACCTATGTCTGGAATTTTAGAGACACAATTATATTATACTAAGGTTCTATTATTCT : 2842
Floradade. : AAGGACCTCTTTCATTATAATTTTAAATTAAAAGATTGTAATAATGAAAGAGAT-AATG--CAAGAATTCTCTCAACCTATGTCTGGAATTTTAGAGACACAATTATATTATACTAAGGTTCTATTATTCT : 2842
LYC1410.1  : AAGGACCTCTTTCATTATAATTTTAAATTAAAAGATTGTAATAATGAAAGAGAT-AATG--CAAGAATTCTCTCAACCTATGTCTGGAATTTTAGAGACACAATTATATTATACTAAGGTTCTATTATTCT : 2842
PI169588.1 : AAGGACCTCTTTCATTATAATTTTAAATTAAAAGATTGTAATAATGAAAGAGAT-AATG--CAAGAATTCTCTCAACCTATGTCTGGAATTTTAGAGACACAATTATATTATACTAAGGTTCTATTATTCT : 2842
Brandywine : AAGGACCTCTTTCATTATAATTTTAAATTAAAAGATTGTAATAATGAAAGAGAT-AATG--CAAGAATTCTCTCAACCTATGTCTGGAATTTTAGAGACACAATTATATTATACTAAGGTTCTATTATTCT : 2842
EA00990.1  : AAGGACCTCTTTCATTATAATTTTAAATTAAAAGATTGTAATAATGAAAGAGAT-AATG--CAAGAATTCTCTCAACCTATGTCTGGAATTTTAGAGACACAATTATATTATACTAAGGTTCTATTATTCT : 2842
PI303721.1 : AAGGACCTCTTTCATTATAATTTTAAATTAAAAGATTGTAATAATGAAAGAGAT-AATG--CAAGAATTCTCTCAACCTATGTCTGGAATTTTAGAGACACAATTATATTATACTAAGGTTCTATTATTCT : 2842
PAS014479. : AAGGACCTCTTTCATTATAATTTTAAATTAAAAGATTGTAATAATGAAAGAGAT-AATGCACAAGAATTCTCTCAATCTATGTCTGGAATTTTAGAGACACAGTTATATTATACTAAGGTTCTATTATTCT : 2841
BGV006775. : AAGGACCTCTTTCATTATAATTTTAAATTAAAAGATTGTAATAATGAAAGAGATAAATGCACAAGAATTCTCTCAACCTATGTCTGGAATTTTAGAGACACAGTTATATTATACTAAGGTTCTATTATTCT : 2831
             AAGGACCTCTTTCATTATAATTTTAAATTAAAAGATTGTAATAATGAAAGAGAT AATG  CAAGAATTcTCTCAAcCTATGTCTGGAATTTTAGAGACACAaTTATATTATACTAAGGTTCTATTATTCT

               3020         *     3040         *     3060         *     3080         *     3100         *     3120         *     3140
ITAG.1     : CCTGAACTTATTATATTAATAATTTTTTACTTCTTTTCGACCTACGTAACACTATCTTGTGGGCCTAATACTGGTTGATTTTTTTTCAAACTAGTGCCACGTAGCCCGAAAAGGATAGAACATTACTTATA : 2972
BGV006865. : CCTGAATTTATTATATTAATAATTTTTTACCTCTTTTCGACCTACGTAACACTATCTTGTGGGCCCAATACTGGTTGATTTTTTTTTAAACTAGTGCCACGTAGCCCGAAAAGGGTAGAACATTACTTATA : 2962
BGV007931. : CCTGAACTTATTATATTAATAATTTTTTACTTCTTTTCGACCTACGTAACACTATCTTGTGGGCCCAATACTGGTTGATTTTTTTTCAAACTAGTGCCACGTAGCCCGAAAAGGGTAGAACATTACTTATA : 2973
BGV007989. : CCTGAACTTATTATATTAATAATTTTTTACTTCTTTTCGACCTACGTAACACTATCTTGTGGGCCTAATACTGGTTGATTTTTTTTCAAACTAGTGCCACGTAGCCCGAAAAGGATAGAACATTACTTATA : 2973
M82.1      : CCTGAACTTATTATATTAATAATTTTTTACTTCTTTTCGACCTACGTAACACTATCTTGTGGGCCTAATACTGGTTGATTTTTTTTCAAACTAGTGCCACGTAGCCCGAAAAGGATAGAACATTACTTATA : 2973
EA00371.1  : CCTGAACTTATTATATTAATAATTTTTTACTTCTTTTCGACCTACGTAACACTATCTTGTGGGCCTAATACTGGTTGATTTTTTTTCAAACTAGTGCCACGTAGCCCGAAAAGGATAGAACATTACTTATA : 2973
Fla.8924.1 : CCTGAACTTATTATATTAATAATTTTTTACTTCTTTTCGACCTACGTAACACTATCTTGTGGGCCTAATACTGGTTGATTTTTTTTCAAACTAGTGCCACGTAGCCCGAAAAGGATAGAACATTACTTATA : 2973
Floradade. : CCTGAACTTATTATATTAATAATTTTTTACTTCTTTTCGACCTACGTAACACTATCTTGTGGGCCTAATACTGGTTGATTTTTTTTCAAACTAGTGCCACGTAGCCCGAAAAGGATAGAACATTACTTATA : 2973
LYC1410.1  : CCTGAACTTATTATATTAATAATTTTTTACTTCTTTTCGACCTACGTAACACTATCTTGTGGGCCTAATACTGGTTGATTTTTTTTCAAACTAGTGCCACGTAGCCCGAAAAGGATAGAACATTACTTATA : 2973
PI169588.1 : CCTGAACTTATTATATTAATAATTTTTTACTTCTTTTCGACCTACGTAACACTATCTTGTGGGCCTAATACTGGTTGATTTTTTTTCAAACTAGTGCCACGTAGCCCGAAAAGGATAGAACATTACTTATA : 2973
Brandywine : CCTGAACTTATTATATTAATAATTTTTTACTTCTTTTCGACCTACGTAACACTATCTTGTGGGCCTAATACTGGTTGATTTTTTTTCAAACTAGTGCCACGTAGCCCGAAAAGGATAGAACATTACTTATA : 2973
EA00990.1  : CCTGAACTTATTATATTAATAATTTTTTACTTCTTTTCGACCTACGTAACACTATCTTGTGGGCCTAATACTGGTTGATTTTTTTTCAAACTAGTGCCACGTAGCCCGAAAAGGATAGAACATTACTTATA : 2973
PI303721.1 : CCTGAACTTATTATATTAATAATTTTTTACTTCTTTTCGACCTACGTAACACTATCTTGTGGGCCTAATACTGGTTGATTTTTTTTCAAACTAGTGCCACGTAGCCCGAAAAGGATAGAACATTACTTATA : 2973
PAS014479. : CCTGATCTTATTATATTAATAATTTTTTACCTCTTTTCGACCTACGTAACACTATCTTGTGGGCCCAATTCTGGTTGATTTTTTTTCAAACTAGTGCCACGTAGCCCGGAAAGGGTAGAACAGTACTTATA : 2972
BGV006775. : CCTGAATTTATTATATTAATAATTTTTTACCTCTTTTCGACCTACGTAACACTATCTTGTGGGCCCAATACTGGTTGATTTTTTTTTAAACTAGTGCCACGTAGCCCGAAAAGGGTAGAACATTACTTATA : 2962
```

```
CCTGAacTTATTATATTAATAATTTTTTACtTCTTTTCGACCTACGTAACACTATCTTGTGGGCC AATaCTGGTTGATTTTTTTTcAAACTAGTGCCACGTAGCCCGaAAAGG TAGAACAtTACTTATA

                  *       3160         *       3180         *       3200
ITAG.1      : AAATAAGTTCAGGAGGTAATAGAACCTTAGAATAGTATAAGTGAGTCT---------- : 3020
BGV006865.  : AAATAAGTTCAGGAGGTAATAGAACCTTAAAATAGTATAAGTGAGTCTCTAAAATTTC : 3020
BGV007931.  : AAATAAGTTCAGGAGGTAATAGAACCTTAGAATAGTATAAGTGAGTC----------- : 3020
BGV007989.  : AAATAAGTTCAGGAGGTAATAGAACCTTAGAATAGTATAAGTGAGTC----------- : 3020
M82.1       : AAATAAGTTCAGGAGGTAATAGAACCTTAGAATAGTATAAGTGAGTC----------- : 3020
EA00371.1   : AAATAAGTTCAGGAGGTAATAGAACCTTAGAATAGTATAAGTGAGTC----------- : 3020
Fla.8924.1  : AAATAAGTTCAGGAGGTAATAGAACCTTAGAATAGTATAAGTGAGTC----------- : 3020
Floradade.  : AAATAAGTTCAGGAGGTAATAGAACCTTAGAATAGTATAAGTGAGTC----------- : 3020
LYC1410.1   : AAATAAGTTCAGGAGGTAATAGAACCTTAGAATAGTATAAGTGAGTC----------- : 3020
PI169588.1  : AAATAAGTTCAGGAGGTAATAGAACCTTAGAATAGTATAAGTGAGTC----------- : 3020
Brandywine  : AAATAAGTTCAGGAGGTAATAGAACCTTAGAATAGTATAAGTGAGTC----------- : 3020
EA00990.1   : AAATAAGTTCAGGAGGTAATAGAACCTTAGAATAGTATAAGTGAGTC----------- : 3020
PI303721.1  : AAATAAGTTCAGGAGGTAATAGAACCTTAGAATAGTATAAGTGAGTC----------- : 3020
PAS014479.  : AAATAAGTTCAGGAGGTAATAGAACCTTAGAATAGTATAAGTGAGTCT---------- : 3020
BGV006775.  : AAATAAGTTCAGGAGGTAATAGAACCTTAAAATAGTATAAGTGAGTCTCTAAAATTTC : 3020
              AAATAAGTTCAGGAGGTAATAGAACCTTAgAATAGTATAAGTGAGTC
```

Solyc02g080300

```
                    *        20         *        40         *        60         *        80         *       100         *       120         *
ITAG.3      : CATAGCTACTTAAATTCATTGGCAAATGCAATCACGGATGGTGCAGATGTGAGAGGGTACTTTGTTTGGTCCCTTCTCGACAACTTTGAGTGGCTAGATGGATATAAGTTAAGATTTGGACTTCACTATGT :  131
BGV006865.  : CATAGCTACTTAAATTCATTGGCAAATGCAATCACGGATGGTGCAGATGTGAGAGGGTACTTTGTTTGGTCCCTTCTCGACAACTTTGAGTGGCTAGATGGATATAAGTTAAGATTTGGACTTCACTATGT :  131
BGV007931.  : CATAGCTACTTAAATTCATTGGCAAATGCAATCACGGATGGTGCAGATGTGAGAGGGTACTTTGTTTGGTCCCTTCTCGACAACTTTGAGTGGCTAGATGGATATAAGTTAAGATTTGGACTTCACTATGT :  131
BGV007989.  : CATAGCTACTTAAATTCATTGGCAAATGCAATCACGGATGGTGCAGATGTGAGAGGGTACTTTGTTTGGTCCCTTCTCGACAACTTTGAGTGGCTAGATGGATATAAGTTAAGATTTGGACTTCACTATGT :  131
M82.3       : CATAGCTACTTAAATTCATTGGCAAATGCAATCACGGATGGTGCAGATGTGAGAGGGTACTTTGTTTGGTCCCTTCTCGACAACTTTGAGTGGCTAGATGGATATAAGTTAAGATTTGGACTTCACTATGT :  131
EA00371.3   : CATAGCTACTTAAATTCATTGGCAAATGCAATCACGGATGGTGCAGATGTGAGAGGGTACTTTGTTTGGTCCCTTCTCGACAACTTTGAGTGGCTAGATGGATATAAGTTAAGATTTGGACTTCACTATGT :  131
Fla.8924.3  : CATAGCTACTTAAATTCATTGGCAAATGCAATCACGGATGGTGCAGATGTGAGAGGGTACTTTGTTTGGTCCCTTCTCGACAACTTTGAGTGGCTAGATGGATATAAGTTAAGATTTGGACTTCACTATGT :  131
Floradade.  : CATAGCTACTTAAATTCATTGGCAAATGCAATCACGGATGGTGCAGATGTGAGAGGGTACTTTGTTTGGTCCCTTCTCGACAACTTTGAGTGGCTAGATGGATATAAGTTAAGATTTGGACTTCACTATGT :  131
LYC1410.3   : CATAGCTACTTAAATTCATTGGCAAATGCAATCACGGATGGTGCAGATGTGAGAGGGTACTTTGTTTGGTCCCTTCTCGACAACTTTGAGTGGCTAGATGGATATAAGTTAAGATTTGGACTTCACTATGT :  131
PI169588.3  : CATAGCTACTTAAATTCATTGGCAAATGCAATCACGGATGGTGCAGATGTGAGAGGGTACTTTGTTTGGTCCCTTCTCGACAACTTTGAGTGGCTAGATGGATATAAGTTAAGATTTGGACTTCACTATGT :  131
Brandywine  : CATAGCTACTTAAATTCATTGGCAAATGCAATCACGGATGGTGCAGATGTGAGAGGGTACTTTGTTTGGTCCCTTCTCGACAACTTTGAGTGGCTAGATGGATATAAGTTAAGATTTGGACTTCACTATGT :  131
EA00990.3   : CATAGCTACTTAAATTCATTGGCAAATGCAATCACGGATGGTGCAGATGTGAGAGGGTACTTTGTTTGGTCCCTTCTCGACAACTTTGAGTGGCTAGATGGATATAAGTTAAGATTTGGACTTCACTATGT :  131
PI303721.3  : CATAGCTACTTAAATTCATTGGCAAATGCAATCACGGATGGTGCAGATGTGAGAGGGTACTTTGTTTGGTCCCTTCTCGACAACTTTGAGTGGCTAGATGGATATAAGTTAAGATTTGGACTTCACTATGT :  131
BGV006775.  : -ATAGCTACTTAAATTCATTGGCAAATGCAATCACGGATGGTGCAGATGTGAGAGGGTACTTTGTTTGGTCCCTTCTCGACAACTTTGAGTGGCTAGATGGATATAAGTTAAGATTTGGACTTCACTATGT :  130
PAS014479.  : CATAGCTACTTAAATTCATTGGCAAATGCAATCACGGATGGTGCAGATGTGAGAGGGTACTTTGTTTGGTCCCTTCTCGACAACTTTGAGTGGCTAGATGGATATAAGTTAAGATTTGGACTTCACTATGT :  131
              cATAGCTACTTAAATTCATTGGCAAATGCAATCACGGATGGTGCAGATGTGAGAGGGTACTTTGTTTGGTCCCTTCTCGACAACTTTGAGTGGCTAGATGGATATAAGTTAAGATTTGGACTTCACTATGT

                140         *       160         *       180         *       200         *       220         *       240         *       260
ITAG.3      : CAACTATACTAATCTTCAGAGAACCCCAAAATTATCAGCTACCATGTATAAACAGCTCATGTATAACTTCAA-CATACAACTCGAAAAAAATACTGCCCAGAACTAGTAGGAGAAGATGATGGAAAGTAAA :  261
BGV006865.  : CAACTATACTAATCTTCAGAGAACCCCAAAATTATCAGCTACCATGTATAAACAGCTCATGTATAACTTCAA-CATACAACTCGAAAAAAATACTGCCCAGAACTAGTAGGAGAAGATGATGGAAAGTAAA :  261
BGV007931.  : CAACTATACTAATCTTCAGAGAACCCCAAAATTATCAGCTACCATGTATAAACAGCTCATGTATAACTTCAA-CATACAACTCGAAAAAAATACTGCCCAGAACTAGTAGGAGAAGATGATGGAAAGTAAA :  261
BGV007989.  : CAACTATACTAATCTTCAGAGAACCCCAAAATTATCAGCTACCATGTATAAACAGCTCATGTATAACTTCAA-CATACAACTCGAAAAAAATACTGCCCAGAACTAGTAGGAGAAGATGATGGAAAGTAAA :  261
M82.3       : CAACTATACTAATCTTCAGAGAACCCCAAAATTATCAGCTACCATGTATAAACAGCTCATGTATAACTTCAA-CATACAACTCGAAAAAAATACTGCCCAGAACTAGTAGGAGAAGATGATGGAAAGTAAA :  261
EA00371.3   : CAACTATACTAATCTTCAGAGAACCCCAAAATTATCAGCTACCATGTATAAACAGCTCATGTATAACTTCAA-CATACAACTCGAAAAAAATACTGCCCAGAACTAGTAGGAGAAGATGATGGAAAGTAAA :  261
Fla.8924.3  : CAACTATACTAATCTTCAGAGAACCCCAAAATTATCAGCTACCATGTATAAACAGCTCATGTATAACTTCAA-CATACAACTCGAAAAAAATACTGCCCAGAACTAGTAGGAGAAGATGATGGAAAGTAAA :  261
Floradade.  : CAACTATACTAATCTTCAGAGAACCCCAAAATTATCAGCTACCATGTATAAACAGCTCATGTATAACTTCAA-CATACAACTCGAAAAAAATACTGCCCAGAACTAGTAGGAGAAGATGATGGAAAGTAAA :  261
LYC1410.3   : CAACTATACTAATCTTCAGAGAACCCCAAAATTATCAGCTACCATGTATAAACAGCTCATGTATAACTTCAA-CATACAACTCGAAAAAAATACTGCCCAGAACTAGTAGGAGAAGATGATGGAAAGTAAA :  261
PI169588.3  : CAACTATACTAATCTTCAGAGAACCCCAAAATTATCAGCTACCATGTATAAACAGCTCATGTATAACTTCAA-CATACAACTCGAAAAAAATACTGCCCAGAACTAGTAGGAGAAGATGATGGAAAGTAAA :  261
Brandywine  : CAACTATACTAATCTTCAGAGAACCCCAAAATTATCAGCTACCATGTATAAACAGCTCATGTATAACTTCAA-CATACAACTCGAAAAAAATACTGCCCAGAACTAGTAGGAGAAGATGATGGAAAGTAAA :  261
EA00990.3   : CAACTATACTAATCTTCAGAGAACCCCAAAATTATCAGCTACCATGTATAAACAGCTCATGTATAACTTCAA-CATACAACTCGAAAAAAATACTGCCCAGAACTAGTAGGAGAAGATGATGGAAAGTAAA :  261
PI303721.3  : CAACTATACTAATCTTCAGAGAACCCCAAAATTATCAGCTACCATGTATAAACAGCTCATGTATAACTTCAA-CATACAACTCGAAAAAAATACTGCCCAGAACTAGTAGGAGAAGATGATGGAAAGTAAA :  261
BGV006775.  : CAACTATACTAATCTTCAGAGAACCCCAAAATTATCAGCTACCATGTATAAACAGCTCATGTATAACTTCAAGAATACAACTCGAAAAAAATACTGCCCAGAACTAGTAGGAGAAGATGATGGAAAGTAAA :  261
PAS014479.  : CAACTATACTAATCTTCAGAGAACCCCAAAATTATCAGCTACCATGTATAAACAGCTCATGTATAACTTCAA-CATACAACTCGAAAAAAATACTGCCCAGAACTAGTAGGAGAAGATGATGGAAAGTAAA :  261
              CAACTATACTAATCTTCAGAGAACCCCAAAATTATCAGCTACCATGTATAAACAGCTCATGTATAACTTCAA cATACAACTCGAAAAAAATACTGCCCAGAACTAGTAGGAGAAGATGATGGAAAGTAAA

                    *       280         *       300         *       320         *       340         *       360         *       380         *
ITAG.3      : TATTATTTTCACTCCAGTGGGACTGTAATCATTTAAACAAGATTTGTTAATTACACAGAAACATTCTGTTGGAAGAGGTTGTCTCATATTTGAATGCTAGTCTATATATAACTTCTTGGCGTAGAGACCCA :  392
BGV006865.  : TATTATTTTCACTCCAGTGGGACTGTAATCATTTAAACAAGATTTGTTAATTACACAGAAACATTCTGTTGGAAGAGGTTGTCTCATATTTGAATGCTAGTCTATATATAACTTCTTGGCGTAGAGACCCA :  392
BGV007931.  : TATTATTTTCACTCCAGTGGGACTGTAATCATTTAAACAAGATTTGTTAATTACACAGAAACATTCTGTTGGAAGAGGTTGTCTCATATTTGAATGCTAGTCTATATATAACTTCTTGGCGTAGAGACCCA :  392
BGV007989.  : TATTATTTTCACTCCAGTGGGACTGTAATCATTTAAACAAGATTTGTTAATTACACAGAAACATTCTGTTGGAAGAGGTTGTCTCATATTTGAATGCTAGTCTATATATAACTTCTTGGCGTAGAGACCCA :  392
M82.3       : TATTATTTTCACTCCAGTGGGACTGTAATCATTTAAACAAGATTTGTTAATTACACAGAAACATTCTGTTGGAAGAGGTTGTCTCATATTTGAATGCTAGTCTATATATAACTTCTTGGCGTAGAGACCCA :  392
EA00371.3   : TATTATTTTCACTCCAGTGGGACTGTAATCATTTAAACAAGATTTGTTAATTACACAGAAACATTCTGTTGGAAGAGGTTGTCTCATATTTGAATGCTAGTCTATATATAACTTCTTGGCGTAGAGACCCA :  392
Fla.8924.3  : TATTATTTTCACTCCAGTGGGACTGTAATCATTTAAACAAGATTTGTTAATTACACAGAAACATTCTGTTGGAAGAGGTTGTCTCATATTTGAATGCTAGTCTATATATAACTTCTTGGCGTAGAGACCCA :  392
Floradade.  : TATTATTTTCACTCCAGTGGGACTGTAATCATTTAAACAAGATTTGTTAATTACACAGAAACATTCTGTTGGAAGAGGTTGTCTCATATTTGAATGCTAGTCTATATATAACTTCTTGGCGTAGAGACCCA :  392
LYC1410.3   : TATTATTTTCACTCCAGTGGGACTGTAATCATTTAAACAAGATTTGTTAATTACACAGAAACATTCTGTTGGAAGAGGTTGTCTCATATTTGAATGCTAGTCTATATATAACTTCTTGGCGTAGAGACCCA :  392
PI169588.3  : TATTATTTTCACTCCAGTGGGACTGTAATCATTTAAACAAGATTTGTTAATTACACAGAAACATTCTGTTGGAAGAGGTTGTCTCATATTTGAATGCTAGTCTATATATAACTTCTTGGCGTAGAGACCCA :  392
Brandywine  : TATTATTTTCACTCCAGTGGGACTGTAATCATTTAAACAAGATTTGTTAATTACACAGAAACATTCTGTTGGAAGAGGTTGTCTCATATTTGAATGCTAGTCTATATATAACTTCTTGGCGTAGAGACCCA :  392
EA00990.3   : TATTATTTTCACTCCAGTGGGACTGTAATCATTTAAACAAGATTTGTTAATTACACAGAAACATTCTGTTGGAAGAGGTTGTCTCATATTTGAATGCTAGTCTATATATAACTTCTTGGCGTAGAGACCCA :  392
PI303721.3  : TATTATTTTCACTCCAGTGGGACTGTAATCATTTAAACAAGATTTGTTAATTACACAGAAACATTCTGTTGGAAGAGGTTGTCTCATATTTGAATGCTAGTCTATATATAACTTCTTGGCGTAGAGACCCA :  392
BGV006775.  : TATTATTTTCACTCCAGTGGGACTGTAATCATTTAAACAAGATTTGTTAATTACACAGAAACATTCTGTTGGAAGAAGTTGTCTCATATTTGAATGCTAGTCTATATATAACTTCTTGGCGTAGAGACCCA :  392
PAS014479.  : TATTATTTTCACTCCAGTGGGACTGTAATCATTTAAACAAGATTTGTTAATTACACAGAAACATTCTGTTGGAAGAGGTTGTCTCATATTTGAATGCTAGTCTATATATAACTTGTTGGCGTAGAGACCCA :  392
              TATTATTTTCACTCCAGTGGGACTGTAATCATTTAAACAAGATTTGTTAATTACACAGAAACATTCTGTTGGAAGAgGTTGTCTCATATTTGAATGCTAGTCTATATATAACTTcTTGGCGTAGAGACCCA

                  400         *       420         *       440         *       460         *       480         *       500         *       520
ITAG.3      : AGCTAACTAGAATCTACAATAACTCCATAATATAGGGAAGTCCAAAGTCTAGCATAATAAAAAAATGAAGTTCAAGAGAGAGCAACCATTTGAATTATATTTTTGTACTAATATACTTTGTTAAATGGTGCA :  523
BGV006865.  : AGCTAACTAGAATCTACAATAACTCCATAATATAGGGAAGTCCAAAGTCTAGCATAATAAAAAAATGAAGTTCAAGAGAGAGCAACCATTTGAATTATATTTTTGTACTAATATACTTTGTTAAATGGTGCA :  523
BGV007931.  : AGCTAACTAGAATCTACAATAACTCCATAATATAGGGAAGTCCAAAGTCTAGCATAATAAAAAAATGAAGTTCAAGAGAGAGCAACCATTTGAATTATATTTTTGTACTAATATACTTTGTTAAATGGTGCA :  523
BGV007989.  : AGCTAACTAGAATCTACAATAACTCCATAATATAGGGAAGTCCAAAGTCTAGCATAATAAAAAAATGAAGTTCAAGAGAGAGCAACCATTTGAATTATATTTTTGTACTAATATACTTTGTTAAATGGTGCA :  523
M82.3       : AGCTAACTAGAATCTACAATAACTCCATAATATAGGGAAGTCCAAAGTCTAGCATAATAAAAAAATGAAGTTCAAGAGAGAGCAACCATTTGAATTATATTTTTGTACTAATATACTTTGTTAAATGGTGCA :  523
EA00371.3   : AGCTAACTAGAATCTACAATAACTCCATAATATAGGGAAGTCCAAAGTCTAGCATAATAAAAAAATGAAGTTCAAGAGAGAGCAACCATTTGAATTATATTTTTGTACTAATATACTTTGTTAAATGGTGCA :  523
Fla.8924.3  : AGCTAACTAGAATCTACAATAACTCCATAATATAGGGAAGTCCAAAGTCTAGCATAATAAAAAAATGAAGTTCAAGAGAGAGCAACCATTTGAATTATATTTTTGTACTAATATACTTTGTTAAATGGTGCA :  523
Floradade.  : AGCTAACTAGAATCTACAATAACTCCATAATATAGGGAAGTCCAAAGTCTAGCATAATAAAAAAATGAAGTTCAAGAGAGAGCAACCATTTGAATTATATTTTTGTACTAATATACTTTGTTAAATGGTGCA :  523
LYC1410.3   : AGCTAACTAGAATCTACAATAACTCCATAATATAGGGAAGTCCAAAGTCTAGCATAATAAAAAAATGAAGTTCAAGAGAGAGCAACCATTTGAATTATATTTTTGTACTAATATACTTTGTTAAATGGTGCA :  523
PI169588.3  : AGCTAACTAGAATCTACAATAACTCCATAATATAGGGAAGTCCAAAGTCTAGCATAATAAAAAAATGAAGTTCAAGAGAGAGCAACCATTTGAATTATATTTTTGTACTAATATACTTTGTTAAATGGTGCA :  523
Brandywine  : AGCTAACTAGAATCTACAATAACTCCATAATATAGGGAAGTCCAAAGTCTAGCATAATAAAAAAATGAAGTTCAAGAGAGAGCAACCATTTGAATTATATTTTTGTACTAATATACTTTGTTAAATGGTGCA :  523
EA00990.3   : AGCTAACTAGAATCTACAATAACTCCATAATATAGGGAAGTCCAAAGTCTAGCATAATAAAAAAATGAAGTTCAAGAGAGAGCAACCATTTGAATTATATTTTTGTACTAATATACTTTGTTAAATGGTGCA :  523
PI303721.3  : AGCTAACTAGAATCTACAATAACTCCATAATATAGGGAAGTCCAAAGTCTAGCATAATAAAAAAATGAAGTTCAAGAGAGAGCAACCATTTGAATTATATTTTTGTACTAATATACTTTGTTAAATGGTGCA :  523
BGV006775.  : AGCTAACTAGAATCTACAATAACTCCATAATATAGGGAAGTCCAAAGTCTAGCATAATAAAAAAATGAAGTTCAAGAGAGAGCAACCATTTGAATTATATTTTTGTACTAATATACTTTGTTAAATGGTGCA :  523
PAS014479.  : AGCTAACTAGAATCTACAATAACTCCATAATATAGGGAAGTCCAAAGTCTAGCATAATAAAAAAATGAAGTTCAAGAGAGAGCAACCATTTGAATTATATTTTTGTACTAATATACTTTGTTAAATGGTGCA :  523
              AGCTAACTAGAATCTACAATAACTCCATAATATAGGGAAGTCCAAAGTCTAGCATAATAAAAAAATGAAGTTCAAGAGAGAGCAACCATTTGAATTATATTTTTGTACTAATATACTTTGTTAAATGGTGCA

                    *       540         *       560         *       580         *       600         *       620         *       640         *
ITAG.3      : GTTATAGAATTGACAGTTAGTAGAATGGCCCTATAAAAATCTTATTACCCTGATAGATTAATGGCTCAGATGTAACAAAGTAGAAAATTATAAACTGCTATACATGCATGGACAATTGACCTAGCGATTAT :  654
BGV006865.  : GTTATAGAATTGACAGTTAGTAGAATGGCCCTATAAAAATCTTATTATCCTGATAGATTAATGGCTCAGATGTAACAAAGTAGAAAATTATAAACTGCTATACATGCATGGACAATTGACCTAGCGATTAT :  654
BGV007931.  : GTTATAGAATTGACAGTTAGTAGAATGGCCCTATAAAAATCTTATTACCCTGATAGATTAATGGCTCAGATGTAACAAAGTAGAAAATTATAAACTGCTATACATGCATGGACAATTGACCTAGCGATTAT :  654
BGV007989.  : GTTATAGAATTGACAGTTAGTAGAATGGCCCTATAAAAATCTTATTACCCTGATAGATTAATGGCTCAGATGTAACAAAGTAGAAAATTATAAACTGCTATACATGCATGGACAATTGACCTAGCGATTAT :  654
M82.3       : GTTATAGAATTGACAGTTAGTAGAATGGCCCTATAAAAATCTTATTACCCTGATAGATTAATGGCTCAGATGTAACAAAGTAGAAAATTATAAACTGCTATACATGCATGGACAATTGACCTAGCGATTAT :  654
EA00371.3   : GTTATAGAATTGACAGTTAGTAGAATGGCCCTATAAAAATCTTATTACCCTGATAGATTAATGGCTCAGATGTAACAAAGTAGAAAATTATAAACTGCTATACATGCATGGACAATTGACCTAGCGATTAT :  654
Fla.8924.3  : GTTATAGAATTGACAGTTAGTAGAATGGCCCTATAAAAATCTTATTACCCTGATAGATTAATGGCTCAGATGTAACAAAGTAGAAAATTATAAACTGCTATACATGCATGGACAATTGACCTAGCGATTAT :  654
Floradade.  : GTTATAGAATTGACAGTTAGTAGAATGGCCCTATAAAAATCTTATTACCCTGATAGATTAATGGCTCAGATGTAACAAAGTAGAAAATTATAAACTGCTATACATGCATGGACAATTGACCTAGCGATTAT :  654
LYC1410.3   : GTTATAGAATTGACAGTTAGTAGAATGGCCCTATAAAAATCTTATTACCCTGATAGATTAATGGCTCAGATGTAACAAAGTAGAAAATTATAAACTGCTATACATGCATGGACAATTGACCTAGCGATTAT :  654
PI169588.3  : GTTATAGAATTGACAGTTAGTAGAATGGCCCTATAAAAATCTTATTACCCTGATAGATTAATGGCTCAGATGTAACAAAGTAGAAAATTATAAACTGCTATACATGCATGGACAATTGACCTAGCGATTAT :  654
Brandywine  : GTTATAGAATTGACAGTTAGTAGAATGGCCCTATAAAAATCTTATTACCCTGATAGATTAATGGCTCAGATGTAACAAAGTAGAAAATTATAAACTGCTATACATGCATGGACAATTGACCTAGCGATTAT :  654
EA00990.3   : GTTATAGAATTGACAGTTAGTAGAATGGCCCTATAAAAATCTTATTACCCTGATAGATTAATGGCTCAGATGTAACAAAGTAGAAAATTATAAACTGCTATACATGCATGGACAATTGACCTAGCGATTAT :  654
PI303721.3  : GTTATAGAATTGACAGTTAGTAGAATGGCCCTATAAAAATCTTATTACCCTGATAGATTAATGGCTCAGATGTAACAAAGTAGAAAATTATAAACTGCTATACATGCATGGACAATTGACCTAGCGATTAT :  654
BGV006775.  : GTTATAGAATTGACAGTTAGTAGAATGGCCCTATAAAAATCTTATTATCCTGATAGATTAATGGCTCAGATGTAACAAAGTAGAAAATTATAAACTGCTATACATGCATGGACAATTGACCTAGCGATTAT :  654
PAS014479.  : GTTATAGAATTGACAGTTAGTAGAATGGCCCTATAAAAATCTTATTATCCTGATAGATTAATGGCTCAGATGTAACAAAGCAGAAAATTATAAACTGCTATACATGCATGGACAATTGACCTAGCGATTAT :  654
              GTTATAGAATTGACAGTTAGTAGAATGGCCCTATAAAAATCTTATTAcCCTGATAGATTAATGGCTCAGATGTAACAAAGtAGAAAATTATAAACTGCTATACATGCATGGACAATTGACCTAGCGATTAT
                                                             epm_Soly_M0_p0m16F_179 711
                                                                                        epm_Soly_M0_p0m00F_5096 730
                  660         *       680         *       700         *       720         *       740         *       760         *       780
ITAG.3      : TTTTCTGTCCATTTGATATTTAGTCACTAAGGCTATATAATTCAAAGCAAAAGTCTTCCAACCCAAAGCTGGATAAACAATACACATAAACAAAAATTATATAATGAATTGAAATGGAGCAAGTATTATTT :  785
BGV006865.  : TTTTCTGTCCATTTGATATTTAGTCACTAAGGCTATATAATTCAAAGCAAAAGTCTTCCAACCCAAAGCTGGATAAACACTACACATAAACAAAAATTATATAATGAATTGAAATGGAGCAAGTATTATTT :  785
BGV007931.  : TTTTCTGTCCATTTGATATTTAGTCACTAAGGCTATATAATTCAAAGCAAAAGTCTTCCAACCCAAAGCTGGATAAACAATACACATAAACAAAAATTATATAATGAATTGAAATGGAGCAAGTATTATTT :  785
BGV007989.  : TTTTCTGTCCATTTGATATTTAGTCACTAAGGCTATATAATTCAAAGCAAAAGTCTTCCAACCCAAAGCTGGATAAACAATACACATAAACAAAAATTATATAATGAATTGAAATGGAGCAAGTATTATTT :  785
M82.3       : TTTTCTGTCCATTTGATATTTAGTCACTAAGGCTATATAATTCAAAGCAAAAGTCTTCCAACCCAAAGCTGGATAAACAATACACATAAACAAAAATTATATAATGAATTGAAATGGAGCAAGTATTATTT :  785
EA00371.3   : TTTTCTGTCCATTTGATATTTAGTCACTAAGGCTATATAATTCAAAGCAAAAGTCTTCCAACCCAAAGCTGGATAAACAATACACATAAACAAAAATTATATAATGAATTGAAATGGAGCAAGTATTATTT :  785
Fla.8924.3  : TTTTCTGTCCATTTGATATTTAGTCACTAAGGCTATATAATTCAAAGCAAAAGTCTTCCAACCCAAAGCTGGATAAACAATACACATAAACAAAAATTATATAATGAATTGAAATGGAGCAAGTATTATTT :  785
Floradade.  : TTTTCTGTCCATTTGATATTTAGTCACTAAGGCTATATAATTCAAAGCAAAAGTCTTCCAACCCAAAGCTGGATAAACAATACACATAAACAAAAATTATATAATGAATTGAAATGGAGCAAGTATTATTT :  785
LYC1410.3   : TTTTCTGTCCATTTGATATTTAGTCACTAAGGCTATATAATTCAAAGCAAAAGTCTTCCAACCCAAAGCTGGATAAACAATACACATAAACAAAAATTATATAATGAATTGAAATGGAGCAAGTATTATTT :  785
PI169588.3  : TTTTCTGTCCATTTGATATTTAGTCACTAAGGCTATATAATTCAAAGCAAAAGTCTTCCAACCCAAAGCTGGATAAACAATACACATAAACAAAAATTATATAATGAATTGAAATGGAGCAAGTATTATTT :  785
Brandywine  : TTTTCTGTCCATTTGATATTTAGTCACTAAGGCTATATAATTCAAAGCAAAAGTCTTCCAACCCAAAGCTGGATAAACAATACACATAAACAAAAATTATATAATGAATTGAAATGGAGCAAGTATTATTT :  785
EA00990.3   : TTTTCTGTCCATTTGATATTTAGTCACTAAGGCTATATAATTCAAAGCAAAAGTCTTCCAACCCAAAGCTGGATAAACAATACACATAAACAAAAATTATATAATGAATTGAAATGGAGCAAGTATTATTT :  785
PI303721.3  : TTTTCTGTCCATTTGATATTTAGTCACTAAGGCTATATAATTCAAAGCAAAAGTCTTCCAACCCAAAGCTGGATAAACAATACACATAAACAAAAATTATATAATGAATTGAAATGGAGCAAGTATTATTT :  785
BGV006775.  : TTTTCTGTCCATTTGATATTTAGTCACTAAGGCTATATAATTCAAAGCAAAAGTCTTCCAACCCAAAGCTGGATAAACAATACACATCAACAAAAATTATATAATGAATTGAAATGGAGCAAGTATTATTT :  785
```

```
PAS014479. : TTTTCTGTCCATTTGATATTTAGTCACTAAGGCTATATAATTCAAAGCAAAAGTCTTCCAACCCAAAGCTGGATAAACAATACACATCAACAAAAATTATATAATGAATTGAAATGGAGCAAGTATTATTT :  785
             TTTTCTGTCCATTTGATATTTAGTCACTAAGGCTATATAATTCAAAGCAAAAGTCTTCCAACCCAAAGCTGGATAAACAaTACACATaAACAAAAATTATATAATGAATTGAAATGGAGCAAGTATTATTT
               epm_Soly_M0_p0m14R_211 790                                                                             epm_Soly_M0_p0m06F_537 890
               *        800         *        820         *        840         *        860         *        880         *        900         *
ITAG.3     : TTGAGATTTTGATCATTTTGTTCTTTAATGCTAAAAGAATACTCTAATCTTGAATTTTTATATTAAAGTAGTAATAATCAAAATGGAATAAAAAGAGTAACAGTAGGAAGAAGAGATAATATTTATTATTC :  916
BGV006865. : TTGAGATTTTGATCATTTTGTTCTTTAATGCTAAAAGAATACTCTAATCTTGAATTTTTATATTAAAGTAGTAATAATCAAAATGGAATAAAAAGAGTAACAGTAGGAAGAAGAGATAATATTTATTATTC :  916
BGV007931. : TTGAGATTTTGATCATTTTGTTCTTTAATGCTAAAAGAATACTCTAATCTTGAATTTTTATATTAAAGTAGTAATAATCAAAATGGAATAAAAAGAGTAACAGTAGGAAGAAGAGATAATATTTATTATTC :  916
BGV007989. : TTGAGATTTTGATCATTTTGTTCTTTAATGCTAAAAGAATACTCTAATCTTGAATTTTTATATTAAAGTAGTAATAATCAAAATGGAATAAAAAGAGTAACAGTAGGAAGAAGAGATAATATTTATTATTC :  916
M82.3      : TTGAGATTTTGATCATTTTGTTCTTTAATGCTAAAAGAATACTCTAATCTTGAATTTTTATATTAAAGTAGTAATAATCAAAATGGAATAAAAAGAGTAACAGTAGGAAGAAGAGATAATATTTATTATTC :  916
EA00371.3  : TTGAGATTTTGATCATTTTGTTCTTTAATGCTAAAAGAATACTCTAATCTTGAATTTTTATATTAAAGTAGTAATAATCAAAATGGAATAAAAAGAGTAACAGTAGGAAGAAGAGATAATATTTATTATTC :  916
Fla.8924.3 : TTGAGATTTTGATCATTTTGTTCTTTAATGCTAAAAGAATACTCTAATCTTGAATTTTTATATTAAAGTAGTAATAATCAAAATGGAATAAAAAGAGTAACAGTAGGAAGAAGAGATAATATTTATTATTC :  916
Floradade. : TTGAGATTTTGATCATTTTGTTCTTTAATGCTAAAAGAATACTCTAATCTTGAATTTTTATATTAAAGTAGTAATAATCAAAATGGAATAAAAAGAGTAACAGTAGGAAGAAGAGATAATATTTATTATTC :  916
LYC1410.3  : TTGAGATTTTGATCATTTTGTTCTTTAATGCTAAAAGAATACTCTAATCTTGAATTTTTATATTAAAGTAGTAATAATCAAAATGGAATAAAAAGAGTAACAGTAGGAAGAAGAGATAATATTTATTATTC :  916
PI169588.3 : TTGAGATTTTGATCATTTTGTTCTTTAATGCTAAAAGAATACTCTAATCTTGAATTTTTATATTAAAGTAGTAATAATCAAAATGGAATAAAAAGAGTAACAGTAGGAAGAAGAGATAATATTTATTATTC :  916
Brandywine : TTGAGATTTTGATCATTTTGTTCTTTAATGCTAAAAGAATACTCTAATCTTGAATTTTTATATTAAAGTAGTAATAATCAAAATGGAATAAAAAGAGTAACAGTAGGAAGAAGAGATAATATTTATTATTC :  916
EA00990.3  : TTGAGATTTTGATCATTTTGTTCTTTAATGCTAAAAGAATACTCTAATCTTGAATTTTTATATTAAAGTAGTAATAATCAAAATGGAATAAAAAGAGTAACAGTAGGAAGAAGAGATAATATTTATTATTC :  916
PI303721.3 : TTGAGATTTTGATCATTTTGTTCTTTAATGCTAAAAGAATACTCTAATCTTGAATTTTTATATTAAAGTAGTAATAATCAAAATGGAATAAAAAGAGTAACAGTAGGAAGAAGAGATAATATTTATTATTC :  916
BGV006775. : TTGAGATTTTGATCATTTTGTTCTTTAATGCTAAAAGAATACTCTAATCTTGAATTTTTATATTAAAGTAGTAATAATCAAAATGGAATAAAAAGAGTAACAGTAGGAAGAAGAGATAATATTTATTATTC :  916
PAS014479. : TTGAGATTTTGATCATTTTGTTCTTTAATGCTAAAAGAATACTCTAATCTTGAATTTTTATATTAAAGTAGTAATAATCAAAATGGAATAAAAAGAGTAACAGTAGGAAGAAGAGATAATATTTATTATTC :  916
             TTGAGATTTTGATCATTTTGTTCTTTAATGCTAAAAGAATACTCTAATCTTGAATTTTTATATTAAAGTAGTAATAATCAAAATGGAATAAAAAGAGTAACAGTAGGAAGAAGAGATAATATTTATTATTC

             920         *        940         *        960         *        980         *       1000         *       1020         *       1040
ITAG.3     : CTATATATGAAATCATATTCAAAATGGACGGGAGAAATTAAGCATTTTTTTCTATTAATAAAGCAAAAGCGTGCTATTATTCTTTATGCCTATTTCTCTGAGCTCGAAAGAGAAATACTAGCTACCACTTC : 1047
BGV006865. : CTATATATGAAATCATATTCAAAATGGACGGGAGAAATTAAGCATTTTTTTCTATTAATAAAGCAAAAGCGTGCTATTATTCTTTATGCCTATTTCTCTGAGCTCGAAAGAGAAATACTAGCTACCACTTC : 1047
BGV007931. : CTATATATGAAATCATATTCAAAATGGACGGGAGAAATTAAGCATTTTTTTCTATTAATAAAGCAAAAGCGTGCTATTATTCTTTATGCCTATTTCTCTGAGCTCGAAAGAGAAATACTAGCTACCACTTC : 1047
BGV007989. : CTATATATGAAATCATATTCAAAATGGACGGGAGAAATTAAGCATTTTTTTCTATTAATAAAGCAAAAGCGTGCTATTATTCTTTATGCCTATTTCTCTGAGCTCGAAAGAGAAATACTAGCTACCACTTC : 1047
M82.3      : CTATATATGAAATCATATTCAAAATGGACGGGAGAAATTAAGCATTTTTTTCTATTAATAAAGCAAAAGCGTGCTATTATTCTTTATGCCTATTTCTCTGAGCTCGAAAGAGAAATACTAGCTACCACTTC : 1047
EA00371.3  : CTATATATGAAATCATATTCAAAATGGACGGGAGAAATTAAGCATTTTTTTCTATTAATAAAGCAAAAGCGTGCTATTATTCTTTATGCCTATTTCTCTGAGCTCGAAAGAGAAATACTAGCTACCACTTC : 1047
Fla.8924.3 : CTATATATGAAATCATATTCAAAATGGACGGGAGAAATTAAGCATTTTTTTCTATTAATAAAGCAAAAGCGTGCTATTATTCTTTATGCCTATTTCTCTGAGCTCGAAAGAGAAATACTAGCTACCACTTC : 1047
Floradade. : CTATATATGAAATCATATTCAAAATGGACGGGAGAAATTAAGCATTTTTTTCTATTAATAAAGCAAAAGCGTGCTATTATTCTTTATGCCTATTTCTCTGAGCTCGAAAGAGAAATACTAGCTACCACTTC : 1047
LYC1410.3  : CTATATATGAAATCATATTCAAAATGGACGGGAGAAATTAAGCATTTTTTTCTATTAATAAAGCAAAAGCGTGCTATTATTCTTTATGCCTATTTCTCTGAGCTCGAAAGAGAAATACTAGCTACCACTTC : 1047
PI169588.3 : CTATATATGAAATCATATTCAAAATGGACGGGAGAAATTAAGCATTTTTTTCTATTAATAAAGCAAAAGCGTGCTATTATTCTTTATGCCTATTTCTCTGAGCTCGAAAGAGAAATACTAGCTACCACTTC : 1047
Brandywine : CTATATATGAAATCATATTCAAAATGGACGGGAGAAATTAAGCATTTTTTTCTATTAATAAAGCAAAAGCGTGCTATTATTCTTTATGCCTATTTCTCTGAGCTCGAAAGAGAAATACTAGCTACCACTTC : 1047
EA00990.3  : CTATATATGAAATCATATTCAAAATGGACGGGAGAAATTAAGCATTTTTTTCTATTAATAAAGCAAAAGCGTGCTATTATTCTTTATGCCTATTTCTCTGAGCTCGAAAGAGAAATACTAGCTACCACTTC : 1047
PI303721.3 : CTATATATGAAATCATATTCAAAATGGACGGGAGAAATTAAGCATTTTTTTCTATTAATAAAGCAAAAGCGTGCTATTATTCTTTATGCCTATTTCTCTGAGCTCGAAAGAGAAATACTAGCTACCACTTC : 1047
BGV006775. : CTATATATGAAATCATATTCAAAATGGACGGGAGAAATTAAGCATTTTTTTCTATTAATAAAGCAAAAGCGTGCTATTATTCTTTATGCCTATTTCTCTGAGCTCGAAAGAGAAATACTAGCTACCACTTC : 1047
PAS014479. : CTATATATGAAATCATATTCAAAATGGACGGGAGAAATTAAGCATTTTTTTCTATTAATAAAGCAAAAGCGTGCTATTATTCTTTATGCCTATTTCTCTGAGCTTGAAAGAGTAATACTAGCTACCACTTC : 1047
             CTATATATGAAATCATATTCAAAATGGACGGGAGAAATTAAGCATTTTTTTCTATTAATAAAGCAAAAGCGTGCTATTATTCTTTATGCCTATTTCTCTGAGCTcGAAAGAGaAATACTAGCTACCACTTC
           epm_Soly_M0_p0m04R_584 1046                     epm_Soly_M0_p0m18R_163 1093
              *       1060         *       1080         *       1100         *       1120         *       1140         *       1160         *        118
ITAG.3     : TCTCTATTTTTCCTGCAATGTTTTGTCGAATAGCAATTTGCATGTTGTTTTGTAGTTTCATAATATCATCATGTCATCAAACAGATATCCTTAATATTATCAAAAAAAATCCTAACCAAGTTTCAACTTCA : 1178
BGV006865. : TCTCTATGCTTCCTGCAATGTTTTGTCGAATAGCAATTTGCATGTTGTTTTGTAGTTTCATAATATCATCATGTCATCAAACAGATATCCTTAATATTATCAAAAAAAATCCTAACCAAGTTTCAACTTCA : 1178
BGV007931. : TCTCTATTTTTCCTGCAATGTTTTGTCGAATAGCAATTTGCATGTTGTTTTGTAGTTTCATAATATCATCATGTCATCAAACAGATATCCTTAATATTATCAAAAAAAATCCTAACCAAGTTTCAACTTCA : 1178
BGV007989. : TCTCTATTTTTCCTGCAATGTTTTGTCGAATAGCAATTTGCATGTTGTTTTGTAGTTTCATAATATCATCATGTCATCAAACAGATATCCTTAATATTATCAAAAAAAATCCTAACCAAGTTTCAACTTCA : 1178
M82.3      : TCTCTATTTTTCCTGCAATGTTTTGTCGAATAGCAATTTGCATGTTGTTTTGTAGTTTCATAATATCATCATGTCATCAAACAGATATCCTTAATATTATCAAAAAAAATCCTAACCAAGTTTCAACTTCA : 1178
EA00371.3  : TCTCTATTTTTCCTGCAATGTTTTGTCGAATAGCAATTTGCATGTTGTTTTGTAGTTTCATAATATCATCATGTCATCAAACAGATATCCTTAATATTATCAAAAAAAATCCTAACCAAGTTTCAACTTCA : 1178
Fla.8924.3 : TCTCTATTTTTCCTGCAATGTTTTGTCGAATAGCAATTTGCATGTTGTTTTGTAGTTTCATAATATCATCATGTCATCAAACAGATATCCTTAATATTATCAAAAAAAATCCTAACCAAGTTTCAACTTCA : 1178
Floradade. : TCTCTATTTTTCCTGCAATGTTTTGTCGAATAGCAATTTGCATGTTGTTTTGTAGTTTCATAATATCATCATGTCATCAAACAGATATCCTTAATATTATCAAAAAAAATCCTAACCAAGTTTCAACTTCA : 1178
LYC1410.3  : TCTCTATTTTTCCTGCAATGTTTTGTCGAATAGCAATTTGCATGTTGTTTTGTAGTTTCATAATATCATCATGTCATCAAACAGATATCCTTAATATTATCAAAAAAAATCCTAACCAAGTTTCAACTTCA : 1178
PI169588.3 : TCTCTATTTTTCCTGCAATGTTTTGTCGAATAGCAATTTGCATGTTGTTTTGTAGTTTCATAATATCATCATGTCATCAAACAGATATCCTTAATATTATCAAAAAAAATCCTAACCAAGTTTCAACTTCA : 1178
Brandywine : TCTCTATTTTTCCTGCAATGTTTTGTCGAATAGCAATTTGCATGTTGTTTTGTAGTTTCATAATATCATCATGTCATCAAACAGATATCCTTAATATTATCAAAAAAAATCCTAACCAAGTTTCAACTTCA : 1178
EA00990.3  : TCTCTATTTTTCCTGCAATGTTTTGTCGAATAGCAATTTGCATGTTGTTTTGTAGTTTCATAATATCATCATGTCATCAAACAGATATCCTTAATATTATCAAAAAAAATCCTAACCAAGTTTCAACTTCA : 1178
PI303721.3 : TCTCTATTTTTCCTGCAATGTTTTGTCGAATAGCAATTTGCATGTTGTTTTGTAGTTTCATAATATCATCATGTCATCAAACAGATATCCTTAATATTATCAAAAAAAATCCTAACCAAGTTTCAACTTCA : 1178
BGV006775. : TCTCTATTTTTCCTGCAATGTTTTGTCGAATAGCAATTTGCATGTTGTTTTGTAGTTTCATAATATCATCATGTCATCAAACAGATATCCTTAATATTATCAAAAAAAATCCTAACCAAGTTTCAACTTCA : 1178
PAS014479. : TCTCTATTTTTCCTGCAATGTTTTGTCGAATAGCAATTTGCATGTTGTTTTGTAGTTTCATAATATCATCATGTCATCAAACAGATATCCTTAATATTATCAAAAAAAATCCTAACCAAGTTTCAACTTCA : 1178
             TCTCTATttTTCCTGCAATGTTTTGTCGAATAGCAATTTGCATGTTGTTTTGTAGTTTCATAATATCATCATGTCATCAAACAGATATCCTTAATATTATCAAAAAAAATCCTAACCAAGTTTCAACTTCA
                                                                                             epm_Soly_M0_p0m16F_179 1242
             0         *       1200         *       1220         *       1240         *       1260         *       1280         *       1300         *
ITAG.3     : AGCAATTTCTTATTTGGAACATCCTCTTCCTGTTACCAGGTATGTACTTCCTACACTTGATTT-TTTTGGGGGGTCTGAATTCTTGTTATGAGTAC--AATTTTTTTTTGTCCGCAGTATGAAGGAGCTA : 1306
BGV006865. : AGCAATTTCTTATTTGGAACATCCTCTTCCTGTTACCAGGTATGTACTTCCTACACTTGTTTTCTTTTTGGGGG------------CCTATGAGTACAAAAAAAATTTTGTCCGCAGTATGAAGGAGCTA : 1297
BGV007931. : AGCAATTTCTTATTTGGAACATCCTCTTCCTGTTACCAGGTATGTACTTCCTACACTTGATTTTTTTTGGGGGGTCTGAATTCTTGTTATGAGTAC--AATTTTTTTTTGTCCGCAGTATGAAGGAGCTA : 1307
BGV007989. : AGCAATTTCTTATTTGGAACATCCTCTTCCTGTTACCAGGTATGTACTTCCTACACTTGATTTTTTTTGGGGGGTCTGAATTCTTGTTATGAGTAC--AATTTTTTTTTGTCCGCAGTATGAAGGAGCTA : 1307
M82.3      : AGCAATTTCTTATTTGGAACATCCTCTTCCTGTTACCAGGTATGTACTTCCTACACTTGATTTTTTTTGGGGGGTCTGAATTCTTGTTATGAGTAC--AATTTTTTTTTGTCCGCAGTATGAAGGAGCTA : 1307
EA00371.3  : AGCAATTTCTTATTTGGAACATCCTCTTCCTGTTACCAGGTATGTACTTCCTACACTTGATTTTTTTTGGGGGGTCTGAATTCTTGTTATGAGTAC--AATTTTTTTTTGTCCGCAGTATGAAGGAGCTA : 1307
Fla.8924.3 : AGCAATTTCTTATTTGGAACATCCTCTTCCTGTTACCAGGTATGTACTTCCTACACTTGATTTTTTTTGGGGGGTCTGAATTCTTGTTATGAGTAC--AATTTTTTTTTGTCCGCAGTATGAAGGAGCTA : 1307
Floradade. : AGCAATTTCTTATTTGGAACATCCTCTTCCTGTTACCAGGTATGTACTTCCTACACTTGATTTTTTTTGGGGGGTCTGAATTCTTGTTATGAGTAC--AATTTTTTTTTGTCCGCAGTATGAAGGAGCTA : 1307
LYC1410.3  : AGCAATTTCTTATTTGGAACATCCTCTTCCTGTTACCAGGTATGTACTTCCTACACTTGATTTTTTTTGGGGGGTCTGAATTCTTGTTATGAGTAC--AATTTTTTTTTGTCCGCAGTATGAAGGAGCTA : 1307
PI169588.3 : AGCAATTTCTTATTTGGAACATCCTCTTCCTGTTACCAGGTATGTACTTCCTACACTTGATTTTTTTTGGGGGGTCTGAATTCTTGTTATGAGTAC--AATTTTTTTTTGTCCGCAGTATGAAGGAGCTA : 1307
Brandywine : AGCAATTTCTTATTTGGAACATCCTCTTCCTGTTACCAGGTATGTACTTCCTACACTTGATTTTTTTTGGGGGGTCTGAATTCTTGTTATGAGTAC--AATTTTTTTTTGTCCGCAGTATGAAGGAGCTA : 1307
EA00990.3  : AGCAATTTCTTATTTGGAACATCCTCTTCCTGTTACCAGGTATGTACTTCCTACACTTGATTTTTTTTGGGGGGTCTGAATTCTTGTTATGAGTAC--AATTTTTTTTTGTCCGCAGTATGAAGGAGCTA : 1307
PI303721.3 : AGCAATTTCTTATTTGGAACATCCTCTTCCTGTTACCAGGTATGTACTTCCTACACTTGATTTTTTTTGGGGGGTCTGAATTCTTGTTATGAGTAC--AATTTTTTTTTGTCCGCAGTATGAAGGAGCTA : 1307
BGV006775. : AGCAATTTCTTATTTGGAACATCCTCTTCCTGTTACCAGGTATGTACTTCCTACACTTGTTTTCTTTTTGGGGG------------CCTATGAGTACAAAAAAATTTTTTGTCCGCAGTATGAAGGAGCTA : 1297
PAS014479. : AGCAATTTCTTATTTGGAACATCCTCTTCCTGTTACCAGGTACGTACTTCCTACACTTGTTTTCTTTTTGGGGG------------CTTATGAGTACAAAAAAAAATTTTGTCCGCAGTATGAAGGAGCTA : 1297
             AGCAATTTCTTATTTGGAACATCCTCTTCCTGTTACCAGGTAtGTACTTCCTACACTTGaTTT TTTTgGGGGGgtctgaattcttgtTATGAGTAC  AAtttttTTTTGTCCGCAGTATGAAGGAGCTA
             epm_Soly_M0_p1m03R_719 1316                          epm_Soly_M0_p1m01R_2282 1355
                 1320         *       1340         *       1360         *       1380         *       1400         *       1420         *       1440
ITAG.3     : TTCTCAGTGATGGAAAAGGTCTCAACAATTGGGATGTTTTTACTCACGAAGCTGGTGAGCTTATAAAAAAAATTAATGAATCTCAATTTCTTTTTAAAATTTATTAATTTCGGTTAGTGTTGTTGCAGGTCA : 1437
BGV006865. : TTCTCAGTGATGGAAAAGGTCTCAACAATTGGGATGTTTTTACTCACGAAGCTGGTGAGCTTATAAAAAAAATTAATGAATCTCAATTTCTTTTTAAAATTTATTAATTTCGGTTAGTGTTGTTGCAGGTCA : 1428
BGV007931. : TTCTCAGTGATGGAAAAGGTCTCAACAATTGGGATGTTTTTACTCACGAAGCTGGTGAGCTTATAAAAAAAATTAATGAATCTCAATTTCTTTTTAAAATTTATTAATTTCGGTTAGTGTTGTTGCAGGTCA : 1438
BGV007989. : TTCTCAGTGATGGAAAAGGTCTCAACAATTGGGATGTTTTTACTCACGAAGCTGGTGAGCTTATAAAAAAAATTAATGAATCTCAATTTCTTTTTAAAATTTATTAATTTCGGTTAGTGTTGTTGCAGGTCA : 1438
M82.3      : TTCTCAGTGATGGAAAAGGTCTCAACAATTGGGATGTTTTTACTCACGAAGCTGGTGAGCTTATAAAAAAAATTAATGAATCTCAATTTCTTTTTAAAATTTATTAATTTCGGTTAGTGTTGTTGCAGGTCA : 1438
EA00371.3  : TTCTCAGTGATGGAAAAGGTCTCAACAATTGGGATGTTTTTACTCACGAAGCTGGTGAGCTTATAAAAAAAATTAATGAATCTCAATTTCTTTTTAAAATTTATTAATTTCGGTTAGTGTTGTTGCAGGTCA : 1438
Fla.8924.3 : TTCTCAGTGATGGAAAAGGTCTCAACAATTGGGATGTTTTTACTCACGAAGCTGGTGAGCTTATAAAAAAAATTAATGAATCTCAATTTCTTTTTAAAATTTATTAATTTCGGTTAGTGTTGTTGCAGGTCA : 1438
Floradade. : TTCTCAGTGATGGAAAAGGTCTCAACAATTGGGATGTTTTTACTCACGAAGCTGGTGAGCTTATAAAAAAAATTAATGAATCTCAATTTCTTTTTAAAATTTATTAATTTCGGTTAGTGTTGTTGCAGGTCA : 1438
LYC1410.3  : TTCTCAGTGATGGAAAAGGTCTCAACAATTGGGATGTTTTTACTCACGAAGCTGGTGAGCTTATAAAAAAAATTAATGAATCTCAATTTCTTTTTAAAATTTATTAATTTCGGTTAGTGTTGTTGCAGGTCA : 1438
PI169588.3 : TTCTCAGTGATGGAAAAGGTCTCAACAATTGGGATGTTTTTACTCACGAAGCTGGTGAGCTTATAAAAAAAATTAATGAATCTCAATTTCTTTTTAAAATTTATTAATTTCGGTTAGTGTTGTTGCAGGTCA : 1438
Brandywine : TTCTCAGTGATGGAAAAGGTCTCAACAATTGGGATGTTTTTACTCACGAAGCTGGTGAGCTTATAAAAAAAATTAATGAATCTCAATTTCTTTTTAAAATTTATTAATTTCGGTTAGTGTTGTTGCAGGTCA : 1438
EA00990.3  : TTCTCAGTGATGGAAAAGGTCTCAACAATTGGGATGTTTTTACTCACGAAGCTGGTGAGCTTATAAAAAAAATTAATGAATCTCAATTTCTTTTTAAAATTTATTAATTTCGGTTAGTGTTGTTGCAGGTCA : 1438
PI303721.3 : TTCTCAGTGATGGAAAAGGTCTCAACAATTGGGATGTTTTTACTCACGAAGCTGGTGAGCTTATAAAAAAAATTAATGAATCTCAATTTCTTTTTAAAATTTATTAATTTCGGTTAGTGTTGTTGCAGGTCA : 1438
BGV006775. : TTCTCAGTGATGGAAAAGGTCTCAACAATTGGGATGTTTTTACTCACGAAGCTGGTGAGCTTATAAAAAAAATTAATGAATCTCAATTTCTTTTTAAAATTTATTAATTTCGGTTAGTGTTGTTGCAGGTCA : 1428
PAS014479. : TTCTCAGTGATGGAAAAGGTCTCAACAATTGGGATGTTTTTACTCACCAAGCTGGTCAGCTTATAAAAAAAATTAATGAATCTCAATTTCTTTTTAAAATTTATTAATTTTGGTTAGTGATGTTGCAGGTCA : 1428
             TTCTCAGTGATGGAAAAGGTCTCAACAATTGGGATGTTTTTACTCACgAAGCTGGTgAGCTTATAAAAAAAATTAATGAATCTCAATTTCTTTTTAAAATTTATTAATTTcGGTTAGTGtTGTTGCAGGTCA
                        epm_Soly_M0_p1m03R_719 1455                                                                        epm_Soly_M0_p1m07F_163 1551
                  *       1460         *       1480         *       1500         *       1520         *       1540         *       1560         *
ITAG.3     : TATTAAGGATGGAAGCAACGGAGATGTTGCTGTTGATCACTACAATCGTTATTTGGTAAACCTn---------nnnnnnnnnnnnnnnnnnCTGAATCCTTCAATAATAGACACCTTGAACGATTTTCAT : 1559
BGV006865. : TATTAAGGATGGAAGCGACGGAGATGTTGCTGTTGATCACTACAATCGTTATTTGGTAAACCTAATTCATCAnnnnnnnnnnnnnnnnnnnn---------------------------------------- : 1520
BGV007931. : TATTAAGGATGGAAGCAACGGAGATGTTGCTGTTGATCACTACAATCGTTATTTGGTAAACCnn----------nnnnnnnnnnnnnnnnnnCTGAATCCTTCAATAATAGACACCTTGAACGATTTTCAT : 1559
BGV007989. : TATTAAGGATGGAAGCAACGGAGATGTTGCTGTTGATCACTACAATCGTTATTTGGTAAACCnn----------nnnnnnnnnnnnnnnnnnCTGAATCCTTCAATAATAGACACCTTGAACGATTTTCAT : 1559
M82.3      : TATTAAGGATGGAAGCAACGGAGATGTTGCTGTTGATCACTACAATCGTTATTTGGTAAACCnn----------nnnnnnnnnnnnnnnnnnCTGAATCCTTCAATAATAGACACCTTGAACGATTTTCAT : 1559
EA00371.3  : TATTAAGGATGGAAGCAACGGAGATGTTGCTGTTGATCACTACAATCGTTATTTGGTAAACCnn----------nnnnnnnnnnnnnnnnnnCTGAATCCTTCAATAATAGACACCTTGAACGATTTTCAT : 1559
Fla.8924.3 : TATTAAGGATGGAAGCAACGGAGATGTTGCTGTTGATCACTACAATCGTTATTTGGTAAACCnn----------nnnnnnnnnnnnnnnnnnCTGAATCCTTCAATAATAGACACCTTGAACGATTTTCAT : 1559
Floradade. : TATTAAGGATGGAAGCAACGGAGATGTTGCTGTTGATCACTACAATCGTTATTTGGTAAACCnn----------nnnnnnnnnnnnnnnnnnCTGAATCCTTCAATAATAGACACCTTGAACGATTTTCAT : 1559
LYC1410.3  : TATTAAGGATGGAAGCAACGGAGATGTTGCTGTTGATCACTACAATCGTTATTTGGTAAACCnn----------nnnnnnnnnnnnnnnnnnCTGAATCCTTCAATAATAGACACCTTGAACGATTTTCAT : 1559
PI169588.3 : TATTAAGGATGGAAGCAACGGAGATGTTGCTGTTGATCACTACAATCGTTATTTGGTAAACCnn----------nnnnnnnnnnnnnnnnnnCTGAATCCTTCAATAATAGACACCTTGAACGATTTTCAT : 1559
Brandywine : TATTAAGGATGGAAGCAACGGAGATGTTGCTGTTGATCACTACAATCGTTATTTGGTAAACCnn----------nnnnnnnnnnnnnnnnnnCTGAATCCTTCAATAATAGACACCTTGAACGATTTTCAT : 1559
EA00990.3  : TATTAAGGATGGAAGCAACGGAGATGTTGCTGTTGATCACTACAATCGTTATTTGGTAAACCnn----------nnnnnnnnnnnnnnnnnnCTGAATCCTTCAATAATAGACACCTTGAACGATTTTCAT : 1559
PI303721.3 : TATTAAGGATGGAAGCAACGGAGATGTTGCTGTTGATCACTACAATCGTTATTTGGTAAACCnn----------nnnnnnnnnnnnnnnnnnCTGAATCCTTCAATAATAGACACCTTGAACGATTTTCAT : 1559
BGV006775. : TATTAAGGATGGAAGCAACGGAGATGTTGCTGTTGATCACTACAATCGTTATTTGGTAAACCTAATTCATCAnnnnnnnnnnnnnnnnnnnn---------------------------------------- : 1520
PAS014479. : TATTAAGGATGGAAGCAACGGAGATGTTGCTGTTGATCACTACAATCGTTATTTGGTAAACCTAATTCATCAnnnnnnnnnnnnnnnnnnnn---------------------------------------- : 1520
             TATTAAGGATGGAAGCaACGGAGATGTTGCTGTTGATCACTACAATCGTTATTTGGTAAACC n          NNNNNNNNNNNNNNNNNNctgaatccttcaataatagacaccttgaacgattttcat
                                                              epm_Soly_M0_p1m04R_296 1624
               1580         *       1600         *       1620         *       1640         *       1660         *       1680         *       1700
ITAG.3     : AGAGTGGACTATATGCGTAGCTACTTAAATTCATTGGCAAATGCAATCACGGATGGTGCAGATGTGAGGGGGTACTTTGTTTGGTCCCTTCTCGACAACTTTGAGTGGCTAGATGGATATAAGTTAAGATT : 1690
BGV006865. : -----------------------------------------------------------------GAGGGGGTACTTTGTTTGGTCCCTTCTCGACAACTTTGAGTGGCTAGATGGATATAAGTTAAGATT : 1586
BGV007931. : AGAGTGGACTATATGCGTAGCTACTTAAATTCATTGGCAAATGCAATCACGGATGGTGCAGATGTGAGGGGGTACTTTGTTTGGTCCCTTCTCGACAACTTTGAGTGGCTAGATGGATATAAGTTAAGATT : 1690
BGV007989. : AGAGTGGACTATATGCGTAGCTACTTAAATTCATTGGCAAATGCAATCACGGATGGTGCAGATGTGAGGGGGTACTTTGTTTGGTCCCTTCTCGACAACTTTGAGTGGCTAGATGGATATAAGTTAAGATT : 1690
M82.3      : AGAGTGGACTATATGCGTAGCTACTTAAATTCATTGGCAAATGCAATCACGGATGGTGCAGATGTGAGGGGGTACTTTGTTTGGTCCCTTCTCGACAACTTTGAGTGGCTAGATGGATATAAGTTAAGATT : 1690
EA00371.3  : AGAGTGGACTATATGCGTAGCTACTTAAATTCATTGGCAAATGCAATCACGGATGGTGCAGATGTGAGGGGGTACTTTGTTTGGTCCCTTCTCGACAACTTTGAGTGGCTAGATGGATATAAGTTAAGATT : 1690
Fla.8924.3 : AGAGTGGACTATATGCGTAGCTACTTAAATTCATTGGCAAATGCAATCACGGATGGTGCAGATGTGAGGGGGTACTTTGTTTGGTCCCTTCTCGACAACTTTGAGTGGCTAGATGGATATAAGTTAAGATT : 1690
Floradade. : AGAGTGGACTATATGCGTAGCTACTTAAATTCATTGGCAAATGCAATCACGGATGGTGCAGATGTGAGGGGGTACTTTGTTTGGTCCCTTCTCGACAACTTTGAGTGGCTAGATGGATATAAGTTAAGATT : 1690
LYC1410.3  : AGAGTGGACTATATGCGTAGCTACTTAAATTCATTGGCAAATGCAATCACGGATGGTGCAGATGTGAGGGGGTACTTTGTTTGGTCCCTTCTCGACAACTTTGAGTGGCTAGATGGATATAAGTTAAGATT : 1690
PI169588.3 : AGAGTGGACTATATGCGTAGCTACTTAAATTCATTGGCAAATGCAATCACGGATGGTGCAGATGTGAGGGGGTACTTTGTTTGGTCCCTTCTCGACAACTTTGAGTGGCTAGATGGATATAAGTTAAGATT : 1690
```

```
Brandywine : AGAGTGGACTATATGCGTAGCTACTTAAATTCATTGGCAAATGCAATCACGGATGGTGCAGATGTGAGGGGGTACTTTGTTTGGTCCCTTCTCGACAACTTTGAGTGGCTAGATGGATATAAGTTAAGATT : 1690
EA00990.3  : AGAGTGGACTATATGCGTAGCTACTTAAATTCATTGGCAAATGCAATCACGGATGGTGCAGATGTGAGGGGGTACTTTGTTTGGTCCCTTCTCGACAACTTTGAGTGGCTAGATGGATATAAGTTAAGATT : 1690
PI303721.3 : AGAGTGGACTATATGCGTAGCTACTTAAATTCATTGGCAAATGCAATCACGGATGGTGCAGATGTGAGGGGGTACTTTGTTTGGTCCCTTCTCGACAACTTTGAGTGGCTAGATGGATATAAGTTAAGATT : 1690
BGV006775. : -----------------------------------------------------------------GAGGGGGTACTTTGTTTGGTCCCTTCTCGACAACTTTGAGTGGCTAGATGGATATAAGTTAAGATT : 1586
PAS014479. : -----------------------------------------------------------------GAGGGGGTACTTTGTTTGGTCCCTTCTCGACAACTTTGAGTGGCTAGATGGATATAAGTTAAGATT : 1586
             agagtggactatatgcgtagctacttaaattcattggcaaatgcaatcacggatggtgcagatgtGAGGGGGTACTTTGTTTGGTCCCTTCTCGACAACTTTGAGTGGCTAGATGGATATAAGTTAAGATT
```

epm_Soly_M0_p1m08R_108 1828

```
                    *      1720          *      1740          *      1760          *      1780          *      1800          *      1820          *
ITAG.3     : TGGACTTCACTATGTCAACTATACTAATCTTCAGAGAACCCCAAAATTATCAGCTACCAGGTATAAACAGCTCATGTATAACTTCAACATACAACTTGAAAAAAATACTGCCCAGAACTAGTGGCCGGGAG : 1821
BGV006865. : TGGACTTCACTATGTCAACTATACTAATCTTCAGAGAACCCCAAAATTATCAGCTACCAGATATAAACAGCTCATGTATAACTTCAAAAAACAGCTTGAAACACATGCTGCCCAGGAGTAGTGGCCGGGAG : 1717
BGV007931. : TGGACTTCACTATGTCAACTATACTAATCTTCAGAGAACCCCAAAATTATCAGCTACCAGGTATAAACAGCTCATGTATAACTTCAACATACAACTTGAAAAAAATACTGCCCAGAACTAGTGGCCGGGAG : 1821
BGV007989. : TGGACTTCACTATGTCAACTATACTAATCTTCAGAGAACCCCAAAATTATCAGCTACCAGGTATAAACAGCTCATGTATAACTTCAACATACAACTTGAAAAAAATACTGCCCAGAACTAGTGGCCGGGAG : 1821
M82.3      : TGGACTTCACTATGTCAACTATACTAATCTTCAGAGAACCCCAAAATTATCAGCTACCAGGTATAAACAGCTCATGTATAACTTCAACATACAACTTGAAAAAAATACTGCCCAGAACTAGTGGCCGGGAG : 1821
EA00371.3  : TGGACTTCACTATGTCAACTATACTAATCTTCAGAGAACCCCAAAATTATCAGCTACCAGGTATAAACAGCTCATGTATAACTTCAACATACAACTTGAAAAAAATACTGCCCAGAACTAGTGGCCGGGAG : 1821
Fla.8924.3 : TGGACTTCACTATGTCAACTATACTAATCTTCAGAGAACCCCAAAATTATCAGCTACCAGGTATAAACAGCTCATGTATAACTTCAACATACAACTTGAAAAAAATACTGCCCAGAACTAGTGGCCGGGAG : 1821
Floradade. : TGGACTTCACTATGTCAACTATACTAATCTTCAGAGAACCCCAAAATTATCAGCTACCAGGTATAAACAGCTCATGTATAACTTCAACATACAACTTGAAAAAAATACTGCCCAGAACTAGTGGCCGGGAG : 1821
LYC1410.3  : TGGACTTCACTATGTCAACTATACTAATCTTCAGAGAACCCCAAAATTATCAGCTACCAGGTATAAACAGCTCATGTATAACTTCAACATACAACTTGAAAAAAATACTGCCCAGAACTAGTGGCCGGGAG : 1821
PI169588.3 : TGGACTTCACTATGTCAACTATACTAATCTTCAGAGAACCCCAAAATTATCAGCTACCAGGTATAAACAGCTCATGTATAACTTCAACATACAACTTGAAAAAAATACTGCCCAGAACTAGTGGCCGGGAG : 1821
Brandywine : TGGACTTCACTATGTCAACTATACTAATCTTCAGAGAACCCCAAAATTATCAGCTACCAGGTATAAACAGCTCATGTATAACTTCAACATACAACTTGAAAAAAATACTGCCCAGAACTAGTGGCCGGGAG : 1821
EA00990.3  : TGGACTTCACTATGTCAACTATACTAATCTTCAGAGAACCCCAAAATTATCAGCTACCAGGTATAAACAGCTCATGTATAACTTCAACATACAACTTGAAAAAAATACTGCCCAGAACTAGTGGCCGGGAG : 1821
PI303721.3 : TGGACTTCACTATGTCAACTATACTAATCTTCAGAGAACCCCAAAATTATCAGCTACCAGGTATAAACAGCTCATGTATAACTTCAACATACAACTTGAAAAAAATACTGCCCAGAACTAGTGGCCGGGAG : 1821
BGV006775. : TGGACTTCACTATGTCAACTATACTAATCTTCAGAGAACCCCAAAATTATCAGCTACCAGATATAAACAGCTCATGTATAACTTCAAAAAACAGCTTGAAACACATGCTGCCCAGGAGTAGTGGCCGGGAG : 1717
PAS014479. : TGGACTTCACTATGTCAACTATACTAATCTTCAGAGAACCCCAAAATTATCAGCTACCAGATATAAACAGCTCATGTATAACTTCAAAAAACAGCTTGAAACACATGCTGCCCAGGACTAGTGGCCGGGAG : 1717
             TGGACTTCACTATGTCAACTATACTAATCTTCAGAGAACCCCAAAATTATCAGCTACCAGgTATAAACAGCTCATGTATAACTTCAAcAtACAaCTTGAAAAaAaATaCTGCCCAGaAcTAGTGGCCGGGAG
```

epm_Soly_M0_p0m08R_491 1872

epm_Soly_M0_p0m15R_209 1872

epm_Soly_M0_p0m14R_211 1877

```
               1840          *      1860          *      1880          *      1900          *      1920          *      1940          *      1960
ITAG.3     : AAGACGATGAAAAGCGGAGGCTTCTTAAGATTATTTTCATTCTGTGTCTGTTGTTGGAAGACAGTTTATTGTGTAATGAAACAAATTAGTGGT-------------------------------------- : 1914
BGV006865. : AAGGCGATGAAAAGCGGAGGCTTCTTAAGATTATTTTCATTCTGTGTCTGTTGTTGGAAGACAGTTTATTGTGTAATGAAACAAATTAGTGGTCAGCCTTATCCCTGTCTTCATATTGCAGGAGTTAGGCT : 1848
BGV007931. : AAGACGATGAAAAGCGGAGGCTTCTTAAGATTATTTTCATTCTGTGTCTGTTGTTGGAAGACAGTTTATTGTGTAATGAAACAAATTAGTGGT-------------------------------------- : 1914
BGV007989. : AAGACGATGAAAAGCGGAGGCTTCTTAAGATTATTTTCATTCTGTGTCTGTTGTTGGAAGACAGTTTATTGTGTAATGAAACAAATTAGTGGT-------------------------------------- : 1914
M82.3      : AAGACGATGAAAAGCGGAGGCTTCTTAAGATTATTTTCATTCTGTGTCTGTTGTTGGAAGACAGTTTATTGTGTAATGAAACAAATTAGTGGT-------------------------------------- : 1914
EA00371.3  : AAGACGATGAAAAGCGGAGGCTTCTTAAGATTATTTTCATTCTGTGTCTGTTGTTGGAAGACAGTTTATTGTGTAATGAAACAAATTAGTGGT-------------------------------------- : 1914
Fla.8924.3 : AAGACGATGAAAAGCGGAGGCTTCTTAAGATTATTTTCATTCTGTGTCTGTTGTTGGAAGACAGTTTATTGTGTAATGAAACAAATTAGTGGT-------------------------------------- : 1914
Floradade. : AAGACGATGAAAAGCGGAGGCTTCTTAAGATTATTTTCATTCTGTGTCTGTTGTTGGAAGACAGTTTATTGTGTAATGAAACAAATTAGTGGT-------------------------------------- : 1914
LYC1410.3  : AAGACGATGAAAAGCGGAGGCTTCTTAAGATTATTTTCATTCTGTGTCTGTTGTTGGAAGACAGTTTATTGTGTAATGAAACAAATTAGTGGT-------------------------------------- : 1914
PI169588.3 : AAGACGATGAAAAGCGGAGGCTTCTTAAGATTATTTTCATTCTGTGTCTGTTGTTGGAAGACAGTTTATTGTGTAATGAAACAAATTAGTGGT-------------------------------------- : 1914
Brandywine : AAGACGATGAAAAGCGGAGGCTTCTTAAGATTATTTTCATTCTGTGTCTGTTGTTGGAAGACAGTTTATTGTGTAATGAAACAAATTAGTGGT-------------------------------------- : 1914
EA00990.3  : AAGACGATGAAAAGCGGAGGCTTCTTAAGATTATTTTCATTCTGTGTCTGTTGTTGGAAGACAGTTTATTGTGTAATGAAACAAATTAGTGGT-------------------------------------- : 1914
PI303721.3 : AAGACGATGAAAAGCGGAGGCTTCTTAAGATTATTTTCATTCTGTGTCTGTTGTTGGAAGACAGTTTATTGTGTAATGAAACAAATTAGTGGT-------------------------------------- : 1914
BGV006775. : AAGGCGATGAAAAGCGGAGGCTTCTTAAGATTATTTTCATTCTGTGTCTGTTGTTGGAAGACAGTTTATTGTGTAATGAAACAAATTAGTGGTCAGCCTTATCCCTGTCTTCATATTGCAGGAGTTAGGCT : 1848
PAS014479. : AAGGCGATGAAAAGCGGAGGCTTCTTAAGATTATTTTCATTCTGTGTCTGTTGTTGGAAGACAGTTTATTGTGTAATGAAACAAATTAGTGGTCAGCCTTATCCCTGTCTTCATATTGCAGGAGTTAGGCT : 1848
             AAGaCGATGAAAAGCGGAGGCTTCTTAAGATTATTTTCATTCTGTGTCTGTTGTTGGAAGACAGTTTATTGTGTAATGAAACAAATTAGTGGT
```

epm_Soly_M0_p1m00F_9780 2091

```
                  *      1980          *      2000          *      2020          *      2040          *      2060          *      2080          *
ITAG.3     : ------------------------------------------------------------------CAGAAATACTCTATTGAAAATTGATTATAATTATCCAGATTTATTGATGAAAAAGAAGAAAATAA : 1979
BGV006865. : CAAGCCTTACCTTGTATTCACTTCAATGGAACTGTAATCATGTAAACAAGATGTATATTAATTATGCAGAAATACTCTATTGAAAATTGATTATAATTATCCAGATTTATTGATGAAAAAGAAGAAAATAA : 1979
BGV007931. : ------------------------------------------------------------------CAGAAATACTCTATTGAAAATTGATTATAATTATCCAGATTTATTGATGAAAAAGAAGAAAATAA : 1979
BGV007989. : ------------------------------------------------------------------CAGAAATACTCTATTGAAAATTGATTATAATTATCCAGATTTATTGATGAAAAAGAAGAAAATAA : 1979
M82.3      : ------------------------------------------------------------------CAGAAATACTCTATTGAAAATTGATTATAATTATCCAGATTTATTGATGAAAAAGAAGAAAATAA : 1979
EA00371.3  : ------------------------------------------------------------------CAGAAATACTCTATTGAAAATTGATTATAATTATCCAGATTTATTGATGAAAAAGAAGAAAATAA : 1979
Fla.8924.3 : ------------------------------------------------------------------CAGAAATACTCTATTGAAAATTGATTATAATTATCCAGATTTATTGATGAAAAAGAAGAAAATAA : 1979
Floradade. : ------------------------------------------------------------------CAGAAATACTCTATTGAAAATTGATTATAATTATCCAGATTTATTGATGAAAAAGAAGAAAATAA : 1979
LYC1410.3  : ------------------------------------------------------------------CAGAAATACTCTATTGAAAATTGATTATAATTATCCAGATTTATTGATGAAAAAGAAGAAAATAA : 1979
PI169588.3 : ------------------------------------------------------------------CAGAAATACTCTATTGAAAATTGATTATAATTATCCAGATTTATTGATGAAAAAGAAGAAAATAA : 1979
Brandywine : ------------------------------------------------------------------CAGAAATACTCTATTGAAAATTGATTATAATTATCCAGATTTATTGATGAAAAAGAAGAAAATAA : 1979
EA00990.3  : ------------------------------------------------------------------CAGAAATACTCTATTGAAAATTGATTATAATTATCCAGATTTATTGATGAAAAAGAAGAAAATAA : 1979
PI303721.3 : ------------------------------------------------------------------CAGAAATACTCTATTGAAAATTGATTATAATTATCCAGATTTATTGATGAAAAAGAAGAAAATAA : 1979
BGV006775. : CAAGCCTTACCTTGTATTCACTTCAATGGAACTGTAATCATGTAAACAAGATGTATATTAATTATGCAGAAATACTCTATTGAAAATTGATTATAATTATCCAGATTTATTGATGAAAAAGAAGAAAATAA : 1979
PAS014479. : CAAGCCTTACCTTGTATTCACTTCAATGGAACTGTAATCATGTAAACAAGATGTATATTAATTATGCAGAAATACTCTATTGAAAATTGATTATAATTATCCAGATTTATTGATGAAAAAGAAGAAAATAA : 1979
                                                                               CAGAAATACTCTATTGAAAATTGATTATAATTATCCAGATTTATTGATGAAAAAGAAGAAAATAA
```

epm_Soly_M0_p1m03F_719 2152

epm_Soly_M0_p0m00F_5096 2161

epm_Soly_M0_p0m19F_137 2161

epm_Soly_M0_p1m00F_9780 2161

```
             2100          *      2120          *      2140          *      2160          *      2180          *      2200          *      2220
ITAG.3     : TACATGCATGGACCATTAAACTAGTAATATTATTTGTGTTGTAAAATTCAGAGCAAATGGCCTCCAACAACACAAAGCTTGAATAATGATAAAAGAGTATTTTAATTAATACTAAGGACAATTAAAATAAG : 2110
BGV006865. : TACATGCATGGACCATTAAACTAGTAATATTATTTGTGTTGTAAAATTCAGAGCAAATGGCCTCCAACAACACAAAGCTTGAATAATGATAAAAGAGTATTTTAATTAATACTAAGGACAATTAAAATAAG : 2110
BGV007931. : TACATGCATGGACCATTAAACTAGTAATATTATTTGTGTTGTAAAATTCAGAGCAAATGGCCTCCAACAACACAAAGCTTGAATAATGATAAAAGAGTATTTTAATTAATACTAAGGACAATTAAAATAAG : 2110
BGV007989. : TACATGCATGGACCATTAAACTAGTAATATTATTTGTGTTGTAAAATTCAGAGCAAATGGCCTCCAACAACACAAAGCTTGAATAATGATAAAAGAGTATTTTAATTAATACTAAGGACAATTAAAATAAG : 2110
M82.3      : TACATGCATGGACCATTAAACTAGTAATATTATTTGTGTTGTAAAATTCAGAGCAAATGGCCTCCAACAACACAAAGCTTGAATAATGATAAAAGAGTATTTTAATTAATACTAAGGACAATTAAAATAAG : 2110
EA00371.3  : TACATGCATGGACCATTAAACTAGTAATATTATTTGTGTTGTAAAATTCAGAGCAAATGGCCTCCAACAACACAAAGCTTGAATAATGATAAAAGAGTATTTTAATTAATACTAAGGACAATTAAAATAAG : 2110
Fla.8924.3 : TACATGCATGGACCATTAAACTAGTAATATTATTTGTGTTGTAAAATTCAGAGCAAATGGCCTCCAACAACACAAAGCTTGAATAATGATAAAAGAGTATTTTAATTAATACTAAGGACAATTAAAATAAG : 2110
Floradade. : TACATGCATGGACCATTAAACTAGTAATATTATTTGTGTTGTAAAATTCAGAGCAAATGGCCTCCAACAACACAAAGCTTGAATAATGATAAAAGAGTATTTTAATTAATACTAAGGACAATTAAAATAAG : 2110
LYC1410.3  : TACATGCATGGACCATTAAACTAGTAATATTATTTGTGTTGTAAAATTCAGAGCAAATGGCCTCCAACAACACAAAGCTTGAATAATGATAAAAGAGTATTTTAATTAATACTAAGGACAATTAAAATAAG : 2110
PI169588.3 : TACATGCATGGACCATTAAACTAGTAATATTATTTGTGTTGTAAAATTCAGAGCAAATGGCCTCCAACAACACAAAGCTTGAATAATGATAAAAGAGTATTTTAATTAATACTAAGGACAATTAAAATAAG : 2110
Brandywine : TACATGCATGGACCATTAAACTAGTAATATTATTTGTGTTGTAAAATTCAGAGCAAATGGCCTCCAACAACACAAAGCTTGAATAATGATAAAAGAGTATTTTAATTAATACTAAGGACAATTAAAATAAG : 2110
EA00990.3  : TACATGCATGGACCATTAAACTAGTAATATTATTTGTGTTGTAAAATTCAGAGCAAATGGCCTCCAACAACACAAAGCTTGAATAATGATAAAAGAGTATTTTAATTAATACTAAGGACAATTAAAATAAG : 2110
PI303721.3 : TACATGCATGGACCATTAAACTAGTAATATTATTTGTGTTGTAAAATTCAGAGCAAATGGCCTCCAACAACACAAAGCTTGAATAATGATAAAAGAGTATTTTAATTAATACTAAGGACAATTAAAATAAG : 2110
BGV006775. : TACATGCATGGACCATTAAACTAGTAATATTATTTGTGTTGTAAAATTCAGAGCAAATGGCCTCCAACAACACAAAGCTTGAATAATGATAAAAGAGTATTTTAATTAATACTAAGGACAATTAAAATAAG : 2110
PAS014479. : TACATGCATGGACCATTAAACTAGTAATATTATTTGTGTTGTAAAATTCAGAGCAAATGGCCTCCAACAACACAAAGCTTGAATAATGATAAAAGAGTATTTTAATTAATACTAAGGACAATTAAAATAAG : 2110
             TACATGCATGGACCATTAAACTAGTAATATTATTTGTGTTGTAAAATTCAGAGCAAATGGCCTCCAACAACACAAAGCTTGAATAATGATAAAAGAGTATTTTAATTAATACTAAGGACAATTAAAATAAG
```

epm_Soly_M0_p0m05F_541 2274

epm_Soly_M0_p0m12R_252 2276

epm_Soly_M0_p1m08R_108 2276

epm_Soly_M0_p0m02R_1076 2287

epm_Soly_M0_p1m08F_108 2292

epm_Soly_M0_p0m06R_537 2295

epm_Soly_M0_p1m08F_108 2295

```
               *      2240          *      2260          *      2280          *      2300          *      2320          *      2340          *      23
ITAG.3     : ACGAAGAAAAAAATTAGATCAATAATTTCTCTACAGGACAAGTAAAATGGATAGGAGGGAGCAGCTCCCCCACTTCTTCTACCTGTGTAATTGAAGCAAAAATAATATTTTTACACTATTCTAATTACGT : 2241
BGV006865. : ACGAAGAAAAAAATTAGATCAATAATTTCTCTACAGGACAAGTAAAATGGATAGGAGGGAGCAGCTCCCCCACTTCTTCTACCTGTGTAATTGAAGCAAAAATAATATTTTTACACTATTCTAATTACGT : 2241
BGV007931. : ACGAAGAAAAAAATTAGATCAATAATTTCTCTACAGGACAAGTAAAATGGATAGGAGGGAGCAGCTCCCCCACTTCTTCTACCTGTGTAATTGAAGCAAAAATAATATTTTTACACTATTCTAATTACGT : 2241
BGV007989. : ACGAAGAAAAAAATTAGATCAATAATTTCTCTACAGGACAAGTAAAATGGATAGGAGGGAGCAGCTCCCCCACTTCTTCTACCTGTGTAATTGAAGCAAAAATAATATTTTTACACTATTCTAATTACGT : 2241
M82.3      : ACGAAGAAAAAAATTAGATCAATAATTTCTCTACAGGACAAGTAAAATGGATAGGAGGGAGCAGCTCCCCCACTTCTTCTACCTGTGTAATTGAAGCAAAAATAATATTTTTACACTATTCTAATTACGT : 2241
EA00371.3  : ACGAAGAAAAAAATTAGATCAATAATTTCTCTACAGGACAAGTAAAATGGATAGGAGGGAGCAGCTCCCCCACTTCTTCTACCTGTGTAATTGAAGCAAAAATAATATTTTTACACTATTCTAATTACGT : 2241
Fla.8924.3 : ACGAAGAAAAAAATTAGATCAATAATTTCTCTACAGGACAAGTAAAATGGATAGGAGGGAGCAGCTCCCCCACTTCTTCTACCTGTGTAATTGAAGCAAAAATAATATTTTTACACTATTCTAATTACGT : 2241
Floradade. : ACGAAGAAAAAAATTAGATCAATAATTTCTCTACAGGACAAGTAAAATGGATAGGAGGGAGCAGCTCCCCCACTTCTTCTACCTGTGTAATTGAAGCAAAAATAATATTTTTACACTATTCTAATTACGT : 2241
LYC1410.3  : ACGAAGAAAAAAATTAGATCAATAATTTCTCTACAGGACAAGTAAAATGGATAGGAGGGAGCAGCTCCCCCACTTCTTCTACCTGTGTAATTGAAGCAAAAATAATATTTTTACACTATTCTAATTACGT : 2241
PI169588.3 : ACGAAGAAAAAAATTAGATCAATAATTTCTCTACAGGACAAGTAAAATGGATAGGAGGGAGCAGCTCCCCCACTTCTTCTACCTGTGTAATTGAAGCAAAAATAATATTTTTACACTATTCTAATTACGT : 2241
Brandywine : ACGAAGAAAAAAATTAGATCAATAATTTCTCTACAGGACAAGTAAAATGGATAGGAGGGAGCAGCTCCCCCACTTCTTCTACCTGTG---TTGAAGCAAAAATAATATTTTTACACTATTCTAATTACGT : 2238
EA00990.3  : ACGAAGAAAAAAATTAGATCAATAATTTCTCTACAGGACAAGTAAAATGGATAGGAGGGAGCAGCTCCCCCACTTCTTCTACCTGTGTAATTGAAGCAAAAATAATATTTTTACACTATTCTAATTACGT : 2241
PI303721.3 : ACGAAGAAAAAAATTAGATCAATAATTTCTCTACAGGACAAGTAAAATGGATAGGAGGGAGCAGCTCCCCCACTTCTTCTACCTGTG---TTGAAGCAAAAATAATATTTTTACACTATTCTAATTACGT : 2238
BGV006775. : ACGAAGAAAAAAATTAGATCAATAATTTCTCTACAGGACAAGTAAAATGGATAGGAGGGAGCAGCTCCCCCACTTCTTCTACCTGTGTAATTGAAGCAAAAATAATATTTTTACACTATTCTAATTACGT : 2241
PAS014479. : ACGAAGAAAAAAATTAGATCAATAATTTCTCTACAGGACAAGTAAAATGGATAGGAGGGAGCAGCTCCCCCACTTCTTCTACCTGTGTAATTGAAGCAAAAATAATATTTTTACACTATTCTAATTACGT : 2241
             ACGAAGAAAAAAATTAGATCAATAATTTCTCTACAGGACAAGTAAAATGGATAGGAGGGAGCAGCTCCCCCACTTCTTCTACCTGTGtaaTTGAAGCAAAAATAATATTTTTACACTATTCTAATTACGT
```

epm_Soly_M0_p0m14F_211 2473

```
              60         *      2380         *      2400         *      2420         *      2440         *      2460         *      2480
ITAG.3      : ATAGTATTATATTAAGTTATTTTAATGAATATAATCTTTAAATATATAGTACTATTTTCAATTGTTATATAAAATTATGCATCAACAAATTGAATGATTACTATATAAGTCTATAACAAAAACAAAATACG : 2372
BGV006865.  : ATAGTATTATATTGAGTTATTTTAATGAATATAATCTTTAAATATATAGTACTATTTTCAATTGTTATATAAAATTATGCATCAACAAATTGAATGATTAC--TATAAGTCTATAACAAAAACAAAATACG : 2370
BGV007931.  : ATAGTATTATATTAAGTTATTTTAATGAATATAATCTTTAAATATATAGTACTATTTTCAATTGTTATATAAAATTATGCATCAACAAATTGAATGATTACTATATAAGTCTATAACAAAAACAAAATACG : 2372
BGV007989.  : ATAGTATTATATTAAGTTATTTTAATGAATATAATCTTTAAATATATAGTACTATTTTCAATTGTTATATAAAATTATGCATCAACAAATTGAATGATTACTATATAAGTCTATAACAAAAACAAAATACG : 2372
M82.3       : ATAGTATTATATTAAGTTATTTTAATGAATATAATCTTTAAATATATAGTACTATTTTCAATTGTTATATAAAATTATGCATCAACAAATTGAATGATTACTATATAAGTCTATAACAAAAACAAAATACG : 2372
EA00371.3   : ATAGTATTATATTAAGTTATTTTAATGAATATAATCTTTAAATATATAGTACTATTTTCAATTGTTATATAAAATTATGCATCAACAAATTGAATGATTACTATATAAGTCTATAACAAAAACAAAATACG : 2372
Fla.8924.3  : ATAGTATTATATTAAGTTATTTTAATGAATATAATCTTTAAATATATAGTACTATTTTCAATTGTTATATAAAATTATGCATCAACAAATTGAATGATTACTATATAAGTCTATAACAAAAACAAAATACG : 2372
Floradade.  : ATAGTATTATATTAAGTTATTTTAATGAATATAATCTTTAAATATATAGTACTATTTTCAATTGTTATATAAAATTATGCATCAACAAATTGAATGATTACTATATAAGTCTATAACAAAAACAAAATACG : 2372
LYC1410.3   : ATAGTATTATATTAAGTTATTTTAATGAATATAATCTTTAAATATATAGTACTATTTTCAATTGTTATATAAAATTATGCATCAACAAATTGAATGATTACTATATAAGTCTATAACAAAAACAAAATACG : 2372
PI169588.3  : ATAGTATTATATTAAGTTATTTTAATGAATATAATCTTTAAATATATAGTACTATTTTCAATTGTTATATAAAATTATGCATCAACAAATTGAATGATTACTATATAAGTCTATAACAAAAACAAAATACG : 2372
Brandywine  : ATAGTATTATATTAAGTTATTTTAATGAATATAATCTTTAAATATATAGTACTATTTTCAATTGTTATATAAAATTATGCATCAACAAATTGAATGATTACTATATAAGTCTATAACAAAAACAAAATACG : 2369
EA00990.3   : ATAGTATTATATTAAGTTATTTTAATGAATATAATCTTTAAATATATAGTACTATTTTCAATTGTTATATAAAATTATGCATCAACAAATTGAATGATTACTATATAAGTCTATAACAAAAACAAAATACG : 2372
PI303721.3  : ATAGTATTATATTAAGTTATTTTAATGAATATAATCTTTAAATATATAGTACTATTTTCAATTGTTATATAAAATTATGCATCAACAAATTGAATGATTACTATATAAGTCTATAACAAAAACAAAATACG : 2369
BGV006775.  : ATAGTATTATATTGAGTTATTTTAATGAATATAATCTTTAAATATATAGTACTATTTTCAATTGTTATATAAAATTATGCATCAACAAATTGAATGATTAC--TATAAGTCTATAACAAAAACAAAATACG : 2370
PAS014479.  : ATAGTATTATATTAAGTTATTTTAATGAATATAATCTTTAAATATATAGTACTACTTTCAATTGTTATATAAAATTATGCATCAACAAATTGAATGATTAC--TATAAGTCTATAACAAAAACAAAATACG : 2370
              ATAGTATTATATTaAGTTATTTTAATGAATATAATCTTTAAATATATAGTACTAtTTTCAATTGTTATATAAAATTATGCATCAACAAATTGAATGATTACtaTATAAGTCTATAACAAAAACAAAATACG

              *      2500         *      2520         *      2540         *      2560         *      2580         *      2600         *      2620
ITAG.3      : GGATAAAACTAATATAAACATGTAGAGTCCAAAATAAAATAAAAATAATTTAATAAAAAGTACAAACGAGTTAAGTGAGAAATAAATATTAAGATAAAAGACATAATTATTTTAGGTACCCGATAGGACAA : 2503
BGV006865.  : GGATAAAACTAATATAAACATGTAGAGTCCAAAATAAAATAAAAATAATTTAATAAAAAGTACAAACGAGTTAAGTGAGAAATAAATATTAAGATAAAAGACATAATTATTTTAGGTACCCGATAGGACAA : 2501
BGV007931.  : GGATAAAACTAATATAAACATGTAGAGTCCAAAATAAAATAAAAATAATTTAATAAAAAGTACAAACGAGTTAAGTGAGAAATAAATATTAAGATAAAAGACATAATTATTTTAGGTACCCGATAGGACAA : 2503
BGV007989.  : GGATAAAACTAATATAAACATGTAGAGTCCAAAATAAAATAAAAATAATTTAATAAAAAGTACAAACGAGTTAAGTGAGAAATAAATATTAAGATAAAAGACATAATTATTTTAGGTACCCGATAGGACAA : 2503
M82.3       : GGATAAAACTAATATAAACATGTAGAGTCCAAAATAAAATAAAAATAATTTAATAAAAAGTACAAACGAGTTAAGTGAGAAATAAATATTAAGATAAAAGACATAATTATTTTAGGTACCCGATAGGACAA : 2503
EA00371.3   : GGATAAAACTAATATAAACATGTAGAGTCCAAAATAAAATAAAAATAATTTAATAAAAAGTACAAACGAGTTAAGTGAGAAATAAATATTAAGATAAAAGACATAATTATTTTAGGTACCCGATAGGACAA : 2503
Fla.8924.3  : GGATAAAACTAATATAAACATGTAGAGTCCAAAATAAAATAAAAATAATTTAATAAAAAGTACAAACGAGTTAAGTGAGAAATAAATATTAAGATAAAAGACATAATTATTTTAGGTACCCGATAGGACAA : 2503
Floradade.  : GGATAAAACTAATATAAACATGTAGAGTCCAAAATAAAATAAAAATAATTTAATAAAAAGTACAAACGAGTTAAGTGAGAAATAAATATTAAGATAAAAGACATAATTATTTTAGGTACCCGATAGGACAA : 2503
LYC1410.3   : GGATAAAACTAATATAAACATGTAGAGTCCAAAATAAAATAAAAATAATTTAATAAAAAGTACAAACGAGTTAAGTGAGAAATAAATATTAAGATAAAAGACATAATTATTTTAGGTACCCGATAGGACAA : 2503
PI169588.3  : GGATAAAACTAATATAAACATGTAGAGTCCAAAATAAAATAAAAATAATTTAATAAAAAGTACAAACGAGTTAAGTGAGAAATAAATATTAAGATAAAAGACATAATTATTTTAGGTACCCGATAGGACAA : 2503
Brandywine  : GGATAAAACTAATATAAACATGTAGAGTCCAAAATAAAATAAAAATAATTTAATAAAAAGTACAAACGAGTTAAGTGAGAAATAAATATTAAGATAAAAGACATAATTATTTTAGGTACCCGATAGGACAA : 2500
EA00990.3   : GGATAAAACTAATATAAACATGTAGAGTCCAAAATAAAATAAAAATAATTTAATAAAAAGTACAAACGAGTTAAGTGAGAAATAAATATTAAGATAAAAGACATAATTATTTTAGGTACCCGATAGGACAA : 2503
PI303721.3  : GGATAAAACTAATATAAACATGTAGAGTCCAAAATAAAATAAAAATAATTTAATAAAAAGTACAAACGAGTTAAGTGAGAAATAAATATTAAGATAAAAGACATAATTATTTTAGGTACCCGATAGGACAA : 2500
BGV006775.  : GGATAAAACTAATATAAACATGTAGAGTCCAAAATAAAATAAAAATAATTTAATAAAAAGTACAAACGAGTTAAGTGAGAAATAAATATTAAGATAAAAGACATAATTATTTTAGGTACCCGATAGGACAA : 2501
PAS014479.  : GGATAAAACTAATATAAACATGTAGAGTCCAAAATAAAATAAAAATAATTTAATAAAAAGTACAAACGAGTTAAGTGAGAAATAAATATTAAGATAAAAGACATAATTATTTTAGGTACCCGATAGGACAA : 2501
              GGATAAAACTAATATAAACATGTAGAGTCCAAAATAAAATAAAAATAATTTAATAAAAAGTACAAACGAGTTAAGTGAGAAATAAATATTAAGATAAAAGACATAATTATTTTAGGTACCCGATAGGACAA

                      *      2640         *      2660         *      2680         *      2700         *      2720         *      2740         *
ITAG.3      : TAGACCGGCACACATCACACATATTATATAGTACATCTTTTTCTTTTTTCTTTCGAATCAATAAAAAACGTGAAGAGGTTGCAATTATTTGTGCCTTTTTCTCAGGAGCTTGAAAGAGAAATATCAATCAC : 2634
BGV006865.  : TAGACCGGCACACATCACACATATTATATAGTACATCTTTTTCTTTTTTCTTTCGAATCAATAAAAAACGTGAAGAGGTTGCAATTATTTGTGCCTTTTTCTCAGGAGCTTGAAAGAGAAATATCAATCAC : 2632
BGV007931.  : TAGACCGGCACACATCACACATATTATATAGTACATCTTTTTCTTTTTTCTTTCGAATCAATAAAAAACGTGAAGAGGTTGCAATTATTTGTGCCTTTTTCTCAGGAGCTTGAAAGAGAAATATCAATCAC : 2634
BGV007989.  : TAGACCGGCACACATCACACATATTATATAGTACATCTTTTTCTTTTTTCTTTCGAATCAATAAAAAACGTGAAGAGGTTGCAATTATTTGTGCCTTTTTCTCAGGAGCTTGAAAGAGAAATATCAATCAC : 2634
M82.3       : TAGACCGGCACACATCACACATATTATATAGTACATCTTTTTCTTTTTTCTTTCGAATCAATAAAAAACGTGAAGAGGTTGCAATTATTTGTGCCTTTTTCTCAGGAGCTTGAAAGAGAAATATCAATCAC : 2634
EA00371.3   : TAGACCGGCACACATCACACATATTATATAGTACATCTTTTTCTTTTTTCTTTCGAATCAATAAAAAACGTGAAGAGGTTGCAATTATTTGTGCCTTTTTCTCAGGAGCTTGAAAGAGAAATATCAATCAC : 2634
Fla.8924.3  : TAGACCGGCACACATCACACATATTATATAGTACATCTTTTTCTTTTTTCTTTCGAATCAATAAAAAACGTGAAGAGGTTGCAATTATTTGTGCCTTTTTCTCAGGAGCTTGAAAGAGAAATATCAATCAC : 2634
Floradade.  : TAGACCGGCACACATCACACATATTATATAGTACATCTTTTTCTTTTTTCTTTCGAATCAATAAAAAACGTGAAGAGGTTGCAATTATTTGTGCCTTTTTCTCAGGAGCTTGAAAGAGAAATATCAATCAC : 2634
LYC1410.3   : TAGACCGGCACACATCACACATATTATATAGTACATCTTTTTCTTTTTTCTTTCGAATCAATAAAAAACGTGAAGAGGTTGCAATTATTTGTGCCTTTTTCTCAGGAGCTTGAAAGAGAAATATCAATCAC : 2634
PI169588.3  : TAGACCGGCACACATCACACATATTATATAGTACATCTTTTTCTTTTTTCTTTCGAATCAATAAAAAACGTGAAGAGGTTGCAATTATTTGTGCCTTTTTCTCAGGAGCTTGAAAGAGAAATATCAATCAC : 2634
Brandywine  : TAGACCGGCACACATCACACATATTATATAGTACATCTTTTTCTTTTTTCTTTCGAATCAATAAAAAACGTGAAGAGGTTGCAATTATTTGTGCCTTTTTCTCAGGAGCTTGAAAGAGAAATATCAATCAC : 2631
EA00990.3   : TAGACCGGCACACATCACACATATTATATAGTACATCTTTTTCTTTTTTCTTTCGAATCAATAAAAAACGTGAAGAGGTTGCAATTATTTGTGCCTTTTTCTCAGGAGCTTGAAAGAGAAATATCAATCAC : 2634
PI303721.3  : TAGACCGGCACACATCACACATATTATATAGTACATCTTTTTCTTTTTTCTTTCGAATCAATAAAAAACGTGAAGAGGTTGCAATTATTTGTGCCTTTTTCTCAGGAGCTTGAAAGAGAAATATCAATCAC : 2631
BGV006775.  : TAGACCGGCACACATCACACATATTATATAGTACATCTTTTTCTTTTTTCTTTCGAATCAATAAAAAACGTGAAGAGGTTGCAATTATTTGTGCCTTTTTCTCAGGAGCTTGAAAGAGAAATATCAATCAC : 2632
PAS014479.  : TAGACCGGCACACATCACACATATTATATAGTACATCTTTTTCTTTTTTCTTTCGAATCAATAAAAAACGTGAAGAGGTTGCAATTATTTGTGCCTTTTTCTCAGGAGCTTGAAAGAGAAATATCAATCAC : 2632
              TAGACCGGCACACATCACACATATTATATAGTACATCTTTTTCTTTTTTCTTTCGAATCAATAAAAAACGTGAAGAGGTTGCAATTATTTGTGCCTTTTTCTCAGGAGCTTGAAAGAGAAATATCAATCAC

                  2760         *      2780         *      2800         *      2820         *      2840         *      2860         *      2880
ITAG.3      : CACTTCTCTCTATTTTTCCTCATTCACTTAACAAATCAAAGCTGTATTATGCTTCCTGCAATGTTTTATCAAATAGCAATTTGCATGTTGTTTTGTGGTTTCATAGTATCATCATATCATCAAAAAGATAT : 2765
BGV006865.  : CACTTCTCTCTCTTTTTCCTCATTCACTTAACAAATCAAAGCTGTATTATGCTTCCTGCAATGTTTTATCAAATAGCAATTTGCATGTTGTTTTGTGGTTTCATAGTATCATCATATCATCAAAAAGATAT : 2763
BGV007931.  : CACTTCTCTCTATTTTTCCTCATTCACTTAACAAATCAAAGCTGTATTATGCTTCCTGCAATGTTTTATCAAATAGCAATTTGCATGTTGTTTTGTGGTTTCATAGTATCATCATATCATCAAAAAGATAT : 2765
BGV007989.  : CACTTCTCTCTATTTTTCCTCATTCACTTAACAAATCAAAGCTGTATTATGCTTCCTGCAATGTTTTATCAAATAGCAATTTGCATGTTGTTTTGTGGTTTCATAGTATCATCATATCATCAAAAAGATAT : 2765
M82.3       : CACTTCTCTCTATTTTTCCTCATTCACTTAACAAATCAAAGCTGTATTATGCTTCCTGCAATGTTTTATCAAATAGCAATTTGCATGTTGTTTTGTGGTTTCATAGTATCATCATATCATCAAAAAGATAT : 2765
EA00371.3   : CACTTCTCTCTATTTTTCCTCATTCACTTAACAAATCAAAGCTGTATTATGCTTCCTGCAATGTTTTATCAAATAGCAATTTGCATGTTGTTTTGTGGTTTCATAGTATCATCATATCATCAAAAAGATAT : 2765
Fla.8924.3  : CACTTCTCTCTATTTTTCCTCATTCACTTAACAAATCAAAGCTGTATTATGCTTCCTGCAATGTTTTATCAAATAGCAATTTGCATGTTGTTTTGTGGTTTCATAGTATCATCATATCATCAAAAAGATAT : 2765
Floradade.  : CACTTCTCTCTATTTTTCCTCATTCACTTAACAAATCAAAGCTGTATTATGCTTCCTGCAATGTTTTATCAAATAGCAATTTGCATGTTGTTTTGTGGTTTCATAGTATCATCATATCATCAAAAAGATAT : 2765
LYC1410.3   : CACTTCTCTCTATTTTTCCTCATTCACTTAACAAATCAAAGCTGTATTATGCTTCCTGCAATGTTTTATCAAATAGCAATTTGCATGTTGTTTTGTGGTTTCATAGTATCATCATATCATCAAAAAGATAT : 2765
PI169588.3  : CACTTCTCTCTATTTTTCCTCATTCACTTAACAAATCAAAGCTGTATTATGCTTCCTGCAATGTTTTATCAAATAGCAATTTGCATGTTGTTTTGTGGTTTCATAGTATCATCATATCATCAAAAAGATAT : 2765
Brandywine  : CACTTCTCTCTATTTTTCCTCATTCACTTAACAAATCAAAGCTGTATTATGCTTCCTGCAATGTTTTATCAAATAGCAATTTGCATGTTGTTTTGTGGTTTCATAGTATCATCATATCATCAAAAAGATAT : 2762
EA00990.3   : CACTTCTCTCTATTTTTCCTCATTCACTTAACAAATCAAAGCTGTATTATGCTTCCTGCAATGTTTTATCAAATAGCAATTTGCATGTTGTTTTGTGGTTTCATAGTATCATCATATCATCAAAAAGATAT : 2765
PI303721.3  : CACTTCTCTCTATTTTTCCTCATTCACTTAACAAATCAAAGCTGTATTATGCTTCCTGCAATGTTTTATCAAATAGCAATTTGCATGTTGTTTTGTGGTTTCATAGTATCATCATATCATCAAAAAGATAT : 2762
BGV006775.  : CACTTCTCTCTATTTTTCCTCATTCACTTAACAAATCAAAGCTGTATTATGCTTCCTGCAATGTTTTATCAAATAGCAATTTGCATGTTGTTTTGTGGTTTCATAGTATCATCATATCATCAAAAAGATAT : 2763
PAS014479.  : CACTTCTCTCTATTTTTCCTCATTCACTTAACAAATCAAAGCTGTATTATGCTTCCTGCAATGTTTTATCAAATAGCAATTTGCATGTTGTTTTGTGGTTTCATAGTATCATCATATCATCAAAAAGATAT : 2763
              CACTTCTCTCTaTTTTTCCTCATTCACTTAACAAATCAAAGCTGTATTATGCTTCCTGCAATGTTTTATCAAATAGCAATTTGCATGTTGTTTTGTGGTTTCATAGTATCATCATATCATCAAAAAGATAT

                    *      2900         *      2920         *      2940         *      2960         *      2980         *      3000         *
ITAG.3      : CCTTAATATTCTCAAAGAAAATCCTAATCAAGTTTCATCTTCAAACAATTTCTTGTTTGGGACAGCCTCTTCTTGTTACCAGGTACTTCCTACACTTG-TTTTTTTTTTTTTTGTTTCGAGTATAACTTTA : 2895
BGV006865.  : CCTTAATATTCTCAAAGAAAATCCTAATCAAGTTTCATCTTCAAACAATTTCTTGTTTGGGACAGCCTCTTCTTGTTACCAGGTACTTCCTACACTTG--TTTTTTTTTTTTTGTTTTGAGTATAACTTTA : 2892
BGV007931.  : CCTTAATATTCTCAAAGAAAATCCTAATCAAGTTTCATCTTCAAACAATTTCTTGTTTGGGACAGCCTCTTCTTGTTACCAGGTACTTCCTACACTTGTTTTTTTTTTTTTTTGTTTCGAGTATAACTTTA : 2896
BGV007989.  : CCTTAATATTCTCAAAGAAAATCCTAATCAAGTTTCATCTTCAAACAATTTCTTGTTTGGGACAGCCTCTTCTTGTTACCAGGTACTTCCTACACTTG-TTTTTTTTTTTTTTGTTTCGAGTATAACTTTA : 2895
M82.3       : CCTTAATATTCTCAAAGAAAATCCTAATCAAGTTTCATCTTCAAACAATTTCTTGTTTGGGACAGCCTCTTCTTGTTACCAGGTACTTCCTACACTTG-TTTTTTTTTTTTTTGTTTCGAGTATAACTTTA : 2895
EA00371.3   : CCTTAATATTCTCAAAGAAAATCCTAATCAAGTTTCATCTTCAAACAATTTCTTGTTTGGGACAGCCTCTTCTTGTTACCAGGTACTTCCTACACTTG-TTTTTTTTTTTTTTGTTTCGAGTATAACTTTA : 2895
Fla.8924.3  : CCTTAATATTCTCAAAGAAAATCCTAATCAAGTTTCATCTTCAAACAATTTCTTGTTTGGGACAGCCTCTTCTTGTTACCAGGTACTTCCTACACTTG-TTTTTTTTTTTTTTGTTTCGAGTATAACTTTA : 2895
Floradade.  : CCTTAATATTCTCAAAGAAAATCCTAATCAAGTTTCATCTTCAAACAATTTCTTGTTTGGGACAGCCTCTTCTTGTTACCAGGTACTTCCTACACTTG-TTTTTTTTTTTTTTGTTTCGAGTATAACTTTA : 2895
LYC1410.3   : CCTTAATATTCTCAAAGAAAATCCTAATCAAGTTTCATCTTCAAACAATTTCTTGTTTGGGACAGCCTCTTCTTGTTACCAGGTACTTCCTACACTTG-TTTTTTTTTTTTTTGTTTCGAGTATAACTTTA : 2895
PI169588.3  : CCTTAATATTCTCAAAGAAAATCCTAATCAAGTTTCATCTTCAAACAATTTCTTGTTTGGGACAGCCTCTTCTTGTTACCAGGTACTTCCTACACTTG-TTTTTTTTTTTTTTGTTTCGAGTATAACTTTA : 2895
Brandywine  : CCTTAATATTCTCAAAGAAAATCCTAATCAAGTTTCATCTTCAAACAATTTCTTGTTTGGGACAGCCTCTTCTTGTTACCAGGTACTTCCTACACTTG-TTTTTTTTTTTTTTGTTTCGAGTATAACTTTA : 2892
EA00990.3   : CCTTAATATTCTCAAAGAAAATCCTAATCAAGTTTCATCTTCAAACAATTTCTTGTTTGGGACAGCCTCTTCTTGTTACCAGGTACTTCCTACACTTG-TTTTTTTTTTTTTTGTTTCGAGTATAACTTTA : 2895
PI303721.3  : CCTTAATATTCTCAAAGAAAATCCTAATCAAGTTTCATCTTCAAACAATTTCTTGTTTGGGACAGCCTCTTCTTGTTACCAGGTACTTCCTACACTTG-TTTTTTTTTTTTTTGTTTCGAGTATAACTTTA : 2892
BGV006775.  : CCTTAATATTCTCAAAGAAAATCCTAATCAAGTTTCATCTTCAAACAATTTCTTGTTTGGGACAGCCTCTTCTTGTTACCAGGTACTTCCTACACTTG--TTTTTTTTTTTTTGTTTTGAGTATAACTTTA : 2892
PAS014479.  : CCTTAATATTCTCAAAGAAAATCCTAATCAAGTTTCATCTTCAAACAATTTCTTGTTTGGGACAGCCTCTTCTTGTTACCAGGTACTTCCTACACTTG-TTTTTTTTTTTTTTGTTTCGAGTATAACTTTA : 2893
              CCTTAATATTCTCAAAGAAAATCCTAATCAAGTTTCATCTTCAAACAATTTCTTGTTTGGGACAGCCTCTTCTTGTTACCAGGTACTTCCTACACTTG tTTTTTTTTTTTTTGTTTcGAGTATAACTTTA

                 3020         *      3040         *      3060         *      3080         *      3100         *      3120         *      3140
ITAG.3      : ATTTTTCTTGTTTTCTCCGTAGTTTGAAGGAGCATTTCTCAGTGATGGAAAAAGTCTCAACAATTGGGACATTTTTACTCACGAAGCTGGTGAGCCCCCTTTTTTAAGGCATAATACATTAATTA---- : 3020
BGV006865.  : ATTTTTCTTGTTTTCTCCGTAGTTTGAAGGAGCATTTCTCAGTGATGGAAAAAGTCTCAACAATTGGGACATTTTTACTCACGAAGCTGGTGAG-CCCCTTTTTTAAGGCATAATACATTAATTATGAC : 3020
BGV007931.  : ATTTTTCTTGTTTTCTCCGTAGTTTGAAGGAGCATTTCTCAGTGATGGAAAAAGTCTCAACAATTGGGACATTTTTACTCACGAAGCTGGTGAGCCCCCTTTTTTAAGGCATAATACATTAATT----- : 3020
BGV007989.  : ATTTTTCTTGTTTTCTCCGTAGTTTGAAGGAGCATTTCTCAGTGATGGAAAAAGTCTCAACAATTGGGACATTTTTACTCACGAAGCTGGTGAGCCCCCTTTTTTAAGGCATAATACATTAATTA---- : 3020
M82.3       : ATTTTTCTTGTTTTCTCCGTAGTTTGAAGGAGCATTTCTCAGTGATGGAAAAAGTCTCAACAATTGGGACATTTTTACTCACGAAGCTGGTGAGCCCCCTTTTTTAAGGCATAATACATTAATTA---- : 3020
EA00371.3   : ATTTTTCTTGTTTTCTCCGTAGTTTGAAGGAGCATTTCTCAGTGATGGAAAAAGTCTCAACAATTGGGACATTTTTACTCACGAAGCTGGTGAGCCCCCTTTTTTAAGGCATAATACATTAATTA---- : 3020
Fla.8924.3  : ATTTTTCTTGTTTTCTCCGTAGTTTGAAGGAGCATTTCTCAGTGATGGAAAAAGTCTCAACAATTGGGACATTTTTACTCACGAAGCTGGTGAGCCCCCTTTTTTAAGGCATAATACATTAATTA---- : 3020
Floradade.  : ATTTTTCTTGTTTTCTCCGTAGTTTGAAGGAGCATTTCTCAGTGATGGAAAAAGTCTCAACAATTGGGACATTTTTACTCACGAAGCTGGTGAGCCCCCTTTTTTAAGGCATAATACATTAATTA---- : 3020
LYC1410.3   : ATTTTTCTTGTTTTCTCCGTAGTTTGAAGGAGCATTTCTCAGTGATGGAAAAAGTCTCAACAATTGGGACATTTTTACTCACGAAGCTGGTGAGCCCCCTTTTTTAAGGCATAATACATTAATTA---- : 3020
PI169588.3  : ATTTTTCTTGTTTTCTCCGTAGTTTGAAGGAGCATTTCTCAGTGATGGAAAAAGTCTCAACAATTGGGACATTTTTACTCACGAAGCTGGTGAGCCCCCTTTTTTAAGGCATAATACATTAATTA---- : 3020
Brandywine  : ATTTTTCTTGTTTTCTCCGTAGTTTGAAGGAGCATTTCTCAGTGATGGAAAAAGTCTCAACAATTGGGACATTTTTACTCACGAAGCTGGTGAGCCCCCTTTTTTAAGGCATAATACATTAATTATGA- : 3020
EA00990.3   : ATTTTTCTTGTTTTCTCCGTAGTTTGAAGGAGCATTTCTCAGTGATGGAAAAAGTCTCAACAATTGGGACATTTTTACTCACGAAGCTGGTGAGCCCCCTTTTTTAAGGCATAATACATTAATTA---- : 3020
PI303721.3  : ATTTTTCTTGTTTTCTCCGTAGTTTGAAGGAGCATTTCTCAGTGATGGAAAAAGTCTCAACAATTGGGACATTTTTACTCACGAAGCTGGTGAGCCCCCTTTTTTAAGGCATAATACATTAATTATGA- : 3020
BGV006775.  : ATTTTTCTTGTTTTCTCCGTAGTTTGAAGGAGCATTTCTCAGTGATGGAAAAAGTCTCAACAATTGGGACATTTTTACTCACGAAGCTGGTGAGCCCCCTTTTTTAAGGCATAATACATTAATTATGA- : 3020
PAS014479.  : ATTTTTCTTGTTTTCTCCGTAGTTTGAAGGAGCATTTCTCAGTGATGGAAAAAGTCTCAACAATTGGGACATTTTTACTCACGAAGCTGGTGAGCCCCCTTTTTTAAGGCATAATACATTAATTATG-- : 3020
              ATTTTTCTTGTTTTCTCCGTAGTTTGAAGGAGCATTTCTCAGTGATGGAAAAAGTCTCAACAATTGGGACATTTTTACTCACGAAGCTGGTGAGcCCCCTTTTTTAAGGCATAATACATTAATTa
```

Solyc09g064610

```
                      *        20         *        40         *        60         *        80         *       100         *       120         *
ITAG.3      : ------------------------------------------------------------------------------------------------------------------------------------ :    -
BGV006865.  : ------------------------------------------------------------------------------------------------------------------------------------ :    -
BGV007931.  : ------------------------------------------------------------------------------------------------------------------------------------ :    -
BGV007989.  : ------------------------------------------------------------------------------------------------------------------------------------ :    -
M82.3       : ------------------------------------------------------------------------------------------------------------------------------------ :    -
EA00371.3   : ------------------------------------------------------------------------------------------------------------------------------------ :    -
Fla.8924.3  : ------------------------------------------------------------------------------------------------------------------------------------ :    -
Floradade.  : ------------------------------------------------------------------------------------------------------------------------------------ :    -
LYC1410.3   : ------------------------------------------------------------------------------------------------------------------------------------ :    -
PI169588.3  : ------------------------------------------------------------------------------------------------------------------------------------ :    -
```

```
Brandywine  : ----------------------------------------------------------------------------------------------------------------------------------- :    -
EA00990.3   : ----------------------------------------------------------------------------------------------------------------------------------- :    -
PI303721.3  : ----------------------------------------------------------------------------------------------------------------------------------- :    -
BGV006775.  : ttatttaatattaaattaattatatattactccctctgtttcaaaaagaatgtccatatttccttttttagtctgtttcaaaaagaataacccatttcctttttggcaacactttacctttaattttccacg :  131
PAS014479.  : ttatttaatattaaattaattatatattactccctctgtttcaaaaagaatgtccatatttccttttttagtctgtttcaaaaagaataacccatttcctttttggcaacactttacctttaattttccacg :  131

                    140         *       160         *       180         *       200         *       220         *       240         *       260
ITAG.3      : ----------------------------------------------------------------------------------------------------------------------------------- :    -
BGV006865.  : ----------------------------------------------------------------------------------------------------------------------------------- :    -
BGV007931.  : ----------------------------------------------------------------------------------------------------------------------------------- :    -
BGV007989.  : ----------------------------------------------------------------------------------------------------------------------------------- :    -
M82.3       : ----------------------------------------------------------------------------------------------------------------------------------- :    -
EA00371.3   : ----------------------------------------------------------------------------------------------------------------------------------- :    -
Fla.8924.3  : ----------------------------------------------------------------------------------------------------------------------------------- :    -
Floradade.  : ----------------------------------------------------------------------------------------------------------------------------------- :    -
LYC1410.3   : ----------------------------------------------------------------------------------------------------------------------------------- :    -
PI169588.3  : ----------------------------------------------------------------------------------------------------------------------------------- :    -
Brandywine  : ----------------------------------------------------------------------------------------------------------------------------------- :    -
EA00990.3   : ----------------------------------------------------------------------------------------------------------------------------------- :    -
PI303721.3  : ----------------------------------------------------------------------------------------------------------------------------------- :    -
BGV006775.  : tgtcatgtttaagaccacataattaaaggacattttggtacatttgacagaattttaatttagaaccacaagattaaaaagtcttctttcttttattaaactccgttccaaatcaaactagatcatccttt :  262
PAS014479.  : tgtcatgtttaagaccacataattaaaggacattttggtacatttgacagaattttaatttagaaccacaagattaaaaagtcttctttcttttattaaactccgttccaaatcaaactagatcatccttt :  262

                     *       280         *       300         *       320         *       340         *       360         *       380         *
ITAG.3      : ----------------------------------------------------------------------------------------------------------------------------------- :    -
BGV006865.  : ----------------------------------------------------------------------------------------------------------------------------------- :    -
BGV007931.  : ----------------------------------------------------------------------------------------------------------------------------------- :    -
BGV007989.  : ----------------------------------------------------------------------------------------------------------------------------------- :    -
M82.3       : ----------------------------------------------------------------------------------------------------------------------------------- :    -
EA00371.3   : ----------------------------------------------------------------------------------------------------------------------------------- :    -
Fla.8924.3  : ----------------------------------------------------------------------------------------------------------------------------------- :    -
Floradade.  : ----------------------------------------------------------------------------------------------------------------------------------- :    -
LYC1410.3   : ----------------------------------------------------------------------------------------------------------------------------------- :    -
PI169588.3  : ----------------------------------------------------------------------------------------------------------------------------------- :    -
Brandywine  : ----------------------------------------------------------------------------------------------------------------------------------- :    -
EA00990.3   : ----------------------------------------------------------------------------------------------------------------------------------- :    -
PI303721.3  : ----------------------------------------------------------------------------------------------------------------------------------- :    -
BGV006775.  : tttaaactgagggagtaacatttaatttagttttaggaataaaataaggatagaatgataattcaatttttaaattagaagctttccacttataataatatatggaatgatcgagatatgatatatgatat :  393
PAS014479.  : tttaaactgagggagtaacatttaatttagttttaggaataaaataaggatagaatgataattcaatttttaaattagaagctttccacttataataatatatggaatgatcgagatatgatctatgatat :  393

                  400         *       420         *       440         *       460         *       480         *       500         *       520
ITAG.3      : --------------------------taattttacttcaaagatagcatcatacttaattttaaaataattcaaccttgattatttaattttatgcatgataaaataaattatgatcatattattattata :  105
BGV006865.  : --------------------------taattttacttcaaagatagcatcatacttaattttaaaataattcaaccttgattatttaattttatgcatgataaaataaattatgatcatattattattata :  105
BGV007931.  : --------------------------taattttacttcaaagatagcatcatacttaattttaaaataattcaaccttgattatttaattttatgcatgataaaataaattatgatcatattattattata :  105
BGV007989.  : --------------------------taattttacttcaaagatagcatcatacttaattttaaaataattcaaccttgattatttaattttatgcatgataaaataaattatgatcatattattattata :  105
M82.3       : --------------------------taattttacttcaaagatagcatcatacttaattttaaaataattcaaccttgattatttaattttatgcatgataaaataaattatgatcatattattattata :  105
EA00371.3   : --------------------------taattttacttcaaagatagcatcatacttaattttaaaataattcaaccttgattatttaattttatgcatgataaaataaattatgatcatattattattata :  105
Fla.8924.3  : ----------------------------attttacttcaaagatagcatcatacttaattttaaaataattcaaccttgattatttaattttatgcatgataaaataaattatgatcatattattattata :  103
Floradade.  : --------------------------taattttacttcaaagatagcatcatacttaattttaaaataattcaaccttgattatttaattttatgcatgataaaataaattatgatcatattattattata :  105
LYC1410.3   : --------------------------taattttacttcaaagatagcatcatacttaattttaaaataattcaaccttgattatttaattttatgcatgataaaataaattatgatcatattattattata :  105
PI169588.3  : --------------------------taattttacttcaaagatagcatcatacttaattttaaaataattcaaccttgattatttaattttatgcatgataaaataaattatgatcatattattattata :  105
Brandywine  : --------------------------taattttacttcaaagatagcatcatacttaattttaaaataattcaaccttgattatttaattttatgcatgataaaataaattatgatcatattattattata :  105
EA00990.3   : --------------------------taattttacttcaaagatagcatcatacttaattttaaaataattcaaccttgattatttaattttatgcatgataaaataaattatgatcatattattattata :  105
PI303721.3  : --------------------------taattttacttcaaagatagcatcatacttaattttaaaataattcaaccttgattatttaattttatgcatgataaaataaattatgatcatattattattata :  105
BGV006775.  : gatgtgatgattgatacttcatattttaattttacttcaaagatagcatcatacttaattttaaaataattcaaccttgcttatttaattttatgcatgataaaataaattatgatcatattattattata :  524
PAS014479.  : gatgtgatgattgatacttcatattttaattttacttcaaagatagcatcatacttaattttaaaataattcaaccttgattatttaattttatgcatgataaaataaattatgatcatattattattata :  524
                                        taATTTTACTTCAAAGATAGCATCATACTTAATTTTAAAATAATTCAACCTTGaTTATTTAATTTTATGCATGATAAAATAAATTATGATCATATTATTATTATA

                   *       540         *       560         *       580         *       600         *       620         *       640         *
ITAG.3      : atttagttagagaccacaaatttcagagtatatgtttataacaattagcaagctttctttttcaaaagcatacatgcccccaccaagttgacagactgataggaccagcaggaagctgccctttatgaaaa :  236
BGV006865.  : atttagttagagaccacaaatttcagagtatatgtttataacaattagcaagctttctttttcaaaagcatacatgcccccaccaagttgacagactgataggaccagcaggaagctgccctttatgaaaa :  236
BGV007931.  : atttagttagagaccacaaatttcagagtatatgtttataacaattagcaagctttctttttcaaaagcatacatgcccccaccaagttgacagactgataggaccagcaggaagctgccctttatgaaaa :  236
BGV007989.  : atttagttagagaccacaaatttcagagtatatgtttataacaattagcaagctttctttttcaaaagcatacatgcccccaccaagttgacagactgataggaccagcaggaagctgccctttatgaaaa :  236
M82.3       : atttagttagagaccacaaatttcagagtatatgtttataacaattagcaagctttctttttcaaaagcatacatgcccccaccaagttgacagactgataggaccagcaggaagctgccctttatgaaaa :  236
EA00371.3   : atttagttagagaccacaaatttcagagtatatgtttataacaattagcaagctttctttttcaaaagcatacatgcccccaccaagttgacagactgataggaccagcaggaagctgccctttatgaaaa :  236
Fla.8924.3  : atttagttagagaccacaaatttcagagtatatgtttataacaattagcaagctttctttttcaaaagcatacatgcccccaccaagttgacagactgataggaccagcaggaagctgccctttatgaaaa :  234
Floradade.  : atttagttagagaccacaaatttcagagtatatgtttataacaattagcaagctttctttttcaaaagcatacatgcccccaccaagttgacagactgataggaccagcaggaagctgccctttatgaaaa :  236
LYC1410.3   : atttagttagagaccacaaatttcagagtatatgtttataacaattagcaagctttctttttcaaaagcatacatgcccccaccaagttgacagactgataggaccagcaggaagctgccctttatgaaaa :  236
PI169588.3  : atttagttagagaccacaaatttcagagtatatgtttataacaattagcaagctttctttttcaaaagcatacatgcccccaccaagttgacagactgataggaccagcaggaagctgccctttatgaaaa :  236
Brandywine  : atttagttagagaccacaaatttcagagtatatgtttataacaattagcaagctttctttttcaaaagcatacatgcccccaccaagttgacagactgataggaccagcaggaagctgccctttatgaaaa :  236
EA00990.3   : atttagttagagaccacaaatttcagagtatatgtttataacaattagcaagctttctttttcaaaagcatacatgcccccaccaagttgacagactgataggaccagcaggaagctgccctttatgaaaa :  236
PI303721.3  : atttagttagagaccacaaatttcagagtatatgtttataacaattagcaagctttctttttcaaaagcatacatgcccccaccaagttgacagactgataggaccagcaggaagctgccctttatgaaaa :  236
BGV006775.  : atttagttggagaccacaaatttcagagtatatgtttataacaattagcaagctttctttttcaaaagcctacatgcccccaccaagttgacagactgataggaccagcaggaagctgccctttatgaaaa :  655
PAS014479.  : atttagttggagaccacaaatttcagagtatatgtttataacaattagcaagctttctttttcaaaagcctacatgcccccaccaagttgacagactgataggaccagcaggaagctgccctttatgaaaa :  655
              ATTTAGTTaGAGACCACAAATTTCAGAGTATATGTTTATAACAATTAGCAAGCTTTCTTTTTCAAAAGCaTACATGCCCCCACCAAGTTGACAGACTGATAGGACCAGCAGGAAGCTGCCCTTTATGAAAA

                660         *       680         *       700         *       720         *       740         *       760         *       780
ITAG.3      : gaaaaagtgttggcatctgcaattttcttgaaatgcatcccacatcattgttgcgataaatagtcattattcttaattaaaatctcgggcacaaattttgcgatacaaatatttttttgttaaaaagtgaa :  367
BGV006865.  : gaaaaagtgttggcatctgcaattttcttgaaatgcatcccacatcattgttgcgataaatagtcattattcttaattaaaatctcgggcacaaattttgcgatacaaatatttttttgttaaaaagtgaa :  367
BGV007931.  : gaaaaagtgttggcatctgcaattttcttgaaatgcatcccacatcattgttgcgataaatagtcattattcttaattaaaatctcgggcacaaattttgcgatacaaatatttttttgttaaaaagtgaa :  367
BGV007989.  : gaaaaagtgttggcatctgcaattttcttgaaatgcatcccacatcattgttgcgataaatagtcattattcttaattaaaatctcgggcacaaattttgcgatacaaatatttttttgttaaaaagtgaa :  367
M82.3       : gaaaaagtgttggcatctgcaattttcttgaaatgcatcccacatcattgttgcgataaatagtcattattcttaattaaaatctcgggcacaaattttgcgatacaaatatttttttgttaaaaagtgaa :  367
EA00371.3   : gaaaaagtgttggcatctgcaattttcttgaaatgcatcccacatcattgttgcgataaatagtcattattcttaattaaaatctcgggcacaaattttgcgatacaaatatttttttgttaaaaagtgaa :  367
Fla.8924.3  : gaaaaagtgttggcatctgcaattttcttgaaatgcatcccacatcattgttgcgataaatagtcattattcttaattaaaatctcgggcacaaattttgcgatacaaatatttttttgttaaaaagtgaa :  365
Floradade.  : gaaaaagtgttggcatctgcaattttcttgaaatgcatcccacatcattgttgcgataaatagtcattattcttaattaaaatctcgggcacaaattttgcgatacaaatatttttttgttaaaaagtgaa :  367
LYC1410.3   : gaaaaagtgttggcatctgcaattttcttgaaatgcatcccacatcattgttgcgataaatagtcattattcttaattaaaatctcgggcacaaattttgcgatacaaatatttttttgttaaaaagtgaa :  367
PI169588.3  : gaaaaagtgttggcatctgcaattttcttgaaatgcatcccacatcattgttgcgataaatagtcattattcttaattaaaatctcgggcacaaattttgcgatacaaatatttttttgttaaaaagtgaa :  367
Brandywine  : gaaaaagtgttggcatctgcaattttcttgaaatgcatcccacatcattgttgcgataaatagtcattattcttaattaaaatctcgggcacaaattttgcgatacaaatatttttttgttaaaaagtgaa :  367
EA00990.3   : gaaaaagtgttggcatctgcaattttcttgaaatgcatcccacatcattgttgcgataaatagtcattattcttaattaaaatctcgggcacaaattttgcgatacaaatatttttttgttaaaaagtgaa :  367
PI303721.3  : gaaaaagtgttggcatctgcaattttcttgaaatgcatcccacatcattgttgcgataaatagtcattattcttaattaaaatctcgggcacaaattttgcgatacaaatatttttttgttaaaaagtgaa :  367
BGV006775.  : gaaaaagtgttggcatctgcaattttcttgaaatgcatcccacatcattgttgcgataaatagtcattattcttaattaaaatctcgggcacaaattttgcgatacaaatatttttttgttaaaaagtgaa :  786
PAS014479.  : gaaaaagtgttggcatctgcaattttcttgaaatgcatcccacatcattgttgcgataaatagtcattattcttaattaaaatctcgggcacaaattttgcgatacaaatatttttttgttaaaaagtgaa :  786
              GAAAAAGTGTTGGCATCTGCAATTTTCTTGAAATGCATCCCACATCATTGTTGCGATAAATAGTCATTATTCTTAATTAAAATCTCGGGCACAAATTTTTGCGATACAAATATTTTTTTGTTAAAAAGTGAA

                 *       800         *       820         *       840         *       860         *       880         *       900         *
ITAG.3      : ttcgaacataaaattacatcaacacactgtcactaatgaataactttactttgtcctggattgtagtagaacgttgacattatttcacccttaactaaaaaaaaaaatttcaatttaactgttaaatatatt :  498
BGV006865.  : ttcgaacataaaattacatcaacacactgtcactaatgaataactttactttgtcctggattgtagtagaacgttgacattatttcacccttaactaaaaaaaaaaatttcaatttaactgttaaatatatt :  498
BGV007931.  : ttcgaacataaaattacatcaacacactgtcactaatgaataactttactttgtcctggattgtagtagaacgttgacattatttcacccttaactaaaaaaaaaaatttcaatttaactgttaaatatatt :  498
BGV007989.  : ttcgaacataaaattacatcaacacactgtcactaatgaataactttactttgtcctggattgtagtagaacgttgacattatttcacccttaactaaaaaaaaaaatttcaatttaactgttaaatatatt :  498
M82.3       : ttcgaacataaaattacatcaacacactgtcactaatgaataactttactttgtcctggattgtagtagaacgttgacattatttcacccttaactaaaaaaaaaaatttcaatttaactgttaaatatatt :  498
EA00371.3   : ttcgaacataaaattacatcaacacactgtcactaatgaataactttactttgtcctggattgtagtagaacgttgacattatttcacccttaactaaaaaaaaaaatttcaatttaactgttaaatatatt :  498
Fla.8924.3  : ttcgaacataaaattacatcaacacactgtcactaatgaataactttactttgtcctggattgtagtagaacgttgacattatttcacccttaactaaaaaaaaaaatttcaatttaactgttaaatatatt :  496
Floradade.  : ttcgaacataaaattacatcaacacactgtcactaatgaataactttactttgtcctggattgtagtagaacgttgacattatttcacccttaactaaaaaaaaaaatttcaatttaactgttaaatatatt :  498
LYC1410.3   : ttcgaacataaaattacatcaacacactgtcactaatgaataactttactttgtcctggattgtagtagaacgttgacattatttcacccttaactaaaaaaaaaaatttcaatttaactgttaaatatatt :  498
PI169588.3  : ttcgaacataaaattacatcaacacactgtcactaatgaataactttactttgtcctggattgtagtagaacgttgacattatttcacccttaactaaaaaaaaaaatttcaatttaactgttaaatatatt :  498
Brandywine  : ttcgaacataaaattacatcaacacactgtcactaatgaataactttactttgtcctggattgtagtagaacgttgacattatttcacccttaactaaaaaaaaaaatttcaatttaactgttaaatatatt :  498
EA00990.3   : ttcgaacataaaattacatcaacacactgtcactaatgaataactttactttgtcctggattgtagtagaacgttgacattatttcacccttaactaaaaaaaaaaatttcaatttaactgttaaatatatt :  498
PI303721.3  : ttcgaacataaaattacatcaacacactgtcactaatgaataactttactttgtcctggattgtagtagaacgttgacattatttcacccttaactaaaaaaaaaaatttcaatttaactgttaaatatatt :  498
BGV006775.  : ttcgaacataaaattacatcaacacactgtcactaatgaataactttactttgtcctggattgtagtagaacgttgacattatttcacccttaactaaaaaaaaaaatttcaatgtaactgttaaatatatt :  917
PAS014479.  : ttcgaacataaaattacatcaacacactgtcactaatgaataactttactttgtcctggattgtagtagaacgttgacattatttcacccttaactaaaaaaaaaaatttcaatgtaactgttaaatatatt :  917
              TTCGAACATAAAATTACATCAACACACTGTCACTAATGAATAACTTTACTTTGTCCTGGATTGTAGTAGAACGTTGACATTATTTCACCCTTAACTAAAAAAAAAAATTTCAATtTAACTGTTAAATATATT

                920         *       940         *       960         *       980         *      1000         *      1020         *      1040
ITAG.3      : tttttaaaattaaaataattactttcgaataaatcttgcaacgtcttatgcagatatagacgaatatttaaccaaactaaattattatataaaacaatttaccaaagtaatacatttttctaaaatttac :  629
BGV006865.  : tttttaaaattaaaataattactttcgaataaatcttgcaacgtcttatgcagatatagacgaatatttaaccaaactaaattattatataaaacaatttaccaaagtaatacatttttctaaaatttac :  629
BGV007931.  : tttttaaaattaaaataattactttcgaataaatcttgcaacgtcttatgcagatatagacgaatatttaatcaaactaaattattatataaaacaatttaccaaagtaatacatttttctaaaatttac :  629
BGV007989.  : tttttaaaattaaaataattactttcgaataaatcttgcaacgtcttatgcagatatagacgaatatttaaccaaactaaattattatataaaacaatttaccaaagtaatacatttttctaaaatttac :  629
M82.3       : tttttaaaattaaaataattactttcgaataaatcttgcaacgtcttatgcagatatagacgaatatttaaccaaactaaattattatataaaacaatttaccaaagtaatacatttttctaaaatttac :  629
EA00371.3   : tttttaaaattaaaataattactttcgaataaatcttgcaacgtcttatgcagatatagacgaatatttaaccaaactaaattattatataaaacaatttaccaaagtaatacatttttctaaaatttac :  629
```

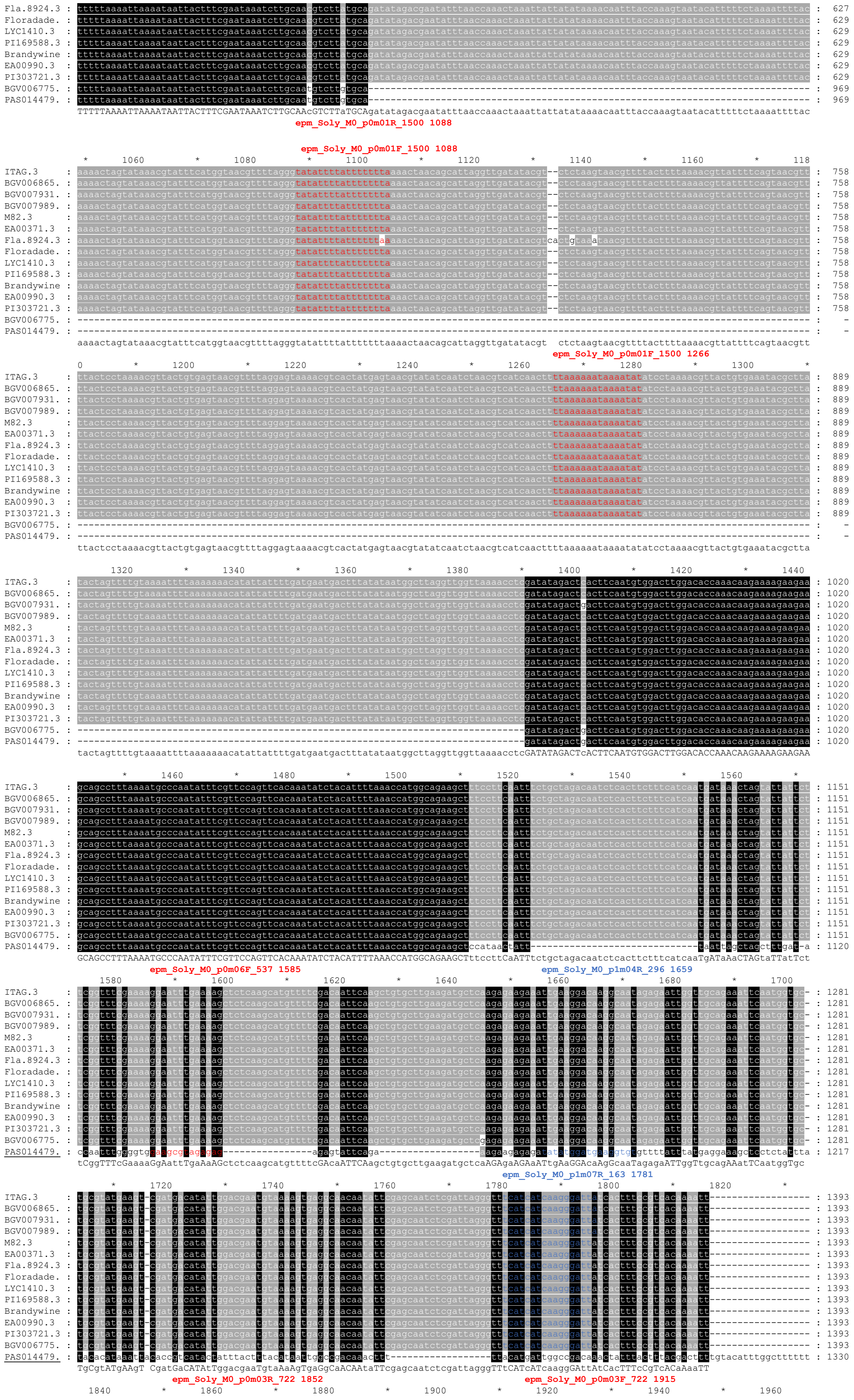

Fla.8924.3
Floradade.
LYC1410.3
PI169588.3
Brandywine
EA00990.3
PI303721.3
BGV006775.
PAS014479.
epm_Soly_M0_p0m01R_1500 1088
epm_Soly_M0_p0m01F_1500 1088
ITAG.3
BGV006865.
BGV007931.
BGV007989.
M82.3
EA00371.3
epm_Soly_M0_p0m01F_1500 1266
epm_Soly_M0_p0m06F_537 1585
epm_Soly_M0_p1m04R_296 1659
epm_Soly_M0_p1m07R_163 1781
epm_Soly_M0_p0m03R_722 1852
epm_Soly_M0_p0m03F_722 1915

```
ITAG.3      : ------------ggggaaacaggtatgagtacatctaaactatcatattttcgcgattttcagatctcaactatacgaacacattt-------------------------------------caatactg : 1475
BGV006865.  : ------------ggggaaacaggtatgagtacatctaaactatcatattttcgcgattttcagatctcaactatacgaacacattt-------------------------------------caatactg : 1475
BGV007931.  : ------------ggggaaacaggtatgagtacatctaaactatcatattttcgcgattttcagatctcaactatacgaacacattt-------------------------------------caatactg : 1475
BGV007989.  : ------------ggggaaacaggtatgagtacatctaaactatcatattttcgcgattttcagatctcaactatacgaacacattt-------------------------------------caatactg : 1475
M82.3       : ------------ggggaaacaggtatgagtacatctaaactatcatattttcgcgattttcagatctcaactatacgaacacattt-------------------------------------caatactg : 1475
EA00371.3   : ------------ggggaaacaggtatgagtacatctaaactatcatattttcgcgattttcagatctcaactatacgaacacattt-------------------------------------caatactg : 1475
Fla.8924.3  : ------------ggggaaacaggtatgagtacatctaaactatcatattttcgcgattttcagatctcaactatacgaacacattt-------------------------------------caatactg : 1475
Floradade.  : ------------ggggaaacaggtatgagtacatctaaactatcatattttcgcgattttcagatctcaactatacgaacacattt-------------------------------------caatactg : 1475
LYC1410.3   : ------------ggggaaacaggtatgagtacatctaaactatcatattttcgcgattttcagatctcaactatacgaacacattt-------------------------------------caatactg : 1475
PI169588.3  : ------------ggggaaacaggtatgagtacatctaaactatcatattttcgcgattttcagatctcaactatacgaacacattt-------------------------------------caatactg : 1475
Brandywine  : ------------ggggaaacaggtatgagtacatctaaactatcatattttcgcgattttcagatctcaactatacgaacacattt-------------------------------------caatactg : 1475
EA00990.3   : ------------ggggaaacaggtatgagtacatctaaactatcatattttcgcgattttcagatctcaactatacgaacacattt-------------------------------------caatactg : 1475
PI303721.3  : ------------ggggaaacaggtatgagtacatctaaactatcatattttcgcgattttcagatctcaactatacgaacacattt-------------------------------------caatactg : 1475
BGV006775.  : ------------ggggaaacaggtatgagtacatctaaactatcatattttcgcgattttcagatctcaactatacgaacacattt-------------------------------------caatactg : 1475
PAS014479.  : cagccgacgcaagacgacaaaaataaaaagacactcactttat-acatatggctgatattcagccaacaataataataataattttttttttttatcttctttttcttagtctattccagttgtactccgg : 1460
                          GggGAaAcAggTAtgAgtACAtctAaacTATcAtATtTtcgcGATtTTCAGatctCAActATAcgAAcAcaTTT                                     cAaTaCtG
```

epm_Soly_M0_p1m03R_719 1976

```
                 *     1980         *     2000         *     2020         *     2040         *     2060         *     2080         *
ITAG.3      : aactgttggtgcctctggtacagca---------------nnnnnnnnnnnnnnnnnnnnagccaatttatttgcttaagcaaatctctactctttatgtatgagctgggataggtccgatagatatgaat : 1591
BGV006865.  : aactgttggtgcctctggtacagca---------------nnnnnnnnnnnnnnnnnnnnagccaatttatttgcttaagcaaatctctactctttatgtatgagctgggataggtccgatagatatgaat : 1591
BGV007931.  : aactgttggtgcctctggtacagca---------------nnnnnnnnnnnnnnnnnnnnagccaatttatttgcttaagcaaatctctactctttatgtatgagctgggataggtccgatagatatgaat : 1591
BGV007989.  : aactgttggtgcctctggtacagca---------------nnnnnnnnnnnnnnnnnnnnagccaatttatttgcttaagcaaatctctactctttatgtatgagctgggataggtccgatagatatgaat : 1591
M82.3       : aactgttggtgcctctggtacagca---------------nnnnnnnnnnnnnnnnnnnnagccaatttatttgcttaagcaaatctctactctttatgtatgagctgggataggtccgatagatatgaat : 1591
EA00371.3   : aactgttggtgcctctggtacagca---------------nnnnnnnnnnnnnnnnnnnnagccaatttatttgcttaagcaaatctctactctttatgtatgagctgggataggtccgatagatatgaat : 1591
Fla.8924.3  : aactgttggtgcctctggtacagca---------------nnnnnnnnnnnnnnnnnnnnagccaatttatttgcttaagcaaatctctactctttatgtatgagctgggataggtccgatagatatgaat : 1591
Floradade.  : aactgttggtgcctctggtacagca---------------nnnnnnnnnnnnnnnnnnnnagccaatttatttgcttaagcaaatctctactctttatgtatgagctgggataggtccgatagatatgaat : 1591
LYC1410.3   : aactgttggtgcctctggtacagca---------------nnnnnnnnnnnnnnnnnnnnagccaatttatttgcttaagcaaatctctactctttatgtatgagctgggataggtccgatagatatgaat : 1591
PI169588.3  : aactgttggtgcctctggtacagca---------------nnnnnnnnnnnnnnnnnnnnagccaatttatttgcttaagcaaatctctactctttatgtatgagctgggataggtccgatagatatgaat : 1591
Brandywine  : aactgttggtgcctctggtacagca---------------nnnnnnnnnnnnnnnnnnnnagccaatttatttgcttaagcaaatctctactctttatgtatgagctgggataggtccgatagatatgaat : 1591
EA00990.3   : aactgttggtgcctctggtacagca---------------nnnnnnnnnnnnnnnnnnnnagccaatttatttgcttaagcaaatctctactctttatgtatgagctgggataggtccgatagatatgaat : 1591
PI303721.3  : aactgttggtgcctctggtacagca---------------nnnnnnnnnnnnnnnnnnnnagccaatttatttgcttaagcaaatctctactctttatgtatgagctgggataggtccgatagatatgaat : 1591
BGV006775.  : aactgttggtgcctctggtacagca---------------nnnnnnnnnnnnnnnnnnnnagccaatttatttgcttaagcaaatctctactctttatgtatgagctgggataggtccgatagatatgaat : 1591
PAS014479.  : ggacatagttatcttcgccattgcattttgaacatagatgnnnnnnnnnnnnnnnnnnnnagccaatttatttgcttaagcaaatctctactctttatgtatgagctgggataggtccgatagatatgaat : 1591
              aactgTtGgTgcCTctGgtAcaGCA               NNNNNNNNNNNNNNNNNNNNAGCCAATTTATTTGCTTAAGCAAATCTCTACTCTTTATGTATGAGCTGGGATAGGTCCGATAGATATGAAT
```

epm_Soly_M0_p0m18F_163 2014

```
              2100         *     2120         *     2140         *     2160         *     2180         *     2200         *     2220
ITAG.3      : cagaaaatgatttggatgaggaagtgcttaagtccctcaaaccacaccccaacctgaaatctttaaaagtaactggcttcagaggtctctgtctcccggactggatgaacgttcagttttgaaaaatgttg : 1722
BGV006865.  : cagaaaatgatttggatgaggaagtgcttaagtccctcaaaccacaccccaacctgaaatctttaaaagtaactggcttcagaggtctctgtctcccggactggatgaacgttcagttttgaaaaatgttg : 1722
BGV007931.  : cagaaaatgatttggatgaggaagtgcttaagtccctcaaaccacaccccaacctgaaatctttaaaagtaactggcttcagaggtctctgtctcccggactggatgaacgttcagttttgaaaaatgttg : 1722
BGV007989.  : cagaaaatgatttggatgaggaagtgcttaagtccctcaaaccacaccccaacctgaaatctttaaaagtaactggcttcagaggtctctgtctcccggactggatgaacgttcagttttgaaaaatgttg : 1722
M82.3       : cagaaaatgatttggatgaggaagtgcttaagtccctcaaaccacaccccaacctgaaatctttaaaagtaactggcttcagaggtctctgtctcccggactggatgaacgttcagttttgaaaaatgttg : 1722
EA00371.3   : cagaaaatgatttggatgaggaagtgcttaagtccctcaaaccacaccccaacctgaaatctttaaaagtaactggcttcagaggtctctgtctcccggactggatgaacgttcagttttgaaaaatgttg : 1722
Fla.8924.3  : cagaaaatgatttggatgaggaagtgcttaagtccctcaaaccacaccccaacctgaaatctttaaaagtaactggcttcagaggtctctgtctcccggactggatgaacgttcagttttgaaaaatgttg : 1722
Floradade.  : cagaaaatgatttggatgaggaagtgcttaagtccctcaaaccacaccccaacctgaaatctttaaaagtaactggcttcagaggtctctgtctcccggactggatgaacgttcagttttgaaaaatgttg : 1722
LYC1410.3   : cagaaaatgatttggatgaggaagtgcttaagtccctcaaaccacaccccaacctgaaatctttaaaagtaactggcttcagaggtctctgtctcccggactggatgaacgttcagttttgaaaaatgttg : 1722
PI169588.3  : cagaaaatgatttggatgaggaagtgcttaagtccctcaaaccacaccccaacctgaaatctttaaaagtaactggcttcagaggtctctgtctcccggactggatgaacgttcagttttgaaaaatgttg : 1722
Brandywine  : cagaaaatgatttggatgaggaagtgcttaagtccctcaaaccacaccccaacctgaaatctttaaaagtaactggcttcagaggtctctgtctcccggactggatgaacgttcagttttgaaaaatgttg : 1722
EA00990.3   : cagaaaatgatttggatgaggaagtgcttaagtccctcaaaccacaccccaacctgaaatctttaaaagtaactggcttcagaggtctctgtctcccggactggatgaacgttcagttttgaaaaatgttg : 1722
PI303721.3  : cagaaaatgatttggatgaggaagtgcttaagtccctcaaaccacaccccaacctgaaatctttaaaagtaactggcttcagaggtctctgtctcccggactggatgaacgttcagttttgaaaaatgttg : 1722
BGV006775.  : cagaaaatgatttggatgaggaagtgcttaagtccctcaaaccacaccccaacctgaaatctttaaaagtaactggcttcagaggtctctgtctcctggactggatgaacgttcagttttgaaaaatgttg : 1722
PAS014479.  : cagaaaatgatttggatgagtaagtgcttaagtccctcaaaccacaccccaacctgaaatctttaaaagtaactggcttcagaggtctctgtctcccggactggatgaacgttcagttttgaaaaatgttg : 1722
              CAGAAAATGATTTGGATGAGgAAGTGCTTAAGTCCCTCAAACCACACCCCAACCTGAAATCTTTAAAAGTAACTGGCTTCAGAGGTCTCTGTCTCCcGGACTGGATGAACGTTCAGTTTTGAAAAATGTTG

                *     2240         *     2260         *     2280         *     2300         *     2320         *     2340         *     23
ITAG.3      : tctctattgatgttgatagctgtaaaaactgtttacgccttccaccctttggagatctgccttgtctagaaagtttatgcttatttggcgggtctgtagattatattgaagacaatgttcatgactgtaga : 1853
BGV006865.  : tctctattgatgttgatagctgtaaaaactgtttacgccttccaccctttggagatctgccttgtctagaaagtttatgcttatttggcgggtctgtagattatattgaagacaatgttcatgactgtaga : 1853
BGV007931.  : tctctattgatgttgatagctgtaaaaactgtttacgccttccaccctttggagatctgccttgtctagaaagtttatgcttatttggcgggtctgtagattatattgaagacaatgttcatgactgtaga : 1853
BGV007989.  : tctctattgatgttgatagctgtaaaaactgtttacgccttccaccctttggagatctgccttgtctagaaagtttatgcttatttggcgggtctgtagattatattgaagacaatgttcatgactgtaga : 1853
M82.3       : tctctattgatgttgatagctgtaaaaactgtttacgccttccaccctttggagatctgccttgtctagaaagtttatgcttatttggcgggtctgtagattatattgaagacaatgttcatgactgtaga : 1853
EA00371.3   : tctctattgatgttgatagctgtaaaaactgtttacgccttccaccctttggagatctgccttgtctagaaagtttatgcttatttggcgggtctgtagattatattgaagacaatgttcatgactgtaga : 1853
Fla.8924.3  : tctctattgatgttgatagctgtaaaaactgtttacgccttccaccctttggagatctgccttgtctagaaagtttatgcttatttggcgggtctgtagattatattgaagacaatgttcatgactgtaga : 1853
Floradade.  : tctctattgatgttgatagctgtaaaaactgtttacgccttccaccctttggagatctgccttgtctagaaagtttatgcttatttggcgggtctgtagattatattgaagacaatgttcatgactgtaga : 1853
LYC1410.3   : tctctattgatgttgatagctgtaaaaactgtttacgccttccaccctttggagatctgccttgtctagaaagtttatgcttatttggcgggtctgtagattatattgaagacaatgttcatgactgtaga : 1853
PI169588.3  : tctctattgatgttgatagctgtaaaaactgtttacgccttccaccctttggagatctgccttgtctagaaagtttatgcttatttggcgggtctgtagattatattgaagacaatgttcatgactgtaga : 1853
Brandywine  : tctctattgatgttgatagctgtaaaaactgtttacgccttccaccctttggagatctgccttgtctagaaagtttatgcttatttggcgggtctgtagattatattgaagacaatgttcatgactgtaga : 1853
EA00990.3   : tctctattgatgttgatagctgtaaaaactgtttacgccttccaccctttggagatctgccttgtctagaaagtttatgcttatttggcgggtctgtagattatattgaagacaatgttcatgactgtaga : 1853
PI303721.3  : tctctattgatgttgatagctgtaaaaactgtttacgccttccaccctttggagatctgccttgtctagaaagtttatgcttatttggcgggtctgtagattatattgaagacaatgttcatgactgtaga : 1853
BGV006775.  : tctctattgatgttgatagctgtaaaaactgtttacgccttccaccctttggagatctgccttgtctagaaagtttatgcttatttggcgggtctgtagattatattgaagacaatgttcatgactgtaga : 1853
PAS014479.  : tctctattgatgttgatagctgtaaaaactgtttacgccttccaccctttggagatctgccttgtctagaaagtttatgcttatttggcgggtctgtagattatattgaagacaatgttcatgactgtaga : 1853
              TCTCTATTGATGTTGATAGCTGTAAAAACTGTTTACGCCTTCCACCCTTTGGAGATCTGCCTTGTCTAGAAAGTTTATGCTTATTTGGCGGGTCTGTAGATTATATTGAAGACAATGTTCATGACTGTAGA

              60         *     2380         *     2400         *     2420         *     2440         *     2460         *     2480
ITAG.3      : aggtttccatccttgcgatgccttgtcataaaaggctttccgaacctgaaaggattgctacaaaaggaagcaaaggatcaatttcccatactcgaggaaatggagaatcatgattgcactatgctcgtctt : 1984
BGV006865.  : aggtttccatccttgcgatgccttgtcataaaaggctttccgaacctgaaaggattgctacaaaaggaagcaaaggatcaatttcccatactcgaggaaatggagaatcatgattgcactatgctcgtctt : 1984
BGV007931.  : aggtttccatccttgcgatgccttgtcataaaaggctttccgaacctgaaaggattgctacaaaaggaagcaaaggatcaatttcccatactcgaggaaatggagaatcatgattgcactatgctcgtctt : 1984
BGV007989.  : aggtttccatccttgcgatgccttgtcataaaaggctttccgaacctgaaaggattgctacaaaaggaagcaaaggatcaatttcccatactcgaggaaatggagaatcatgattgcactatgctcgtctt : 1984
M82.3       : aggtttccatccttgcgatgccttgtcataaaaggctttccgaacctgaaaggattgctacaaaaggaagcaaaggatcaatttcccatactcgaggaaatggagaatcatgattgcactatgctcgtctt : 1984
EA00371.3   : aggtttccatccttgcgatgccttgtcataaaaggctttccgaacctgaaaggattgctacaaaaggaagcaaaggatcaatttcccatactcgaggaaatggagaatcatgattgcactatgctcgtctt : 1984
Fla.8924.3  : aggtttccatccttgcgatgccttgtcataaaaggctttccgaacctgaaaggattgctacaaaaggaagcaaaggatcaatttcccatactcgaggaaatggagaatcatgattgcactatgctcgtctt : 1984
Floradade.  : aggtttccatccttgcgatgccttgtcataaaaggctttccgaacctgaaaggattgctacaaaaggaagcaaaggatcaatttcccatactcgaggaaatggagaatcatgattgcactatgctcgtctt : 1984
LYC1410.3   : aggtttccatccttgcgatgccttgtcataaaaggctttccgaacctgaaaggattgctacaaaaggaagcaaaggatcaatttcccatactcgaggaaatggagaatcatgattgcactatgctcgtctt : 1984
PI169588.3  : aggtttccatccttgcgatgccttgtcataaaaggctttccgaacctgaaaggattgctacaaaaggaagcaaaggatcaatttcccatactcgaggaaatggagaatcatgattgcactatgctcgtctt : 1984
Brandywine  : aggtttccatccttgcgatgccttgtcataaaaggctttccgaacctgaaaggattgctacaaaaggaagcaaaggatcaatttcccatactcgaggaaatggagaatcatgattgcactatgctcgtctt : 1984
EA00990.3   : aggtttccatccttgcgatgccttgtcataaaaggctttccgaacctgaaaggattgctacaaaaggaagcaaaggatcaatttcccatactcgaggaaatggagaatcatgattgcactatgctcgtctt : 1984
PI303721.3  : aggtttccatccttgcgatgccttgtcataaaaggctttccgaacctgaaaggattgctacaaaaggaagcaaaggatcaatttcccatactcgaggaaatggagaatcatgattgcactatgctcgtctt : 1984
BGV006775.  : aggtttccatccttgcgatgccttgtcataaaaggctttccgaacctgaaaggattgctacaaaaggaagcaaaggatcaatttcccatactcgaggaaatggagaatcatgattgcactatgctcgtctt : 1984
PAS014479.  : aggtttccatccttgcgatgccttgtcataaaaggctttccgaacctgaaaggattgctacaaaaggaagcaaaggatcaatttcccatactcgaggaaatggagaatcatgattgcactatgctcgtctt : 1984
              AGGTTTCCATCCTTGCGATGCCTTGTCATAAAAGGCTTTCCGAACCTGAAAGGATTGCTACAAAAGGAAGCAAAGGATCAATTTCCCATACTCGAGGAAATGGAGAATCATGATTGCACTATGCTCGTCTT

               *     2500         *     2520         *     2540         *     2560         *     2580         *     2600         *     2620
ITAG.3      : tcaaacccttgcttgtgtcaagaaattagaagtttgaggcaaagacagatcacagatgcaacgactttgagctccatatctgatcttagcactctcaccttgttgtatattagtcacaatgccgaacagac : 2115
BGV006865.  : tcaaacccttgcttgtgtcaagaaattagaagtttgaggcaaagacagatcacagatgcaacgactttgagctccatatctgatcttagcactctcaccttgttgtatattagtcacaatgccgaacagac : 2115
BGV007931.  : tcaaacccttgcttgtgtcaagaaattagaagtttgaggcaaagacagatcacagatgcaacgactttgagctccatatctgatcttagcactctcaccttgttgtatattagtcacaatgccgaacagac : 2115
BGV007989.  : tcaaacccttgcttgtgtcaagaaattagaagtttgaggcaaagacagatcacagatgcaacgactttgagctccatatctgatcttagcactctcaccttgttgtatattagtcacaatgccgaacagac : 2115
M82.3       : tcaaacccttgcttgtgtcaagaaattagaagtttgaggcaaagacagatcacagatgcaacgactttgagctccatatctgatcttagcactctcaccttgttgtatattagtcacaatgccgaacagac : 2115
EA00371.3   : tcaaacccttgcttgtgtcaagaaattagaagtttgaggcaaagacagatcacagatgcaacgactttgagctccatatctgatcttagcactctcaccttgttgtatattagtcacaatgccgaacagac : 2115
Fla.8924.3  : tcaaacccttgcttgtgtcaagaaattagaagtttgaggcaaagacagatcacagatgcaacgactttgagctccatatctgatcttagcactctcaccttgttgtatattagtcacaatgccgaacagac : 2115
Floradade.  : tcaaacccttgcttgtgtcaagaaattagaagtttgaggcaaagacagatcacagatgcaacgactttgagctccatatctgatcttagcactctcaccttgttgtatattagtcacaatgccgaacagac : 2115
LYC1410.3   : tcaaacccttgcttgtgtcaagaaattagaagtttgaggcaaagacagatcacagatgcaacgactttgagctccatatctgatcttagcactctcaccttgttgtatattagtcacaatgccgaacagac : 2115
PI169588.3  : tcaaacccttgcttgtgtcaagaaattagaagtttgaggcaaagacagatcacagatgcaacgactttgagctccatatctgatcttagcactctcaccttgttgtatattagtcacaatgccgaacagac : 2115
Brandywine  : tcaaacccttgcttgtgtcaagaaattagaagtttgaggcaaagacagatcacagatgcaacgactttgagctccatatctgatcttagcactctcaccttgttgtatattagtcacaatgccgaacagac : 2115
EA00990.3   : tcaaacccttgcttgtgtcaagaaattagaagtttgaggcaaagacagatcacagatgcaacgactttgagctccatatctgatcttagcactctcaccttgttgtatattagtcacaatgccgaacagac : 2115
PI303721.3  : tcaaacccttgcttgtgtcaagaaattagaagtttgaggcaaagacagatcacagatgcaacgactttgagctccatatctgatcttagcactctcaccttgttgtatattagtcacaatgccgaacagac : 2115
BGV006775.  : tcaaacccttgcttgtgtcaagaaattagaagtttgaggcaaagacagatcacagatgcaacgactttgagctccatatctgatctttgcactctcaccttgttgtatattagtcacaatgccgaacagac : 2115
PAS014479.  : tcaaacccttgcttgtgtcaagaaattagaagtttgaggcaaagacagatcacagatgcaacgactttgagctccatatctgatcttagcactctcaccttgttgtatattagtcacaatgccgaacagac : 2115
              TCAAACCCTTGCTTGTGTCAAGAAATTAGAAGTTTGAGGCAAAGACAGATCACAGATGCAACGACTTTGAGCTCCATATCTGATCTTaGCACTCTCACCTTGTTGTATATTAGTCACAATGCCGAACAGAC

                       *     2640         *     2660         *     2680         *     2700         *     2720         *     2740         *
ITAG.3      : ttcaccctagaagaaatgttcaaacaccttgcctatctcgagtccacgagtattacatccttcaaaaaacttgaagagctgcctaccagcatggccagtctaactgctttgaagcgtttggatattcgaag : 2246
BGV006865.  : ttcaccctagaagaaatgttcaaacaccttgcctatctcgagtccacgagtattacatccttcaaaaaacttgaagagctgcctaccagcatggccagtctaactgctttgaagcgtttggatattcgaag : 2246
BGV007931.  : ttcaccctagaagaaatgttcaaacaccttgcctatctcgagtccacgagtattacatccttcaaaaaacttgaagagctgcctaccagcatggccagtctaactgctttgaagcgtttggatattcgaag : 2246
BGV007989.  : ttcaccctagaagaaatgttcaaacaccttgcctatctcgagtccacgagtattacatccttcaaaaaacttgaagagctgcctaccagcatggccagtctaactgctttgaagcgtttggatattcgaag : 2246
M82.3       : ttcaccctagaagaaatgttcaaacaccttgcctatctcgagtccacgagtattacatccttcaaaaaacttgaagagctgcctaccagcatggccagtctaactgctttgaagcgtttggatattcgaag : 2246
EA00371.3   : ttcaccctagaagaaatgttcaaacaccttgcctatctcgagtccacgagtattacatccttcaaaaaacttgaagagctgcctaccagcatggccagtctaactgctttgaagcgtttggatattcgaag : 2246
Fla.8924.3  : ttcaccctagaagaaatgttcaaacaccttgcctatctcgagtccacgagtattacatccttcaaaaaacttgaagagctgcctaccagcatggccagtctaactgctttgaagcgtttggatattcgaag : 2246
Floradade.  : ttcaccctagaagaaatgttcaaacaccttgcctatctcgagtccacgagtattacatccttcaaaaaacttgaagagctgcctaccagcatggccagtctaactgctttgaagcgtttggatattcgaag : 2246
LYC1410.3   : ttcaccctagaagaaatgttcaaacaccttgcctatctcgagtccacgagtattacatccttcaaaaaacttgaagagctgcctaccagcatggccagtctaactgctttgaagcgtttggatattcgaag : 2246
PI169588.3  : ttcaccctagaagaaatgttcaaacaccttgcctatctcgagtccacgagtattacatccttcaaaaaacttgaagagctgcctaccagcatggccagtctaactgctttgaagcgtttggatattcgaag : 2246
Brandywine  : ttcaccctagaagaaatgttcaaacaccttgcctatctcgagtccacgagtattacatccttcaaaaaacttgaagagctgcctaccagcatggccagtctaactgctttgaagcgtttggatattcgaag : 2246
EA00990.3   : ttcaccctagaagaaatgttcaaacaccttgcctatctcgagtccacgagtattacatccttcaaaaaacttgaagagctgcctaccagcatggccagtctaactgctttgaagcgtttggatattcgaag : 2246
PI303721.3  : ttcaccctagaagaaatgttcaaacaccttgcctatctcgagtccacgagtattacatccttcaaaaaacttgaagagctgcctaccagcatggccagtctaactgctttgaagcgtttggatattcgaag : 2246
BGV006775.  : ttcaccctagaagaaatgttcaaacaccttgcctatctcgagtccacgagtatttcatccttcaaaaaacttaaagagctgcctaccagcatggccagtctaactgctttgaagcgtttggatattcgaag : 2246
```

```
PAS014479. : ttcaccctagaagaaatgttcaaacaccttgcctatctcgagtccacgagtatttcatccttcaaaaaacttaaagagctgcctaccagcatggtcagtctaactgctttgaagcgtttggatattcgaag : 2246
             TTCACCCTAGAAGAAATGTTCAAACACCTTGCCTATCTCGAGTCCACGAGTATTaCATCCTTCAAAAAACTTgAAGAGCTGCCTACCAGCATGGcCAGTCTAACTGCTTTGAAGCGTTTGGATATTCGAAG

                  2760         *       2780         *       2800         *       2820         *       2840         *       2860         *       2880
ITAG.3      : ttgtcattcactagagagtctccctgagcaagtactggaaggtttaacttcactcatggagttattcattcaggattgtgagatgctaaagactatcagagggattgcaacacctaacaacccttacaaga : 2377
BGV006865.  : ttgtcattcactagagagtctccctgagcaagtactggaaggtttaacttcactcatggagttattcattcaggattgtgagatgctaaagactatcagagggattgcaacacctaacaacccttacaaga : 2377
BGV007931.  : ttgtcattcactagagagtctccctgagcaagtactggaaggtttaacttcactcatggagttattcattcaggattgtgagatgctaaagactatcagagggattgcaacacctaacaacccttacaaga : 2377
BGV007989.  : ttgtcattcactagagagtctccctgagcaagtactggaaggtttaacttcactcatggagttattcattcaggattgtgagatgctaaagactatcagagggattgcaacacctaacaacccttacaaga : 2377
M82.3       : ttgtcattcactagagagtctccctgagcaagtactggaaggtttaacttcactcatggagttattcattcaggattgtgagatgctaaagactatcagagggattgcaacacctaacaacccttacaaga : 2377
EA00371.3   : ttgtcattcactagagagtctccctgagcaagtactggaaggtttaacttcactcatggagttattcattcaggattgtgagatgctaaagactatcagagggattgcaacacctaacaacccttacaaga : 2377
Fla.8924.3  : ttgtcattcactagagagtctccctgagcaagtactggaaggtttaacttcactcatggagttattcattcaggattgtgagatgctaaagactatcagagggattgcaacacctaacaacccttacaaga : 2377
Floradade.  : ttgtcattcactagagagtctccctgagcaagtactggaaggtttaacttcactcatggagttattcattcaggattgtgagatgctaaagactatcagagggattgcaacacctaacaacccttacaaga : 2377
LYC1410.3   : ttgtcattcactagagagtctccctgagcaagtactggaaggtttaacttcactcatggagttattcattcaggattgtgagatgctaaagactatcagagggattgcaacacctaacaacccttacaaga : 2377
PI169588.3  : ttgtcattcactagagagtctccctgagcaagtactggaaggtttaacttcactcatggagttattcattcaggattgtgagatgctaaagactatcagagggattgcaacacctaacaacccttacaaga : 2377
Brandywine  : ttgtcattcactagagagtctccctgagcaagtactggaaggtttaacttcactcatggagttattcattcaggattgtgagatgctaaagactatcagagggattgcaacacctaacaacccttacaaga : 2377
EA00990.3   : ttgtcattcactagagagtctccctgagcaagtactggaaggtttaacttcactcatggagttattcattcaggattgtgagatgctaaagactatcagagggattgcaacacctaacaacccttacaaga : 2377
PI303721.3  : ttgtcattcactagagagtctccctgagcaagtactggaaggtttaacttcactcatggagttattcattcaggattgtgagatgctaaagactatcagagggattgcaacacctaacaacccttacaaga : 2377
BGV006775.  : ttgtcattcactagagagtctccctgagcaagtactggaaggtttaacttcactcacggagttattcattcaggattgtgagatgctaaagactatcagagggattgcaacacctaacaacccttacaaga : 2377
PAS014479.  : ttgtcattcactagagagtctccctgagcaagtactggaaggtttaacttcactcacggagttattcattcaggattgtgagatgctaaagactatcagagggattgcaacacctaacaacccttacaaga : 2377
              TTGTCATTCACTAGAGAGTCTCCCTGAGCAAGTACTGGAAGGTTTAACTTCACTCAtGGAGTTATTCATTCAGGATTGTGAGATGCTAAAGACTATCAGAGGGATTGCAACACCTAACAACCCTTACAAGA

                     *       2900         *       2920         *       2940         *       2960         *       2980         *       3000         *
ITAG.3      : ctagtagttgctctttgtccagagatggtgaccttgcctttgaggattcaaaacttacactctcttcaatcactggtgatctggaattgccctcgtatctgcaatcacttccagctgggattatggacaca : 2508
BGV006865.  : ctagtagttgctctttgtccagagatggtgaccttgcctttgaggattcaaaacttacactctcttcaatcactggtgatctggaattgccctcgtatctgcaatcacttccagctgggattatggacaca : 2508
BGV007931.  : ctagtagttgctctttgtccagagatggtgaccttgcctttgaggattcaaaacttacactctcttcaatcactggtgatctggaattgccctcgtatctgcaatcacttccagctgggattatggacaca : 2508
BGV007989.  : ctagtagttgctctttgtccagagatggtgaccttgcctttgaggattcaaaacttacactctcttcaatcactggtgatctggaattgccctcgtatctgcaatcacttccagctgggattatggacaca : 2508
M82.3       : ctagtagttgctctttgtccagagatggtgaccttgcctttgaggattcaaaacttacactctcttcaatcactggtgatctggaattgccctcgtatctgcaatcacttccagctgggattatggacaca : 2508
EA00371.3   : ctagtagttgctctttgtccagagatggtgaccttgcctttgaggattcaaaacttacactctcttcaatcactggtgatctggaattgccctcgtatctgcaatcacttccagctgggattatggacaca : 2508
Fla.8924.3  : ctagtagttgctctttgtccagagatggtgaccttgcctttgaggattcaaaacttacactctcttcaatcactggtgatctggaattgccctcgtatctgcaatcacttccagctgggattatggacaca : 2508
Floradade.  : ctagtagttgctctttgtccagagatggtgaccttgcctttgaggattcaaaacttacactctcttcaatcactggtgatctggaattgccctcgtatctgcaatcacttccagctgggattatggacaca : 2508
LYC1410.3   : ctagtagttgctctttgtccagagatggtgaccttgcctttgaggattcaaaacttacactctcttcaatcactggtgatctggaattgccctcgtatctgcaatcacttccagctgggattatggacaca : 2508
PI169588.3  : ctagtagttgctctttgtccagagatggtgaccttgcctttgaggattcaaaacttacactctcttcaatcactggtgatctggaattgccctcgtatctgcaatcacttccagctgggattatggacaca : 2508
Brandywine  : ctagtagttgctctttgtccagagatggtgaccttgcctttgaggattcaaaacttacactctcttcaatcactggtgatctggaattgccctcgtatctgcaatcacttccagctgggattatggacaca : 2508
EA00990.3   : ctagtagttgctctttgtccagagatggtgaccttgcctttgaggattcaaaacttacactctcttcaatcactggtgatctggaattgccctcgtatctgcaatcacttccagctgggattatggacaca : 2508
PI303721.3  : ctagtagttgctctttgtccagagatggtgaccttgcctttgaggattcaaaacttacactctcttcaatcactggtgatctggaattgccctcgtatctgcaatcacttccagctgggattatggacaca : 2508
BGV006775.  : ctagtagttgctctttgtccagagatggtgaccttgcctttgaggattcaaaacttacactctcttcaatcactggtgatctggaattgccctcgtatctgcaatcacttccagctgggattatggacaca : 2508
PAS014479.  : ctagtagttgctctttgtccagagatggtgaccttgcctttgaggattcaaaacttacactctcttcaatcactggtgatctggaattgccctcgtatctgcaatcacttccagctgggattatggacaca : 2508
              CTAGTAGTTGCTCTTTGTCCAGAGATGGTGACCTTGCCTTTGAGGATTCAAAACTTACACTCTCTTCAATCACTGGTGATCTGGAATTGCCCTCGTATCTGCAATCACTTCCAGCTGGGATTATGGACACA

                3020         *       3040         *       3060         *       3080         *       3100         *       3120         *       3140
ITAG.3      : gagaatctgcaagcatcgcgaattgtttattgtccagaactggcaaagcgctgcgggaaggagataggggaggactggaacaaaattgctcacattccaaatgtgcaagcatatcagttagttttatttgt : 2639
BGV006865.  : gagaatctgcaagcatcgcgaattgtttattgtccagaactggcaaagcgctgcgggaaggagataggggaggactggaacaaaattgctcacattccaaatgtgcaagcatatcagttagttttatttgt : 2639
BGV007931.  : gagaatctgcaagcatcgcgaattgtttattgtccagaactggcaaagcgctgcgggaaggagataggggaggactggaacaaaattgctcacattccaaatgtgcaagcatatcagttagttttatttgt : 2639
BGV007989.  : gagaatctgcaagcatcgcgaattgtttattgtccagaactggcaaagcgctgcgggaaggagataggggaggactggaacaaaattgctcacattccaaatgtgcaagcatatcagttagttttatttgt : 2639
M82.3       : gagaatctgcaagcatcgcgaattgtttattgtccagaactggcaaagcgctgcgggaaggagataggggaggactggaacaaaattgctcacattccaaatgtgcaagcatatcagttagttttatttgt : 2639
EA00371.3   : gagaatctgcaagcatcgcgaattgtttattgtccagaactggcaaagcgctgcgggaaggagataggggaggactggaacaaaattgctcacattccaaatgtgcaagcatatcagttagttttatttgt : 2639
Fla.8924.3  : gagaatctgcaagcatcgcgaattgtttattgtccagaactggcaaagcgctgcgggaaggagataggggaggactggaacaaaattgctcacattccaaatgtgcaagcatatcagttagttttatttgt : 2639
Floradade.  : gagaatctgcaagcatcgcgaattgtttattgtccagaactggcaaagcgctgcgggaaggagataggggaggactggaacaaaattgctcacattccaaatgtgcaagcatatcagttagttttatttgt : 2639
LYC1410.3   : gagaatctgcaagcatcgcgaattgtttattgtccagaactggcaaagcgctgcgggaaggagataggggaggactggaacaaaattgctcacattccaaatgtgcaagcatatcagttagttttatttgt : 2639
PI169588.3  : gagaatctgcaagcatcgcgaattgtttattgtccagaactggcaaagcgctgcgggaaggagataggggaggactggaacaaaattgctcacattccaaatgtgcaagcatatcagttagttttatttgt : 2639
Brandywine  : gagaatctgcaagcatcgcgaattgtttattgtccagaactggcaaagcgctgcgggaaggagataggggaggactggaacaaaattgctcacattccaaatgtgcaagcatatcagttagttttatttgt : 2639
EA00990.3   : gagaatctgcaagcatcgcgaattgtttattgtccagaactggcaaagcgctgcgggaaggagataggggaggactggaacaaaattgctcacattccaaatgtgcaagcatatcagttagttttatttgt : 2639
PI303721.3  : gagaatctgcaagcatcgcgaattgtttattgtccagaactggcaaagcgctgcgggaaggagataggggaggactggaacaaaattgctcacattccaaatgtgcaagcatatcagttagttttatttgt : 2639
BGV006775.  : gagaatctgcaagcatcgcgaattgtttattgtccagaactggcaaagcgctgcgggaaggagataggggaggactggaacaaaattgctcacattccaaatgtgcaagcatatcagttagttttatttgt : 2639
PAS014479.  : gagaatctgcaagcatcgcgaattgtttattgtccagaactggcaaagcgctgcgggaaggagataggggaggactggaacaaaattgctcacattccaaatgtgcaagcatatcagttagttttatttgt : 2639
              GAGAATCTGCAAGCATCGCGAATTGTTTATTGTCCAGAACTGGCAAAGCGCTGCGGGAAGGAGATAGGGGAGGACTGGAACAAAATTGCTCACATTCCAAATGTGCAAGCATATCAGTTAGTTTTATTTGT

                     *       3160         *       3180         *       3200         *       3220         *       3240         *       3260         *
ITAG.3      : agttcttcttccaggtaagccttccatatagttgacataactgtgatgcagcaatgcatgagggtattaggatatcttgtagtaactaaaatcttttggcatgctgtgtcgcgtgcatgctagacttatca : 2770
BGV006865.  : agttcttcttccaggtaagccttccatatagttgacataactgtgatgcagcaatgcatgagggtattaggatatcttgtagtaactaaaatcttttggcatgctgtgtcgcgtgcatgctagacttatca : 2770
BGV007931.  : agttcttcttccaggtaagccttccatatagttgacataactgtgatgcagcaatgcatgagggtattaggatatcttgtagtaactaaaatcttttggcatgctgtgtcgcgtgcatgctagacttatca : 2770
BGV007989.  : agttcttcttccaggtaagccttccatatagttgacataactgtgatgcagcaatgcatgagggtattaggatatcttgtagtaactaaaatcttttggcatgctgtgtcgcgtgcatgctagacttatca : 2770
M82.3       : agttcttcttccaggtaagccttccatatagttgacataactgtgatgcagcaatgcatgagggtattaggatatcttgtagtaactaaaatcttttggcatgctgtgtcgcgtgcatgctagacttatca : 2770
EA00371.3   : agttcttcttccaggtaagccttccatatagttgacataactgtgatgcagcaatgcatgagggtattaggatatcttgtagtaactaaaatcttttggcatgctgtgtcgcgtgcatgctagacttatca : 2770
Fla.8924.3  : agttcttcttccaggtaagccttccatatagttgacataactgtgatgcagcaatgcatgagggtattaggatatcttgtagtaactaaaatcttttggcatgctgtgtcgcgtgcatgctagacttatca : 2770
Floradade.  : agttcttcttccaggtaagccttccatatagttgacataactgtgatgcagcaatgcatgagggtattaggatatcttgtagtaactaaaatcttttggcatgctgtgtcgcgtgcatgctagacttatca : 2770
LYC1410.3   : agttcttcttccaggtaagccttccatatagttgacataactgtgatgcagcaatgcatgagggtattaggatatcttgtagtaactaaaatcttttggcatgctgtgtcgcgtgcatgctagacttatca : 2770
PI169588.3  : agttcttcttccaggtaagccttccatatagttgacataactgtgatgcagcaatgcatgagggtattaggatatcttgtagtaactaaaatcttttggcatgctgtgtcgcgtgcatgctagacttatca : 2770
Brandywine  : agttcttcttccaggtaagccttccatatagttgacataactgtgatgcagcaatgcatgagggtattaggatatcttgtagtaactaaaatcttttggcatgctgtgtcgcgtgcatgctagacttatca : 2770
EA00990.3   : agttcttcttccaggtaagccttccatatagttgacataactgtgatgcagcaatgcatgagggtattaggatatcttgtagtaactaaaatcttttggcatgctgtgtcgcgtgcatgctagacttatca : 2770
PI303721.3  : agttcttcttccaggtaagccttccatatagttgacataactgtgatgcagcaatgcatgagggtattaggatatcttgtagtaactaaaatcttttggcatgctgtgtcgcgtgcatgctagacttatca : 2770
BGV006775.  : agttcttcttccaggtaagccttccatatagttgacataactgtgatgcagcaatgcatgagggtattaggatatcttgtagtaactaaaatcttttggcatgctgtgtcgcgtgcatgctagacttatca : 2770
PAS014479.  : agttcttcttccaggtaagccttccatatagttgacataactgtgatgcagcaatgcatgagggtattaggatatcttgtagtaactaaaatcttttggcatgctgtgtcgcgtgcatgctagacttatca : 2770
              AGTTCTTCTTCCAGGTAAGCCTTCCATATAGTTGACATAACTGTGATGCAGCAATGCATGAGGGTATTAGGATATCTTGTAGTAACTAAAATCTTTTGGCATGCTGTGTCGCGTGCATGCTAGACTTATCA

                3280         *       3300         *       3320         *       3340         *       3360         *       3380         *       3400
ITAG.3      : ccaaaaagctattcattctctagattgttgtagtactagtgttatattgcacaattttaatgttttgctgagtaaaatggattgccattttgcagatgaatcatattggaaagcaatgaataaaaaaatc : 2901
BGV006865.  : ccaaaaagctattcattctctagattgttgtagtactagtgttatattgcacaattttaatgttttgctgagtaaaatggattgccattttgcagatgaatcatattggaaagcaatgaataaaaaaatc : 2901
BGV007931.  : ccaaaaagctattcattctctagattgttgtagtactagtttatattgcacaattttaatgttttgctgagtaaaatggattgccattttgcagatgaatcatattggaaagcaatgaataaataaatc : 2901
BGV007989.  : ccaaaaagctattcattctctagattgttgtagtactagtgttatattgcacaattttaatgttttgctgagtaaaatggattgccattttgcagatgaatcatattggaaagcaatgaataaaaaaatc : 2901
M82.3       : ccaaaaagctattcattctctagattgttgtagtactagtgttatattgcacaattttaatgttttgctgagtaaaatggattgccattttgcagatgaatcatattggaaagcaatgaataaaaaaatc : 2901
EA00371.3   : ccaaaaagctattcattctctagattgttgtagtactagtgttatattgcacaattttaatgttttgctgagtaaaatggattgccattttgcagatgaatcatattggaaagcaatgaataaaaaaatc : 2901
Fla.8924.3  : ccaaaaagctattcattctctagattgttgtagtactagtgttatattgcacaattttaatgttttgctgagtaaaatggattgccattttgcagatgaatcatattggaaagcaatgaataaaaaaatc : 2901
Floradade.  : ccaaaaagctattcattctctagattgttgtagtactagtgttatattgcacaattttaatgttttgctgagtaaaatggattgccattttgcagatgaatcatattggaaagcaatgaataaaaaaatc : 2901
LYC1410.3   : ccaaaaagctattcattctctagattgttgtagtactagtgttatattgcacaattttaatgttttgctgagtaaaatggattgccattttgcagatgaatcatattggaaagcaatgaataaaaaaatc : 2901
PI169588.3  : ccaaaaagctattcattctctagattgttgtagtactagtgttatattgcacaattttaatgttttgctgagtaaaatggattgccattttgcagatgaatcatattggaaagcaatgaataaaaaaatc : 2901
Brandywine  : ccaaaaagctattcattctctagattgttgtagtactagtgttatattgcacaattttaatgttttgctgagtaaaatggattgccattttgcagatgaatcatattggaaagcaatgaataaaaaaatc : 2901
EA00990.3   : ccaaaaagctattcattctctagattgttgtagtactagtgttatattgcacaattttaatgttttgctgagtaaaatggattgccattttgcagatgaatcatattggaaagcaatgaataaaaaaatc : 2901
PI303721.3  : ccaaaaagctattcattctctagattgttgtagtactagtgttatattgcacaattttaatgttttgctgagtaaaatggattgccattttgcagatgaatcatattggaaagcaatgaataaaaaaatc : 2901
BGV006775.  : ccaaaaagctattcattctctagattgttgtagtactagtgttatattgcacaattttaatgttttgctgagtaaaatggattgccattttgcagatgaatcatattggaaagcaatgaataaaaaaatc : 2901
PAS014479.  : ccaaaaagctattcattctctagattgttgtagtactagtgttatattgcacaattttaatgttttgctgagtaaaatggattgccattttgcagatgaatcatgttggaaagcaatgaataaaaaaatc : 2901
              CCAAAAAGCTATTCATTCTCTAGATTGTTGTAGTACTAGTgTTATATTGCACAATTTTAATGTTTTGCTGAGTAAAATGGATTGCCATTTTGCAGATGAATCATaTTGGAAAGCAATGAATAAAaAAATC

                     *       3420         *       3440         *       3460         *       3480         *       3500         *       3520
ITAG.3      : atattggccaatttctttcactttacctaatacaagctaataataacacatggaaaagaaaattatatgctgacttgtgtaacttaaagaacaataattttcttgaaccaagcatgtga : 3020
BGV006865.  : atattggccaatttctttcactttacctaatacaagctaataataacacatggaaaagaaaattatatgctgacttgtgtaacttaaagaacaataattttcttgaaccaagcatgtga : 3020
BGV007931.  : atattggccaatttctttcactttacctaatacaagctaataataacacatggaaaagaaaattatatgctgacttgtgtaacttaaagaacaataattttcttgaaccaagcatgtga : 3020
BGV007989.  : atattggccaatttctttcactttacctaatacaagctaataataacacatggaaaagaaaattatatgctgacttgtgtaacttaaagaacaataattttcttgaaccaagcatgtga : 3020
M82.3       : atattggccaatttctttcactttacctaatacaagctaataataacacatggaaaagaaaattatatgctgacttgtgtaacttaaagaacaataattttcttgaaccaagcatgtga : 3020
EA00371.3   : atattggccaatttctttcactttacctaatacaagctaataataacacatggaaaagaaaattatatgctgacttgtgtaacttaaagaacaataattttcttgaaccaagcatgtga : 3020
Fla.8924.3  : atattggccaatttctttcactttacctaatacaagctaataataacacatggaaaagaaaattatatgctgacttgtgtaacttaaagaacaataattttcttgaaccaagcatgtga : 3020
Floradade.  : atattggccaatttctttcactttacctaatacaagctaataataacacatggaaaagaaaattatatgctgacttgtgtaacttaaagaacaataattttcttgaaccaagcatgtga : 3020
LYC1410.3   : atattggccaatttctttcactttacctaatacaagctaataataacacatggaaaagaaaattatatgctgacttgtgtaacttaaagaacaataattttcttgaaccaagcatgtga : 3020
PI169588.3  : atattggccaatttctttcactttacctaatacaagctaataataacacatggaaaagaaaattatatgctgacttgtgtaacttaaagaacaataattttcttgaaccaagcatgtga : 3020
Brandywine  : atattggccaatttctttcactttacctaatacaagctaataataacacatggaaaagaaaattatatgctgacttgtgtaacttaaagaacaataattttcttgaaccaagcatgtga : 3020
EA00990.3   : atattggccaatttctttcactttacctaatacaagctaataataacacatggaaaagaaaattatatgctgacttgtgtaacttaaagaacaataattttcttgaaccaagcatgtga : 3020
PI303721.3  : atattggccaatttctttcactttacctaatacaagctaataataacacatggaaaagaaaattatatgctgacttgtgtaacttaaagaacaataattttcttgaaccaagcatgtga : 3020
BGV006775.  : atattggccaatttctttcactttacctaatacaagctaataataacacatggaaaagaaaattatatgctgacttgtgtaacttaaagaacaataattttcttgaaccaagcatgtga : 3020
PAS014479.  : atattggccaatttctttcactttacctaatacaagctaataataacacatggaaaagaaaattatatgctgacttgtgtaacttaaagaacaataattttcttgaaccaagcatgtga : 3020
              ATATTGGCCAATTTCTTTCACTTTACCTAATACAAGCTAATAATAACACATGGAAAAGAAAATTATATGCTGACTTGTGTAACTTAAAGAACAATAATTTTCTTGAACCAAGCATGTGA
```

Solyc04g051600

```
                      *        20         *        40         *        60         *        80         *       100         *       120         *
ITAG.4      : gcccaacgatgattgacttttttttaaactaatgccatgtatatcgaaaagggatagaaaattacatataaaataagttcaggagagtaataggacattagtataatataagtgtatctctaaaatttcgag :  131
BGV006865.  : ----------------------------------------------------------------------------------------------------------------------------------- :    -
BGV007931.  : gcccaacgatgattgacttttttttaaactaatgccatgtatatcgaaaagggatagaaaattacatataaaataagttcaggagagtaataggacattagtataatataagtgtatctctaaaatttcgag :  131
BGV007989.  : gcccaacgatgattgacttttttttaaactaatgccatgtatatcgaaaagggatagaaaattacatataaaataagttcaggagagtaataggacattagtataatataagtgtatctctaaaatttcgag :  131
M82.4       : ----------------------------------------------------------------------------------------------------------------------------------- :    -
EA00371.4   : gcccaacgatgattgacttttttttaaactaatgccatgtatatcgaaaagggatagaaaattacatataaaataagttcaggagagtaataggacattagtataatataagtgtatctctaaaatttcgag :  131
```

```
Fla.8924.4 : gcccaacgatgattgacttttttaaactaatgccatgtatatcgaaaagggatagaaaattacatataaaataagttcaggagagtaataggacattagtataatataagtgtatctctaaaatttcgag :  131
Floradade. : gcccaacgatgattgacttttttaaactaatgccatgtatatcgaaaagggatagaaaattacatataaaataagttcaggagagtaataggacattagtataatataagtgtatctctaaaatttcgag :  131
LYC1410.4  : gcccaacgatgattgacttttttaaactaatgccatgtatatcgaaaagggatagaaaattacatataaaataagttcaggagagtaataggacattagtataatataagtgtatctctaaaatttcgag :  131
PI169588.4 : gcccaacgatgattgacttttttaaactaatgccatgtatatcgaaaagggatagaaaattacatataaaataagttcaggagagtaataggacattagtataatataagtgtatctctaaaatttcgag :  131
Brandywine : gcccaacgatgattgacttttttaaactaatgccatgtatatcgaaaagggatagaaaattacatataaaataagttcaggagagtaataggacattagtataatataagtgtatctctaaaatttcgag :  131
EA00990.4  : gcccaacgatgattgacttttttaaactaatgccatgtatatcgaaaagggatagaaaattacatataaaataagttcaggagagtaataggacattagtataatataagtgtatctctaaaatttcgag :  131
PI1303721. : gcccaacgatgattgacttttttaaactaatgccatgtatatcgaaaagggatagaaaattacatataaaataagttcaggagagtaataggacattagtataatataagtgtatctctaaaatttcgag :  131
BGV006775. : ----------------------------------------------------------------------------------------------------------------------------------- :    -
PAS014479. : ----------------------------------------------------------------------------------------------------------------------------------- :    -

                   140         *       160         *       180         *       200         *       220         *       240         *       260
ITAG.4     : catagattaagaagatatttatacatttccctatttatacttgtaaaattatttagatagaactaattgattatattaattcagacatgtatatagacattagataattaataattatgtattttgccgc :  262
BGV006865. : ----------------------------------------------------------------------------------------------------------------------------------- :    -
BGV007931. : catagattaagaagatatttatacatttccctatttatacttgtaaaattatttagatagaactaattgattatattaattcagacatgtatatagacattagataattaataattatgtattttgccgc :  262
BGV007989. : catagattaagaagatatttatacatttccctatttatacttgtaaaattatttagatagaactaattgattatattaattcagacatgtatatagacattagataattaataattatgtattttgccgc :  262
M82.4      : ----------------------------------------------------------------------------------------------------------------------------------- :    -
EA00371.4  : catagattaagaagatatttatacatttccctatttatacttgtaaaattatttagatagaactaattgattatattaattcagacatgtatatagacattagataattaataattatgtattttgccgc :  262
Fla.8924.4 : catagattaagaagatatttatacatttccctatttatacttgtaaaattatttagatagaactaattgattatattaattcagacatgtatatagacattagataattaataattatgtattttgccgc :  262
Floradade. : catagattaagaagatatttatacatttccctatttatacttgtaaaattatttagatagaactaattgattatattaattcagacatgtatatagacattagataattaataattatgtattttgccgc :  262
LYC1410.4  : catagattaagaagatatttatacatttccctatttatacttgtaaaattatttagatagaactaattgattatattaattcagacatgtatatagacattagataattaataattatgtattttgccgc :  262
PI169588.4 : catagattaagaagatatttatacatttccctatttatacttgtaaaattatttagatagaactaattgattatattaattcagacatgtatatagacattagataattaataattatgtattttgccgc :  262
Brandywine : catagattaagaagatatttatacatttccctatttatacttgtaaaattatttagatagaactaattgattatattaattcagacatgtatatagacattagataattaataattatgtattttgccgc :  262
EA00990.4  : catagattaagaagatatttatacatttccctatttatacttgtaaaattatttagatagaactaattgattatattaattcagacatgtatatagacattagataattaataattatgtattttgccgc :  262
PI1303721. : catagattaagaagatatttatacatttccctatttatacttgtaaaattatttagatagaactaattgattatattaattcagacatgtatatagacattagataattaataattatgtattttgccgc :  262
BGV006775. : ----------------------------------------------------------------------------------------------------------------------------------- :    -
PAS014479. : ----------------------------------------------------------------------------------------------------------------------------------- :    -

                     *       280         *       300         *       320         *       340         *       360         *       380         *
ITAG.4     : ttaaaaattacttatgttactttgttacaataaccaaataaataatttaaatcttcaaaatcaacttaataataaaaaattaagcatttcatgtaaaatgggtttattggtgaggatcgatatctcattt :  393
BGV006865. : ----------------------------------------------------------------------------------------------------------------------------------- :    -
BGV007931. : ttaaaaattacttatgttactttgttacaataaccaaataaataatttaaatcttcaaaatcaacttaataataaaaaattaagcatttcatgtaaaatgggtttattggtgaggatcgatatctcattt :  393
BGV007989. : ttaaaaattacttatgttactttgttacaataaccaaataaataatttaaatcttcaaaatcaacttaataataaaaaattaagcatttcatgtaaaatgggtttattggtgaggatcgatatctcattt :  393
M82.4      : ----------------------------------------------------------------------------------------------------------------------------------- :    -
EA00371.4  : ttaaaaattacttatgttactttgttacaataaccaaataaataatttaaatcttcaaaatcaacttaataataaaaaattaagcatttcatgtaaaatgggtttattggtgaggatcgatatctcattt :  393
Fla.8924.4 : ttaaaaattacttatgttactttgttacaataaccaaataaataatttaaatcttcaaaatcaacttaataataaaaaattaagcatttcatgtaaaatgggtttattggtgaggatcgatatctcattt :  393
Floradade. : ttaaaaattacttatgttactttgttacaataaccaaataaataatttaaatcttcaaaatcaacttaataataaaaaattaagcatttcatgtaaaatgggtttattggtgaggatcgatatctcattt :  393
LYC1410.4  : ttaaaaattacttatgttactttgttacaataaccaaataaataatttaaatcttcaaaatcaacttaataataaaaaattaagcatttcatgtaaaatgggtttattggtgaggatcgatatctcattt :  393
PI169588.4 : ttaaaaattacttatgttactttgttacaataaccaaataaataatttaaatcttcaaaatcaacttaataataaaaaattaagcatttcatgtaaaatgggtttattggtgaggatcgatatctcattt :  393
Brandywine : ttaaaaattacttatgttactttgttacaataaccaaataaataatttaaatcttcaaaatcaacttaataataaaaaattaagcatttcatgtaaaatgggtttattggtgaggatcgatatctcattt :  393
EA00990.4  : ttaaaaattacttatgttactttgttacaataaccaaataaataatttaaatcttcaaaatcaacttaataataaaaaattaagcatttcatgtaaaatgggtttattggtgaggatcgatatctcattt :  393
PI1303721. : ttaaaaattacttatgttactttgttacaataaccaaataaataatttaaatcttcaaaatcaacttaataataaaaaattaagcatttcatgtaaaatgggtttattggtgaggatcgatatctcattt :  393
BGV006775. : ----------------------------------------------------------------------------------------------------------------------------------- :    -
PAS014479. : ----------------------------------------------------------------------------------------------------------------------------------- :    -

                 400         *       420         *       440         *       460         *       480         *       500         *       520
ITAG.4     : ttacttgttcaccggtaatcgacatattaccttgatatatcattttataacaaataaaaatgttatttttctaaccttaagttttaaaaaagatgaaaaagaaattgctcatggttcaacatcacactaac :  524
BGV006865. : ----------------------------------------------------------------------------------------------------------------------------------- :    -
BGV007931. : ttacttgttcaccagtaatcgacatattaccttgatatatcattttataacaaataaaaatgttatttttctaaccttaagttttaaaaaagatgaaaaagaaattgctcatggttcaacatcacactaac :  524
BGV007989. : ttacttgttcaccggtaatcgacatattaccttgatatatcattttataacaaataaaaatgttatttttctaaccttaagttttaaaaaagatgaaaaagaaattgctcatggttcaacatcacactaac :  524
M82.4      : ----------------------------------------------------------------------------------------------------------------------------------- :    -
EA00371.4  : ttacttgttcaccagtaatcgacatattaccttgatatatcattttataacaaataaaaatgttatttttctaaccttaagttttaaaaaagatgaaaaagaaattgctcatggttcaacatcacactaac :  524
Fla.8924.4 : ttacttgttcaccggtaatcgacatattaccttgatatatcattttataacaaataaaaatgttatttttctaaccttaagttttaaaaaagatgaaaaagaaattgctcatggttcaacatcacactaac :  524
Floradade. : ttacttgttcaccggtaatcgacatattaccttgatatatcattttataacaaataaaaatgttatttttctaaccttaagttttaaaaaagatgaaaaagaaattgctcatggttcaacatcacactaac :  524
LYC1410.4  : ttacttgttcaccagtaatcgacatattaccttgatatatcattttataacaaataaaaatgttatttttctaaccttaagttttaaaaaagatgaaaaagaaattgctcatggttcaacatcacactaac :  524
PI169588.4 : ttacttgttcaccggtaatcgacatattaccttgatatatcattttataacaaataaaaatgttatttttctaaccttaagttttaaaaaagatgaaaaagaaattgctcatggttcaacatcacactaac :  524
Brandywine : ttacttgttcaccggtaatcgacatattaccttgatatatcattttataacaaataaaaatgttatttttctaaccttaagttttaaaaaagatgaaaaagaaattgctcatggttcaacatcacactaac :  524
EA00990.4  : ttacttgttcaccggtaatcgacatattaccttgatatatcattttataacaaataaaaatgttatttttctaaccttaagttttaaaaaagatgaaaaagaaattgctcatggttcaacatcacactaac :  524
PI1303721. : ttacttgttcaccggtaatcgacatattaccttgatatatcattttataacaaataaaaatgttatttttctaaccttaagttttaaaaaagatgaaaaagaaattgctcatggttcaacatcacactaac :  524
BGV006775. : ----------------------------------------------------------------------------------------------------------------------------------- :    -
PAS014479. : ----------------------------------------------------------------------------------------------------------------------------------- :    -

                   *       540         *       560         *       580         *       600         *       620         *       640         *
ITAG.4     : aatatatttggatcattgttatctattatattatattataatgtttgttttaattgttacttaaaatgtattgtattgtattattaaatttcgttgttacgtatcaatgaaaatctctattttatggaata :  655
BGV006865. : ----------------------------------------------------------------------------------------------------------------------------------- :    -
BGV007931. : aatatatttggatcattgttatctattatattatattataatgtttgttttaattgttacttaaaatgtattgtattgtattattaaatttcgttgttacgtatcaatgaaaatctctattttatggaata :  655
BGV007989. : aatatatttggatcattgttatctattatattatattataatgtttgttttaattgttacttaaaatgtattgtattgtattattaaatttcgttgttacgtatcaatgaaaatctctattttatggaata :  655
M82.4      : ----------------------------------------------------------------------------------------------------------------------------------- :    -
EA00371.4  : aatatatttggatcattgttatctattatattatattataatgtttgttttaattgttacttaaaatgtattgtattgtattattaaatttcgttgttacgtatcaatgaaaatctctattttatggaata :  655
Fla.8924.4 : aatatatttggatcattgttatctattatattatattataatgtttgttttaattgttacttaaaatgtattgtattgtattattaaatttcgttgttacgtatcaatgaaaatctctattttatggaata :  655
Floradade. : aatatatttggatcattgttatctattatattatattataatgtttgttttaattgttacttaaaatgtattgtattgtattattaaatttcgttgttacgtatcaatgaaaatctctattttatggaata :  655
LYC1410.4  : aatatatttggatcattgttatctattatattatattataatgtttgttttaattgttacttaaaatgtattgtattgtattattaaatttcgttgttacgtatcaatgaaaatctctattttatggaata :  655
PI169588.4 : aatatatttggatcattgttatctattatattatattataatgtttgttttaattgttacttaaaatgtattgtattgtattattaaatttcgttgttacgtatcaatgaaaatctctattttatggaata :  655
Brandywine : aatatatttggatcattgttatctattatattatattataatgtttgttttaattgttacttaaaatgtattgtattgtattattaaatttcgttgttacgtatcaatgaaaatctctattttatggaata :  655
EA00990.4  : aatatatttggatcattgttatctattatattatattataatgtttgttttaattgttacttaaaatgtattgtattgtattattaaatttcgttgttacgtatcaatgaaaatctctattttatggaata :  655
PI1303721. : aatatatttggatcattgttatctattatattatattataatgtttgttttaattgttacttaaaatgtattgtattgtattattaaatttcgttgttacgtatcaatgaaaatctctattttatggaata :  655
BGV006775. : ----------------------------------------------------------------------------------------------------------------------------------- :    -
PAS014479. : ----------------------------------------------------------------------------------------------------------------------------------- :    -

               660         *       680         *       700         *       720         *       740         *       760         *       780
ITAG.4     : acaaatttagtgtattcccattgttacttaatttcttttttccaattatatttttacctaatattttaaaaatactattttaccctttatcttaattatttaaacatagtcaaaccttttatcctaaaatac :  786
BGV006865. : ---------------------------------------tccaattgtatttttacctaatattttaaaaatactattttaccctttatcttaattatttaaacatagtcaaaccttttatcctaaaatac :   92
BGV007931. : acaaatttagtgtattcccattgttacttaatttcttttttccaattatatttttacctaatattttaaaaatactattttaccctttatcttaattatttaaacatagtcaaaccttttatcctaaaatac :  786
BGV007989. : acaaatttagtgtattcccattgttacttaatttcttttttccaattatatttttacctaatattttaaaaatactattttaccctttatcttaattatttaaacatagtcaaaccttttatcctaaaatac :  786
M82.4      : ---------------------------------------tccaattatatttttacctaatattttaaaaatactattttaccctttatcttaattatttaaacatagtcaaaccttttatcctaaaatac :   92
EA00371.4  : acaaatttagtgtattcccattgttacttaatttcttttttccaattatatttttacctaatattttaaaaatactattttaccctttatcttaattatttaaacatagtcaaaccttttatcctaaaatac :  786
Fla.8924.4 : acaaatttagtgtattcccattgttacttaatttcttttttccaattatatttttacctaatattttaaaaatactattttaccctttatcttaattatttaaacatagtcaaaccttttatcctaaaatac :  786
Floradade. : acaaatttagtgtattcccattgttacttaatttcttttttccaattatatttttacctaatattttaaaaatactattttaccctttatcttaattatttaaacatagtcaaaccttttatcctaaaatac :  786
LYC1410.4  : acaaatttagtgtattcccattgttacttaatttcttttttccaattatatttttacctaatattttaaaaatactattttaccctttatcttaattatttaaacatagtcaaaccttttatcctaaaatac :  786
PI169588.4 : acaaatttagtgtattcccattgttacttaatttcttttttccaattatatttttacctaatattttaaaaatactattttaccctttatcttaattatttaaacatagtcaaaccttttatcctaaaatac :  786
Brandywine : acaaatttagtgtattcccattgttacttaatttcttttttccaattatatttttacctaatattttaaaaatactattttaccctttatcttaattatttaaacatagtcaaaccttttatcctaaaatac :  786
EA00990.4  : acaaatttagtgtattcccattgttacttaatttcttttttccaattatatttttacctaatattttaaaaatactattttaccctttatcttaattatttaaacatagtcaaaccttttatcctaaaatac :  786
PI1303721. : acaaatttagtgtattcccattgttacttaatttcttttttccaattatatttttacctaatattttaaaaatactattttaccctttatcttaattatttaaacatagtcaaaccttttatcctaaaatac :  786
BGV006775. : ---------------------------------------tccaattgtatttttacctaatattttaaaaatactattttaccctttatcttaattatttaaacatagtcaaaccttttatcctaaaatac :   92
PAS014479. : ---------------------------------------tccaattatatttttacctaatattttaaaaatactattttaccctttatcttaattatttaaacatagtcaaaccttttatcctaaaatac :   92
                                                      TCCAATTaTATTTTTACCTAATATTTTAAAAATACTATTTTACCCTTTATCTTAATTATTTAAACATAGTCAAACCTTTTATCCTAAAATAC

                 *       800         *       820         *       840         *       860         *       880         *       900         *
ITAG.4     : ttaagaataattaagtaaatttataaattacaatacaatacgataaataattaaaatgttactaaataataacaaacaatataatctagctaaatattgtatctatcatacaatacagtataatagagtac :  917
BGV006865. : ttaagaataattaagtaaatttataaattacaatacaatacgataaataattaaaatgttactaaataataacaaacaatataatctagctaaatattatatctatcatacgatacagtataatagagtac :  223
BGV007931. : ttaagaataattaagtaaatttataaattacaatacaatacgataaataattaaaatgttactaaataataacaaacaatataatctagctaaatattgtatctatcatacaatacagtataatagagtac :  917
BGV007989. : ttaagaataattaagtaaatttataaattacaatacaatacgataaataattaaaatgttactaaataataacaaacaatataatctagctaaatattgtatctatcatacaatacagtataatagagtac :  917
M82.4      : ttaagaataattaagtaaatttataaattacaatacaatacgataaataattaaaatgttactaaataataacaaacaatataatctagctaaatattgtatctatcatacaatacagtataatagagtac :  223
EA00371.4  : ttaagaataattaagtaaatttataaattacaatacaatacgataaataattaaaatgttactaaataataacaaacaatataatctagctaaatattgtatctatcatacaatacagtataatagagtac :  917
Fla.8924.4 : ttaagaataattaagtaaatttataaattacaatacaatacgataaataattaaaatgttactaaataataacaaacaatataatctagctaaatattgtatctatcatacaatacagtataatagagtac :  917
Floradade. : ttaagaataattaagtaaatttataaattacaatacaatacgataaataattaaaatgttactaaataataacaaacaatataatctagctaaatattgtatctatcatacaatacagtataatagagtac :  917
LYC1410.4  : ttaagaataattaagtaaatttataaattacaatacaatacgataaataattaaaatgttactaaataataacaaacaatataatctagctaaatattgtatctatcatacaatacagtataatagagtac :  917
PI169588.4 : ttaagaataattaagtaaatttataaattacaatacaatacgataaataattaaaatgttactaaataataacaaacaatataatctagctaaatattgtatctatcatacaatacagtataatagagtac :  917
Brandywine : ttaagaataattaagtaaatttataaattacaatacaatacgataaataattaaaatgttactaaataataacaaacaatataatctagctaaatattgtatctatcatacaatacagtataatagagtac :  917
EA00990.4  : ttaagaataattaagtaaatttataaattacaatacaatacgataaataattaaaatgttactaaataataacaaacaatataatctagctaaatattgtatctatcatacaatacagtataatagagtac :  917
PI1303721. : ttaagaataattaagtaaatttataaattacaatacaatacgataaataattaaaatgttactaaataataacaaacaatataatctagctaaatattgtatctatcatacaatacagtataatagagtac :  917
BGV006775. : ttaagaataattaagtaaatttataaattacaatacaatacgataaataattaaaatgttactaaataataacaaacaatataatctagctaaatattatatctatcatacgatacagtataatagagtac :  223
PAS014479. : ttaagaataattaagtaaatttataaattacaatacaatacgataaataattaaaatgttactaaataataacaaataatataatctagctaaatattgtatctatcatacaatacagtataatagagtac :  223
             TTAAGAATAATTAAGTAAATTTATAAATTACAATACAATACGATAAATAATTAAAATGTTACTAAATAATAACAAAcAATATAATCTAGCTAAATATTgTATCTATCATACaATACAGTATAATAGAGTAC

             920         *       940         *       960         *       980         *      1000         *      1020         *      1040
ITAG.4     : attataatataatatattataaaacaatgcgtaacaatgatctaaataaagcgtaaaagccaaaagaataaggaacaaaagatgccttctacagcttccctatgtaccccatactaaattgtgaaataata : 1048
BGV006865. : attataatataatatattatgaaacaatgcgcaacaatgatctaaataaagcgtaaaagccaaaagaataaggaacaaaagatgccttctacaacttccctatgtaccccatattaaattgtgaaataata :  354
BGV007931. : attataatataatatattataaaacaatgcgtaacaatgatctaaataaagcgtaaaagccaaaagaataaggaacaaaagatgccttctacagcttccctatgtaccccatattaaattgtgaaataata : 1048
BGV007989. : attataatataatatattataaaacaatgcgtaacaatgatctaaataaagcgtaaaagccaaaagaataaggaacaaaagatgccttctacagcttccctatgtaccccatactaaattgtgaaataata : 1048
M82.4      : attataatataatatattatgaaacaatgcgcaacaatgatctaaataaagcgtaaaagccaaaagaataaggaacaaaagatgccttctacaacttccctatgtaccccatattaaattgtgaaataata :  354
EA00371.4  : attataatataatatattataaaacaatgcgtaacaatgatctaaataaagcgtaaaagccaaaagaataaggaacaaaagatgccttctacagcttccctatgtaccccatactaaattgtgaaataata : 1048
Fla.8924.4 : attataatataatatattataaaacaatgcgtaacaatgatctaaataaagcgtaaaagccaaaagaataaggaacaaaagatgccttctacagcttccctatgtaccccatactaaattgtgaaataata : 1048
Floradade. : attataatataatatattataaaacaatgcgtaacaatgatctaaataaagcgtaaaagccaaaagaataaggaacaaaagatgccttctacagcttccctatgtaccccatactaaattgtgaaataata : 1048
LYC1410.4  : attataatataatatattataaaacaatgcgtaacaatgatctaaataaagcgtaaaagccaaaagaataaggaacaaaagatgccttctacagcttccctatgtaccccatactaaattgtgaaataata : 1048
PI169588.4 : attataatataatatattataaaacaatgcgtaacaatgatctaaataaagcgtaaaagccaaaagaataaggaacaaaagatgccttctacagcttccctatgtaccccatactaaattgtgaaataata : 1048
Brandywine : attataatataatatattataaaacaatgcgtaacaatgatctaaataaagcgtaaaagccaaaagaataaggaacaaaagatgccttctacagcttccctatgtaccccatactaaattgtgaaataata : 1048
EA00990.4  : attataatataatatattataaaacaatgcgtaacaatgatctaaataaagcgtaaaagccaaaagaataaggaacaaaagatgccttctacagcttccctatgtaccccatactaaattgtgaaataata : 1048
```

```
PI1303721. : attataatataatatattataaaacaatgcgtaacaatgatctaaataaagcgtaaaagccaaaagaataaggaacaaaagatgccttctacagcttccctatgtaccccatactaaattgtgaaataata : 1048
BGV006775. : attataatataatatattatgaaacaatgcgcaacaatgatctaaataaagcgtaaaagccaaaagaataaggaacaaaagatgccttctacaacttccctatgtaccccatattaaattgtgaaataata :  354
PAS014479. : attataatataatatattatgaaacaatgcgcaacaatgatctaaataaagcgtaaaagccaaaagaataaggaacaaaagatgccttctacaacttccctatgtaccccatattaaattgtgaaataata :  354
             ATTATAATATAATATATTAT AAACAATGCG AACAATGATCTAAATAAAGCGTAAAAGCCAAAAGAATAAGGAACAAAAGATGCCTTCTACA CTTCCCTATGTACCCCATA TAAATTGTGAAATAATA

                 *      1060         *      1080         *      1100         *      1120         *      1140         *      1160         *      118
ITAG.4     : tctttcatcgctttataatttattacatatacaaatataattgcttaataatatgagaaatgatgaaaatagtccttatatttgagggtagatttaaatgatctttaaatatataggcaacaattttgtt : 1179
BGV006865. : tctttcatcgctttataatttattacatatacaaatataattgcttaataatatgagaaatgatgaaaatagtccttatatttgagggtagatttaaatgatctttaaatatatacgcaacaattttgtt :  485
BGV007931. : tctttcatcgctttataatttattacatatacaaatataattgcttaataatatgagaaatgatgaaaatagtccttatatttgagggtagatttaaatgatctttaaatatataggcaacaattttgtt : 1179
BGV007989. : tctttcatcgctttataatttattacatatacaaatataattgcttaataatatgagaaatgatgaaaatagtccttatatttgagggtagatttaaatgatctttaaatatataggcaacaattttgtt : 1179
M82.4      : tctttcatcgctttataatttattacatatacaaatataattgcttaataatatgagaaatgatgaaaatagtccttatatttgagggtagatttaaatgatctttaaatatatacgcaacaattttgtt :  485
EA00371.4  : tctttcatcgctttataatttattacatatacaaatataattgcttaataatatgagaaatgatgaaaatagtccttatatttgagggtagatttaaatgatctttaaatatataggcaacaattttgtt : 1179
Fla.8924.4 : tctttcatcgctttataatttattacatatacaaatataattgcttaataatatgagaaatgatgaaaatagtccttatatttgagggtagatttaaatgatctttaaatatataggcaacaattttgtt : 1179
Floradade. : tctttcatcgctttataatttattacatatacaaatataattgcttaataatatgagaaatgatgaaaatagtccttatatttgagggtagatttaaatgatctttaaatatataggcaacaattttgtt : 1179
LYC1410.4  : tctttcatcgctttataatttattacatatacaaatataattgcttaataatatgagaaatgatgaaaatagtccttatatttgagggtagatttaaatgatctttaaatatataggcaacaattttgtt : 1179
PI169588.4 : tctttcatcgctttataatttattacatatacaaatataattgcttaataatatgagaaatgatgaaaatagtccttatatttgagggtagatttaaatgatctttaaatatataggcaacaattttgtt : 1179
Brandywine : tctttcatcgctttataatttattacatatacaaatataattgcttaataatatgagaaatgatgaaaatagtccttatatttgagggtagatttaaatgatctttaaatatataggcaacaattttgtt : 1179
EA00990.4  : tctttcatcgctttataatttattacatatacaaatataattgcttaataatatgagaaatgatgaaaatagtccttatatttgagggtagatttaaatgatctttaaatatataggcaacaattttgtt : 1179
PI1303721. : tctttcatcgctttataatttattacatatacaaatataattgcttaataatatgagaaatgatgaaaatagtccttatatttgagggtagatttaaatgatctttaaatatataggcaacaattttgtt : 1179
BGV006775. : tctttcatcgctttataatttattacatatacaaatataattgcttaataatatgagaaatgatgaaaatagtccttatatttgagggtagatttaaatgatctttaaatatatacgcaacaattttgtt :  485
PAS014479. : tctttcatcgctttataatttattacatatacaaatataattgcttaataatatgagaaatgatgaaaatagtccttatatttgagggtagatttaaatgatctttaaatatatacgcaacaattttgtt :  485
             TCTTTCATCGCTTTATAATTTATTACATATACAAATATAATTGCTTAATAATATGAGAAATGATGAAAATAGTCCTTATATTTGAGGGTAGATTTAAATGATCTTTAAATATATA GCAACAATTTTGTT

             0         *      1200         *      1220         *      1240         *      1260         *      1280         *      1300         *
ITAG.4     : ttaagtatgttaaaaattaatttcaataaatattaatcaatcgattagttaaatttgatgaaattcataaaaaaaaatggaaattaacaaaaactcttggtagaggaataatttgtatttttttctatttt : 1310
BGV006865. : ttaagtatgttaaaaattaatttcaataaatattaatcaatcgattagttaaatttgatgaaattcat-aaaaaaaatggaaattaacaaaaactcttggtagaggaataatttgtatttttttctatttt :  615
BGV007931. : ttaagtatgttaaaaattaatttcaataaatattaatcaatcgattagttaaatttgatgaaattcataaaaaaaaatggaaattaacaaaaactcttggtagaggaataatttgtatttttttctatttt : 1310
BGV007989. : ttaagtatgttaaaaattaatttcaataaatattaatcaatcgattagttaaatttgatgaaattcataaaaaaaaatggaaattaacaaaaactcttggtagaggaataatttgtatttttttctatttt : 1310
M82.4      : ttaagtatgttaaaaattaatttcaataaatattaatcaatcgattagttaaatttgatgaaattcataaaaaaaaatggaaattaacaaaaactcttggtagaggaataatttgtatttttttctatttt :  616
EA00371.4  : ttaagtatgttaaaaattaatttcaataaatattaatcaatcgattagttaaatttgatgaaattcataaaaaaaaatggaaattaacaaaaactcttggtagaggaataatttgtatttttttctatttt : 1310
Fla.8924.4 : ttaagtatgttaaaaattaatttcaataaatattaatcaatcgattagttaaatttgatgaaattcataaaaaaaaatggaaattaacaaaaactcttggtagaggaataatttgtatttttttctatttt : 1310
Floradade. : ttaagtatgttaaaaattaatttcaataaatattaatcaatcgattagttaaatttgatgaaattcataaaaaaaaatggaaattaacaaaaactcttggtagaggaataatttgtatttttttctatttt : 1310
LYC1410.4  : ttaagtatgttaaaaattaatttcaataaatattaatcaatcgattagttaaatttgatgaaattcataaaaaaaaatggaaattaacaaaaactcttggtagaggaataatttgtatttttttctatttt : 1310
PI169588.4 : ttaagtatgttaaaaattaatttcaataaatattaatcaatcgattagttaaatttgatgaaattcataaaaaaaaatggaaattaacaaaaactcttggtagaggaataatttgtatttttttctatttt : 1310
Brandywine : ttaagtatgttaaaaattaatttcaataaatattaatcaatcgattagttaaatttgatgaaattcataaaaaaaaatggaaattaacaaaaactcttggtagaggaataatttgtatttttttctatttt : 1310
EA00990.4  : ttaagtatgttaaaaattaatttcaataaatattaatcaatcgattagttaaatttgatgaaattcataaaaaaaaatggaaattaacaaaaactcttggtagaggaataatttgtatttttttctatttt : 1310
PI1303721. : ttaagtatgttaaaaattaatttcaataaatattaatcaatcgattagttaaatttgatgaaattcataaaaaaaaatggaaattaacaaaaactcttggtagaggaataatttgtatttttttctatttt : 1310
BGV006775. : ttaagtatgttaaaaattaatttcaataaatattaatcaatcgattagttaaatttgatgaaattcat-aaaaaaaatggaaattaacaaaaactcttggtagaggaataatttgtatttttttctatttt :  615
PAS014479. : ttaagtatgttaaaaattaatttcaataaatattaatcaatcgattagttaaatttgatgaaattcataaaaaaaaatggaaattaacaaaaactcttggtagaggaataatttgtatttttttctatttt :  616
             TTAAGTATGTTAAAAATTAATTTCAATAAATATTAATCAATCGATTAGTTAAATTTGATGAAATTCATaAAAAAAAATGGAAATTAACAAAAACTCTTGGTAGAGGAATAATTTGTATTTTTTTCTATTTT

                  1320         *      1340         *      1360         *      1380         *      1400         *      1420         *      1440
ITAG.4     : ttgtttattttttagaatttggtatagtttttttatttatattttttcagtatctactgttatatttgtaatgtatattttagaatttgataaaacttttcaatttttttatagattaatctttttttcatacc : 1441
BGV006865. : ttgtttattttttagaatttggtatagtttttttatttatattttttcagtatctactgttatatttgtaatgtatattttagaatttgataaaacttttcaatttttttatagattaatctttttttcatacc :  746
BGV007931. : ttgtttattttttagaatttggtatagtttttttatttatattttttcagtatctactgttatatttgtaatgtatattttagaatttgataaaacttttcaatttttttatagattaatctttttttcatacc : 1441
BGV007989. : ttgtttattttttagaatttggtatagtttttttatttatattttttcagtatctactgttatatttgtaatgtatattttagaatttgataaaacttttcaatttttttatagattaatctttttttcatacc : 1441
M82.4      : ttgttta-ttttggaatttggtatagtttttttatttatattttttcagtatctactgttatatttgtaatgtatattttagaatttgataaaacttttcaatttttttatagattaatctttttttcatacc :  746
EA00371.4  : ttgtttattttttagaatttggtatagtttttttatttatattttttcagtatctactgttatatttgtaatgtatattttagaatttgataaaacttttcaatttttttatagattaatctttttttcatacc : 1441
Fla.8924.4 : ttgtttattttttagaatttggtatagtttttttatttatattttttcagtatctactgttatatttgtaatgtatattttagaatttgataaaacttttcaatttttttatagattaatctttttttcatacc : 1441
Floradade. : ttgtttattttttagaatttggtatagtttttttatttatattttttcagtatctactgttatatttgtaatgtatattttagaatttgataaaacttttcaatttttttatagattaatctttttttcatacc : 1441
LYC1410.4  : ttgtttattttttagaatttggtatagtttttttatttatattttttcagtatctactgttatatttgtaatgtatattttagaatttgataaaacttttcaatttttttatagattaatctttttttcatacc : 1441
PI169588.4 : ttgtttattttttagaatttggtatagtttttttatttatattttttcagtatctactgttatatttgtaatgtatattttagaatttgataaaacttttcaatttttttatagattaatctttttttcatacc : 1441
Brandywine : ttgtttattttttagaatttggtatagtttttttatttatattttttcagtatctactgttatatttgtaatgtatattttagaatttgataaaacttttcaatttttttatagattaatctttttttcatacc : 1441
EA00990.4  : ttgtttattttttagaatttggtatagtttttttatttatattttttcagtatctactgttatatttgtaatgtatattttagaatttgataaaacttttcaatttttttatagattaatctttttttcatacc : 1441
PI1303721. : ttgtttattttttagaatttggtatagtttttttatttatattttttcagtatctactgttatatttgtaatgtatattttagaatttgataaaacttttcaatttttttatagattaatctttttttcatacc : 1441
BGV006775. : ttgtttattttttagaatttggtatagtttttttatttatattttttcagtatctactgttatatttgtaatgtatattttagaatttgataaaacttttcaatttttttatagattaatctttttttcatacc :  746
PAS014479. : ttgttta-ttttggaatttggtatagtttttttatttatattttttcagtatctactgttatatttgtaatgtatattttagaatttgataaaacttttcaatttttttatagattaatctttttttcatacc :  746
             TTGTTTAtTTTTaGAATTTGGTATAGTTTTTTTATTTATATTTTTTCAGTATCTACTGTTATATTTGTAATGTATATTTTAGAATTTGATAAAACTTTTCAATTTTTTTATAGATTAATCTTTTTTTCATACC

                   *      1460         *      1480         *      1500         *      1520         *      1540         *      1560         *
ITAG.4     : tagattattaaatactatgacaaattttaaacaaataaaaaggaccaaaatttatcaca------------------------------------------------------------------------ : 1500
BGV006865. : tagattattaaatactatgacaaattttaaacaaataaaaaggaccaaaatttatcacatattacttaaaataaaaaaggactatttcaaattttaccctttaaaacataatggaaacattttgtcaattt :  877
BGV007931. : tagattattaaatactatgacaaattttaaacaaataaaaaggaccaaaatttatcaca------------------------------------------------------------------------ : 1500
BGV007989. : tagattattaaatactatgacaaattttaaacaaataaaaaggaccaaaatttatcaca------------------------------------------------------------------------ : 1500
M82.4      : tagattattaaatactatgacaaattttaaacaaataaaaaggaccaaaatttatcacatattacttaaaataaaaaaggactatttcaaattttaccctttaaaacataatggaaacattttgtcaattt :  877
EA00371.4  : tagattattaaatactatgacaaattttaaacaaataaaaaggaccaaaatttatcaca------------------------------------------------------------------------ : 1500
Fla.8924.4 : tagattattaaatactatgacaaattttaaacaaataaaaaggaccaaaatttatcaca------------------------------------------------------------------------ : 1500
Floradade. : tagattattaaatactatgacaaattttaaacaaataaaaaggaccaaaatttatcaca------------------------------------------------------------------------ : 1500
LYC1410.4  : tagattattaaatactatgacaaattttaaacaaataaaaaggaccaaaatttatcaca------------------------------------------------------------------------ : 1500
PI169588.4 : tagattattaaatactatgacaaattttaaacaaataaaaaggaccaaaatttatcaca------------------------------------------------------------------------ : 1500
Brandywine : tagattattaaatactatgacaaattttaaacaaataaaaaggaccaaaatttatcaca------------------------------------------------------------------------ : 1500
EA00990.4  : tagattattaaatactatgacaaattttaaacaaataaaaaggaccaaaatttatcaca------------------------------------------------------------------------ : 1500
PI1303721. : tagattattaaatactatgacaaattttaaacaaataaaaaggaccaaaatttatcaca------------------------------------------------------------------------ : 1500
BGV006775. : tagattattaaatactatgacaaattttaaacaaataaaaaggaccaaaatttatcacatattacttaaaataaaaaaggactatttcaaattttaccctttaaaacataatggaaacattttgtcaattt :  877
PAS014479. : tagattattaaatactatgacaaattttaaacaaataaaaaggaccaaaatttatcacatattacctaaaataaaaaaggactatttcaaattttaccctttaaaacataatggaaacattttgtcaattt :  877
             TAGATTATTAAATACTATGACAAATTTTAAACAAATAAAAAGGACCAAAATTTATCACA

                 1580         *      1600         *      1620         *      1640         *      1660         *      1680         *      1700
ITAG.4     : ----------------------------------------------------------------------------------------------------------------------------------- :    -
BGV006865. : gtagaaataaataataatatcaaagaattcttctaaccaaaaagacaaaaaaataaatcaagtgaatatataattactgattaattacatacataaaatatatttctgagtttgtgcataagccaccattt : 1008
BGV007931. : ----------------------------------------------------------------------------------------------------------------------------------- :    -
BGV007989. : ----------------------------------------------------------------------------------------------------------------------------------- :    -
M82.4      : gtagaaataaataataatatcaaagaattcttctaaccaaaaagacaaaaaaataaatcaagtgaatatataattactgattaattacatacataaaatatatttctgagtttgtgcataagccaccattt : 1008
EA00371.4  : ----------------------------------------------------------------------------------------------------------------------------------- :    -
Fla.8924.4 : ----------------------------------------------------------------------------------------------------------------------------------- :    -
Floradade. : ----------------------------------------------------------------------------------------------------------------------------------- :    -
LYC1410.4  : ----------------------------------------------------------------------------------------------------------------------------------- :    -
PI169588.4 : ----------------------------------------------------------------------------------------------------------------------------------- :    -
Brandywine : ----------------------------------------------------------------------------------------------------------------------------------- :    -
EA00990.4  : ----------------------------------------------------------------------------------------------------------------------------------- :    -
PI1303721. : ----------------------------------------------------------------------------------------------------------------------------------- :    -
BGV006775. : gtagaaataaataataatatcaaagaattcttctaaccaaaaagacaaaaaaataaatcaagtgaatatataattactgattaattacatacataaaatatatttctgagtttgtgcataagccaccattt : 1008
PAS014479. : gtagaaataaataataatatcaaagaattcttctaaccaaaaagacaaaaaaataaatcaagtgaatatataattactgattaattacatacataaaatatatttctgagtttgtgcataagccaccattt : 1008

                   *      1720         *      1740         *      1760         *      1780         *      1800         *      1820         *
ITAG.4     : ----------------------------------------------------------------------------------------------------------------------------------- :    -
BGV006865. : tttttttctttggttcgaatcgtgagcaaaagaaaggggaagaagactatgagaagcagagctttcagaaaacacagtttctgctaacaatcgatcaattaaaccctatccttttcaggttttcttctaac : 1139
BGV007931. : ----------------------------------------------------------------------------------------------------------------------------------- :    -
BGV007989. : ----------------------------------------------------------------------------------------------------------------------------------- :    -
M82.4      : tttttttctttggttcgaatcgtgagcaaaagaaaggggaagaagactatgagaagcagagctttcagaaaacacagtttctgctaacaatcgatcaattaaaccctatccttttcaggttttcttctaac : 1139
EA00371.4  : ----------------------------------------------------------------------------------------------------------------------------------- :    -
Fla.8924.4 : ----------------------------------------------------------------------------------------------------------------------------------- :    -
Floradade. : ----------------------------------------------------------------------------------------------------------------------------------- :    -
LYC1410.4  : ----------------------------------------------------------------------------------------------------------------------------------- :    -
PI169588.4 : ----------------------------------------------------------------------------------------------------------------------------------- :    -
Brandywine : ----------------------------------------------------------------------------------------------------------------------------------- :    -
EA00990.4  : ----------------------------------------------------------------------------------------------------------------------------------- :    -
PI1303721. : ----------------------------------------------------------------------------------------------------------------------------------- :    -
BGV006775. : tttttttctttggttcgaatcgtgagcaaaagaaaggggaagaagactatgagaagcagagctttcagaaaacacagtttctgctaacaatcgatcaattaaaccctatccttttcaggttttcttcta-- : 1137
PAS014479. : tttttttctttggttcgaatcgtgagcaaaagaaaggggaagaagactatgagaagcagagctttcagaaaacacagtttctgctaacaatcgatcaattaaaccctatccttttcaggttttcttctaac : 1139

               1840         *      1860         *      1880         *      1900         *      1920         *      1940         *      1960
ITAG.4     : ----------------------------------------------------------------------------------------------------------------------------------- :    -
BGV006865. : tgttcccccagttatgagttgaaagtttgataaaactgctaaggttttaatcgttgattatttttgtttctgttttatgtttgtttttttgcaagaatctttgtgaattaagaccccttttgaaatctgg : 1270
BGV007931. : ----------------------------------------------------------------------------------------------------------------------------------- :    -
BGV007989. : ----------------------------------------------------------------------------------------------------------------------------------- :    -
M82.4      : t--gttccccagttatgagttgaaagtttgataaaactgctaaggttttaatcgttgattatttttgtttctgttttatgtttgtttttttgcaagaatctttgtgaattaagaccccttttgaaatctgg : 1268
EA00371.4  : ----------------------------------------------------------------------------------------------------------------------------------- :    -
Fla.8924.4 : ----------------------------------------------------------------------------------------------------------------------------------- :    -
Floradade. : ----------------------------------------------------------------------------------------------------------------------------------- :    -
LYC1410.4  : ----------------------------------------------------------------------------------------------------------------------------------- :    -
PI169588.4 : ----------------------------------------------------------------------------------------------------------------------------------- :    -
Brandywine : ----------------------------------------------------------------------------------------------------------------------------------- :    -
EA00990.4  : ----------------------------------------------------------------------------------------------------------------------------------- :    -
PI1303721. : ----------------------------------------------------------------------------------------------------------------------------------- :    -
BGV006775. : ---tttccccagttatgagttgaaagtttgataaaactgctaaggttttaatcgttgattatttttgtttctgttttatgtttgtttttttgcaagaatctttgtgaattaagaccccttttgaaatctgg : 1265
PAS014479. : t--gttccccagttatgagttgaaagtttgataaaactgctaaggttttaatcgttgattatttttgtttctgttttatgtttgtttttttgcaagaatctttgtgaattaagaccccttttgtaatctgg : 1268

                 *      1980         *      2000         *      2020         *      2040         *      2060         *      2080         *
```

```
ITAG.4      : ----------------------------------------------------------------------------------------------------------------------------------- :    -
BGV006865.  : tatctgtgtcaattttagtttttaatttggttaaaaaagtggttttttttctgcaaatcttatagatgactgaattggattcatttttgatgagtttgaaactaattttcgcattagaatatcaagaaca : 1401
BGV007931.  : ----------------------------------------------------------------------------------------------------------------------------------- :    -
BGV007989.  : ----------------------------------------------------------------------------------------------------------------------------------- :    -
M82.4       : tatctgtgtcaattttagtttttaatttggttaaaaaagtggttttttttctgcaaatcttatagatgactgaattggattcatttttgatgagtttgaaactaattttcgcattagaatatcaagaaca : 1399
EA00371.4   : ----------------------------------------------------------------------------------------------------------------------------------- :    -
Fla.8924.4  : ----------------------------------------------------------------------------------------------------------------------------------- :    -
Floradade.  : ----------------------------------------------------------------------------------------------------------------------------------- :    -
LYC1410.4   : ----------------------------------------------------------------------------------------------------------------------------------- :    -
PI169588.4  : ----------------------------------------------------------------------------------------------------------------------------------- :    -
Brandywine  : ----------------------------------------------------------------------------------------------------------------------------------- :    -
EA00990.4   : ----------------------------------------------------------------------------------------------------------------------------------- :    -
PI1303721.  : ----------------------------------------------------------------------------------------------------------------------------------- :    -
BGV006775.  : tatctgtgtcaattttagtttttaatttggttaaaaaagtggttttttttctgcaaatcttatagatgactgaattggattcatttttgatgagtttgaaactaattttcgcattagaatatcaagaaca : 1396
PAS014479.  : tatctgtgtcaattttagtttttaatttggttaaaaaagtggttttttttctgcaaatcttatagatgactgaattggattcatttttgatgagtttgaaactaattttcgcattagaatatcaagaaca : 1399

              2100         *       2120         *       2140         *       2160         *       2180         *       2200         *       2220
ITAG.4      : --------------------------------------------------------------------------------------------------------nnnnnnnnnnnnnnnnnnnnaacaatt : 1527
BGV006865.  : gtgtgaatatgatgtggatttttgggctattgagtttacaaagatggtttagactggaggattggaaattttggaactggcatgctagtttgggtgtgt-----nnnnnnnnnnnnnnnnnnnnaacaatt : 1527
BGV007931.  : --------------------------------------------------------------------------------------------------------nnnnnnnnnnnnnnnnnnnnaacaatt : 1527
BGV007989.  : --------------------------------------------------------------------------------------------------------nnnnnnnnnnnnnnnnnnnnaacaatt : 1527
M82.4       : gtgtgaatatgatgtggatttttgggctattgagtttacaaagatggtttagactggaggattggaaattttggaactggcatgctagtttgggtgtgtct---nnnnnnnnnnnnnnnnnnnnaacaatt : 1527
EA00371.4   : --------------------------------------------------------------------------------------------------------nnnnnnnnnnnnnnnnnnnnaacaatt : 1527
Fla.8924.4  : --------------------------------------------------------------------------------------------------------nnnnnnnnnnnnnnnnnnnnaacaatt : 1527
Floradade.  : --------------------------------------------------------------------------------------------------------nnnnnnnnnnnnnnnnnnnnaacaatt : 1527
LYC1410.4   : --------------------------------------------------------------------------------------------------------nnnnnnnnnnnnnnnnnnnnaacaatt : 1527
PI169588.4  : --------------------------------------------------------------------------------------------------------nnnnnnnnnnnnnnnnnnnnaacaatt : 1527
Brandywine  : --------------------------------------------------------------------------------------------------------nnnnnnnnnnnnnnnnnnnnaacaatt : 1527
EA00990.4   : --------------------------------------------------------------------------------------------------------nnnnnnnnnnnnnnnnnnnnaacaatt : 1527
PI1303721.  : --------------------------------------------------------------------------------------------------------nnnnnnnnnnnnnnnnnnnnaacaatt : 1527
BGV006775.  : gtgtgaatatgatgtggatttttgggctattgagtttacaaagatggtttagactggaggattggaaattttggaactggcatgctagtttgggtgtgtctcgannnnnnnnnnnnnnnnnnnnaacaatt : 1527
PAS014479.  : gtgtgaatatgatgtggatttttgggctattgagtttacaaagatggtttagactggaggattggaaattttggaactggcatgctagtttgggtgtgtct---nnnnnnnnnnnnnnnnnnnnaacaatt : 1527
                                                                                                                      NNNNNNNNNNNNNNNNNNNNAACAATT

                *     2240         *       2260         *       2280         *       2300         *       2320         *       2340         *       23
ITAG.4      : atcaacattgaatagctcccactctttgataccacagctatctaatgcagatatcaacctttgtttttccttctcagctggatattgtccatctgggctgacaagaacagccccagtatatgactgccccg : 1658
BGV006865.  : atcaacattgaatagctcccactctttgataccacagctatctaatgcagatatcaacctttgtttttccttctcagctggatattgtccatctgggctgacaagaacagccccagtatatgactgccccg : 1658
BGV007931.  : atcaacattgaatagctcccactctttgataccacagctatctaatgcagatatcaacctttgtttttccttctcagctggatattgtccatctgggctgacaagaacagccccagtatatgactgccccg : 1658
BGV007989.  : atcaacattgaatagctcccactctttgataccacagctatctaatgcagatatcaacctttgtttttccttctcagctggatattgtccatctgggctgacaagaacagccccagtatatgactgccccg : 1658
M82.4       : atcaacattgaatagctcccactctttgataccacagctatctaatgcagatatcaacctttgtttttccttctcagctggatattgtccatctgggctgacaagaacagccccagtatatgactgccccg : 1658
EA00371.4   : atcaacattgaatagctcccactctttgataccacagctatctaatgcagatatcaacctttgtttttccttctcagctggatattgtccatctgggctgacaagaacagccccagtatatgactgccccg : 1658
Fla.8924.4  : atcaacattgaatagctcccactctttgataccacagctatctaatgcagatatcaacctttgtttttccttctcagctggatattgtccatctgggctgacaagaacagccccagtatatgactgccccg : 1658
Floradade.  : atcaacattgaatagctcccactctttgataccacagctatctaatgcagatatcaacctttgtttttccttctcagctggatattgtccatctgggctgacaagaacagccccagtatatgactgccccg : 1658
LYC1410.4   : atcaacattgaatagctcccactctttgataccacagctatctaatgcagatatcaacctttgtttttccttctcagctggatattgtccatctgggctgacaagaacagccccagtatatgactgccccg : 1658
PI169588.4  : atcaacattgaatagctcccactctttgataccacagctatctaatgcagatatcaacctttgtttttccttctcagctggatattgtccatctgggctgacaagaacagccccagtatatgactgccccg : 1658
Brandywine  : atcaacattgaatagctcccactctttgataccacagctatctaatgcagatatcaacctttgtttttccttctcagctggatattgtccatctgggctgacaagaacagccccagtatatgactgccccg : 1658
EA00990.4   : atcaacattgaatagctcccactctttgataccacagctatctaatgcagatatcaacctttgtttttccttctcagctggatattgtccatctgggctgacaagaacagccccagtatatgactgccccg : 1658
PI1303721.  : atcaacattgaatagctcccactctttgataccacagctatctaatgcagatatcaacctttgtttttccttctcagctggatattgtccatctgggctgacaagaacagccccagtatatgactgccccg : 1658
BGV006775.  : atcaacattgaatagctcccactctttgataccacagctatctaatgcagatatcaacctttgtttttccttctcagctggatattgtccatctgggctgacaagaacagccccagtatatgactgccccg : 1658
PAS014479.  : atcaacattgaatagctcccactctttgataccacagctatctaatgcagatatcaacctttgtttttccttctcagctggatattgtccatctgggctgacaagaacagccccagtatatgactgccccg : 1658
              ATCAACATTGAATAGCTCCCACTCTTTGATACCACAGCTATCTAATGCAGATATCAACCTTTGTTTTTCCTTCTCAGCTGGATATTGTCCATCTGGGCTGACAAGAACAGCCCCAGTATATGACTGCCCCG

              60         *     2380         *       2400         *       2420         *       2440         *       2460         *       2480
ITAG.4      : ccactcgtgcagctccatgatagtggaacagacccagctcaaatcatcagaaacatccacaattgtccagaactcgtttgaaacgactccattatccaatacactgaagtgaaatgttccggtaactttt : 1789
BGV006865.  : ccactcgtgcagctccatgatagtggaacagacccagctcaaatcatcagaaacatccacaattgtccagaactcgtttgaaacgactccattatccaatacactgaagtgaaatgttccggtaactttt : 1789
BGV007931.  : ccactcgtgcagctccatgatagtggaacagacccagctcaaatcatcagaaacatccacaattgtccagaactcgtttgaaacgactccattatccaatacactgaagtgaaatgttccggtaactttt : 1789
BGV007989.  : ccactcgtgcagctccatgatagtggaacagacccagctcaaatcatcagaaacatccacaattgtccagaactcgtttgaaacgactccattatccaatacactgaagtgaaatgttccggtaactttt : 1789
M82.4       : ccactcgtgcagctccatgatagtggaacagacccagctcaaatcatcagaaacatccacaattgtccagaactcgtttgaaacgactccattatccaatacactgaagtgaaatgttccggtaactttt : 1789
EA00371.4   : ccactcgtgcagctccatgatagtggaacagacccagctcaaatcatcagaaacatccacaattgtccagaactcgtttgaaacgactccattatccaatacactgaagtgaaatgttccggtaactttt : 1789
Fla.8924.4  : ccactcgtgcagctccatgatagtggaacagacccagctcaaatcatcagaaacatccacaattgtccagaactcgtttgaaacgactccattatccaatacactgaagtgaaatgttccggtaactttt : 1789
Floradade.  : ccactcgtgcagctccatgatagtggaacagacccagctcaaatcatcagaaacatccacaattgtccagaactcgtttgaaacgactccattatccaatacactgaagtgaaatgttccggtaactttt : 1789
LYC1410.4   : ccactcgtgcagctccatgatagtggaacagacccagctcaaatcatcagaaacatccacaattgtccagaactcgtttgaaacgactccattatccaatacactgaagtgaaatgttccggtaactttt : 1789
PI169588.4  : ccactcgtgcagctccatgatagtggaacagacccagctcaaatcatcagaaacatccacaattgtccagaactcgtttgaaacgactccattatccaatacactgaagtgaaatgttccggtaactttt : 1789
Brandywine  : ccactcgtgcagctccatgatagtggaacagacccagctcaaatcatcagaaacatccacaattgtccagaactcgtttgaaacgactccattatccaatacactgaagtgaaatgttccggtaactttt : 1789
EA00990.4   : ccactcgtgcagctccatgatagtggaacagacccagctcaaatcatcagaaacatccacaattgtccagaactcgtttgaaacgactccattatccaatacactgaagtgaaatgttccggtaactttt : 1789
PI1303721.  : ccactcgtgcagctccatgatagtggaacagacccagctcaaatcatcagaaacatccacaattgtccagaactcgtttgaaacgactccattatccaatacactgaagtgaaatgttccggtaactttt : 1789
BGV006775.  : ccactcgtgcagctccatgatagtggaacagacccagctcaaatcatcagaaacatccacaattgtccagaactcgtttgaaacgactccattatccaatacactgaagtgaaatgttccggtaactttt : 1789
PAS014479.  : ccactcgtgcagctccatgatagtggaacagacccagctcaaatcatcagaaacatccacaattgtccagaactcgtttgaaacgactccattatccaatacactgaagtgaaatgttccggtaactttt : 1789
              CCACTCGTGCAGCTCCATGATAGTGGAACAGACCCAGCTCAAATCATCAGAAACATCCACAATTGTCCAGAACTCGTTTGAAACGACTCCATTATCCAATACACTGAAGTGAAATGTTCCGGTAACTTTT

              *     2500         *       2520         *       2540         *       2560         *       2580         *       2600         *       2620
ITAG.4      : cctcttttcactctatacttgcgccttctccagactaggtctccttcaagtgttcttacctggaaaactggttcataccagaatgatcctctagcttttccccgatagaataattggtactggcatggaaa : 1920
BGV006865.  : cctcttttcactctatacttgcgccttctccagactaggtctccttcaagtgttcttacctggaaaactggttcataccagaatgatcctctagcttttccccgatagaataattggtactggcatggaaa : 1920
BGV007931.  : cctcttttcactctatacttgcgccttctccagactaggtctccttcaagtgttcttacctggaaaactggttcataccagaatgatcctctagcttttccccgatagaataattggtactggcatggaaa : 1920
BGV007989.  : cctcttttcactctatacttgcgccttctccagactaggtctccttcaagtgttcttacctggaaaactggttcataccagaatgatcctctagcttttccccgatagaataattggtactggcatggaaa : 1920
M82.4       : cctcttttcactctatacttgcgccttctccagactaggtctccttcaagtgttcttacctggaaaactggttcataccagaatgatcctctagcttttccccgatagaataattggtactggcatggaaa : 1920
EA00371.4   : cctcttttcactctatacttgcgccttctccagactaggtctccttcaagtgttcttacctggaaaactggttcataccagaatgatcctctagcttttccccgatagaataattggtactggcatggaaa : 1920
Fla.8924.4  : cctcttttcactctatacttgcgccttctccagactaggtctccttcaagtgttcttacctggaaaactggttcataccagaatgatcctctagcttttccccgatagaataattggtactggcatggaaa : 1920
Floradade.  : cctcttttcactctatacttgcgccttctccagactaggtctccttcaagtgttcttacctggaaaactggttcataccagaatgatcctctagcttttccccgatagaataattggtactggcatggaaa : 1920
LYC1410.4   : cctcttttcactctatacttgcgccttctccagactaggtctccttcaagtgttcttacctggaaaactggttcataccagaatgatcctctagcttttccccgatagaataattggtactggcatggaaa : 1920
PI169588.4  : cctcttttcactctatacttgcgccttctccagactaggtctccttcaagtgttcttacctggaaaactggttcataccagaatgatcctctagcttttccccgatagaataattggtactggcatggaaa : 1920
Brandywine  : cctcttttcactctatacttgcgccttctccagactaggtctccttcaagtgttcttacctggaaaactggttcataccagaatgatcctctagcttttccccgatagaataattggtactggcatggaaa : 1920
EA00990.4   : cctcttttcactctatacttgcgccttctccagactaggtctccttcaagtgttcttacctggaaaactggttcataccagaatgatcctctagcttttccccgatagaataattggtactggcatggaaa : 1920
PI1303721.  : cctcttttcactctatacttgcgccttctccagactaggtctccttcaagtgttcttacctggaaaactggttcataccagaatgatcctctagcttttccccgatagaataattggtactggcatggaaa : 1920
BGV006775.  : cctcttttcactctatacttgcgccttctccagactaggtctccttcaagtgttcttacctggaaaactggttcataccagaatgatcctctagcttttccccgatagaataattggtactggcatggaaa : 1920
PAS014479.  : cctcttttcactctatacttgcgccttctccagactaggtctccttcaagtgttcttacctggaaaactggttcataccagaatgatcctctagcttttccccgatagaataattggtactggcatggaaa : 1920
              CCTCTTTTCACTCTATACTTGCGCCTTCTCCAGACTAGGTCTCCTTCAAGTGTTCTTACCTGGAAAACTGGTTCATACCAGAATGATCCTCTAGCTTTTCCCCGATAGAATAATTGGTACTGGCATGGAAA

                      *     2640         *       2660         *       2680         *       2700         *       2720         *       2740         *
ITAG.4      : ttgatcataagctggattctgcccggctacaacgcgccaactccattccaatgtccctaaccaaccaacaaggaggtcttcagcaatttcagaagacaatttctcccctcgaaactctgccattggagtca : 2051
BGV006865.  : ttgatcataagctggattctgcccggctacaacgcgccaactccattccaatgtccctaaccaaccaacaaggaggtcttcagcaatttcagaagacaatttctcccctcgaaactctgccattggagtca : 2051
BGV007931.  : ttgatcataagctggattctgcccggctacaacgcgccaactccattccaatgtccctaaccaaccaacaaggaggtcttcagcaatttcagaagacaatttctcccctcgaaactctgccattggagtca : 2051
BGV007989.  : ttgatcataagctggattctgcccggctacaacgcgccaactccattccaatgtccctaaccaaccaacaaggaggtcttcagcaatttcagaagacaatttctcccctcgaaactctgccattggagtca : 2051
M82.4       : ttgatcataagctggattctgcccggctacaacgcgccaactccattccaatgtccctaaccaaccaacaaggaggtcttcagcaatttcagaagacaatttctcccctcgaaactctgccattggagtca : 2051
EA00371.4   : ttgatcataagctggattctgcccggctacaacgcgccaactccattccaatgtccctaaccaaccaacaaggaggtcttcagcaatttcagaagacaatttctcccctcgaaactctgccattggagtca : 2051
Fla.8924.4  : ttgatcataagctggattctgcccggctacaacgcgccaactccattccaatgtccctaaccaaccaacaaggaggtcttcagcaatttcagaagacaatttctcccctcgaaactctgccattggagtca : 2051
Floradade.  : ttgatcataagctggattctgcccggctacaacgcgccaactccattccaatgtccctaaccaaccaacaaggaggtcttcagcaatttcagaagacaatttctcccctcgaaactctgccattggagtca : 2051
LYC1410.4   : ttgatcataagctggattctgcccggctacaacgcgccaactccattccaatgtccctaaccaaccaacaaggaggtcttcagcaatttcagaagacaatttctcccctcgaaactctgccattggagtca : 2051
PI169588.4  : ttgatcataagctggattctgcccggctacaacgcgccaactccattccaatgtccctaaccaaccaacaaggaggtcttcagcaatttcagaagacaatttctcccctcgaaactctgccattggagtca : 2051
Brandywine  : ttgatcataagctggattctgcccggctacaacgcgccaactccattccaatgtccctaaccaaccaacaaggaggtcttcagcaatttcagaagacaatttctcccctcgaaactctgccattggagtca : 2051
EA00990.4   : ttgatcataagctggattctgcccggctacaacgcgccaactccattccaatgtccctaaccaaccaacaaggaggtcttcagcaatttcagaagacaatttctcccctcgaaactctgccattggagtca : 2051
PI1303721.  : ttgatcataagctggattctgcccggctacaacgcgccaactccattccaatgtccctaaccaaccaacaaggaggtcttcagcaatttcagaagacaatttctcccctcgaaactctgccattggagtca : 2051
BGV006775.  : ttgatcataagctggattctgcccggctacaacgcgccaactccattccaatgtccctaaccaaccaacaaggaggtcttcagcaatttcagaagacaatttctcccctcgaaactctgccattggagtca : 2051
PAS014479.  : ttgatcataagctggattctgcccggctacaacgcgccaactccattccaatgtccctaaccaaccaacaaggaggtcttcagcaatttcagaagacaatttctcccctcgaaactctgccattggagtca : 2051
              TTGATCATAAGCTGGATTCTGCCCGGCTACAACGCGCCAACTCCATTCCAATGTCCCTAACCAACCAACAAGGAGGTCTTCAGCAATTTCAGAAGACAATTTCTCCCCTCGAAACTCTGCCATTGGAGTCA

                  2760         *       2780         *       2800         *       2820         *       2840         *       2860         *       2880
ITAG.4      : cataaggcttttcaggaatctttgcatcaagttcaagacaattattcttctgtagcacacagagagagaactcttccaggtatttgctctcataggaagcaatacagcgataattgcatacctgatctaca : 2182
BGV006865.  : cataaggcttttcaggaatctttgcatcaagttcaagacaattattcttctgtagcacacagagagagaactcttccaggtatttgctctcataggaagcaatacagcgataattgcatacctgatctaca : 2182
BGV007931.  : cataaggcttttcaggaatctttgcatcaagttcaagacaattattcttctgtagcacacagagagagaactcttccaggtatttgctctcataggaagcaatacagcgataattgcatacctgatctaca : 2182
BGV007989.  : cataaggcttttcaggaatctttgcatcaagttcaagacaattattcttctgtagcacacagagagagaactcttccaggtatttgctctcataggaagcaatacagcgataattgcatacctgatctaca : 2182
M82.4       : cataaggcttttcaggaatctttgcatcgagttcaagacaattattcttctgtagcacacagagagagaactcttccaggtatttgctctcataggaagcaatacagcgataattgcatacctgatctaca : 2182
EA00371.4   : cataaggcttttcaggaatctttgcatcaagttcaagacaattattcttctgtagcacacagagagagaactcttccaggtatttgctctcataggaagcaatacagcgataattgcatacctgatctaca : 2182
Fla.8924.4  : cataaggcttttcaggaatctttgcatcaagttcaagacaattattcttctgtagcacacagagagagaactcttccaggtatttgctctcataggaagcaatacagcgataattgcatacctgatctaca : 2182
Floradade.  : cataaggcttttcaggaatctttgcatcaagttcaagacaattattcttctgtagcacacagagagagaactcttccaggtatttgctctcataggaagcaatacagcgataattgcatacctgatctaca : 2182
LYC1410.4   : cataaggcttttcaggaatctttgcatcaagttcaagacaattattcttctgtagcacacagagagagaactcttccaggtatttgctctcataggaagcaatacagcgataattgcatacctgatctaca : 2182
PI169588.4  : cataaggcttttcaggaatctttgcatcaagttcaagacaattattcttctgtagcacacagagagagaactcttccaggtatttgctctcataggaagcaatacagcgataattgcatacctgatctaca : 2182
Brandywine  : cataaggcttttcaggaatctttgcatcaagttcaagacaattattcttctgtagcacacagagagagaactcttccaggtatttgctctcataggaagcaatacagcgataattgcatacctgatctaca : 2182
EA00990.4   : cataaggcttttcaggaatctttgcatcaagttcaagacaattattcttctgtagcacacagagagagaactcttccaggtatttgctctcataggaagcaatacagcgataattgcatacctgatctaca : 2182
PI1303721.  : cataaggcttttcaggaatctttgcatcaagttcaagacaattattcttctgtagcacacagagagagaactcttccaggtatttgctctcataggaagcaatacagcgataattgcatacctgatctaca : 2182
BGV006775.  : cataaggcttttcaggaatctttgcatcaagttcaagacaattattcttctgtagcacacagagagagaactcttccaggtatttgctctcataggaagcaatacagcgataattgcatacctgatctaca : 2182
PAS014479.  : cataaggcttttcaggaatctttgcatcaagttcaagacaattattcttctgtagcacacagagagagaactcttccaggtatttgctctcataggaagcaatacagcgataattgcatacctgatctaca : 2182
              CATAAGGCTTTTCAGGAATCTTTGCATCaAGTTCAAGACAATTATTCTTCTGTAGCACACAGAGAGAGAACTCTTCCAGGTATTTGCTCTCATAGGAAGCAATACAGCGATAATTGCATACCTGATCTACA

                    *     2900         *       2920         *       2940         *       2960         *       2980         *       3000         *
ITAG.4      : gggctgcagttgttcaagcattgaagtgcctttctacagttaggatctaatagacagttcaatatctgaggcccgcaatttgtcaccatgcagttcaaagtggaaaatccttttgctctaaggttcttcag : 2313
BGV006865.  : gggctgcagttgttcaagcattgaagtgcctttctacagttaggatctaatagacagttcaatatctgaggcccgcaatttgtcaccatgcagttcaaagtggaaaatccttttgctctaaggttcttcag : 2313
BGV007931.  : gggctgcagttgttcaagcattgaagtgcctttctacagttaggatctaatagacagttcaatatctgaggcccgcaatttgtcaccatgcagttcaaagtggaaaatccttttgctctaaggttcttcag : 2313
BGV007989.  : gggctgcagttgttcaagcattgaagtgcctttctacagttaggatctaatagacagttcaatatctgaggcccgcaatttgtcaccatgcagttcaaagtggaaaatccttttgctctaaggttcttcag : 2313
M82.4       : gggctgcagttgttcaagcattgaagtgcctttctacagttaggatctaatagacagttcaatatctgaggcccgcaatttgtcaccatgcagttcaaagtggaaaatccttttgctctaaggttcttcag : 2313
EA00371.4   : gggctgcagttgttcaagcattgaagtgcctttctacagttaggatctaatagacagttcaatatctgaggcccgcaatttgtcaccatgcagttcaaagtggaaaatccttttgctctaaggttcttcag : 2313
```

```
Fla.8924.4 : gggctgcagttgttcaagcattgaagtgcctttctacagttaggatctaatagacagttcaatatctgaggcccgcaatttgtcaccatgcagttcaaagtggaaaatccttttgctctaaggttcttcag : 2313
Floradade. : gggctgcagttgttcaagcattgaagtgcctttctacagttaggatctaatagacagttcaatatctgaggcccgcaatttgtcaccatgcagttcaaagtggaaaatccttttgctctaaggttcttcag : 2313
LYC1410.4  : gggctgcagttgttcaagcattgaagtgcctttctacagttaggatctaatagacagttcaatatctgaggcccgcaatttgtcaccatgcagttcaaagtggaaaatccttttgctctaaggttcttcag : 2313
PI169588.4 : gggctgcagttgttcaagcattgaagtgcctttctacagttaggatctaatagacagttcaatatctgaggcccgcaatttgtcaccatgcagttcaaagtggaaaatccttttgctctaaggttcttcag : 2313
Brandywine : gggctgcagttgttcaagcattgaagtgcctttctacagttaggatctaatagacagttcaatatctgaggcccgcaatttgtcaccatgcagttcaaagtggaaaatccttttgctctaaggttcttcag : 2313
EA00990.4  : gggctgcagttgttcaagcattgaagtgcctttctacagttaggatctaatagacagttcaatatctgaggcccgcaatttgtcaccatgcagttcaaagtggaaaatccttttgctctaaggttcttcag : 2313
PI1303721. : gggctgcagttgttcaagcattgaagtgcctttctacagttaggatctaatagacagttcaatatctgaggcccgcaatttgtcaccatgcagttcaaagtggaaaatccttttgctctaaggttcttcag : 2313
BGV006775. : gggctgcagttgttcaagcattgaagtgcctttctacagttaggatctaatagacagttcaatatctgaggcccgcaatttgtcaccatgcagttcaaagtggaaaatccttttgctctaaggttcttcag : 2313
PAS014479. : gggctgcagttgttcaagcattgaagtgcctttctacagttaggatctaatagacagttcaatatctgaggcccgcaatttgtcaccatgcagttcaaagtggaaaatccttttgctctaaggttcttcag : 2313
             GGGCTGCAGTTGTTCAAGCATTGAAGTGCCTTTCTACAGTTAGGATCTAATAGACAGTTCAATATCTGAGGCCCGCAATTTGTCACCATGCAGTTCAAAGTGGAAAATCCTTTTGCTCTAAGGTTCTTCAG

                3020         *       3040         *       3060         *       3080         *       3100         *       3120         *       3140
ITAG.4     : tgttgagaccggtgttatataagcattaattataactaataagcagaaccgtatgtcatcagaattatgcctttcccaagcatcaaacactgttcgggttatctccacagattcatccagtttcttagatt : 2444
BGV006865. : tgttgagaccggtgttatataagcattaattataactaataagcagaaccgtatgtcatcagaattatgcctttcccaagcatcaaacactgttcgggttatctccacagattcatccagtttcttagatt : 2444
BGV007931. : tgttgagaccggtgttatataagcattaattataactaataagcagaaccgtatgtcatcagaattatgcctttcccaagcatcaaacactgttcgggttatctccacagattcatccagtttcttagatt : 2444
BGV007989. : tgttgagaccggtgttatataagcattaattataactaataagcagaaccgtatgtcatcagaattatgcctttcccaagcatcaaacactgttcgggttatctccacagattcatccagtttcttagatt : 2444
M82.4      : tgttgagaccggtgttatataagcattaattataactaataagcagaaccgtatgtcatcagaattatgcctttcccaagcatcaaacactgttcgggttatctccacagattcatccagtttcttagatt : 2444
EA00371.4  : tgttgagaccggtgttatataagcattaattataactaataagcagaaccgtatgtcatcagaattatgcctttcccaagcatcaaacactgttcgggttatctccacagattcatccagtttcttagatt : 2444
Fla.8924.4 : tgttgagaccggtgttatataagcattaattataactaataagcagaaccgtatgtcatcagaattatgcctttcccaagcatcaaacactgttcgggttatctccacagattcatccagtttcttagatt : 2444
Floradade. : tgttgagaccggtgttatataagcattaattataactaataagcagaaccgtatgtcatcagaattatgcctttcccaagcatcaaacactgttcgggttatctccacagattcatccagtttcttagatt : 2444
LYC1410.4  : tgttgagaccggtgttatataagcattaattataactaataagcagaaccgtatgtcatcagaattatgcctttcccaagcatcaaacactgttcgggttatctccacagattcatccagtttcttagatt : 2444
PI169588.4 : tgttgagaccggtgttatataagcattaattataactaataagcagaaccgtatgtcatcagaattatgcctttcccaagcatcaaacactgttcgggttatctccacagattcatccagtttcttagatt : 2444
Brandywine : tgttgagaccggtgttatataagcattaattataactaataagcagaaccgtatgtcatcagaattatgcctttcccaagcatcaaacactgttcgggttatctccacagattcatccagtttcttagatt : 2444
EA00990.4  : tgttgagaccggtgttatataagcattaattataactaataagcagaaccgtatgtcatcagaattatgcctttcccaagcatcaaacactgttcgggttatctccacagattcatccagtttcttagatt : 2444
PI1303721. : tgttgagaccggtgttatataagcattaattataactaataagcagaaccgtatgtcatcagaattatgcctttcccaagcatcaaacactgttcgggttatctccacagattcatccagtttcttagatt : 2444
BGV006775. : tgttgagaccggtgttatataagcattaattataactaataagcagaaccgtatgtcatcagaattatgcctttcccaagcatcaaacactgttcgggttatctccacagattcatccagtttcttagatt : 2444
PAS014479. : tgttgagaccggtgttatataagcattaattataactaataagcagaaccgtatgtcatcagaattatgcctttcccaagcatcaaacactgttcgggttatctccacagattcatccagtttcttagatt : 2444
             TGTTGAGACCGGTGTTATATAAGCATTAATTATAACTAATAAGCAGAACCGTATGTCATCAGAATTATGCCTTTCCCAAGCATCAAACACTGTTCGGGTTATCTCCACAGATTCATCCAGTTTCTTAGATT

                   *       3160         *       3180         *       3200         *       3220         *       3240         *       3260         *
ITAG.4     : gagagaatggaagtaaactgctaaatatgtctccccttttttttagtttcaacaaaacttccacctagcttgtttctgagttccatagaggaatcaaagcatatgatgtttgggacattcaggctgttagac : 2575
BGV006865. : gagagaatggaagtaaactgctaaatatgtctcccctttttttcgtttcaacaaaacttccacctagcttgtttctgagttccatagaggaatcaaagcatatgatgtttgggacattcaggctgttagac : 2575
BGV007931. : gagagaatggaagtaaactgctaaatatgtctcccctttttttagtttcaacaaaacttccacctagcttgtttctgagttccatagaggaatcaaagcatatgatgtttgggacattcaggctgttagac : 2575
BGV007989. : gagagaatggaagtaaactgctaaatatgtctcccctttttttagtttcaacaaaacttccacctagcttgtttctgagttccatagaggaatcaaagcatatgatgtttgggacattcaggctgttagac : 2575
M82.4      : gagagaatggaagtaaactgctaaatatgtctcccctttttttcgtttcaacaaaacttccacctagcttgtttctgagttccatagaggaatcaaagcatatgatgtttgggacattcaggctgttagac : 2575
EA00371.4  : gagagaatggaagtaaactgctaaatatgtctcccctttttttagtttcaacaaaacttccacctagcttgtttctgagttccatagaggaatcaaagcatatgatgtttgggacattcaggctgttagac : 2575
Fla.8924.4 : gagagaatggaagtaaactgctaaatatgtctcccctttttttagtttcaacaaaacttccacctagcttgtttctgagttccatagaggaatcaaagcatatgatgtttgggacattcaggctgttagac : 2575
Floradade. : gagagaatggaagtaaactgctaaatatgtctcccctttttttagtttcaacaaaacttccacctagcttgtttctgagttccatagaggaatcaaagcatatgatgtttgggacattcaggctgttagac : 2575
LYC1410.4  : gagagaatggaagtaaactgctaaatatgtctcccctttttttagtttcaacaaaacttccacctagcttgtttctgagttccatagaggaatcaaagcatatgatgtttgggacattcaggctgttagac : 2575
PI169588.4 : gagagaatggaagtaaactgctaaatatgtctcccctttttttagtttcaacaaaacttccacctagcttgtttctgagttccatagaggaatcaaagcatatgatgtttgggacattcaggctgttagac : 2575
Brandywine : gagagaatggaagtaaactgctaaatatgtctcccctttttttagtttcaacaaaacttccacctagcttgtttctgagttccatagaggaatcaaagcatatgatgtttgggacattcaggctgttagac : 2575
EA00990.4  : gagagaatggaagtaaactgctaaatatgtctcccctttttttagtttcaacaaaacttccacctagcttgtttctgagttccatagaggaatcaaagcatatgatgtttgggacattcaggctgttagac : 2575
PI1303721. : gagagaatggaagtaaactgctaaatatgtctcccctttttttagtttcaacaaaacttccacctagcttgtttctgagttccatagaggaatcaaagcatatgatgtttgggacattcaggctgttagac : 2575
BGV006775. : gagagaatggaagtaaactgctaaatatgtctcccctttttttcgtttcaacaaaacttccacctagcttgtttctgagttccatagaggaatcaaagcatatgatgtttgggacattcaggctgttagac : 2575
PAS014479. : gagagaatggaagtaaactgctaaatatgtctcccctttttttcgtttcaacaaaacttccacctagcttgtttctgagttccatagaggaatcaaagcatatgatgtttgggacattcaggctgttagac : 2575
             GAGAGAATGGAAGTAAACTGCTAAATATGTCTCCCCTTTTTTT GTTTCAACAAAACTTCCACCTAGCTTGTTTCTGAGTTCCATAGAGGAATCAAAGCATATGATGTTTGGGACATTCAGGCTGTTAGAC

              3280         *       3300         *       3320         *       3340         *       3360         *       3380         *       3400
ITAG.4     : tgtatccagttgactgatccttcctttgtgacagcaacgaccaccaaaatgtcagcatgatccagttcattgctgacgtggttggcagtttcatcttcagatttgacaaagttgtcagaaaagacaatcat : 2706
BGV006865. : tgtatccagttgactgatccttcctttgtgacagcaacgaccaccaaaatgtcagcatgatccagttcattgctgacgtggttggcagtttcatcttcagatttgacaaagttgtcagaaaagacaatcat : 2706
BGV007931. : tgtatccagttgactgatccttcctttgtgacagcaacgaccaccaaaatgtcagcatgatccagttcattgctgacgtggttggcagtttcatcttcagatttgacaaagttgtcagaaaagacaatcat : 2706
BGV007989. : tgtatccagttgactgatccttcctttgtgacagcaacgaccaccaaaatgtcagcatgatccagttcattgctgacgtggttggcagtttcatcttcagatttgacaaagttgtcagaaaagacaatcat : 2706
M82.4      : tgtatccagttgactgatccttcctttgtgacagcaacgaccaccaaaatgtcagcatgatccagttcattgctgacgtggttggcagtttcatcttcagatttgacaaagttgtcagaaaagacaatcat : 2706
EA00371.4  : tgtatccagttgactgatccttcctttgtgacagcaacgaccaccaaaatgtcagcatgatccagttcattgctgacgtggttggcagtttcatcttcagatttgacaaagttgtcagaaaagacaatcat : 2706
Fla.8924.4 : tgtatccagttgactgatccttcctttgtgacagcaacgaccaccaaaatgtcagcatgatccagttcattgctgacgtggttggcagtttcatcttcagatttgacaaagttgtcagaaaagacaatcat : 2706
Floradade. : tgtatccagttgactgatccttcctttgtgacagcaacgaccaccaaaatgtcagcatgatccagttcattgctgacgtggttggcagtttcatcttcagatttgacaaagttgtcagaaaagacaatcat : 2706
LYC1410.4  : tgtatccagttgactgatccttcctttgtgacagcaacgaccaccaaaatgtcagcatgatccagttcattgctgacgtggttggcagtttcatcttcagatttgacaaagttgtcagaaaagacaatcat : 2706
PI169588.4 : tgtatccagttgactgatccttcctttgtgacagcaacgaccaccaaaatgtcagcatgatccagttcattgctgacgtggttggcagtttcatcttcagatttgacaaagttgtcagaaaagacaatcat : 2706
Brandywine : tgtatccagttgactgatccttcctttgtgacagcaacgaccaccaaaatgtcagcatgatccagttcattgctgacgtggttggcagtttcatcttcagatttgacaaagttgtcagaaaagacaatcat : 2706
EA00990.4  : tgtatccagttgactgatccttcctttgtgacagcaacgaccaccaaaatgtcagcatgatccagttcattgctgacgtggttggcagtttcatcttcagatttgacaaagttgtcagaaaagacaatcat : 2706
PI1303721. : tgtatccagttgactgatccttcctttgtgacagcaacgaccaccaaaatgtcagcatgatccagttcattgctgacgtggttggcagtttcatcttcagatttgacaaagttgtcagaaaagacaatcat : 2706
BGV006775. : tgtatccagttgactgatccttcctttgtgacagcaacgaccaccaaaatgtcagcatgatccagttcattgctgacgtggttggcagtttcatcttcagatttgacaaagttgtcagaaaagacaatcat : 2706
PAS014479. : tgtatccagttgactgatccttcctttgtgacagcaacgaccaccaaaatgtcagcatgatccagttcattgctgacgtggttggcagtttcatcttcagatttgacaaagttgtcagaaaagacaatcat : 2706
             TGTATCCAGTTGACTGATCCTTCCTTTGTGACAGCAACGACCACCAAAATGTCAGCATGATCCAGTTCATTGCTGACGTGGTTGGCAGTTTCATCTTCAGATTTGACAAAGTTGTCAGAAAAGACAATCAT

                 *       3420         *       3440         *       3460         *       3480         *       3500         *       3520         *       3
ITAG.4     : ttcaaattcttcatcaacccatttcagtctctctgccttgcatttataaaaggaagcaaagtacaacatcatcaacaaagtacaattcatatatatatactcttttccctttcttttctctcgtaactag : 2837
BGV006865. : ttcaaattcttcatcaacccatttcagtctctctgccttgcatttataaaaggaagcaaagtacaacatcatcaacaaagtacaattcatatatatatactcttttccctttcttttctctcgtaactag : 2837
BGV007931. : ttcaaattcttcatcaacccatttcagtctctctgccttgcatttataaaaggaagcaaagtacaacatcatcaacaaagtacaattcatatatatatactcttttccctttcttttctctcgtaactag : 2837
BGV007989. : ttcaaattcttcatcaacccatttcagtctctctgccttgcatttataaaaggaagcaaagtacaacatcatcaacaaagtacaattcatatatatatactcttttccctttcttttctctcgtaactag : 2837
M82.4      : ttcaaattcttcatcaacccatttcagtctctctgccttgcatttataaaaggaagcaaagtacaacatcatcaacaaagtacaattcatatatatatactcttttccctttcttttctctcgtaactag : 2837
EA00371.4  : ttcaaattcttcatcaacccatttcagtctctctgccttgcatttataaaaggaagcaaagtacaacatcatcaacaaagtacaattcatatatatatactcttttccctttcttttctctcgtaactag : 2837
Fla.8924.4 : ttcaaattcttcatcaacccatttcagtctctctgccttgcatttataaaaggaagcaaagtacaacatcatcaacaaagtacaattcatatatatatactcttttccctttcttttctctcgtaactag : 2837
Floradade. : ttcaaattcttcatcaacccatttcagtctctctgccttgcatttataaaaggaagcaaagtacaacatcatcaacaaagtacaattcatatatatatactcttttccctttcttttctctcgtaactag : 2837
LYC1410.4  : ttcaaattcttcatcaacccatttcagtctctctgccttgcatttataaaaggaagcaaagtacaacatcatcaacaaagtacaattcatatatatatactcttttccctttcttttctctcgtaactag : 2837
PI169588.4 : ttcaaattcttcatcaacccatttcagtctctctgccttgcatttataaaaggaagcaaagtacaacatcatcaacaaagtacaattcatatatatatactcttttccctttcttttctctcgtaactag : 2837
Brandywine : ttcaaattcttcatcaacccatttcagtctctctgccttgcatttataaaaggaagcaaagtacaacatcatcaacaaagtacaattcatatatatatactcttttccctttcttttctctcgtaactag : 2837
EA00990.4  : ttcaaattcttcatcaacccatttcagtctctctgccttgcatttataaaaggaagcaaagtacaacatcatcaacaaagtacaattcatatatatatactcttttccctttcttttctctcgtaactag : 2837
PI1303721. : ttcaaattcttcatcaacccatttcagtctctctgccttgcatttataaaaggaagcaaagtacaacatcatcaacaaagtacaattcatatatatatactcttttccctttcttttctctcgtaactag : 2837
BGV006775. : ttcaaattcttcatcaacccatttcagtctctctgccttgcatttataaaaggaagcaaagtacaacatcatcaacaaagtacaattcatatatatatactcttttccctttcttttctctcgtaactag : 2837
PAS014479. : ttcaaattcttcatcaacccatttcagtctctctgccttgcatttataaaaggaagcaaagtacaacatcatcaacaaagtacaattcatatatatatactcttttccctttcttttctctcgtaactag : 2837
             TTCAAATTCTTCATCAACCCATTTCAGTCTCTCTGCCTTGCATTTATAAAAGGAAGCAAAGTACAACATCATCAACAAAGTACAATTCATATATATATACTCTTTTCCCTTTCTTTTCTCTCGTAACTAG

             540         *       3560         *       3580         *       3600         *       3620         *       3640         *       3660
ITAG.4     : tatgtttccgtttgccagattatctcataatttcattcctacaagtagctaaaccagcagcggatattgctcatcatcaaattccacaggatgatgaagcaacagagcagaatttcagcagtctatcaact : 2968
BGV006865. : tatgtttccgtttgccagattatctcataatttcattcctacaagtagctaaaccagcagcggatattgctcatcatcaaattccgcaggatgatgaagcaacagagcagaatttcagcagtctatcaact : 2968
BGV007931. : tatgtttccgtttgccagattatctcataatttcattcctacaagtagctaaaccagcagcggatattgctcatcatcaaattccacaggatgatgaagcaacagagcagaatttcagcagtctatcaact : 2968
BGV007989. : tatgtttccgtttgccagattatctcataatttcattcctacaagtagctaaaccagcagcggatattgctcatcatcaaattccacaggatgatgaagcaacagagcagaatttcagcagtctatcaact : 2968
M82.4      : tatgtttccgtttgccagattatctcataatttcattcctacaagtagctaaaccagcagcggatattgctcatcatcaaattccacaggatgatgaagcaacagagcagaatttcagcagtctatcaact : 2968
EA00371.4  : tatgtttccgtttgccagattatctcataatttcattcctacaagtagctaaaccagcagcggatattgctcatcatcaaattccacaggatgatgaagcaacagagcagaatttcagcagtctatcaact : 2968
Fla.8924.4 : tatgtttccgtttgccagattatctcataatttcattcctacaagtagctaaaccagcagcggatattgctcatcatcaaattccacaggatgatgaagcaacagagcagaatttcagcagtctatcaact : 2968
Floradade. : tatgtttccgtttgccagattatctcataatttcattcctacaagtagctaaaccagcagcggatattgctcatcatcaaattccacaggatgatgaagcaacagagcagaatttcagcagtctatcaact : 2968
LYC1410.4  : tatgtttccgtttgccagattatctcataatttcattcctacaagtagctaaaccagcagcggatattgctcatcatcaaattccacaggatgatgaagcaacagagcagaatttcagcagtctatcaact : 2968
PI169588.4 : tatgtttccgtttgccagattatctcataatttcattcctacaagtagctaaaccagcagcggatattgctcatcatcaaattccacaggatgatgaagcaacagagcagaatttcagcagtctatcaact : 2968
Brandywine : tatgtttccgtttgccagattatctcataatttcattcctacaagtagctaaaccagcagcggatattgctcatcatcaaattccacaggatgatgaagcaacagagcagaatttcagcagtctatcaact : 2968
EA00990.4  : tatgtttccgtttgccagattatctcataatttcattcctacaagtagctaaaccagcagcggatattgctcatcatcaaattccacaggatgatgaagcaacagagcagaatttcagcagtctatcaact : 2968
PI1303721. : tatgtttccgtttgccagattatctcataatttcattcctacaagtagctaaaccagcagcggatattgctcatcatcaaattccacaggatgatgaagcaacagagcagaatttcagcagtctatcaact : 2968
BGV006775. : tatgtttccgtttgccagattatctcataatttcattcctacaagtagctaaaccagcagcggatattgctcatcatcaaattccgcaggatgatgaagcaacagagcagaatttcagcagtctatcaact : 2968
PAS014479. : tatgtttccgtttgccagattatctcataatttcattcctacaagtagctaaaccagcagcggatattgctcatcatcaaattccacaggatgatgaagcaacagagcagaatttcagcagtctatcaact : 2968
             TATGTTTCCGTTTGCCAGATTATCTCATAATTTCATTCCTACAAGTAGCTAAACCAGCAGCGGATATTGCTCATCATCAAATTCCaCAGGATGATGAAGCAACAGAGCAGAATTTCAGCAGTCTATCAACT

               *       3680         *       3700         *       3720
ITAG.4     : agttgtatgagcaacatatttttgaaatccggagcccaacaaaaatttcaaa : 3020
BGV006865. : agttgtatgagcaacatatttttgaaatccggagcccaacaaaaatttcaaa : 3020
BGV007931. : agttgtatgagcaacatatttttgaaatccggagcccaacaaaaatttcaaa : 3020
BGV007989. : agttgtatgagcaacatatttttgaaatccggagcccaacaaaaatttcaaa : 3020
M82.4      : agttgtatgagcaacatatttttgaaatccggagcccaacaaaaatttcaaa : 3020
EA00371.4  : agttgtatgagcaacatatttttgaaatccggagcccaacaaaaatttcaaa : 3020
Fla.8924.4 : agttgtatgagcaacatatttttgaaatccggagcccaacaaaaatttcaaa : 3020
Floradade. : agttgtatgagcaacatatttttgaaatccggagcccaacaaaaatttcaaa : 3020
LYC1410.4  : agttgtatgagcaacatatttttgaaatccggagcccaacaaaaatttcaaa : 3020
PI169588.4 : agttgtatgagcaacatatttttgaaatccggagcccaacaaaaatttcaaa : 3020
Brandywine : agttgtatgagcaacatatttttgaaatccggagcccaacaaaaatttcaaa : 3020
EA00990.4  : agttgtatgagcaacatatttttgaaatccggagcccaacaaaaatttcaaa : 3020
PI1303721. : agttgtatgagcaacatatttttgaaatccggagcccaacaaaaatttcaaa : 3020
BGV006775. : agttgtatgagcaacatatttttgaaatccggagcccaacaaaaatttcaaa : 3020
PAS014479. : agttgtatgagcaacatatttttgaaatccggagcccaacaaaaatttcaaa : 3020
             AGTTGTATGAGCAACATATTTTTGAAATCCGGAGCCCAACAAAAATTTCAAA
```
