# Supplementary material for: Deep learning the cis-regulatory code for gene expression in selected model plants
Source: Nat Commun. 2024 Apr 25;15:3488. doi: 10.1038/s41467-024-47744-0 (PMC11045779; doi:10.1038/s41467-024-47744-0)
Supplement: Supplementary file 15 — Reporting Summary [file 41467_2024_47744_MOESM15_ESM.pdf]

nature portfolio

Corresponding author(s): Jędrzej Jakub Szymański

Last updated by author(s): Mar 11, 2024

Reporting Summary

Nature Portfolio wishes to improve the reproducibility of the work that we publish. This form provides structure for consistency and transparency in reporting. For further information on Nature Portfolio policies, see our Editorial Policies and the Editorial Policy Checklist.

Statistics

For all statistical analyses, confirm that the following items are present in the figure legend, table legend, main text, or Methods section.

n/a	Confirmed	
☐	☒	The exact sample size (n) for each experimental group/condition, given as a discrete number and unit of measurement
☐	☒	A statement on whether measurements were taken from distinct samples or whether the same sample was measured repeatedly
☐	☒	The statistical test(s) used AND whether they are one- or two-sided<br>*Only common tests should be described solely by name; describe more complex techniques in the Methods section.*
☐	☒	A description of all covariates tested
☐	☒	A description of any assumptions or corrections, such as tests of normality and adjustment for multiple comparisons
☐	☒	A full description of the statistical parameters including central tendency (e.g. means) or other basic estimates (e.g. regression coefficient) AND variation (e.g. standard deviation) or associated estimates of uncertainty (e.g. confidence intervals)
☐	☒	For null hypothesis testing, the test statistic (e.g. F, t, r) with confidence intervals, effect sizes, degrees of freedom and P value noted<br>*Give P values as exact values whenever suitable.*
☒	☐	For Bayesian analysis, information on the choice of priors and Markov chain Monte Carlo settings
☒	☐	For hierarchical and complex designs, identification of the appropriate level for tests and full reporting of outcomes
☒	☐	Estimates of effect sizes (e.g. Cohen's d, Pearson's r), indicating how they were calculated

Our web collection on statistics for biologists contains articles on many of the points above.

Software and code

Policy information about availability of computer code

Data collection	We collected from public repositories, namely NCBI-SRA (https://www.ncbi.nlm.nih.gov/sra), Ensemble-plants (https://plants.ensembl.org/index.html) and Solgenomics (https://solgenomics.net/) or (http://www.plabipd.de/portal/solanum-pennellii)
Data analysis	We used community repositories for the deployment of code (https://github.com/NAMlab/DeepCRE) and open source material for the development of code in Phyton 3.7 or newer and R4.3.2.

For manuscripts utilizing custom algorithms or software that are central to the research but not yet described in published literature, software must be made available to editors and reviewers. We strongly encourage code deposition in a community repository (e.g. GitHub). See the Nature Portfolio guidelines for submitting code & software for further information.

Data

Policy information about availability of data

All manuscripts must include a data availability statement. This statement should provide the following information, where applicable:

- Accession codes, unique identifiers, or web links for publicly available datasets
- A description of any restrictions on data availability
- For clinical datasets or third party data, please ensure that the statement adheres to our policy

The reference genomes sequence and annotations (A. thaliana, S. lycopersicum, S. bicolor and Z. mays) used for extraction of gene flanking regions and estimation of transcript profiles were downloaded from Ensembl plants database v52 (plants.ensembl.org) GCA_000001735.1 [https://plants.ensembl.org/Arabidopsis_thaliana], GCA_000188115.3 [https://plants.ensembl.org/Solanum_lycopersicum], GCA_000003195.3 [https://plants.ensembl.org/Sorghum_bicolor]

and GCA_902167145.1 [[https://plants.ensembl.org/Zea_mays]. Transcriptomic short-read data was downloaded from the National Center for Biotechnology Information (NCBI) Sequence Read Archive (SRA) database for leaf and root data from Bioprojects to determine transcript profiles PRJEB32665 [https://www.ncbi.nlm.nih.gov/bioproject/PRJEB32665], SRP010775 [https://trace.ncbi.nlm.nih.gov/Traces/?view=study&acc=SRP010775], PRJNA171684 [https://www.ncbi.nlm.nih.gov/bioproject/?term=PRJNA171684], [PRJEB22168 https://www.ncbi.nlm.nih.gov/bioproject/?term=PRJEB22168], PRJNA237342 [https://www.ncbi.nlm.nih.gov/bioproject/?term=PRJNA237342], PRJNA640858 [https://www.ncbi.nlm.nih.gov/bioproject/?term=PRJNA640858], PRJNA217523 [https://www.ncbi.nlm.nih.gov/bioproject/?term=PRJNA217523] and PRJNA271595 https://www.ncbi.nlm.nih.gov/bioproject/?term=PRJNA271595]. For the analyses of the fifteen Solanum genotypes, we used as reference sequences and annotations the Sol Genomics Network [https://solgenomics.net/ftp/genomes/]. For Solanum pennellii we used the accessions from Schmidt and colleagues (2017)83 [http://www.plabipd.de/portal/solanum-pennellii]. These reference datasets were processed as described in the methods section to generate results.

Human research participants

Policy information about studies involving human research participants and Sex and Gender in Research.

Reporting on sex and gender	not applicable
Population characteristics	not applicable
Recruitment	not applicable
Ethics oversight	not applicable

Note that full information on the approval of the study protocol must also be provided in the manuscript.

Field-specific reporting

Please select the one below that is the best fit for your research. If you are not sure, read the appropriate sections before making your selection.

☒ Life sciences ☐ Behavioural & social sciences ☐ Ecological, evolutionary & environmental sciences

For a reference copy of the document with all sections, see nature.com/documents/nr-reporting-summary-flat.pdf

Life sciences study design

All studies must disclose on these points even when the disclosure is negative.

Sample size	This study included four plant species: Arabidopsis thaliana, Solanum lycopersicum, Sorghum bicolor and Zea mays.<br>As raw data we have used short read transcriptomic data (RNA seq), required from the NCBI Sequencing Read Archive (SRA). For each species at least five SRA experiments per contidion (tissue-specifity) were obtained for our analyses. For each plant N models were generated and used for the prediction of gene expression levels, according to the number of the organisms chromosomes (5,12,10,10). These models were used for the analyses is this study.
Data exclusions	We did not exclude raw data after obtaining it from the NCBI SRA. All models were trained following the description within the Methods section or as described in the public repository https://github.com/NAMlab/DeepCRE.
Replication	Because we have used raw RNA-seq data obtained from public repository NCBI-SRA and genome annotations, for which we applied objective processing, our results can be replicated. The manuscript itself includes controls that confirm reproduceability. In addition, we provide full code (including versions) on community platform Github repository, the taken stages should be transparent.
Randomization	We included controls for each experiment that controlled covariates. For example, the training of models was performed for genes, excluding isoforms or randomized sequences to contol for batch effects.<br>Other experiments included random sampling for the normalization in unbalanced datasets avoiding effects due to under- or oversampling.
Blinding	Blinding of the investigators was not possible, nor necessary to this study, because deep learning model generation is difficult to manipulate independent of manipulation of the training data. The data used in this study has not been selected or processed specifically in any way. In addition, the authors are not aware of any sampling or research bias, or if so, have introduced controls accordingly.

Reporting for specific materials, systems and methods

We require information from authors about some types of materials, experimental systems and methods used in many studies. Here, indicate whether each material, system or method listed is relevant to your study. If you are not sure if a list item applies to your research, read the appropriate section before selecting a response.

Materials & experimental systems

n/a	Involved in the study
☒	☐ Antibodies
☒	☐ Eukaryotic cell lines
☒	☐ Palaeontology and archaeology
☒	☐ Animals and other organisms
☒	☐ Clinical data
☒	☐ Dual use research of concern

Methods

n/a	Involved in the study
☒	☐ ChIP-seq
☒	☐ Flow cytometry
☒	☐ MRI-based neuroimaging
